# Computable properties of selected monomeric acylphloroglucinols with anticancer and/or antimalarial activities and first-approximation docking study

**DOI:** 10.1007/s00894-025-06299-7

**Published:** 2025-03-12

**Authors:** Neani Tshilande, Liliana Mammino

**Affiliations:** Faculty of Science, Engineering and Agriculture, University of Venda, University Road, Thohoyandou, 0950 South Africa

**Keywords:** Acylphloroglucinols, Anticancers, Antimalarials, Molecular docking, Molecular properties

## Abstract

**Context:**

Malaria and cancer tend to become drug-resistant a few years after a drug is introduced into clinical use. This prompts the search for new molecular structures that are sufficiently different from the drugs for which resistance has developed. The present work considers eight selected acylphloroglucinols (ACPLs) with proven antimalarial and/or anticancer activities. ACPLs are compounds of natural origin structurally derivative from 1,3,5-trihydroxybenzene and characterized by the presence of an acyl group R–C = O. The selected ACPLs contain only one acylphloroglucinol moiety and are, therefore, monomeric ACPLs (also occasionally called “simple” ACPLs). They were studied computationally *in vacuo* and in-three-solvents with different polarities, using different levels of theory. The findings on molecular properties relevant to the understanding of biological activities align with previous studies, enhancing the reliability of predictions for molecules of the same class and providing insights into their behaviour in different environments. Structure-based virtual screening was used to study the interactions between these molecules and selected proteins known as relevant drug targets for antimalarial and anticancer activities; the screening showed that most of these ACPLs bind well with the selected proteins, thus being interesting for further studies. The results also suggest that most of these ACPLs have the potential for dual therapeutic applications (antimalarial and anticancer), offering a cost-effective drug development option. Furthermore, the ADME-T predictions indicated favourable pharmacokinetic properties for these ACPLs.

**Methods:**

Computational studies of the selected ACPLs were performed using Gaussian-09, *in vacuo* and in-three-solvents with different polarities. Three different levels of theory were used – Hartree Fock (HF), Density Functional Theory (DFT) with the B3LYP functional, and second order Møller-Plesset Perturbation Theory (MP2). HF and MP2 used a 6-31G(d,p) basis set, while DFT used a 6-31G + (d,p), for consistency with previous studies on ACPLs. The investigated molecular properties include conformational preferences, intramolecular hydrogen bonding patterns, HOMO–LUMO energy gap, dipole moments, as well as the solvent effect for the three considered solvents. Virtual screening was conducted using the Schrödinger suite, including Maestro 9.3 with GLIDE for docking and GlideScore for evaluating binding affinities. In addition, the QikProp tool provided ADME-T predictions for pharmacokinetic properties.

**Supplementary Information:**

The online version contains supplementary material available at 10.1007/s00894-025-06299-7.

## Introduction

The various forms of cancer (a disease involving fast multiplication of somehow mutated cells, forming growths within the body) are altogether a prime cause of death worldwide [1, 2]. Malaria (a mosquito-borne disease caused by protozoa of the *plasmodium* genus) is responsible for hundreds of thousands of deaths every year, above all from the *Plasmodium falciparum* protozoon [3]. While effective treatment is not yet available for some forms of cancer, it has been largely available for malaria. However, the parasite develops resistance to drugs few years after they enter in clinical use. Resistant parasites cease responding to those drugs; therefore, it becomes necessary to search for new molecules that can be active against resistant parasites, and it is extremely important that the identification of new effective molecules keeps the pace with the rate of resistance development, to maintain the possibility of treating malaria. Cancers too develop resistance to drugs, although at a slower rate than plasmodia.

The present work considers antimalarial and anticancer activities together because many compounds that are active against cancer are active against malaria and vice versa [e.g., [4–9]. This frequent double action may be useful in the search for new drugs against both diseases.

The work focuses on acylphloroglucinols (ACPLs) that have proven active against one of the two diseases or against both ([10–14], selecting a subclass identified by a specific characteristics of their molecular structures. A comprehensive review of the various ACPL subclasses exhibiting these activities is offered in [15]. ACPLs may be particularly interesting because they have not yet been used clinically against either disease and are therefore ‘new’ from a therapeutical point of view.

ACPLs are actually a broad class of compounds – mostly of natural origin – comprising molecules with a variety of biological activities, including antibacterial, antifungal, antioxidant, anticancer, antiplasmodial, antiviral, and others [10, 16]. Their potentialities for drug development have been extensively investigated, from the possibility of being lead structures for the treatment of degenerative diseases [11] to their possible abilities against the SARS-CoV-2 virus [17]. Parallelly, the search for feasible synthetic pathways has become more active [12, 18, 19].

**Fig. 1:**
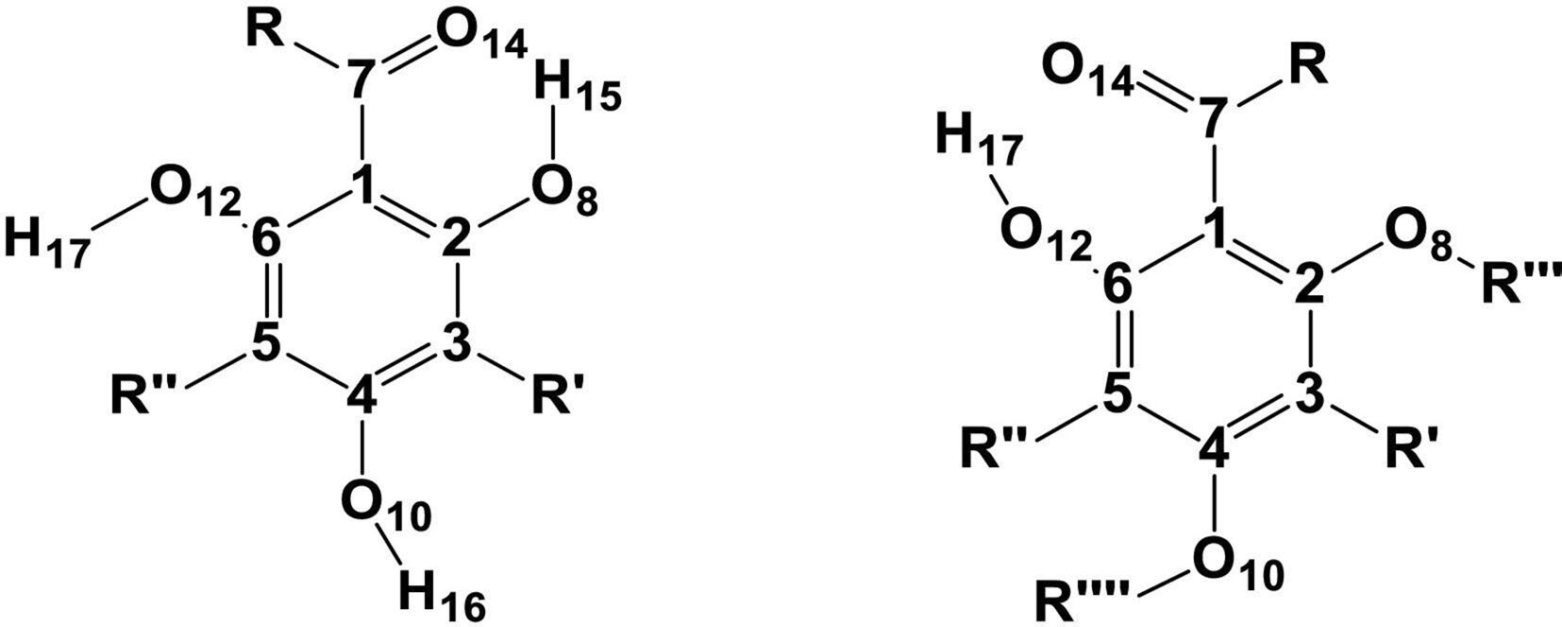
General structure of acylphloroglucinols in its more common form (left) and when the possibility of substituents at O8 and O10 is also considered (right), and atom numbering utilized in this work. The C atoms of the benzene ring are represented by their numbers. The first C atom in R is given the number C13, the first C atom in R’ is given the number C9, the first C atom in R" is given the number C11, the first C atom in R’" is given the number C15 and the first C atom in R"" is given the number C16

The ACPL molecules are structurally derived from phloroglucinol (1,3,5-trihydroxybenzene) with at least one acyl group, R–C = O, attached to the aromatic ring (Fig. 1). Biological activities depend on the finest details of the molecular properties of active compounds [20] and, therefore, it is important to know as much as possible about such properties. Previous studies of ACPLs have mainly focused on their conformational preferences and the factors influencing them [21]; the identification of intramolecular hydrogen bonds (IHBs) as the major stabilizing factors prompted specific studies of IHBs in ACPLs as a general class of compounds, in some subclasses [22–27], and also in selected molecules [28, 29]. The study of IHBs is particularly important for biologically active molecules because of their roles in processes such as selective binding, molecular recognition, and anticancer activity [30–32]. Studying individual structural features, or the structural features characterizing specific ACPL subclasses, and the roles of such features for biological activities, provides insights valuable for a better understanding of their various modes of action against pathogens [12].

Most drugs exert their therapeutic effects by interacting with a biological target (commonly a protein) in the human body or in the pathogen and inhibiting the activity that is crucial for the progress of the disease or the survival of the pathogen. The identification of suitable targets and the estimation of their interactions with an active molecule enable predictions of the pharmacological potentialities of that molecule. Computational approaches can model such interactions by docking the molecule to the target. Performing this operation on many molecules (virtual screening) enables the identification of promising molecules at a much faster rate than random experimental screening [33–37]. Then, the experimental work focuses on a comparative small number of promising molecules. The information about the interactions with the biological target is relevant for a variety of investigations, including the design of multi-target drugs – molecules or combinations of molecules that can simultaneously interact with more than one biological target [38–40].

**Fig. 2:**
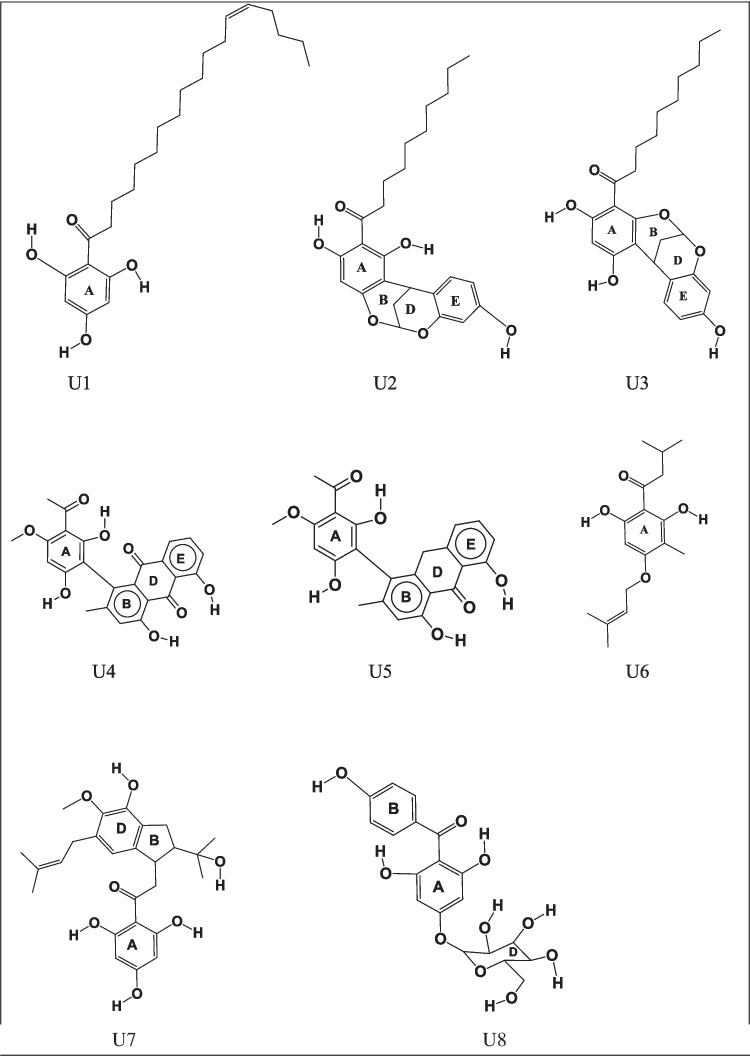
Molecular structures of the eight monomeric ACPL molecules considered in this study. The atoms of the phloroglucinol moiety are numbered as shown in Fig. 1. Only the atoms that are relevant for the analysis of the results are numbered individually, as shown in Fig. 3. The rings are denoted with uppercase letters (**A**, **B**, **D**, and **E**), with the letter A denoting the benzene ring of the phloroglucinol moiety

**Fig. 3:**
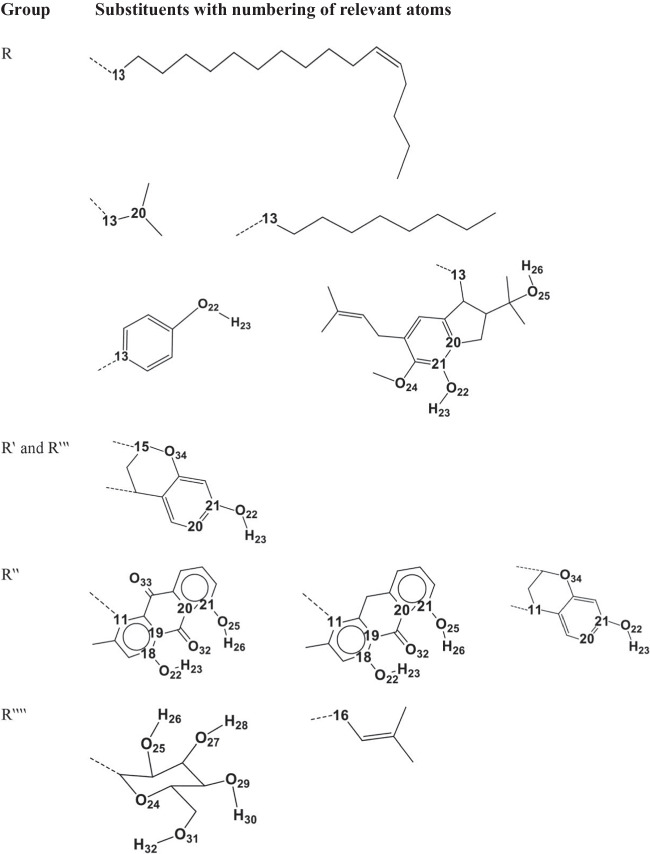
Atom numbering of the different R, R‵, R‵‵, R′″ and R‵‵‵‵ substituents appearing in the ACPL molecules shown in Fig. 2. The figure shows only the structures of the substituents, organised according to their positions (R, R‵, R‵‵, R′″, R‵‵‵‵). The first carbon atom in each substituent is numbered as explained in the caption of Fig. 1. The other atoms that are relevant for the analysis of the results are numbered as indicated in this figure. When there is only one OH group in the substituent, it is conventionally numbered as O22–H23. Additional OH groups are assigned different and conventional numbers

The current work focuses on eight monomeric ACPLs (ACPLs whose molecules contain only one acylphloroglucinol moiety, sometimes also called “simple” ACPLs) with proven antimalarial and/or anticancer activities. Table 1 lists them showing their common and IUPAC names, the acronyms utilised to denote them concisely in this work, and their proven activities. Figure 2 shows their structures. In the present text, the atoms of the acylphloroglucinol moiety are numbered as shown in Fig. 1 and the atoms of individual substituents, which are relevant for the analysis of the results, are numbered as shown in Fig. 3. In practice, only the atoms of the OH groups and the sp^2^ O atoms present in the substituents are numbered because they are the ones that can be involved in IHBs and other types of interactions; they are numbered in an independent way for each compound because of the substituents’ differences, and the numbers assigned to them are sufficiently high to prevent the risk of overlaps with the numbering of the acylphloroglucinol moiety. Table 2 summarises the nature of the substituents and the presence of OH groups and sp^2^ O atoms in the compounds, to offer an easy synopsis of their numbers and positions, in addition to the visualization offered by Fig. 2. It can also be noted that U2 and U3 are structural isomers.

**Table 1:** Common and IUPAC names of the ACPL molecules considered in this study, acronyms utilised to denote them concisely in this work, and reported biological activities

Common name of molecule	IUPAC name	Acronym denoting the molecule	Reported biological activities *	
Thouvenol A	(E)−1-(2,4,6-trihydroxyphenyl)octadec-13-en-1-one	U1	anticancer	
Myristicyclin A	1-(1,3,9-trihydroxy-12H-6,12-methanodibenzo[d,g][1, 3]dioxocin-2-yl)decan-1-one	U2		antimalarial
Myristicyclin B	1-(1,3,9-trihydroxy-12H-6,12-methanodibenzo[d,g][1, 3]dioxocin-4-yl)decan-1-one	U3		antimalarial
Knipholone	1-(3-acetyl-2,6-dihydroxy-4-methoxyphenyl)−4,5-dihydroxy-2-methylanthracene-9,10-dione	U4	anticancer	antimalarial
knipholone anthrone	4-(3-acetyl-2,6-dihydroxy-4-methoxyphenyl)−1,8-dihydroxy-3-methylanthracen-9(10H)-one	U5	anticancer	antimalarial
–	1-(2,6-dihydroxy-3-methyl-4-((3-methylbut-2-en-1-yl)oxy)phenyl)−3-methylbutan-1-one	U6		antimalarial
Antiarone J	2-(4-hydroxy-2-(2-hydroxypropan-2-yl)−5-methoxy-6-(3-methylbut-2-en-1-yl)−2,3-dihydro-1H-inden-1-yl)−1-(2,4,6-trihydroxyphenyl)ethanone	U7	anticancer	
Iriflophenone 4-glucoside	(2,6-dihydroxy-4-(((3S,4S,5R,6S)−3,4,5-trihydroxy-6-(hydroxymethyl)tetrahydro-2H-pyran-2-yl)oxy)phenyl)(4-hydroxyphenyl)methanone	U8	anticancer	

* All the activities mentioned here are reported in [1]. The following additional references refer to individual compounds: [41] for U1; [42] for U2; [43] for U3; [44] for U4; [45] for U5; [46] for U6; [47] for U7; [48] for U8

(-) means that there is no reported common name for the given compound

**Table 2:** Nature of the R, R’, R", R’’’, R"" substituents and presence of OH groups and sp^2^ O in the molecules of the acylphloroglucinols considered in this study

Acronym	R, R’, R", R’’’, R"" substituents	OH groups	sp^2^ O
U1	R = (CH_2_)_11_C = C(CH_2_)_3_CH_3_	O8H15, O10H16, O12H17	O14
U2	R = (CH_2_)_8_CH_3_ Terpene moiety attached at C4-C5	O8H15, O12H17, O22H23,	O14
U3	R = (CH_2_)_8_CH_3_ Terpene moiety attached at C2-C3	O10H16, O12H17, O22H23	O14
U4	R = R’’’ = CH_3_ R" = an anthraquinone moiety	O10H16, O12H17, O22H23, O25H26	O14, O32, O33
U5	R = R’’’ = CH_3_ R" = an anthraquinone moiety	O10H16, O12H17, O22H23, O25H26	O14, O32
U6	R = isobutyl, R’ = CH_3_ R’’" = prenyl	O8H15, O12H17	O14
U7	R = dihydrobenzofuran	O8H15, O10H16, O12H17, O22H23, O25H26	O14
U8	R = phenol R’’" = glucoside	O10H16, O12H17, O22H23, O25H26, O27H28, O29H30, O31H32	O14

The atoms of the phloroglucinol moiety are numbered as shown in Fig. 1. Only the atoms that are relevant for the analysis of the results are numbered individually, as shown in Fig. 3. The rings are denoted with uppercase letters (A, B, D, and E), with the letter A denoting the benzene ring of the phloroglucinol moiety.

Computational studies were performed on these compounds, both *in vacuo* and in three suitably selected solvents, to identify their molecular properties, including conformational preferences, IHB patterns, HOMO–LUMO energy gap, dipole moments, and solvent effects for the studies in solution. In addition, preliminary docking studies were performed with selected target proteins, to investigate the details of the interactions between these molecules and their possible biological targets, to evaluate their perspectives as potential drugs and to recognize the most promising ones.

The conformational results confirm the dominant stabilising roles of IHBs and highlight trends in molecular properties, including the ways in which they are influenced by the medium. The docking studies show that most of these compounds bind well to the target proteins, and their interactions compare well with those of recognised antimalarial or anticancer compounds. Most of them also show good ADMET (administration, distribution, metabolism, excretion, toxicity) profiles, which is crucial for the identification of promising potential drugs.

The presentation of the results is substantiated by tables and figures that can be suitably included in the text. In addition, tables and figures presenting all the obtained numerical values and all the obtained optimised geometries are included in the Supplementary Information, and references to them are made in the text, with their numbers preceded by an S for the sake of easy identification.

## Computational details

### Approaches for the conformational studies

The work presented here is part of a much broader computational study of ACPLs, which also included [21–29] and various other works. The same computational approaches as in the previous works are here selected to ensure the possibility of meaningful comparisons with the results of those works. The approaches enable a reasonable balance between result quality and computational cost, also in consideration of the high number of molecules, their non-small size, and the frequent high number of possible conformers for each molecule in some ACPL subclasses (e.g., [48, 49]). In addition, it is opted to use more than one level of theory to have an intrinsic verification of obtained results and inferred trends, as different methods may yield better evaluations of certain properties and weaker evaluations of others, while the comparison of patterns provides verification of inferred trends.

The selected levels comprise two ab initio methods {Hartree–Fock (HF) and second-order Møller-Plesset Perturbation Theory (MP2, [50])} and Density Functional Theory (DFT). Of these, HF is the least expensive method, while MP2 is the most costly. HF and MP2 calculations utilized the 6-31G(d,p) basis set. Previous studies (e.g., [21–29]) had shown that HF/6-31G(d,p) calculations provide reasonable results for ACPLs, above all for comparison-based trends identification; the results’ reasonability extended to the description of IHBs. It is used here because it is always interesting to verify the performance of a less expensive method for the study of a newly-considered subclass of ACPLs. Furthermore, the use of the 6-31G(d,p) basis set for MP2 calculations was necessary because of the high computational costs of larger bases, above all for the larger molecules.

DFT calculations utilized the commonly-employed B3LYP functional [50–52] and the 6–31 + G(d,p) basis set. Previous studies (e.g., [21–29]) had shown the importance of diffuse functions on the heavy atoms for the quality of DFT results for ACPLs. A more general study [53] had shown the importance of diffuse functions on heavy atoms, above all for the description of properties related to molecules’ polarization. Although B3LYP does not take into account electron correlation to a great extent [54], it was decided not to add dispersion corrections because it had not been added in the previous general type studies on ACPLs (e.g., [21–25]) and because the parallel utilization of MP2 calculations provides results where correlation and dispersion effects are incorporated.

For the sake of conciseness, and in the evident absence of any risk of confusion, these calculation methods are denoted by acronyms in the rest of the text: DFT for DFT/B3LYP/6-31G + (d,p), HF for HF/6-31G(d,p), and MP2 for MP2/6-31G(d,p).

Calculations in solution were carried out at the HF and DFT levels utilising the conductor-like polarizable continuum model (CPCM, [55, 56]).

Vibrational frequencies (harmonic approximations) were computed at the HF and DFT levels on the respective optimized geometries, with the computed values scaled by factors of 0.9632 for DFT results and 0.8992 for HF results [57]. The absence of imaginary frequencies confirms the true-minima character of the obtained stationary points. Frequency calculations also provide energetics values related to vibrational motions, such as the zero-point energy (ZPE) corrections, the energy values corrected for ZPE, and the free energy values. Changes in the IR vibrational frequency of the OH groups acting as hydrogen bond donors enable comparisons of hydrogen bond strengths.

All conformational calculations were conducted with fully relaxed geometry, both *in vacuo* and in solution – re-optimization in solution being important to identify conformational changes induced by the solvent. The HF-optimized geometries served as input geometries for DFT and MP2 calculations. The calculations were performed using Gaussian-09, Revision E.01 [58]. The visualization of the input and output geometries utilised Gauss View 4.1.2 [59], Chem3D Ultra and ChemDraw Ultra [60].

All the energy values included in this work are in kcal mol^−1^, and the distances are in Ångströms (Å).

### Approaches for the docking studies

#### General features

Molecular docking investigates the interactions between a given active molecule (ligand) and the proteins’ active site (receptor) that the ligand is expected to inhibit, thus slowing down or blocking the progress of the disease. It can be conducted at various levels. The best approximation to what may actually happen within the human body would consider both the ligand and the protein (at least its receptor area) as flexible [61–67], as well as the explicit presence of water molecules that might facilitate the ligand–protein interactions [68–74]. The inclusion of these components depends on the utilised software and on the objectives of the study.

In the current study, the Schrödinger [75] suite of programs was employed, using the standard ligand docking procedure in Maestro 9.3 (which is part of the Schrödinger suite). GLIDE (Grid-based Ligand Docking with Energetics) was used, which typically treats the protein as a rigid entity and allows flexibility only for the ligand, exploring various conformations and orientations to find its optimal binding mode with the protein’s site. Explicit water molecules are usually removed in the standard procedure, to simplify the system, although bridging waters crucial for some of the interactions can be retained through manual selection within the receptor grid generation. Since the current study is meant as a first approximation (preliminary) exploration, explicit water molecules were omitted. Both features (rigidity of the protein and omission of explicit water molecules) constitute limitations that will be removed in a future study, after preliminary docking results for all the subclasses of anticancer and/or antimalarial ACPLs are obtained.

The overall docking procedure entails various components: ligand preparation, protein preparation, receptor grid generation and the actual ligand-receptor docking. They are briefly described in the next subsections.

#### Ligands’ preparation

For each molecule, the lowest energy conformer obtained from the DFT calculation was selected as ligand for the docking studies. The relevant characteristics predicted by the DFT calculations were maintained without alteration, i.e., the ionization states of the ligands were maintained, no tautomers different from the one utilised for the DFT calculations were generated, and no different chiralities were generated.

#### Preparation of the proteins

The X-ray crystal structures of proteins that are considered suitable biological targets for molecules with anticancer or antimalarial activities were obtained from the Protein Data Bank, PDB [76]. The Maestro 9.3 protein preparation wizard application [77], which is part of the Schrödinger suite of programs, was utilized to refine these “raw” PDB structures and make them ready for the docking procedure. Depending on the protein, the “refining” involves adjustments such as adding hydrogen atoms, assigning bond orders, creating zero-order bonds to metals, forming disulphide bonds, adjusting charges, and orienting groups.

#### Receptor grid generation

GLIDE uses a series of hierarchical filters to search for possible locations suitable for ligand binding within the binding-site region of a receptor, allowing different possible conformations of the ligand for different locations. The shape and properties of the receptor are represented on a grid by different sets of fields, providing progressively more accurate scoring of the ligand pose [78].

A grid file was generated from the prepared protein using the Maestro 9.3 protein preparation wizard. The option for *Pick to Identify the Ligand (Molecule) and Show Markers* was selected in the *Define Receptor* section. Subsequently, an atom within the co-crystallised ligand was selected to help define the centre of the active site of the receptor. The scaling factor and partial charge cut-off for van der Waals radius scaling were set to 0.25 and 1 Å, respectively. Other features such as sites, constraints, rotatable groups, and excluded volume were maintained at their default settings in Maestro 9.3 [79, 80].

#### The GLIDE molecular docking procedure

The GLIDE XP visualizer module was utilised to analyse the ligand–protein interactions of the considered ACPLs against the proteins selected as suitable antimalarial or anticancer targets. GLIDE utilizes a computational simulation approach to systematically search for favourable interaction regions, i.e., for particular locations of the ligand in the active-site of the protein, which correspond to best interactions [81]. It uses scoring functions to classify compounds on the basis of their binding strength, with greater binding strength corresponding to greater activity (compounds that bind weakly or do not bind are inactive) [78]. The empirical GlideScore scoring function comprises terms responding to the characteristics of the given interaction, such as lipophilic-lipophilic terms, hydrogen bond terms, and various others, and also terms accounting for the displacement of water molecules by a ligand from mainly lipophilic areas [82, 83]. The binding scores enable comparisons of the suitability of different ligands against the same target.

### ADME-T properties

The ADME properties relate to what happens to a drug within the human body: administration, distribution, metabolism and excretion. They are predicted through parameters (descriptors). Their prediction is important because compounds with poor ADME properties are not viable as drugs. The T in ADME-T stands for toxicity – a property that also determines the viability of a drug: toxicity needs to be as low as possible, and remain below levels recognised as acceptable.

The QikProp tool [84] included in Schrödinger 2012 can predict a variety of pharmaceutically relevant properties. The following properties related to molecules’ permeability and absorption [85, 86] were calculated for the ACPLs considered in this work: the predicted apparent permeability (QPPCaco, nm/sec), with Caco-2 cells being a line of human epithelial colorectal adenocarcinoma cells commonly used as model for the gut/blood barrier; the predicted apparent permeability across MDCK (Madin-Darby Canine Kidney) cells (QPPMDCK, nm/sec), with MDCK cells being a good model for the blood/brain barrier; the predicted blood–brain partition coefficient (QPlogBB); the predicted binding capacity to human serum albumin (QPlogKhsa); the percent predicted human oral absorption (%PHOA).

## Results

### Results *in vacuo*

#### Naming of conformers

**Fig. 4:**
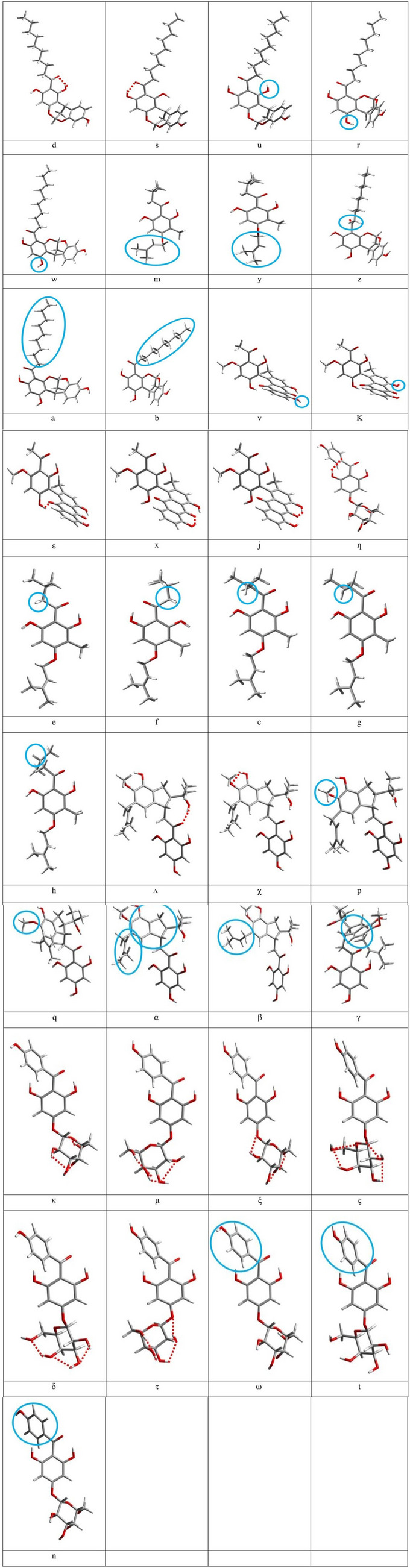
Illustrations of the characteristics that are represented by letters in the acronyms denoting the calculated conformers of the ACPL molecules considered in this work. The characteristics and the corresponding letters are listed in Table 3. For better visualization, blue circles highlight the parts concerned with the given characteristic, and red dashed segments represent the considered IHBs

Following a practice introduced since the initial studies of ACPLs [21–29], conformers are concisely denoted with acronyms whose individual letters indicate each of the relevant geometry characteristics. The characteristics include the presence of O–H⋅⋅⋅O IHBs (e.g., H15⋅⋅⋅O14, H17⋅⋅⋅O14, H23⋅⋅⋅O32, H26⋅⋅⋅O32, H26⋅⋅⋅O14, H16⋅⋅⋅O33, etc.) and of O–H⋅⋅⋅π interactions, the orientation of the OH groups, the orientation of the substituents (including ring systems) relative to moiety A, and the position of C13 relative to the plane of ring A. The symbols used to represent each of these geometry features are listed in Table 3 and their meaning is also illustrated in Fig. 4.

**Table 3:** Symbols utilised to specify the geometric features of individual conformers in the acronyms denoting molecules and conformers

Moiety or molecule considered	Feature category	Symbol	Meaning of symbol
Acylphloroglucinol moiety	Presence and position of the first IHB	d	the H15⋅⋅⋅O14 first IHB is present
		s	the H17⋅⋅⋅O14 first IHB is present
	Orientation of OHs *ortho* to acyl group	–	if engaged in the first IHB, indicated by *d* or *s*
		–	if not engaged in the first IHB and oriented away from the acyl group, no indication is given
		u	if not engaged in the first IHB and oriented toward the acyl group
	C3 − C4 − O10 − H16 (or C16 if R"" is present) torsion angle	r	the angle is close to 0^o^
		w	the angle is close to 180^o^
		m	the angle is close to + 90° (‘’towards us’’)
		y	the angle is close to −90^o^ (‘’away from us’’)
	Orientation of O14 when not engaged in the first IHB	z	oriented off-plane with respect to the plane of ring A, and away from us
General	absence of IHB		no IHB-related symbol is present
U1, U2 and U3	Orientation of R with respect to ring A	a	R (including C13) is coplanar to ring A
		b	R (including C13) is off-plane with respect to ring A and “towards us”
U2, U3, U4 and U5	C19-C18-O22-H23 torsion angle	v	the angle is close to 180^o^
	C20-C21-O25-H26 torsion angle	k	the angle is close to 180^o^
U4 and U5	Presence and position of other IHBs	ε	the H16··O33 IHB is present (U4)
		x	the H23⋅⋅⋅O32 IHB is present (U4, U5)
		j	the H25⋅⋅⋅O32 IHB is present (U4, U5)
U6	Orientation of the isobutyl group	e	oriented to the other side with respect to O14
		f	oriented towards O14
		c	oriented “towards us”, with the H atom attached to C20 oriented towards O14
		i	oriented “towards us”, with the H atom attached to C20 oriented away from O14
		g	oriented “away from us”, with the H atom attached to C20 oriented towards O14
		h	oriented “away from us”, with the H atom attached to C20 oriented away from O14
U7	Presence and position of other IHBs	Λ	the H26···O14 IHB is present
		χ	the H23···O24 IHB is present
	Orientation of O22 − H23	λ	oriented away with respect to O24
	C19-C18-O22-H23 torsion angle	p	the angle is close to + 90°
		q	the angle is close to −90^o^
	Orientation of the B-D ring system and the prenyl chain	α	ring system oriented away with respect to O14; prenyl chain oriented “towards us”
		β	ring system oriented away with respect to O14; prenyl chain oriented “away from us”
		γ	ring system oriented towards O14
U8	IHBs present in ring D	ƞ	H17···π (C13)
		κ	O27–H28···O29, O29–H30···O31, O31–H32···O24
		μ	O25–H26···O10, O27–H28···O25, O29–H30···O27, O31–H32···O29
		ξ	O25–H26···O10, O27–H28···O29, O29–H30···O31, O31–H32···O24
		ς	O25–H26···O10, O27–H28···O25, O29–H30···O31, O31–H32···O24
		δ	O25–H26···O27, O27–H28···O29, O29–H30···O31
		τ	O27–H328···O25, O29–H30···O27, O31–H32···O29
	Orientation of ring B and O22 − H23	ω	ring B oriented “towards us”; O22 − H23 oriented “downwards”
		t	ring oriented “towards us”; O22 − H23 oriented “upwards”
		n	ring oriented “away from us”; O22 − H23 oriented “downwards”

The symbols for the characteristics of the acylphloroglucinol moiety are maintained from previous works on ACPLs, for the sake of consistency. It may be interesting to recall the structure-related definitions of some of them. When there are different substituents in *meta* to the acyl group, the C atoms of the ring are numbered in such a way that the larger substituent is attached at C3; then, “d” is the first IHB forming on the same side as the substituent at C3 (H15⋅⋅⋅O14) and “s” is the first IHB forming on the other side with respect to this substituent (H17⋅⋅⋅O14); “r” indicates that O10 − H16 is oriented towards the side of the substituent at C3, and “w” indicates that it is oriented towards the other side. In this way, the difference between “d” and “s” relates to the presence of different substituents at C3 and C5, and is more significant when there is a substituent ≠ H at C3 and H at C5 [21, 22]. When there are no substituents ≠ H at C3 and C5, the ensuing symmetry cancels the difference; in these case, it has been conventionally opted to use the letter “d” for the first IHB, and there are no “s” IHBs; then, “r” indicates that O10 − H16 is oriented towards the side of the first IHB and “w” indicates that it is oriented towards the other side. Characteristics, specific of the substituents in individual ACPLs, are assigned other letters or symbols (letters or symbols different from those used for the acylphloroglucinol moiety).

#### Geometrical characteristics of the conformers

**Fig. 5:**
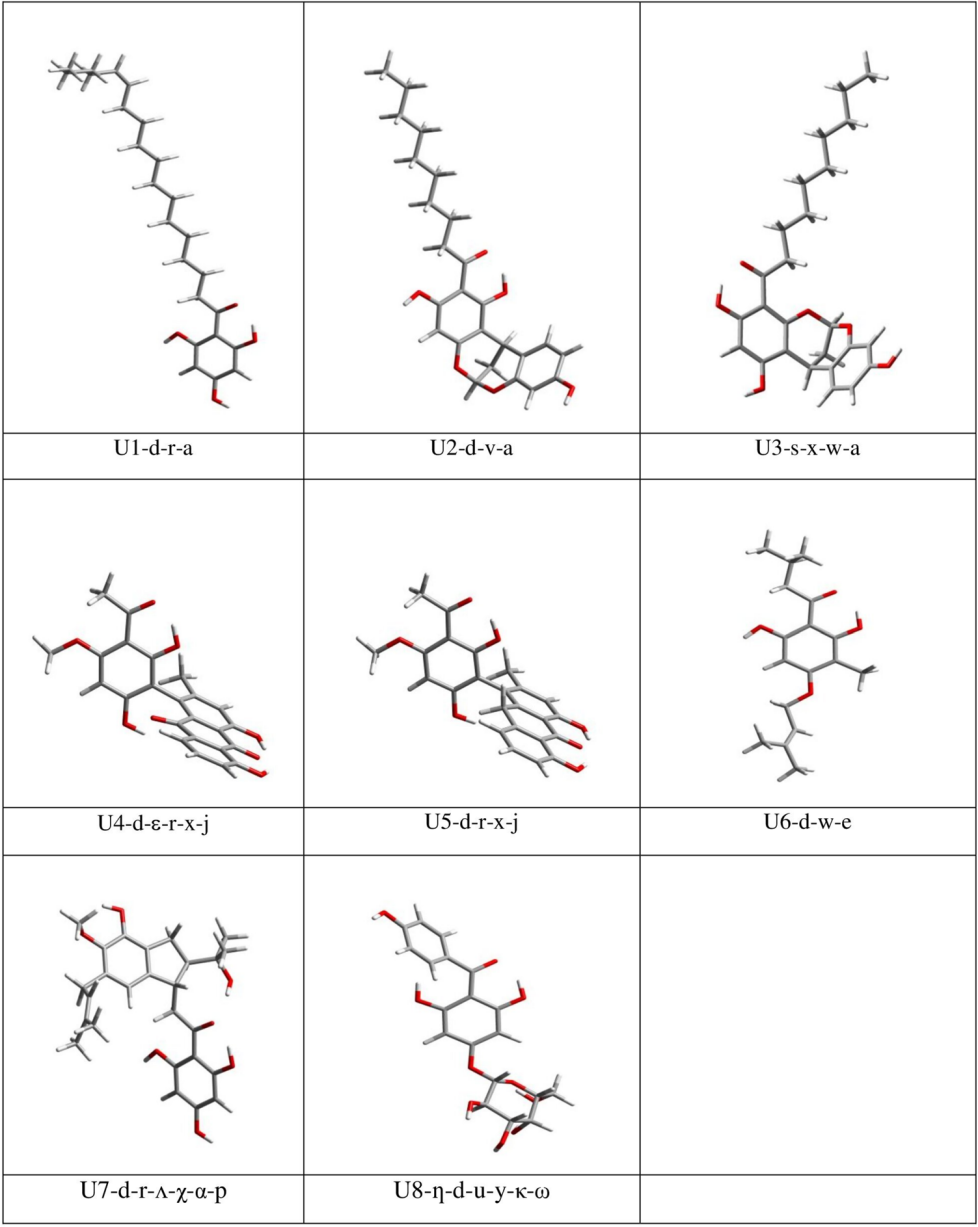
The lowest energy conformer of each of the considered ACPL molecules. DFT results *in vacuo*

The optimized geometries of the lowest energy conformer of each of the considered ACPL molecules are shown in Fig. 5, and the geometries of all the calculated conformers are shown in Figure S1.

Like in the ACPLs studied in previous works, the dominant feature determining conformational preferences is the formation of IHBs. The sp^2^ O of the acyl group (O14) can form an IHB with either of the *ortho* OHs, resulting in H15⋅⋅⋅O14 or H17⋅⋅⋅O14; they are both termed “first IHB” and distinguished by the letters *d* or *s* in the conformers’ acronyms. Previous studies had shown that they are moderate H-bond bordering on strong [22]. They cannot form when the H15 or H17 is replaced by other functions (e.g., H15 in U3, U4, U5).

Additional O–H⋅⋅⋅O IHBs can form if the substituents contain additional donors or acceptors. Both U4 and U5 contain an sp^2^ O (O32) in the intermediate D ring of the B-D-E ring system, and U4 contains an additional one (O33). O32 can form IHBs with H23 and H26 simultaneously (cooperative H23⋅⋅⋅O32 and H26⋅⋅⋅O30, with bifurcation on O32) or with only one of them. In U4, O33 can form IHBs with H16 whereas it remains too distant (3.9 Å) from H17 even when the H17⋅⋅⋅O14 IHB is not present and H17 is oriented toward it. In U7, H25 can form an IHB with O14, which would be cooperative with H17⋅⋅⋅O14 when the latter is also present.

Although being somewhat weaker than IHBs with an sp^2^ O acceptor, O–H⋅⋅⋅O IHBs with an sp^3^ O acceptor have considerable stabilising roles [23]. The glucoside moiety of U8 shows the highest variety of possible IHBs of this type, namely, O25–H26···O10, O25–H26···O27, O27–H28···O29, O27–H28···O25, O29–H30···O31, O29–H30···O27, O31–H32···O24, and O31–H32···O29; up to four of them can be present simultaneously, and are consecutive and cooperative.

O–H⋅⋅⋅π interactions have been recognised as hydrogen bonds since long, above all because of their roles in biomolecules [87, 88] and have continued being objects of attention [e.g., [89]). Their roles for the conformational preferences of ACPLs have been analysed in [24], and further highlighted for individual ACPL molecules (e.g., [28]). O8 − H15 and O12 − H17 can form this type of IHB with ring B in U8.

**Fig. 6:**
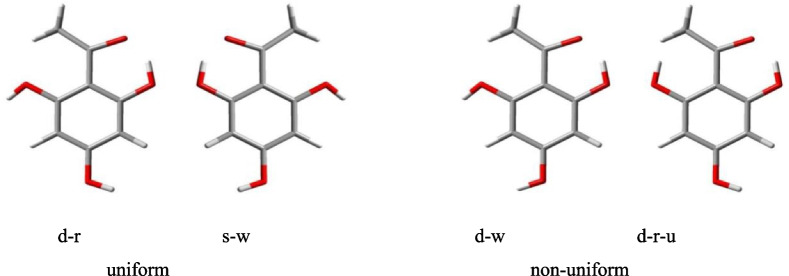
Examples of uniform and non-uniform orientation of the OH groups of the acylphloroglucinol moiety

The orientations of the OH groups also influence the energy of the conformers and are, therefore, specified in their acronyms (Table 2). When they are engaged in the first IHB, the orientation of O8–H15 or O12–H17 is automatically known (towards O14); when they are not engaged in the first IHB, only the higher energy orientation (towards the acyl group) is specified (letter *u*). The OHs of the acylphloroglucinol moiety tend to be coplanar to the benzene ring [21, 90]; from the point of view of rotational symmetry, they prefer uniform orientation (C_3v_ symmetry, Fig. 6).

The orientations of the substituents with respect to the acylphloroglucinol moiety may also influence conformational preference and energy, above all in the cases in which some orientations favour interactions – such as IHBs – between the two.

The optimised geometries largely resemble the input geometries, with the phenomena already observed for ACPLs in general. For instance, since R ≠ H in all the considered compounds, when O14 is not engaged in first IHB and is coplanar to the ring in the input, the acyl group rotates on optimization, so that O14 becomes off-plane (although not perpendicular to it) as a way of smoothing repulsion with O8 and O12 [21, 22].

#### Conformers’ relative energies and factors influencing them

The relative energies of the four lowest energy conformers of each molecule are shown in Table 4 and those of all the calculated conformers are reported in Table S1.

**Table 4:** Relative energies of the four lowest energy conformers of the calculated ACPL molecules *in vacuo*

Molecules and conformers	Relative energy (kcal mol^−1^)		
	DFT	HF	MP2
U1-d-r-a	0.000	0.000	0.000
U1-d-w-a	1.380	1.516	1.402
U1-d-u-r-a	2.879	4.373	2.820
U1-d-u-w-a	3.229	4.785	3.189
U2-d-v-a	0.000	0.041	0.051
U2-s-v-a	0.080	0.000	0.000
U2-s-v-u-a	4.178	5.019	3.165
U2-d-x-a	4.327	5.822	4.096
U3-s-x-w-a	0.000	0.000	3.334
U3-s-v-w-a	0.030	0.135	3.491
U3-s-x-w-b	0.534	1.024	0.000
U3-s-x-r-a	3.135	3.994	5.772
U4-d-ε-r-x-j	0.000	0.000	0.000
U4-d-w-x-j	2.091	2.669	3.410
U4-d-ε-r-v-j	12.259	10.941	11.408
U4-d-ε-r-x-k	12.923	11.510	12.342
U5-d-r-x-j	0.000	0.000	0.000
U5-d-w-x-j	3.873	4.058	4.286
U5-d-r-v-j	12.880	11.948	11.683
U5-d-r-x-k	13.489	12.498	12.124
U6-d-w-e	0.000	0.000	0.461
U6-d-w-g	0.418	0.889	0.000
U6-d-w–c	0.443	0.887	0.000
U6-s-w–f	1.133	1.064	1.320
U7-d-r-Λ-χ-α-p	0.000	0.000	0.000
U7-d-w-Λ-χ-α-p	1.325	1.466	1.321
U7-d-w-Λ-χ-α-q	1.561	1.589	2.252
U7-d-w-Λ-χ-β-p	1.845	1.815	0.150
U8-ƞ-d-u-y-κ-ω	0.000	0.951	0.019
U8-ƞ-d-u-y-κ-t	0.000	1.038	0.000
U8-ƞ-d-u-w-μ-t	1.213	0.000	1.416
U8-d-y-κ-ω	1.378	1.607	3.948

HF/6-31G(d,p), DFT/B3LYP/6–31 + G(d,p) and MP2/6-31G(d,p) results from full optimisation calculations, respectively denoted as DFT, HF and MP2 in the columns’ headings. For each molecule, the conformers are listed in order of increasing relative energies in the DFT results

Comparisons of conformers differing only by one characteristic provide indications about the stabilising roles of that characteristic. The acronyms facilitate this comparison. For instance, the U2-s-v-a and U2-d-v-a acronyms indicate that the two conformers are suitable for comparison of the effects of the two first IHBs (H15⋅⋅⋅O14 and H17⋅⋅⋅O14) because they only differ by the position of the first IHB (letters *s* and *d*); the acronyms U2-s-v-a and U2-s-v-u-a indicate that the two conformers are suitable for comparison of the effect of the different orientations of O8–H15 when not engaged in the first IHB (away from the acyl group for U2-s-v-a and towards the acyl group for U2-s-v-u-a); comparison between U2-d-v-a and U2-x-a informs about the energy increase on removal of the H15⋅⋅⋅O14 IHB. These comparisons also highlight the extent of consistency with the findings from previous studies on ACPLs; for instance, the comparison of U2-s-v-a and U2-s-v-u-a (and other similar pairs in all the considered compounds) shows that the results for these molecules are consistent with the general trends identified for ACPLs, namely, that the orientation towards the acyl group entails higher energy [21, 22].

Compounds U2 and U6 are the only ones for which it is possible to compare the effects of the presence of H15⋅⋅⋅O14 or H17⋅⋅⋅O14, because conformers with either of them are possible. The relative energy values indicate that the energy of *d* conformers is slightly lower than the energy of corresponding s conformers, consistently with the results obtained for all the previously-studied ACPLs.

The values of the relative energies corrected for zero-point energy (ZPE), the ZPE corrections, the relative Gibbs free energies (ΔG_corrected_, sum of electronic and thermal free energies), and its correction, G_corr_, for all the calculated conformers of the considered ACPL molecules, are reported in Table S2 for the DFT results and in Table S3 for the HF results. The ranges of the values of the ZPE corrections and the corrections to the free energies are shown in Table 5.

**Table 5:** Ranges of the ZPE correction to the electronic energy (ZPE_corr_) and of the thermal correction to the Gibbs free energy (G_corr_) for the calculated conformers of the molecules considered in this work

Molecule	ZPE_corr_ (kcal mol^−1^)		G_corr_ (kcal mol^−1^)	
	DFT	HF	DFT	HF
U1	364.29–365.30	390.82–391.52	319.07–323.08	347.40–347.87
U2	319.69–320.77	343.41–344.79	278.46–281.20	303.12–305.93
U3	319.82–321.16	343.55–345.14	279.04–281.47	303.55–305.82
U4	235.02–236.20	252.57–255.20	195.93–199.65	215.37–219.64
U5	246.75–247.93	266.03–267.48	209.70–212.11	229.82–232.10
U6	238.09–239.21	255.56–256.95	203.17–205.23	221.04–223.41
U7	337.66–338.40	363.49–364.33	294.51–296.26	321.50–323.35
U8	238.86–239.73	259.03–260.07	201.38–203.68	221.53–225.11

DFT/B3LYP/6–31 + G(d,p) and HF/6-31G(d,p) results in vacuo from full optimization calculations, respectively denoted as DFT and HF in the columns’ headings

The relative energies corrected for ZPE and the uncorrected ones show similar trends, with some exceptions (e.g., U7-d-w-Λ-λ-α-p at the DFT level, and U8-ƞ-d-u-y-κ-t, U8-ƞ-d-u-w-μ-t at the HF level). In most cases, conformers with higher relative energy also have higher relative Gibbs free energy. Both the ZPE corrections and the corrections to G are relatively close for all the conformers of the same molecule but differ for different molecules. The greatest corrections pertain to the conformers of U1 and the smallest to those of U4.

Figure S2 compares the trends of the relative energies − both uncorrected and corrected for zero-point energy (ZPE) − for the calculated conformers of each molecule, in the results of the computational methods utilised. All the diagrams display a slow-gradient lower-energy region, which corresponds to the conformers with the first O–H⋅⋅⋅O IHBs. A sharp increase to a high energy region corresponds to the few calculated conformers without IHBs.

#### Characteristics of the intramolecular hydrogen bonds

The strength of IHBs can be compared by comparing their parameters (H⋅⋅⋅O bond length, O⋅⋅⋅O distance, OĤO angle), the energy increase when one of them is removed and no new one is formed, and the decrease in the IR vibrational frequencies of the donor OHs. Table S4 reports the parameters of the IHBs present in the calculated conformers of each considered molecule, and Table 6 shows the ranges of their lengths. In the case of O − H⋅⋅⋅π IHBs, the acceptor is not an individual atom but a π electron cloud; the length of the distance between H and the closest atom in the π system is reported in the tables, to enable comparisons.

**Table 6:** Ranges of the lengths of the intramolecular hydrogen bonds in the calculated conformers of the considered ACPL molecules *in vacuo*

Molecules	Considered IHB	Range of the length of the IHB (Å)		
		DFT	HF	MP2
U1	H15···O14	1.563–1.584	1.692–1.719	1.615–1.652
U2	H15···O14	1.545–1.546	1.672–1.673	1.599–1.600
	H17···O14	1.570–1.588	1.696–1.733	1.630–1.661
U3	H17···O14	1.545–1.565	1.681–1.697	1.615–1.667
U4	H15···O14	1.539–1.547	1.665–1.671	1.607–1.649
	H23···O32	1.615–1.654	1.766–1.785	1.682–1.707
	H25···O32	1.684–1.694	1.800–1.814	1.712–1.727
U5	H15···O14	1.536–1.541	1.666–1.670	1.590–1.595
	H23···O32	1.609–1.660	1.747–1.760	1.687–1.697
	H25···O32	1.627–1.682	1.767–1.783	1.699–1.714
U6	H15···O14	1.538–1.585	1.663–1.722	1.607–1.649
	H17···O14	1.563	1.691	1.614
U7	H15···O14	1.565–1.601	1.703–1.746	1.612–1.683
	H26···O14	1.923–1.979	2.057–2.128	1.963–2.838
U8	H15···O14	1.627–1.656	1.808–1.856	1.712–1.751
	H32···O10	2.535–2.632	2.535–2.632	2.415–2.521
	H32···O28	2.416–2.430	2.441–2.452	2.335–2.351
	H33···O27	2.353–2.402	2.371–2.408	2.306–2.340
	H33···O29	2.183–2.402	2.209–2.408	2.134–2.340
	H34···O28	2.202–2.219	2.227–2.233	2.135–2.151
	H34···O31	1.898–2.219	1.947–2.233	1.861–2.151
	H35···O29	1.992–2.617	2.036–2.429	1.946–2.269
	H17···π (C13)	2.172–2.193	2.301–2.638	2.144–2.161

DFT/B3LYP/6–31 + G(d,p), HF/6-31G(d,p) and MP2/6-31G(d,p) results from full optimisation calculations, respectively denoted as DFT, HF and MP2 in the columns’ headings. The molecules are denoted with the symbols listed in Table 1

For the first IHB, the parameters suggest that it falls within the moderate-bordering-on-strong range, similarly to the findings for other ACPLs [22]. The facts that it closes a 6-member ring and that it is a resonance-assisted hydrogen bond [91–94] contribute to its strength [22]. Other trends are also consistent with those already identified for other ACPLs. For instance, H15⋅⋅⋅O14 is somewhat shorter than H17⋅⋅⋅O14, and each of them is somewhat longer in u-conformers than in corresponding conformers in which the other *ortho* OH is oriented away from the acyl group.

The nature of R also influences the length of the first IHB, both through steric factors and in the cases when R can interact with O8 − H15 or O12 − H17. For instance, in u-type conformers of U8, the first IHB is longer than in the other compounds because R is a phenol and the *ortho* OH not engaged in the first IHB can interact with it through an O–H⋅⋅⋅π IHB; as a consequence, the acyl group is drawn towards the OH not engaged in the first IHB, what increases the length of the first IHB. In U7, R contains an OH group (O25 − H26) which can form an IHB with O14; when this happens (Λ-conformers), the H26⋅⋅⋅O14 IHB is consecutive to the first IHB (bifurcation on O14) and somewhat *pulls* O14, resulting in a comparatively longer first IHB; the first IHB remains the stronger one, because it closes a 6-member ring and it is resonance-assisted, whereas the H26⋅⋅⋅O14 IHB closes an 8-member ring and is not resonance-assisted.

Among the IHBs in the glucoside moiety in U8, O25–H26···O27 is slightly shorter than O25–H26···O10, O27–H28···O29 is shorter than O27–H28···O25, O29–H30···O31 is shorter than O29–H30···O27, and O31–H32···O29 is shorter than O31–H32···O24; overall, O29–H30···O31 is the shortest and O25–H26···O10 is the longest due to the position of O10.

Two bifurcated IHBs (H23⋅⋅⋅O32 and H26⋅⋅⋅O32) are present in the ring systems in U4 and U5. Both of them are shorter in U5 than in U4. This is likely due to the presence of O33 (an sp^2^ O) in U4.

The energy increase on an H-bond removal (by 180° rotation of the donor) does not correspond to the H-bond strength, because other modifications of the molecular geometry usually accompany the removal (like the off-plane shift of O14 in the acylphloroglucinol moiety). On the other hand, when the removal of an IHB does not lead to the formation of a different IHB, the comparison of the energy increases provides indications about the relative stabilising effects of the IHBs concerned (e.g., the previously-mentioned comparison between U2-d-x-a and U2-x-a). Table S5 reports the energy increase accompanying the removal of specific IHBs, comparing suitably selected conformers with and without a given IHB.

The infrared (IR) vibrational frequencies (harmonic approximation) were calculated for all the compounds. The frequencies of the O–H groups are relevant for IHBs comparisons because the frequency of a polar group decreases when it acts as H-bond donor (while its bond length increases); the decrease is called *redshift*; its value provides indications about the strength of an H-bond, because it is greater for stronger H-bonds.

The calculated frequencies of all the OHs present in each molecule are reported in Table S6 for the DFT results and Table S7 for the HF results. The vibrations of some OHs are coupled, with symmetric and asymmetric vibrations (clearly shown by the vibrations’ visualization). In U4 and U5, the vibrations of O22–H23 and O25–H26 are coupled, with identical frequency values, when they form simultaneous (and cooperative) IHBs with O32.

The redshift of an H-bond donor is evaluated as the difference between its frequency when it is free (not engaged in an H-bond) and its frequency when it forms an H-bond. In the present work, the reference frequency for each OH has been evaluated as the average of its frequencies in the conformers of the given molecule, in which it is free. Table S8 reports the redshifts for the IHBs in the calculated conformers of the considered ACPLs in the DFT results, and Table S9 reports those in the HF results. Table 7 reports their ranges. The computed redshifts are consistent with the IHB-strengths comparison that can be derived from the IHB lengths. The redshift is greatest for the IHBs in which O8–H15 is the donor; among these, it is somewhat smaller in the conformers of U7 and U8 than in the conformers of the other molecules, likely because R is cyclic in U7 and U8.

**Table 7:** Range of the red-shifts in the calculated vibrational frequencies (harmonic approximation) of the O–H bonds that act as IHB donors in the calculated conformers of the considered ACPL molecules

Molecules	Considered IHB	Range of the red-shifts (cm^−1^)	
		DFT	HF
U1	H15···O14	674.92–755.20	240.38–306.78
U2	H15···O14	799.89–806.20	236.34–295.69
	H17···O14	701.82–802.49	340.49–342.33
U3	H17···O14	721.40–804.83	286.62–326.76
U4	H15⋅⋅⋅O14	47.69–786.17	316.36–336.80
	H23···O32	482.45–603.90	179.91–191.87
	H25···O32	415.40–541.73	164.91–184.79
U5	H15⋅⋅⋅O14	730.52–779.67	305.61–325.88
	H23···O32	449.35–635.64	208.61–243.60
	H25···O32	438.51–589.70	178.15–221.17
U6	H15···O14	629.13–789.35	250.00–341.51
U7	H15···O14	575.69–724.94	203.26–272.35
	H26···O14	86.41–147.25	37.49–82.90
	H23···O32	79.46–86.26	42.77–45.67
U8	H15···O14	515.89–598.79	146.45–177.59

DFT/B3LYP/6–31 + G(d,p) and HF/6-31G(d,p) results from full optimisation, respectively denoted as DFT and HF in the columns’ headings. The molecules are denoted with the symbols listed in Table 1

The redshifts when O22–H23 and O25–H29 form H23⋅⋅⋅O32 and H26⋅⋅⋅O32 simultaneously are considerably smaller than when either IHB is present alone; this is consistent with the greater length of these IHBs when they are present simultaneously, in relation with them being bifurcated on O32. A similar phenomenon is noted for the redshifts when O22–H23 and O25–H26 form H23⋅⋅⋅O32 and H26⋅⋅⋅O32 simultaneously than when either IHB is present alone.

#### HOMO–LUMO energy gap of the conformers

The frontier molecular orbitals, i.e., the highest occupied molecular orbital (HOMO) and lowest unoccupied molecular orbital (LUMO) relate to important molecular properties: the HOMO to the electron-donating ability of a molecule and the LUMO to its electron-accepting ability. The HOMO–LUMO energy gap relates to a molecule’s reactivity and various other properties, including spectroscopic and electric conductivity ones. A molecule’s reactivity influences also its biological activities. The HOMO and LUMO individual energies and their energy gap are among the descriptors utilised in the study of a molecule’s activity, including quantitative structure–activity relationships, QSAR [95, 96], and the study of anticancer activities [97–100].

Table S10 reports the values of the HOMO–LUMO energy gap for the conformers of the considered molecules, in the results of the three calculation methods utilised. Figure S3 compares these values in terms of the computational methods. The results show the known phenomenon for which the DFT values are substantially smaller than the values obtained from the two ab initio methods. However, the trends across conformers of the same molecule are similar in the results of the three methods. Table 8 shows the ranges of the HOMO–LUMO energy gap, thus also showing how it depends on the nature of the molecule: the lowest end of the ranges indicate that the smallest energy gap pertains to U4, while the greatest pertains to U1.

**Table 8:** Ranges of the HOMO–LUMO energy gap of the calculated conformers of the considered ACPL molecules

Molecules	HOMO–LUMO energy gap range (kcal mol^−1^)		
	DFT	HF	MP2
U1	103.30–126.82	260.06–277.56	253.92–287.81
U2	95.79–123.32	245.39–271.57	237.10–263.66
U3	99.12–121.23	249.94–272.86	243.27–267.02
U4	63.96–79.84	213.01–228.71	198.24–215.05
U5	84.22–96.30	232.99–247.21	225.21–236.25
U6	96.99–122.23	251.45–288.63	244.26–281.30
U7	92.04–102.01	245.77–261.28	235.02–258.62
U8	92.53–111.09	239.68–265.48	237.42–258.52

DFT/B3LYP/6–31 + G(d,p), HF/6-31G(d,p) and MP2/6-31G(d,p) results in vacuo from full optimization calculations, respectively denoted as DFT, HF and MP2 in the columns’ headings

Besides the nature of the given molecule, the energy gap is influenced by relevant conformational features such as IHBs and other intramolecular interactions, or the orientation of the OH groups. For OH groups that can form IHBs, the effects of the presence of the IHB and of the orientation of the donor OH are integrated. The influence by the position of the first IHB depends on the type of Rʹ present and on the molecule altogether: for instance, the d-conformers (O14⋅⋅⋅H15 IHB) have greater HOMO–LUMO energy gap than the s-conformers (O14⋅⋅⋅H17 IHB) in the case of U2, while the opposite occurs for the conformers of U5. The presence of the first IHB also entails that O14 and C13 are coplanar to ring A; when the first IHB is not present, O14 and C13 are off-plane, and the HOMO–LUMO energy gap is smaller. In the case of U4 and U5, the gap is smaller when the two OH groups attached to R‵‵ are both oriented towards O32, forming two simultaneous IHBs bifurcated on O32; it increases when one of them is oriented away from O32, removing one IHB; and it further increases when both of them are oriented away from O32, removing both IHBs.

The orientation of the OHs that do not, or cannot, form IHBs also has noticeable influence. For instance, the energy gap is greater when the *ortho* phenol OH not engaged in the first IHB is oriented away from the acyl group than when it is oriented towards it (u-conformers). Considering the orientation of O10 − H16, the gap is greater in the w-conformer than in the r-conformer.

#### Dipole moments of the conformers

Table S11 reports the values of the dipole moment of the calculated conformers of the considered ACPLs. Figure S4 visually compares the results from the three utilized calculation methods and highlights similar patterns. Figure S5 compares the values in terms of the types of conformers.

The dipole moment is primarily influenced by the mutual orientation of the OH groups [21]. The influence of the phenol OHs of the phloroglucinol moiety is easier to interpret, because they are coplanar: their total contribution is zero when they have uniform orientation (Fig. 5) and significant when they do not have uniform orientation. Thus, in molecules with uniform orientations of the three phenol OHs, the contributions to the dipole moment are due to the rest of the molecule.

When a molecule has additional OH groups, their individual contributions are not always easy to analyse (e.g., [49]), above all when the OHs pertaining to different moieties are on different planes. Some features may be identified. For instance, in the conformers of U2, the dipole moment is greater when O22–H23 (in the E ring) and O8–H15 are oriented in the same way, and smaller when they are oriented in opposite ways; the conformer with the smallest dipole moment is U2-d-x-a, where the orientations of O22–H23 and O12–H17 are opposite to the orientation of O8–H15. Similarly, in the conformers of U3, the dipole moment is greater when O22–H23 and O10–H16 are oriented in the same way, and smaller when they are oriented in opposite ways, with the smallest value pertaining to U4-d-ε-r-x-k. Conformers of U4 show smaller dipole moments than the corresponding conformers of U5, likely because of the opposing contribution from the C = O33 bond (present in U4 and not in U5) with respect to the total contribution of the two OHs on the other side of the ring system (which is the same in U4 and U5). The importance of the contributions of the orientation of additional OH groups is also highlighted by the case of U8, where different orientations of the four OH groups in the glucoside moiety may lead to dipole moments as high as 6.050, 8.507, and 6.398 debye, or as low as 1.191, 1.454, and 1.610 debye.

### Results in solution

Calculations in solution were performed at the HF and DFT levels in chloroform, acetonitrile and water, i.e., the same solvents utilised in previous studies on other ACPLs. Their dielectric constants (ε) are 4.1, 36.6, and 80 respectively [101] and the dielectric constant of *vacuum* is 1. Chloroform is a nonpolar solvent, suitable to model non-polar environments within living organisms; acetonitrile is an aprotic solvent with considerable polarity; and water is a highly polar solvent and is also the dominant medium within living organisms. The changes that these solvents cause in the molecular properties of the solutes are analysed in the next subsections.

#### Relative energies of the conformers

Table S12 compares the relative energies of the calculated conformers in the DFT results for the four media considered (*vacuum*, chloroform, acetonitrile, and water), and Table S13 compares them in the HF results. Figure S6 provides graphical representations of these comparisons for each of the considered ACPL molecules.

In the DFT results, the lowest energy conformer is the same in all the media except for U3 in acetonitrile, where there is a reversal with the second-lowest energy conformers. The relative energy of a conformer decreases as the solvent polarity increases, with few exceptions for U1, U6 and U7. The decrease is sharper for conformers without any IHB; this can be related to the fact OH groups that are not engaged in IHBs become available for stronger solute–solvent interactions such as solute–solvent H-bonds; PCM models – although not capable of taking into explicit account strong solute–solvent interactions such as H-bonds – take the presence of stronger interactions into partial account in an implicit way, through the polarization charges [102].

The free energy of solvation (ΔG_solv_), or solvent effect, is the change in Gibbs free energy when a solute is dissolved in a solvent; it indicates the stabilization of the solute molecules resulting from solute–solvent interactions in solution. The more negative the value of ΔG_solv_, the greater the tendency of the given molecule to dissolve in the given solvent. Table S14 reports the solvation free energy (ΔG_solv_) and its electrostatic (G_el_, [103]) and dispersion [104–108] components for the calculated conformers of the considered ACPLs in chloroform, acetonitrile and water in the DFT results, and Table S15 reports them in the HF results. Figure S7 provides graphical comparisons.

ΔG_solv_ has the highest values in acetonitrile, where it is always positive for the conformers of compounds U1-U7, with the exception of U4-d-w-v-k, U4-w-v-k and U5-d-v-k-w, and negative for all the conformers of U8. The values in chloroform are considerably smaller than in acetonitrile; they are always positive for the conformers of U1, U2 (except U2-x-a), U3, U6 and U7 (with only one non-significant exception); they are mostly negative for U4 and U5, and they are all negative, and with considerably greater magnitude, for U8. The values in water have small magnitude (some positive and some negative) for U1, are negative and with greater magnitude for U2-U7, and negative and with the greatest magnitudes for U8. The ΔG_solv_ of the conformers without the first IHB is smaller than that of the other conformers for all the molecules and in all the three solvents. The compounds with additional OHs (U4, U5, U8) have more negative values than the others, with U8 having the most negative ones (negative and with considerably greater magnitudes). Table 9 shows the ranges of ΔG_solv_ for the conformers of each molecule in the three solvents considered, excluding the conformers without any IHB because of the significant difference of their values with respect to the others.

**Table 9:** Ranges of the solvation free energy (ΔG_solv,_ kcal mol^−1^) for the calculated conformers of the molecules considered in this work

Molecule	DFT			HF		
	chlrf	actn	aq	chlrf	actn	aq
U1	6.39–7.10	13.62–14.78	(−0.26)–0.95	(−4.00)–(−2.47)	2.57–4.64	(−12.47_–(−7.67)
U2	1.08–2.49	6.53–9.04	(−7.51)–(−6.05)	(−4.82)–1.83	0.56–8.39	(−17.67_–(−8.80)
U3	0.61–3.58	6.11–9.68	(−11.35)–(−5.15)	(−3.80)–1.96	(1.68)–8.19	(−16.40)–(−8.92)
U4	(−8.28)–(−1.06)	(−5.69)–3.80	(−28.25)–(−12.36)	(−0.82)–1.68	3.79–7.39	(−12.08)–(−5.50)
U5	(−2.87)–0.41	(1.17)–5.09	(−16.19)–(−9.39)	(−1.34)–1.32	3.30–7.10	(−12.76)–(−6.20)
U6	2.20–3.62	6.35–8.36	(−4.08)–(−1.73)	(−0.72)–2.10	3.36–6.56	(−6.70)–(−3.51)
U7	(−0.03)–2.38	4.59–7.46	(−14.67)–(−10.86)	(−1.96)–3.19	(−2.04)–8.99	(−17.54)–(−11.76)
U8	(−9.72)–(−8.04)	(−7.30)–(−4.12)	(−31.96)–(−27.64)	(−8.49)–(−6.10)	(−6.46)–(−2.63)	(−33.68)–(−28.60)

DFT/B3LYP/6–31 + G(d,p) and HF/6-31G(d,p) results from full optimization calculations, respectively denoted as DFT and HF in the columns’ headings. The values of conformers without any IHB are excluded from the identification of the ranges because of their significant difference with respect to the other values

The smaller or more negative values of ΔG_solv_ correspond to the molecules with greater numbers of OHs, i.e., to the molecules with more sites capable of stronger solute–solvent interactions; U8, with seven OH groups, has the ΔG_solv_ most favourable to the dissolution process. Of the molecules with more positive values, U6 has only two OH groups and U1 has a long alkyl chain which is not favourable to interactions with polar solvents. For analogous reasons, conformers with no IHBs, or smaller numbers of IHBs, have ΔG_solv_ values more favourable to the dissolution process, because the OHs not engaged in IHBs are more available for solute–solvent interactions.

The electrostatic (G_el_) and dispersion components of ΔG_solv_ are negative in all three solvents, with their magnitude being greater in water than in the other two solvents. For each molecule, G_el_ has greater magnitude for conformers without any first IHBs; among the considered molecules, it has considerably greater magnitude for the calculated conformers of U8 – the compound with the highest number of OH groups. The dispersion component has greater magnitude in compounds with long R chains, with the greatest magnitudes pertaining to U1.

The DFT and HF results show various significant differences. It has to be taken into account that all the solute–solvent interactions possible for these molecules are non-covalent and, therefore, they depend significantly on dispersion components, which are taken into account to a greater extent by DFT.

#### Characteristics of the intramolecular hydrogen bonds

A solvent may cause some modifications in the IHBs by interacting with the donor and/or the acceptor. Table S16 shows the parameters of the various IHBs present in the calculated conformers, in the three selected solvents, considering the DFT results, and Table S17 reports the parameters in the HF results. Table S18 compares the lengths of the IHBs in the four media considered (*vacuum* and the three solvents) and Figure S8 illustrates this comparison. Table 10 shows the ranges of the IHB lengths in the four media.

**Table 10:** Ranges of the lengths of the intramolecular hydrogen bonds in the calculated conformers of the considered ACPL molecules in chloroform, acetonitrile and water (respectively denoted as chlrf, actn, and aq in the column headings)

Molecules	Considered IHB	Method	Range of the length of the IHB (Å)			
			vac	chlrf	actn	aq
U1	H15···O14	DFT	1.563–1.585	1.552–1.719	1.548–1.719	1.547–1.719
		HF	1.692–1.719	1.679–1.708	1.677–1.703	1.677–1.702
U2	H15···O14	DFT	1.545–1.546	1.538–1.539	1.536–1.536	1.535–1.536
		HF	1.672–1.673	1.669–1.669	1.667–1.667	1.667–1.667
	H17···O14	DFT	1.570–1.588	1.554–1.567	1.548–1.559	1.559–1.696
		HF	1.696–1.733	1.686–1.718	1.682–1.712	1.682–1.711
U3	H17···O14	DFT	1.545–1.565	1.536–1.550	1.533–1.545	1.532–1.544
		HF	1.681–1.697	1.677–1.686	1.678–1.683	1.678–1.682
U4	H15···O14	DFT	1.539–1.547	1.533–1.537	1.530–1.533	1.529–1.533
		HF	1.665–1.671	1.660–1.664	1.658–1.661	1.665–1.671
	H23···O32	DFT	1.615–1.654	1.602–1.644	1.596–1.640	1.595–1.640
		HF	1.766–1.785	1.767–1.775	1.767–1.772	1.766–1.785
	H25···O32	DFT	1.684–1.694	1.679–1.686	1.677–1.683	1.677–1.682
		HF	1.800–1.814	1.803–1.812	1.803–1.811	1.800–1.814
U5	H15···O14	DFT	1.536–1.541	1.529–1.535	1.527–1.530	1.527–1.530
		HF	1.666–1.670	1.660–1.662	1.657–1.658	1.658–1.671
	H23···O32	DFT	1.609–1.660	1.591–1.651	1.583–1.647	1.582–1.647
		HF	1.747–1.760	1.743–1.754	1.740–1.752	1.747–1.758
	H25···O32	DFT	1.627–1.682	1.606–1.669	1.598–1.663	1.597–1.663
		HF	1.767–1.783	1.758–1.774	1.753–1.770	1.762–1.776
U6	H15···O14	DFT	1.538–1.585	1.525–1.573	1.520–1.557	1.519–1.574
		HF	1.663–1.722	1.663–1.713	1.661–1.708	1.661–1.707
	H17···O14	DFT	1.563	1.548	1.543	1.542
		HF	1.691	1.681	1.678	1.678
U7	H15···O14	DFT	1.565–1.601	1.536–1.580	1.550–1.575	1.538–1.574
		HF	1.703–1.746	1.689–1.740	1.684–1.736	1.683–1.735
	H26···O14	DFT	1.923–1.979	1.863–1.885	1.850–1.860	1.849–1.858
		HF	2.057–2.128	2.014–2.060	1.990–2.036	1.988–2.034
U8	H15···O14	DFT	1.627–1.656	1.619–1.639	1.615–1.633	1.615–1.632
		HF	1.808–1.856	1.809–1.837	1.809–1.861	1.809–1.861
	H32···O10	DFT	2.535–2.632	2.562–2.681	2.559–2.691	2.558–2.692
		HF	2.535–2.632	2.540–2.633	2.538–2.641	2.537–2.642
	H32···O28	DFT	2.416–2.430	2.434–2.444	2.435–2.445	2.435–2.445
		HF	2.441–2.452	2.445–2.455	2.447–2.452	2.447–2.452
	H33···O27	DFT	2.353–2.402	2.383–2.422	2.396–2.441	2.397–2.428
		HF	2.371–2.408	2.395–2.428	2.404–2.448	2.405–2.450
	H33···O29	DFT	2.183–2.402	2.192–2.239	2.192–2.248	2.192–2.249
		HF	2.209–2.408	2.215–2.241	2.215–2.249	2.215–2.249
	H34···O28	DFT	2.202–2.219	2.239–2.249	2.255–2.261	2.256–2.262
		HF	2.227–2.233	2.258–2.261	2.269–2.272	2.270–2.273
	H34···O31	DFT	1.898–2.219	1.871–1.926	1.866–1.907	1.865–1.906
		HF	1.947–2.233	1.941–2.015	1.943–2.009	1.944–2.009
	H35···O29	DFT	1.992–2.617	2.018–2.501	2.013–2.522	2.013–2.524
		HF	2.036–2.429	1.941–2.261	1.943–2.272	1.944–2.273
	H17···π (C13)	DFT	2.172–2.193	2.170–2.315	2.168–2.322	2.300–2.323
		HF	2.301–2.638	2.300–2.323	2.295–2.315	2.300–2.323

DFT/B3LYP/6–31 + G(d,p) and HF/6-31G(d,p) results from full optimisation PCM calculations, respectively denoted as DFT and HF in the third column. The molecules are denoted with the symbols listed in Table 1

The H-bond length of many of the IHBs present in these molecules decreases as the medium polarity increases. It is the case of H17···O14, H15···O14, H23···O32, H26···O32, H26···O14, and H30···O31. With the exception of O31, these are all IHBs in which the acceptor is an sp^2^ O, which makes them significantly stronger and, therefore, less prone to be weakened by a solvent; in addition, the region in their vicinity is hydrophobic [25] and, therefore, water molecules do not tend to disrupt them. On the other hand, the length of H26···O10, H26···O27, H28···O25, H28···O29, H30···O27, and H32···O29 increases as the medium polarity increases. It can also be noted that the IHB bond lengths tend to be closer for corresponding conformers in acetonitrile and in water (the two polar solvents) than in chloroform or *in vacuo*. The HF results show some exceptions to these trends.

Tables S16, S17, and S18 also consider the O–H⋅⋅⋅π interactions with the B aromatic ring, using the H17⋅⋅⋅C13 distance as an indication of how the OH approaches the π cloud. This distance increases with the medium’s polarity.

#### HOMO–LUMO energy gap

The presence of a solvent may influence the energies of HOMO and LUMO differently, and this reflects on the value of the HOMO–LUMO energy gap [109, 110]. The influence on these energies depends on the nature of the molecular system and of the solvent.

Table S19 reports the values of the HOMO–LUMO energy gap of the calculated conformers of the selected ACPLs, in the four media considered, from the DFT results, and Table S20 reports the values from the HF results. Figure S9 offers graphical representations comparing the values in the four media, with separate diagrams for DFT and HF results, because of the different scales of the values.

The trends in the four media are fairly similar for the conformers of the same molecule. Interestingly, the minimum and maximum values of the gap pertain to the same conformers in all the four media. Specific trends can be identified for some molecules. For instance, the values of the HOMO–LUMO energy gap *in vacuo* are significantly smaller than those in solution for most of the calculated conformers of U7 and U8, and they moderately increase as the medium’s polarity increases. The values *in vacuo* are greater than those in solution for all the calculated conformers of U3, and they decrease as the medium’s polarity increases. The trends are less regular for the conformers of the other molecules.

#### Dipole moment of the conformers

Solvents with different polarities affect the dipole moment of the solute molecules differently. Table S21 reports the values of the dipole moment of the calculated conformers of the selected ACPLs in the four media considered from the DFT results and Table S22 reports the values from the HF results. Figure S10 offers a graphical representation of the corresponding trends.

The results are consistent with commonly observed behaviours, i.e., the dipole moment of a conformer tends to increase as the medium polarity increases (some abnormalities appear for the HF results of compound U8, with some values *in vacuo* being greater than in solution). The greatest increases appear for molecule U5.

### Molecular docking results

#### Background information

The docking protocols and parameters were firstly validated by using the re-docking method [111]. A co-crystal structure of the protein of interest with a known inhibitor (ligand) is taken from the Protein Data Bank (PDB); the ligand is separated and then docked again into the same protein, using the selected docking method. The validity of the selected docking method is confirmed if the ligand fits back into the protein in the same way as in the original co-crystal structure. This occurred for all the re-docking operations in the current study, confirming the reliability of the selected docking protocols and of the information obtained with these targets.

Eleven molecular targets (proteins) were selected, eight of which are suitable to test anticancer activity and three for antimalarial activities, related to *Plasmodium Falciparum*. Their names and PDB IDs, as well as short descriptions, are presented in Table 11. Their structures are shown in Figure S11, and their binding sites are highlighted by co-crystalized ligands. Dockings were performed between each of the eight considered ACPLs and the active sites of each of the eleven proteins. The binding energies and the intermolecular interactions for each of the resulting ligand–protein docked pairs are reported in Table S23, together with those of the co-crystal ligands which serve as comparison references.

**Table 11:** ADME related properties for the eight ACPL compounds considered in this study

Molecule considered	Percent PHOA	QPPCaco	QPPMDCK	QPlogBB	QPlogKhsa
	< 25% poor, > 80% high	< 25-poor, > 500-great	< 25-poor, > 500-great	−3 to 1.2	−1.5 to 1.5
U1	100.0	370.3	169.0	−2.4	1.4
U2	94.8	514.9	241.4	−1.6	1.1
U3	100.0	321.7	145.2	−1.9	0.8
U4	72.8	56.0	21.9	−2.1	0.2
U5	81.6	85.2	34.5	−1.9	0.6
U6	100.0	1470.5	750.5	−0.8	0.7
U7	92.0	129.4	54.3	−2.3	0.9
U8	79.2	196.9	85.4	−1.4	−0.1

The following properties are considered: percent predicted human oral absorption (%PHOA); predicted apparent Caco-2 cell permeability (QPPCaco, nm/sec); predicted apparent MDCK cell permeability (QPPMDCK, nm/sec); predicted brain/blood partition coefficient (QPlogBB); and predicted binding capacity to human serum albumin (QPlogKhsa). The values were calculated using QikProp [73]. The row under the names of the properties shows the recommended values-ranges

The ligand–protein interactions for molecules like the ones considered in this work (and as they are taken into account by the Maestro software) comprise H-bonds, aromatic H-bonds, hydrophobic interactions, π⋅⋅⋅π stacking interactions, polar interactions, glycine interactions and cation⋅⋅⋅π interactions. H-bonds are well known and do not require additional explanations; they are also the strongest non-covalent interactions. They are the prevailing directional intermolecular interactions in biological complexes and play dominant roles in the specificity of molecular recognition [112]. Aromatic H-bonds are O–H⋅⋅⋅π H-bonds, in which an aromatic ring has the role of acceptor.

In their primary meaning, hydrophobic interactions refer to the tendency of non-polar molecules, or non-polar parts of molecules, to aggregate together in water solution [113]. In the ligand–protein complex, they involve the non-polar part of the ligand and the binding site of the protein and they often play important roles in the overall interaction [112]. They can also be modulated, which may be particularly expedient in certain forms of drug design [114].

π⋅⋅⋅π stacking interactions are interactions between the π clouds or aromatic planes, mostly with edge-to-face or parallel displaced face-to-face geometries. In a ligand–protein complex, they involve the aromatic ring of an aminoacid in the protein (most frequently, phenylalanine, followed by tyrosine, tryptophan and histidine) and an aromatic ring in the ligand [112]. They play vital roles in biological recognition and in the organization of biomolecular structures [115].

Polar interactions refer to non-bonded polar contacts between non-hydrogen donor and acceptor atoms in the ligand–protein complex [116]. An atom–atom distance range for which the interaction is recognized as present can be selected; for instance, [116] selects a range of 2.5–3.5 Å. [112] finds that polar interactions are often over-represented in bound fragments, when the solvent is not included in the model.

Glycine interactions refers to the fact that glycine is a frequent acceptor of C-H–-O H-bonds and a frequent donor of amide⋯π interactions [112].

Cation⋅⋅⋅π interactions are interactions between a positively charged N atom and the electron rich cloud of an aromatic ring [112]. In the case of the considered ACPLs (which do not contain N atoms), as well as in the majority of the other cases, the N atom pertains to a cationic amino acid residue in the receptor and the aromatic ring to the ligand. Cation⋅⋅⋅π interactions make important contribution to small-molecule recognition at a protein’s binding site [117].

#### Molecular docking results against the selected anticancer-targets

This section presents the results of the molecular docking of the eight considered ACPLs against each of the targets selected for the anticancer activity, organising the presentation in terms of the proteins. As already mentioned, Table S23 reports the interaction energy of each case and enables its comparison with that of the co-crystal ligand. Figure S12 provides individual illustrations of the types of interactions and the residues involved, for the cases in which the interaction energy of the given ACPL is better than, or close to, that of the co-crystal ligand. These are also the cases that are emphasised in the analysis in this section. Table S24 lists the residues involved in the interactions with the ligand, offering two different organisations (table a and table b) to facilitate different comparisons outlooks; both tables are organised in terms of the ACPL molecules; in table a, the targets are listed in the same sequence for each molecule whereas, in table b, they are listed in order of decreasing magnitude of the interaction energy.

##### Docking with EGFR

The results show that all the considered molecules can interact with its active site (Table S24). U4 emerges as the top-docked compound, with a binding energy of −8.013 kcal.mol^−1^. It forms two H-bonds with residue SER797 and shows hydrophobic interactions with atoms of other residues within the active site (Figure S12.a).

##### Docking with JAK3

The results show that all the considered molecules can interact with its active site. U8 emerges as the best-docked compound, with a binding energy of −9.814 kcal.mol^−1^, which is comparable to that of the co-crystal ligand. It engages in three H-bonds, with ASP912, DYS909, and TYR904, along with two aromatic H-bonds, with ASP967 and TYR904. It also shows hydrophobic interactions with ALA853, LEU828, VAL836, and CYS909 (Figure S12.b). These findings suggest that U8 holds promising potential as a JAK3 inhibitor.

##### Docking with Topo I

The results show that all the considered molecules can interact with its active site. U5 emerges as the best-docked compound, with a binding energy of −9.928 kcal.mol^−1^, which is notably stronger than that of the co-crystal ligand. It forms three H-bonds, with ASN A:722, DT B:10, and DA D:112, along with an aromatic H-bond with ASN352, π⋅⋅⋅π stacking interactions with DT B:10, and polar interactions with ASN A:352, THR A:718, and ASN A:352 (Figure S12.c). These findings suggest U5’s potentialities as a promising Topo I inhibitor.

##### Docking with PI3K

PK13 has two active sites, denoted as B1 and B2. Most of the considered ACPL molecules bind to site B1, and a few bind to site B2. U5 emerges as the best-docked compound on-site B1, with a binding score energy of −10.250 kcal.mol^−1^, which is comparatively stronger than that of the co-crystal ligand. It forms three H-bonds, with VAL882, ASP950, and TYR867, and an aromatic H-bond with TRP 812. It also shows hydrophobic interactions with PRO810, TYP812, ILE879, ILE881, VAL882, and ALA885 (Figure S12.d). These findings suggest U5’s potentialities as a promising PI3K inhibitor targeting site B1.

U8 is the best-docked compound on-site B2 of PI3K, with a binding score energy of −7.224 kcal.mol^−1^, which is also comparatively stronger than that of the co-crystal ligand. U8 forms three H-bonds, with VAL882, LYS833, and ASP950, an aromatic H-bond with TRP 812, π⋅⋅⋅π stacking interaction with TYR867, and hydrophobic interactions with VAL882, ILE881, ILE879, and TYR867 (Figure S12.e).

##### Docking with BRAF V600B

BRAF V600B has three identified docking sites, denoted as C1, C2, and C3. The ligand–protein complexes show better binding scores on site C1 than on sites C2 and C3. U4 shows the best docking performance with C1, with a binding score energy of −11.013 kcal.mol^−1^. It forms two H-bonds, with ASN580 and CYS532, an aromatic H-bond with ASN580, a π⋅⋅⋅π stacking interaction with PHE595, and hydrophobic interactions via ILE463 (Figure S12.f).

The C2 site shows the best docking complex with U7, with a binding score energy of −10.862 kcal.mol^−1^. U7 forms two H-bond, with CYS532 and THR529, π⋅⋅⋅π stacking interactions with PHE595, and hydrophobic interactions via ILE527, TRP531, CYS532, PHE595, and ILE463 (Figure S12.g). These findings suggest that – although its binding score is weaker than that of the co-crystal ligand – the interaction is strong enough to consider that U7 may have potential as a BRAF V600B inhibitor targeting site C2.

The C3 site shows the best docking performance with U2, with a binding score energy of −6.853 kcal.mol^−1^, which is comparatively stronger than the co-crystal ligand one. U2 forms three H-bonds, with ASP576, SER579, and GLU648, and also hydrophobic interactions with other residues (Figure S12.h).

##### Docking with H5P90

H5P90 has two active sites, G1 and G2. Most of the considered ACPL molecules bind to both sites. U8 shows the best docking performance in both sites, with binding score energies of −9.458 kcal.mol^−1^ and −8.944 kcal.mol^−1^ for sites G1 and G2, respectively. At site G1, the interaction of U8 with H5P90 is stronger than the co-crystal ligand. U8 forms five H-bonds – two with ASN51 and one each with LYS58, ASP54 and GLY135 –, an aromatic H-bond with ASP93, polar interactions with ASN106 and ASN51, and hydrophobic interactions with ILE91 and LEU107 (Figure S12.i). These findings suggest that U8 has significant potential as a ligand for both active sites, particularly G1.

At site G2, the interaction of U8 with H5P90 is relatively close to one of the co-crystal ligands. It forms five H-bonds – two with ASN106 and one each with ASN51, LYS58, THR184 –, one aromatic H-bond with ASP93, polar interactions with SER113, LYS112,ALA111, ILE110, LEU107, ASN106, TYR139, PHE138, LEU48 and hydrophobic interactions with ASN51, SER52, ALA55, LYS58, MET98 (Figure S12.j).

##### Docking with HER2

Docking with HER2. HER2 has six active sites, H1, H2, H3, H4, H5 and H6. All the considered ACPL molecules successfully bind to active sites H1 and H5 with favourable binding score energies; however, only a few ACPLs interact with the other sites. The best-docked complexes for each active site are the following: HER2-U4 (H1) with binding score energy of −10.779, HER2-U8 (H2) with −6.200, HER2-U7 (H3) with −5.955, HER2-U7 (H4) with −5.412, HER2-U4 (H5) with −10.611, and HER2-U4 (H6) with −5.756. These results suggest that, while all considered ACPLs have strong binding affinity for active sites H1 and H5, their interactions with sites H2, H3, H4, and H6 are considerably weaker and likely not adequate for inhibitory activity. The interactions between U4 and site H1, U8 and site H2, U7 and site H4, U4 and site H5, U4 and site H6, are not stronger than that of the co-crystal ligand, whereas the interaction between U7 and site H3 is comparatively stronger than that of the co-crystal ligand.

At site H1, U4 forms two H-bonds, with MET801 and ARG849, and two aromatic H-bonds with GLN799 and ASN85. Additionally, there are glycine interactions with GLY727, SER728, GLY729, PHE731, GLY732, THR733, VAL734, ARG849, GLY804, and CYS805, and hydrophobic interactions with ASN850, LEU852, SER783, THR798, GLN799, LEU800, MET801, and ALA751 (Figure S12.k).

At site H2, U8 forms five H-bonds, with ARG713, GLU744, GLN799, LYS860, and GLY778, and an aromatic H-bond with GLU744. Additionally, there are glycine interactions with GLY778, polar interactions with GLN799, and hydrophobic interactions with PRO780, LYS860, and HIE858 (Figure S12.*l*).

At site H3, U7 forms two H-bonds, with MET801 and GLU812, and glycine interactions with MET801 and LEU800 residues (Figure S12.m).

At site H4, U7 forms three H-bonds, with MET801 and ASP808, and one aromatic H-bond with MET801. Additionally, there are polar interactions with GLN799, THR798, and THR862 and hydrophobic interactions with ASP808, CYS805, MET801, and LEU800 (Figure S12.n).

At site H5, U4 forms two H-bonds – two with MET801 and one with ARG849 –, and one aromatic H-bond with ASN850. It also forms hydrophobic interactions with MET801, LEU800, GLN799, THR798, LEU726, and glycine interactions with CYS805, GLY804, GLY727, SER728, GLY729, and ALA730 (Figure S12.o).

At site H6, U4 forms two H-bonds, with TYR1005 and LYS854. Additionally, there are hydrophobic interactions with TYR1005, LEU1009, and LYS854 (Figure S12.p).

##### Docking with CDK-2

All the considered ACPL molecules, except U3, are able to interact at the active site of CDK-2. Among them, U8 shows the strongest binding affinity, −8.491 kcal.mol^−1^. It forms four H-bonds, with LEU83, ASP86, GLU8, and ASN132, an aromatic H-bond with GLU8, a π-cation interaction with LYS89 and hydrophobic interactions with LEU134, LYS33, ALA31, VAL64, and GLY13 (Figure S12.q).

#### Molecular docking results against the selected antimalarial-targets

This section presents the results of the molecular docking of the eight considered ACPLs against each of the targets selected for the antimalarial activity, utilising criteria analogous to those of Sect. "Molecular docking results against the selected anticancer-targets". Table S23 reports the interaction energy of each case and includes the values of the co-crystal ligand. Figure S13 provides illustrations of the interactions for the cases in which the value of the given ACPL is better than, or close to, that of the co-crystal ligand. Table S25 lists the residues involved in the interactions with the ligand, providing two tables organised with the same criteria as in Table S24.

##### Docking with PFPMT

U6 is the only molecule capable of interacting with PFPMT; the binding score energy is −6.168 kcal.mol^−1^, which is not stronger than that of the co-crystal ligand. U6 forms two H-bonds, with ILE36 and ASP128. It also forms hydrophobic interactions with GLY63, GLY65, ILE90, ASP85, ILE36, TYR27, and LEU240, and glycine interactions with GLY243 and TRP244 (Figure S13.a).

##### Docking with PFLDH

All the considered ACPL molecules are capable of binding at the active site of PFLDH. U8 emerges as the best-docked compound, with a binding energy of −8.209 kcal.mol^−1^, which is comparatively stronger than that of the co-crystal ligand. It forms H-bonds with LYS198, ASN197, ACE108, and VAL233, and hydrophobic interactions with THR232, VAL233, ASN234, ASN235, and MET325 (Figure S13.b).

##### Docking with PFMDH

PFMDH has five active sites, M1, M2, M3, M4, and M5. Only some of the considered ACPLs are capable of binding to some of these sites, indicating considerable sites’ selectivity. Site M3 interacts with all the considered ACPLs, showing broader binding capability. The best-docked complexes for each active site are PFMDH-U2 at M1, PFMDH-U8 at both M2 and M3, and PFMDH-U9 at both M4 and M5, with binding score energies of −6.842, −8.030, −7.533, −6.080, and −6.247, respectively. Of these, only the interaction of U8 at site M2 is stronger than that of the co-crystal ligand. Brief descriptions of these interactions are given in the next paragraphs.

At site M1, U2 engages in four H-bonds, with LYS B:273, HIE B:280, PRO B:281, and LYS D:198; it also forms polar interactions with VAL B:282, GLU B:283, PHE B:284, THR B:252, and PHE B:251, and hydrophobic interactions with ALA B:272, LEU B:250, and PHE D:195 (Figure S13.c). At site M2, U8 forms three H-bonds, two with LYS B:160 and one with LEU B:159, and an aromatic H-bond with LEU B:159; it also forms hydrophobic interactions with PHE B:195, VAL B:187, LEU D:250, and PHE D:284 (Figure S13.d).

At site M3, U8 forms four H-bonds – two with ASP32, one with ASN119 and one with THR76 – and three aromatic H-bonds, with GLN80, GLY78, and VAL117; it also forms glycine interactions with GLY10, SER9, GLY8, THR76, ALA77, GLY78, VAL79, and GLN80, hydrophobic interactions with GLN11, and polar interactions with ASN94, ASN119, SER118, VAL117, MET142, and PRO23 (Figure S13.e).

At site M4, U7 forms two H-bonds, with LYS C:273 and HIE C:280, and one aromatic H-bond with PHE 195; it also forms polar interactions with ASN A:188, VAL A:190, and PHE C:284, and hydrophobic interactions with PRO C:281, VAL A:187, LEU A:159, VAL A:161, and MET A:200 (Figure S13.f).

At site M5, U8 forms one H-bond with LYS A:273, polar interactions with THR A:252 and PHE A:251, and hydrophobic interactions with VAL C:161, LEU C:159, HIE A:280, VAL A:282, and PHE A:284 (Figure S13.g).

### Pharmaceutically relevant physicochemical properties

Quantities relevant for the prediction of the suitability of a compound as potential drug were also evaluated. These include the properties considered by the Lipinski’s rule of five and the ADME-T properties.

The “Lipinski’s rule of five” is a rule-of-thumb utilising four criteria for a preliminary prediction of the drug-likeness of a certain compound. The criteria comprise a molecular mass less than 500 a.m.u. (denoted as MW from the old-type ‘molecular weight’ term), an octanol/water partition coefficient (ClogP_o/w_) less than 5, a maximum number of 5 H-bond donors (HBD), and a maximum number of 10 H-bond acceptors (HBA). The values for the compounds considered in this work are reported in Table S26. The Pfizer registration systems used to generate an alert for compounds for which two or more of these conditions were not satisfied [118]. Of the ACPLs considered here, only U1 and U2 have a ClogP_o/w_ greater than 5 (indicating greater lipophilicity), while all the other conditions are satisfied for all the compounds. Therefore, all the compounds can be considered suitable candidates on the basis of Lipinski’s rule.

The ADME (Absorption, Distribution, Metabolism, and Excretion) properties are crucial to predict what happens to a drug within the organism. The calculated ones are listed in Sect. "ADME-T properties"., and the recommended values’ ranges are shown in the second row of Table 11. The physiological and pharmacological relevance of these parameters can be briefly recalled as follows [119, 120]. The absorption of a drug is indicated by the %PHOA and the QPPCaco descriptors – the latter relating to intestinal absorption. Two descriptors are associated with the possibility of penetration of the blood–brain barrier (BBB): QPlogBB and QPPMDCK. The BBB is a crucial barrier that separates the brain from the rest of the body. Good ability to penetrate it is important for drugs targeting the central nervous system (CNS), whereas drugs targeting other organs should have low BBB penetration to minimize any potential side effects on the CNS. Once a drug enters the blood stream, it may bind to blood plasma proteins or remain free to circulate in the blood stream, i.e., remain available to exert its action; human serum albumin is the most abundant plasma protein, and has high binding capacity for drugs; its binding to a compound (QPlogKhsa) is therefore selected as an indicator of the proportion of drug molecules that do not remain available to exert the intended action.

Table 11 reports the values of these descriptors for the ACPLs considered in this work. Comparison with the recommended ranges shows that all these compounds are suitable as potential drugs.

## Discussion and conclusions

The computational study of the selected ACPLs has identified patterns in their molecular properties, which can be useful for the investigation of possible derived structures within a search for structures with improved activities. The fact that many findings (such as the importance of IHBs for conformers’ stabilization, their characteristics, or the conformational preferences) are consistent with the general findings of previous studies on ACPLs strengthens the possibility of reliable predictions for derived molecules, as the identified trends’ framework becomes progressively more detailed and gains additional verifications. The same holds for the solvents’ effects on the molecular properties – particularly important because the consideration of a non-polar solvent and water mimics the effects of the major media present in a living organism.

Both the HOMO–LUMO energy gap and the dipole moments depend on the properties of individual molecules and conformers. The relationship between HOMO–LUMO energy gap and reactivity is reflected in the fact that molecules with smaller gaps, such as U4 and U5, show stronger protein–ligand interactions in docking studies, whereas the protein–ligand interaction of molecules with larger HOMO–LUMO gaps, like U1, remains comparatively weaker. The results for the dipole moment confirm the dominant influence of the orientations of the OH groups on its value.

Molecular docking studies have shown that the considered ACPLs can interact with biological targets with frequently good binding affinities, thanks to their ability to form stable H-bonds and other types of interactions with the active sites of the target. Some of the considered ACPLs (U4 and U5) had already been recognised as having both anticancer and antimalarial activities. The docking studies showed that other ACPLs could also potentially exert both activities. For instance (Table S23 and Figure S14), compounds U1, U7, and U8, known for anticancer properties, show good interactions with malarial targets like PFMDH. Conversely, compounds U2, U3, and U6, known for antimalarial activity, show promising interactions with cancer-related targets such as BRAF V600E, Topo I, and EGFR. The ADME-T predictions for the considered ACPLs are also favourable.

Overall, the docking studies identified H-bonding and hydrophobic interactions as the most significant interactions stabilizing molecule-protein complexes with targets for anticancer and antimalarial activities (Tables S24 and S25). Complexes with anticancer targets show a broader variety of interactions than those with antimalarial targets. H-bonding and hydrophobic contacts play dominant roles in the complexes with antimalarial targets, while interactions like π-π stacking and π-cation appear frequently as additional interactions in the complexes with anticancer targets. The difference may be related to greater simplicity of the binding site in the antimalarial targets with respect to the anticancer targets utilised in this work.

In conclusion, the obtained information suggests that the considered ACPL molecules can be of interest both in their native structures and for the explorations of derivatives which might have enhanced activity. The possibility of two therapeutical activities for each molecule further increases their interest, as the cost of the development of one drug would lead to the treatment of two burden diseases.

The limitations referring to the utilised computational approaches have already been highlighted in the relevant sections. The current study focuses on a subclass of ACPLs with antimalarial or anticancer activities; other parallel studies utilising the same approaches are focusing on other subclasses. When results are obtained for all the subclasses, it will be possible to evaluate the interest of additional studies. One of them would aim at identifying the effects of the inclusion of dispersion correction for DFT/B3LYP calculations, with the goal of relating the effects to the structural characteristics of the various subclasses and comparing possible structure-effects relationships (so far, a study of these effects has been carried out only for trimeric ACPLs [27]). The other study would involve the removal of some simplifications in the docking approach, by allowing some flexibility for the protein’s active sites (as far as allowed by the docking software) and by including the explicit water molecules that might facilitate ligand–protein interactions, e.g., by acting as bridges for H-bonds. This study would enable both the identification of the effects of such refinements and their comparison across the various subclasses of antimalarial and anticancer ACPLs.

## Supplementary Information

Below is the link to the electronic supplementary material.

ESM 1 (DOCX 2.64 MB)

ESM 2 (DOCX 58.3 KB)

ESM 3 (DOCX 56.6 KB)

ESM 4 (DOCX 55.4 KB)

ESM 5 (DOCX 41.6 KB)

ESM 6 (DOCX 59.3 KB)

ESM 7 (DOCX 59.6 KB)

ESM 8 (DOCX 112 KB)

ESM 9 (DOCX 123 KB)

ESM 10 (DOCX 59.1 KB)

ESM 11 (DOCX 7.30 MB)

ESM 12 (DOCX 3.58 MB)

ESM 13 (DOCX 2.17 MB)

ESM 14 (DOCX 1.87 MB)

ESM 15 (DCOX 24.1 KB)

ESM 16 (DOCX 23.4 KB)

ESM 17 (DOCX 23.3 KB)

ESM 18 (DOCX 56.9 KB)

ESM 19 (DOCX 17.7 KB)

ESM 20 (DOCX 31.5 KB)

ESM 21 (DOCX 31.5 KB)

ESM 22 (DOCX 29.6 KB)

ESM 23 (DOCX 28.5 KB)

ESM 24 (DOCX 21.9 KB)

ESM 25 (DOCX 21.9 KB)

ESM 26 (DOCX 23.7 KB)

ESM 27 (DOCX 22.9 KB)

ESM 28 (DOCX 29.8 KB)

ESM 29 (DOCX 30.3 KB)

ESM 30 (DOCX 57.1 KB)

ESM 31 (DOCX 56.1 KB)

ESM 32 (DOCX 46.8 KB)

ESM 33 (DOCX 22.3 KB)

ESM 34 (DOCX 23.9 KB)

ESM 35 (DOCX 23.1 KB)

ESM 36 (DOCX 23.1 KB)

ESM 37 (DOCX 23.0 KB)

ESM 38 (DOCX 160 KB)

ESM 39 (DOCX 72.1 KB)

ESM 40 (DOCX 15.6 KB)

## Data Availability

No datasets were generated or analysed during the current study.
