# Supplementary material for: Computable properties of selected monomeric acylphloroglucinols with anticancer and/or antimalarial activities and first-approximation docking study
Source: J Mol Model. 2025 Mar 12;31(4):113. doi: 10.1007/s00894-025-06299-7 (PMC11903629; doi:10.1007/s00894-025-06299-7)
Supplement: Supplementary file 1 — (DOCX 2.64 MB) [file 894_2025_6299_MOESM1_ESM.docx]

**Figure S1**

**Optimized geometries of the calculated conformers of the acylphloroglucinol molecules considered in this work.** DFT/B3LYP/6-31+G(d,p) results *in vacuo*.

The molecules are denoted with the symbols listed in Table 1, and the conformers are denoted with the acronyms indicating their geometric characteristics through the symbols listed in Table 2.

| **Calculated conformers of thouvenol A** | | | |
| --- | --- | --- | --- |
| **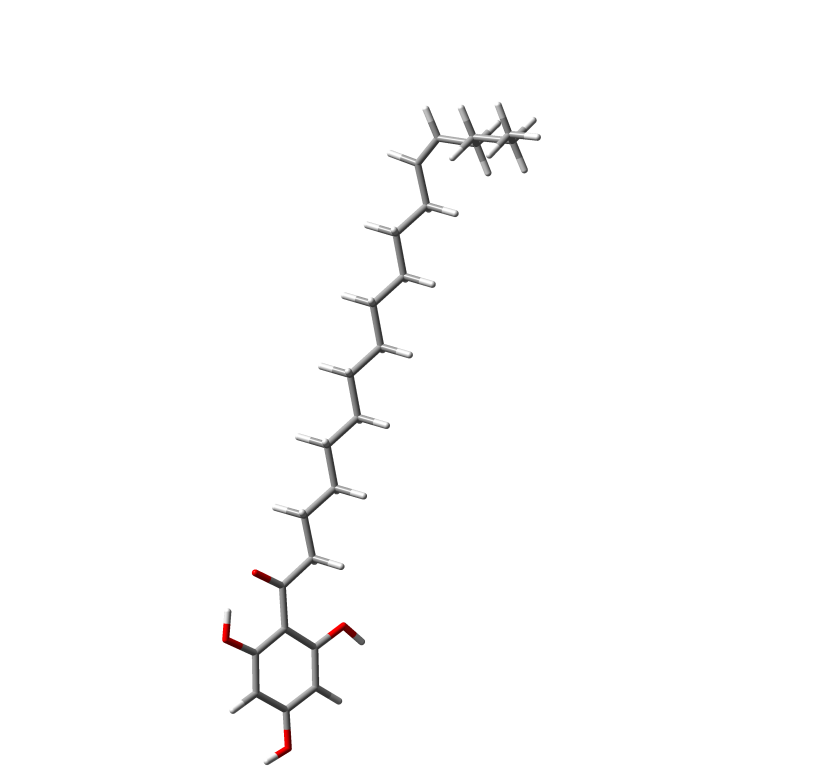** | **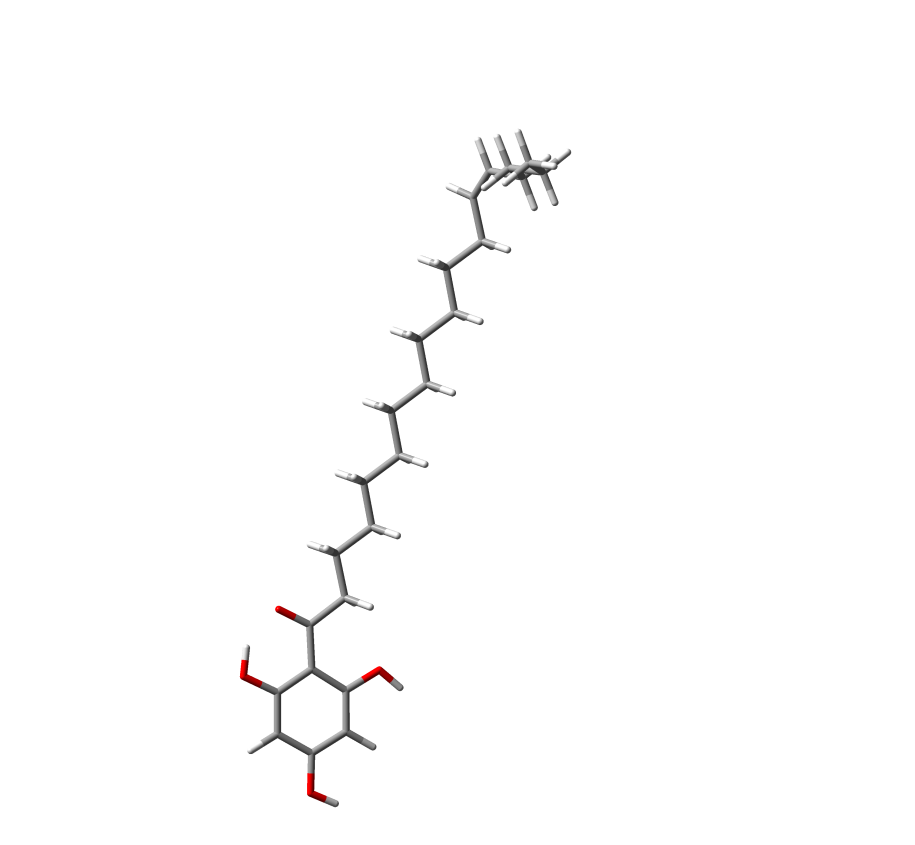** | **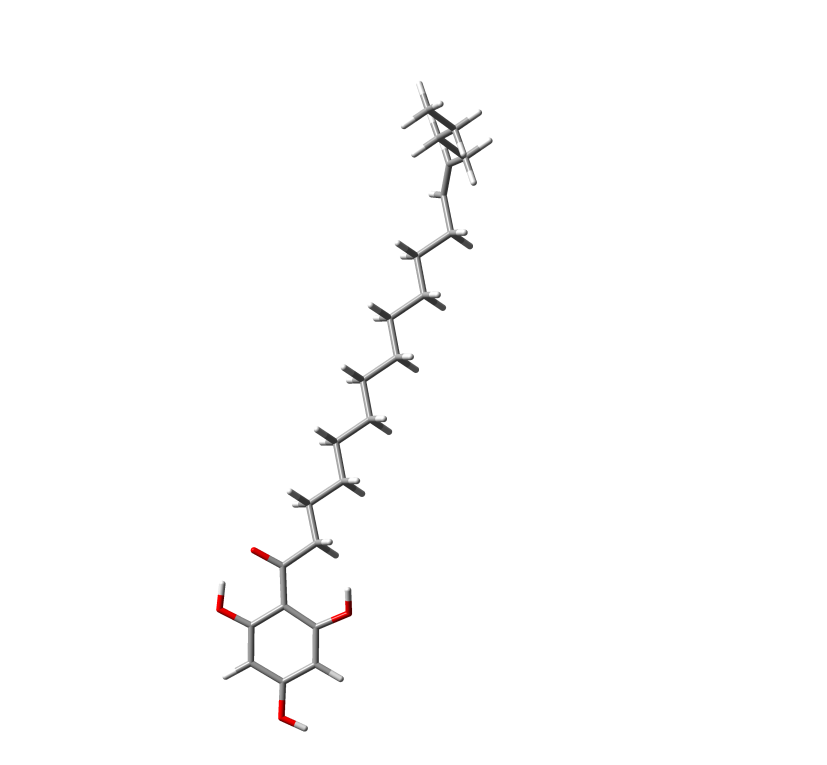** | **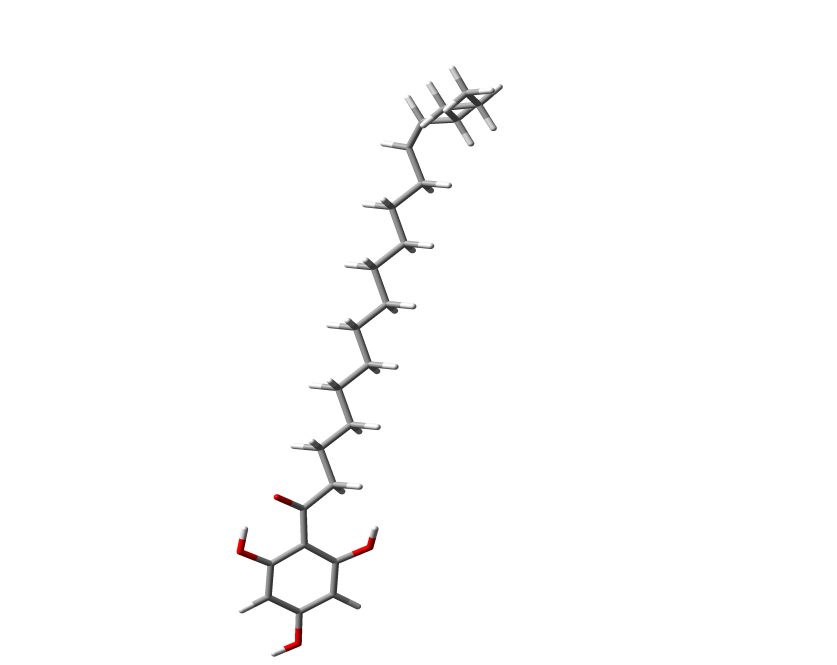** |
| U1-d-r-a | U1-d-w-a | U1-d-u-r-a | U1-d-u-w-a |
| **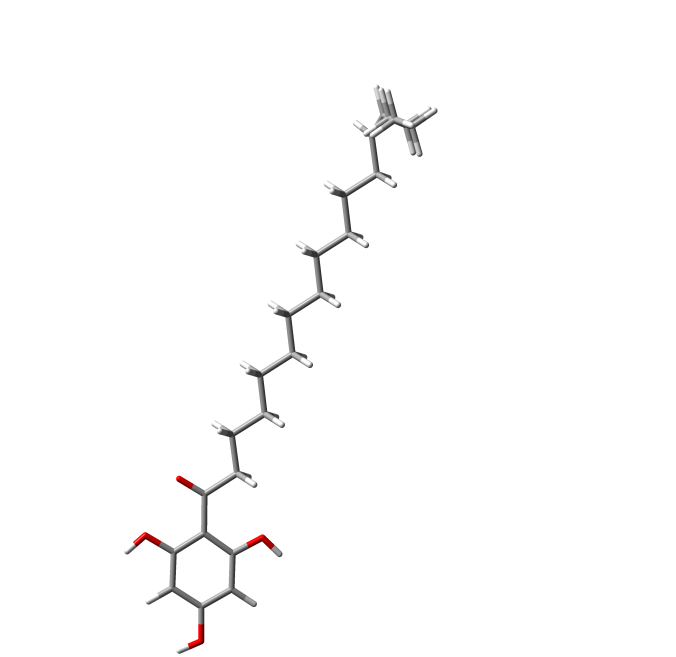** |  |  |  |
| U1-r-a |  |  |  |
| **Calculated conformers of myristicyclin A** | | | |
| **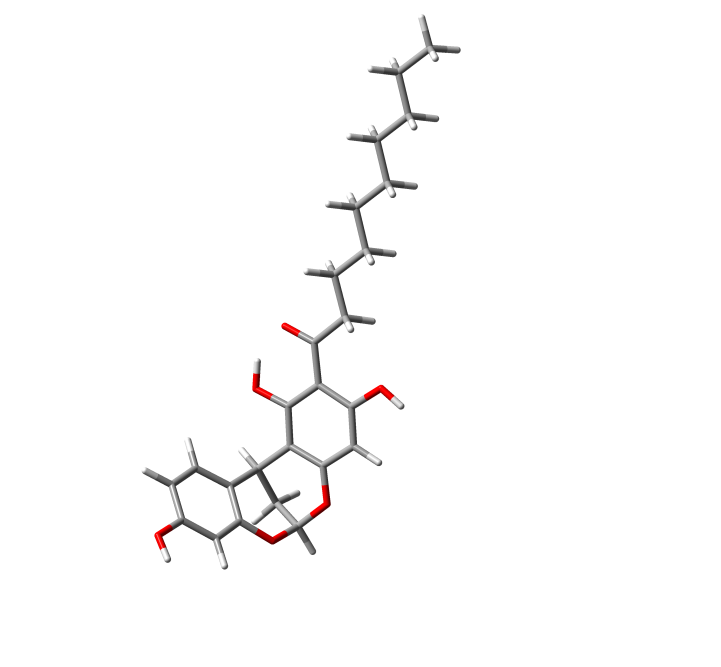** | **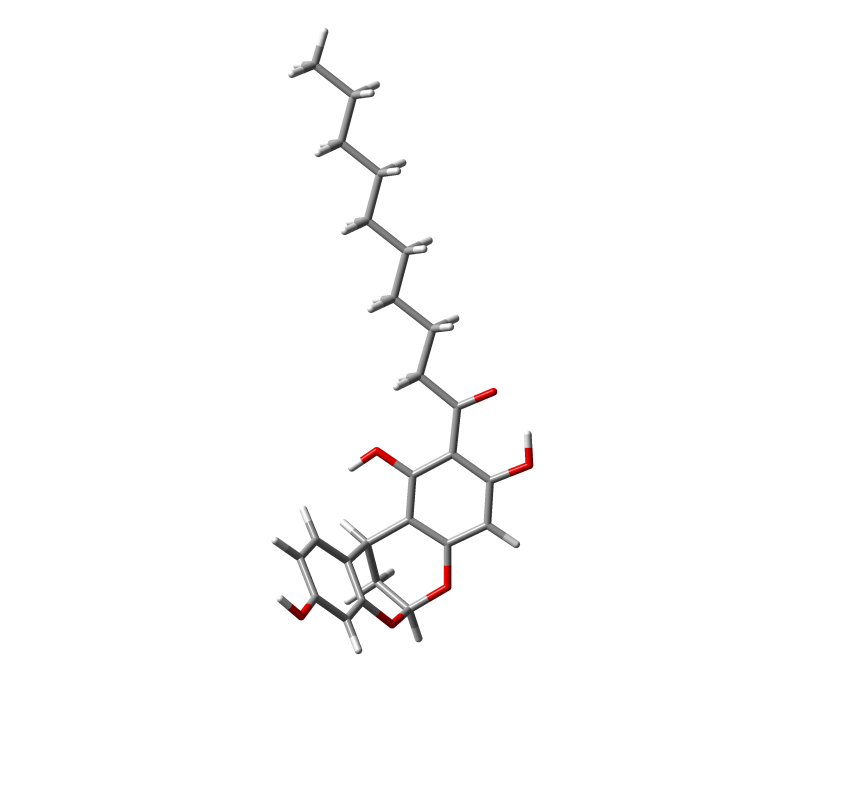** | **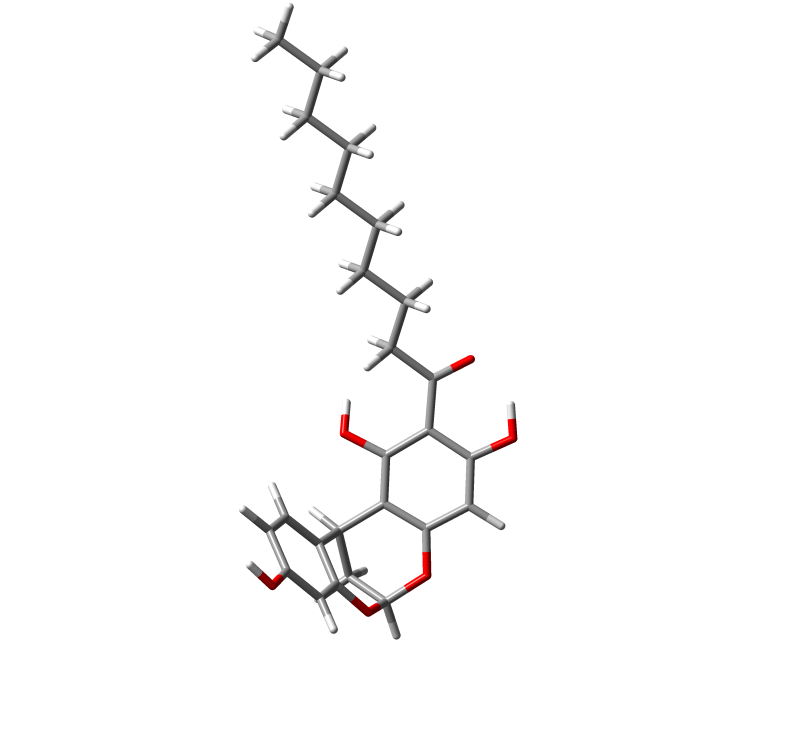** | **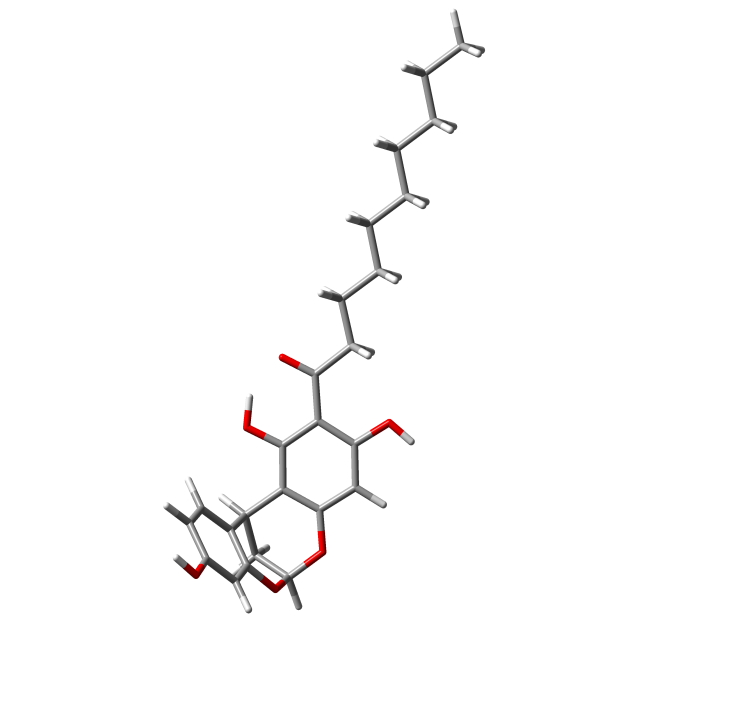** |
| U2-d-v-a | U2-s-v-a | U2-s-v-u-a | U2-d-x-a |
| 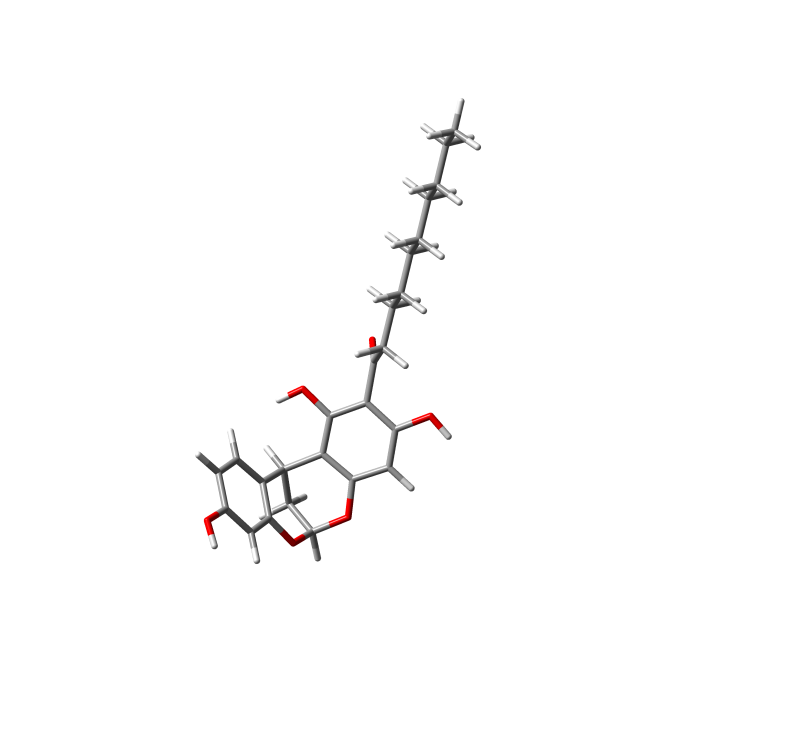 |  |  |  |
| U2-x-a |  |  |  |
| **Calculated conformers of myristicyclin B** | | | |
| **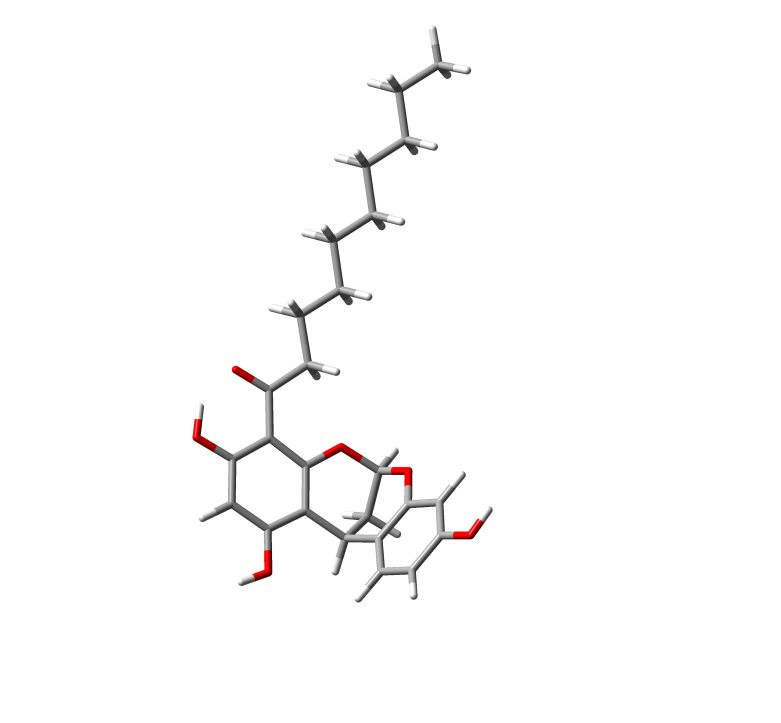** | **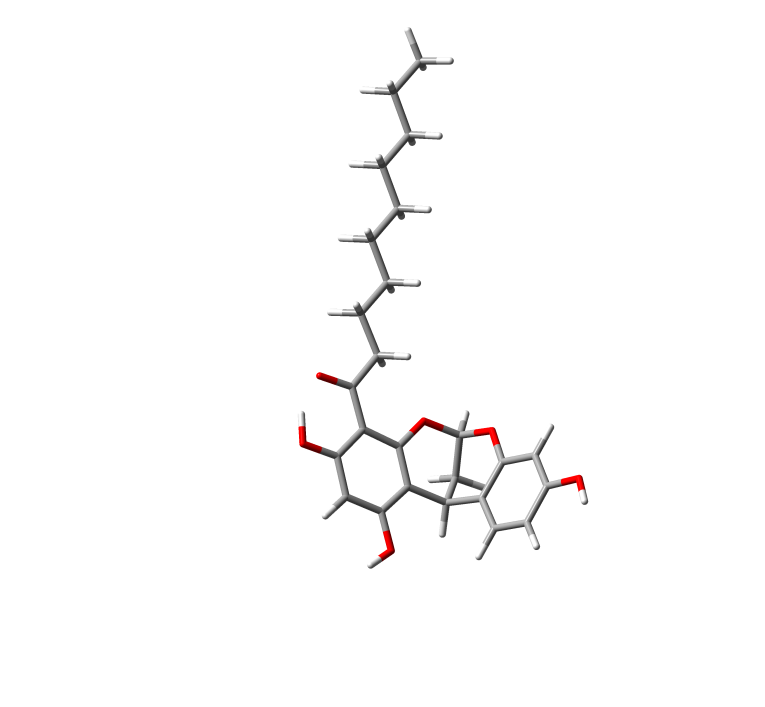** | **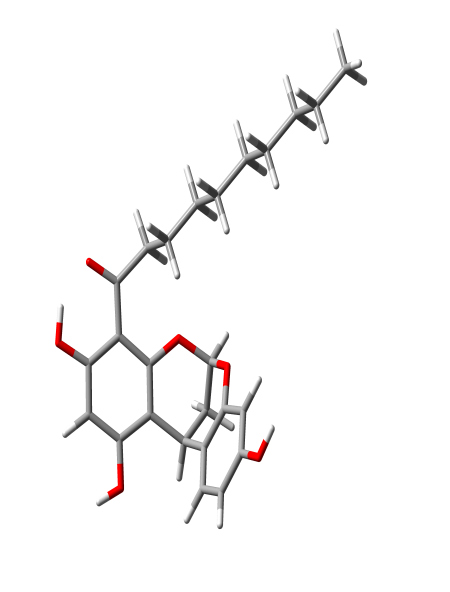** | **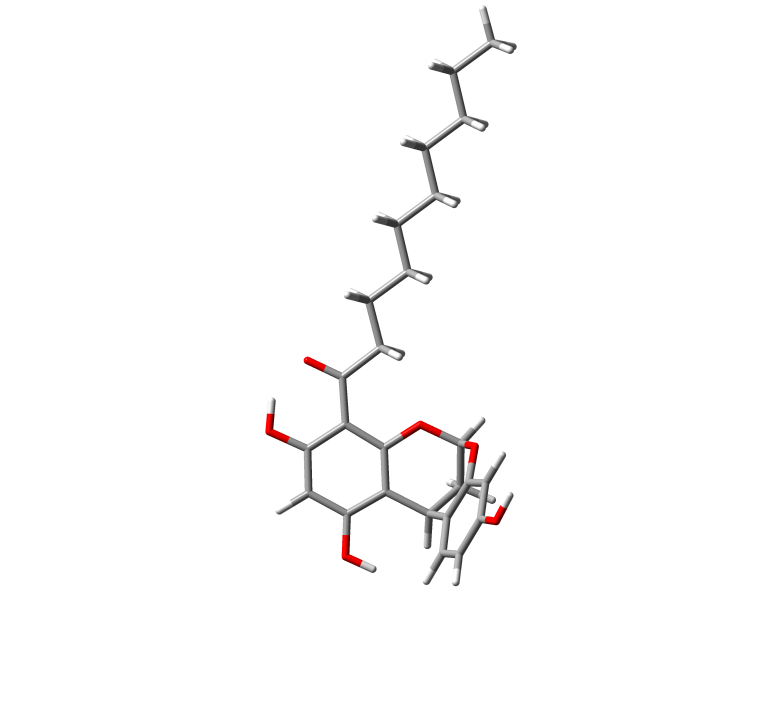** |
| U3-s-x-w-a | U3-s-v-w-a | U3-s-x-w-b | U3-s-x-r-a |
| **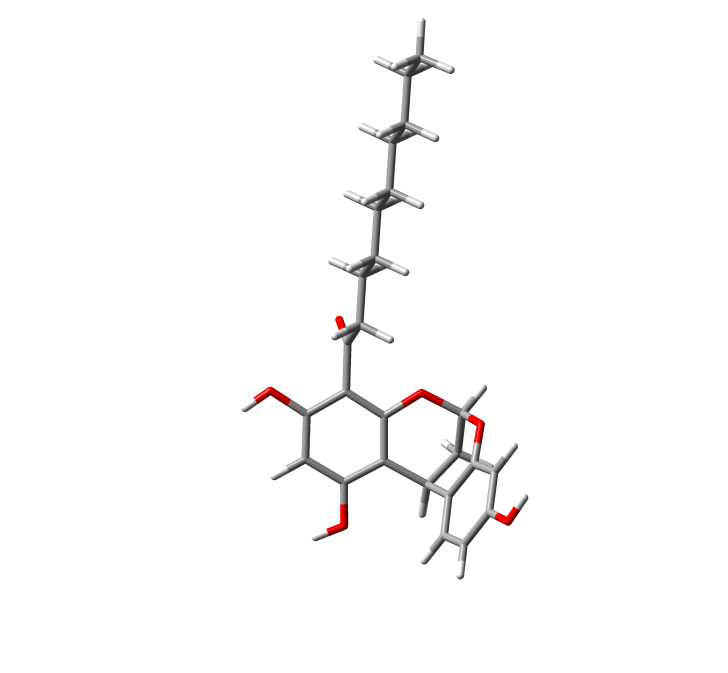** | 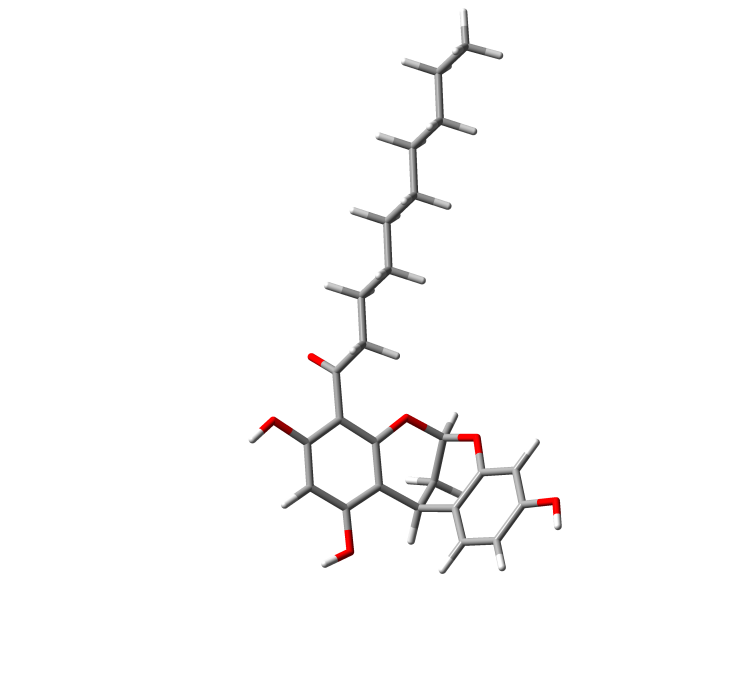 |  |  |
| U3-z-x-w | U3-v-w-a |  |  |
| **Calculated conformers of knipholone** | | | |
| **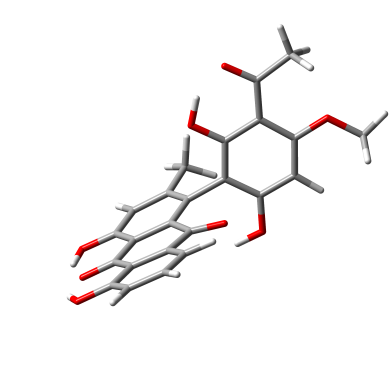** | **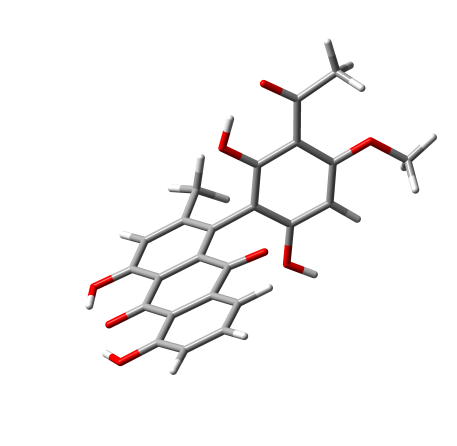** | **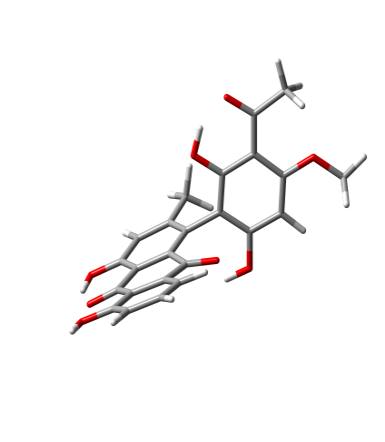** | **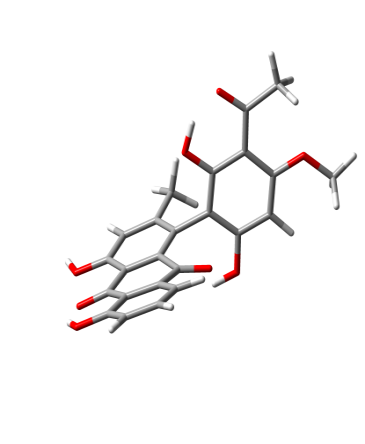** |
| U4-d-ε-r-x-j | U4-d-w-x-j | U4-d-ε-r-v-j | U4-d-ε-r-x-k |
| **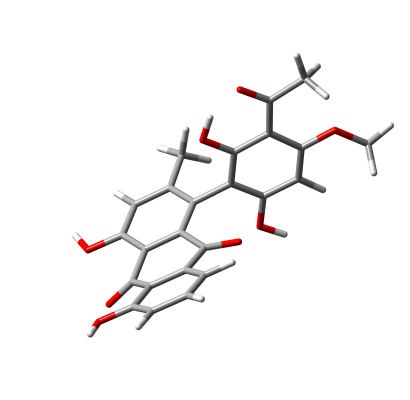** | **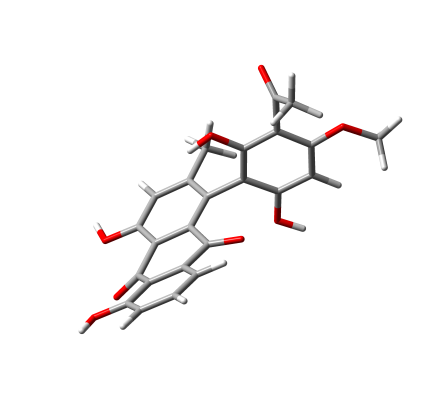** |  |  |
| U4-d-w-v-k | U4-w-v-k |  |  |
| **Calculated conformers of knipholoneanthrone** | | | |
| **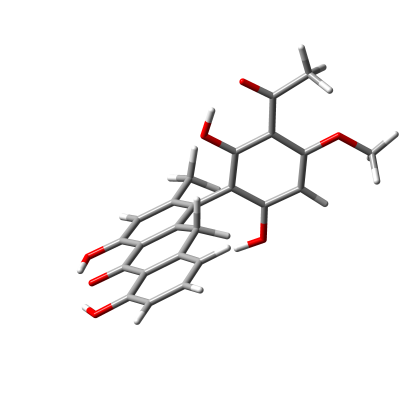** | **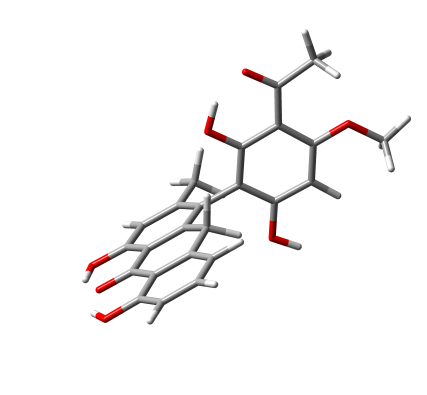** | **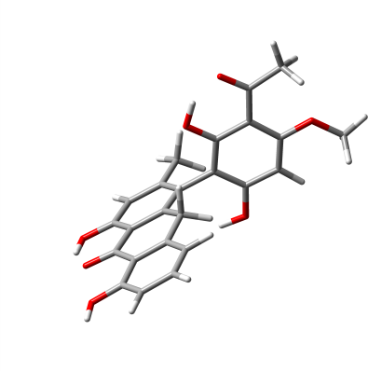** | **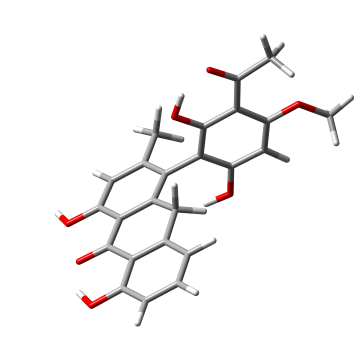** |
| U5-d-r-x-j | U5-d-w-x-j | U5-d-r-v-j | U5-d-r-x-k |
| 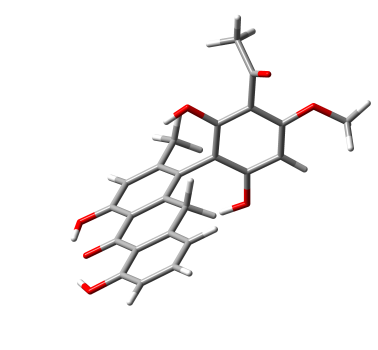 | **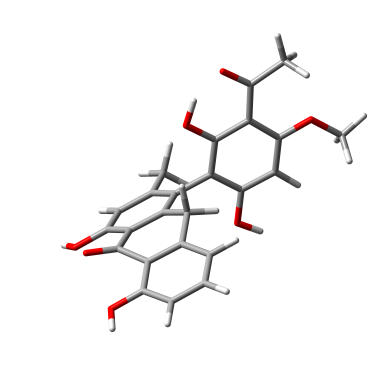** |  |  |
| U5-r-x-j | U5-d-w-v-k |  |  |
| **Calculated conformers of 1-(2,6-dihydroxy-3-methyl-4-((3-methylbut-2-en-1-yl)oxy)phenyl)-3-methylbutan-1-one** | | | |
| **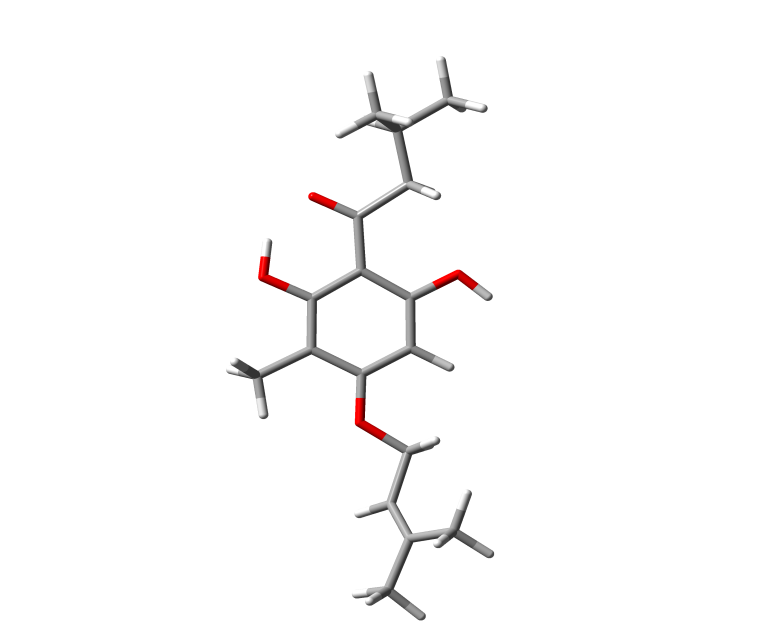** | **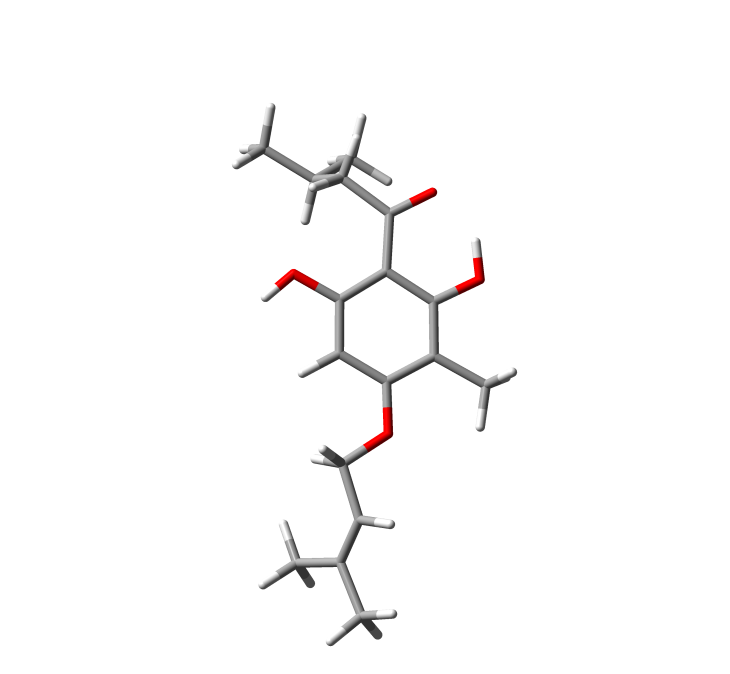** | **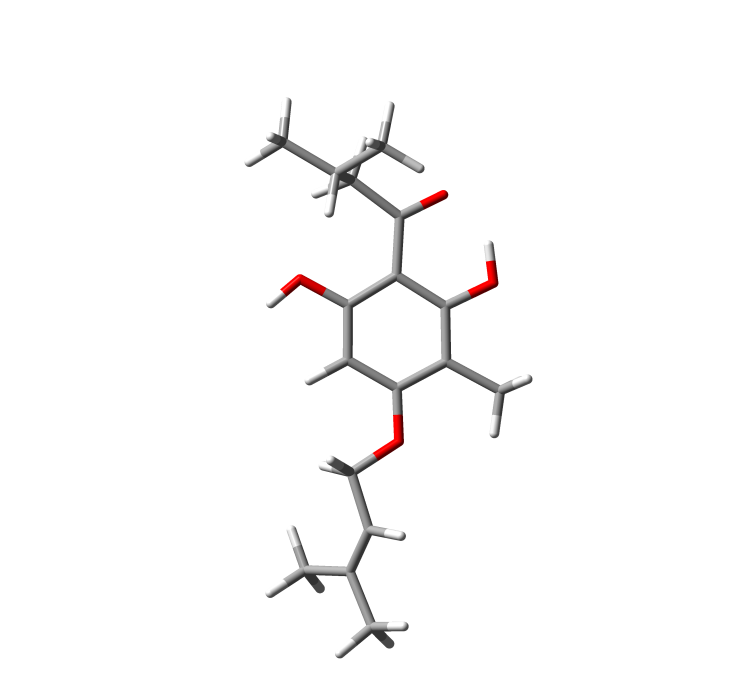** | **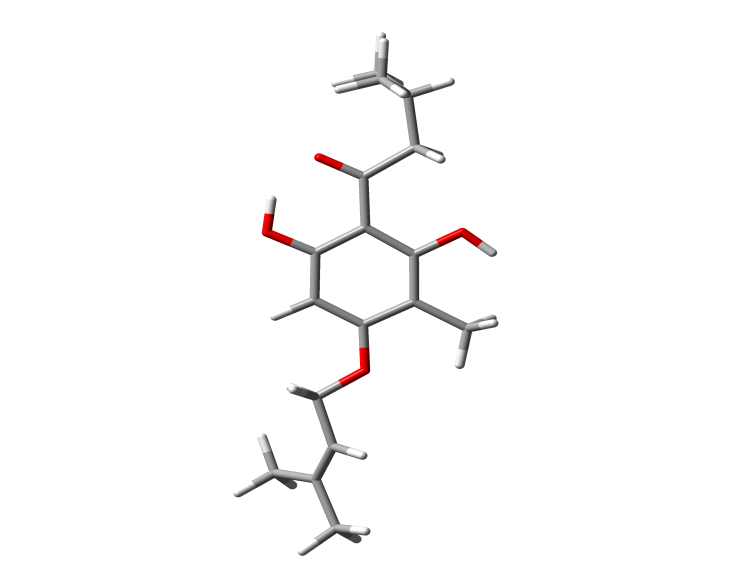** |
| U6-d-w-e | U6-d-w-g | U6-d-w-c | U6-s-w-f |
| 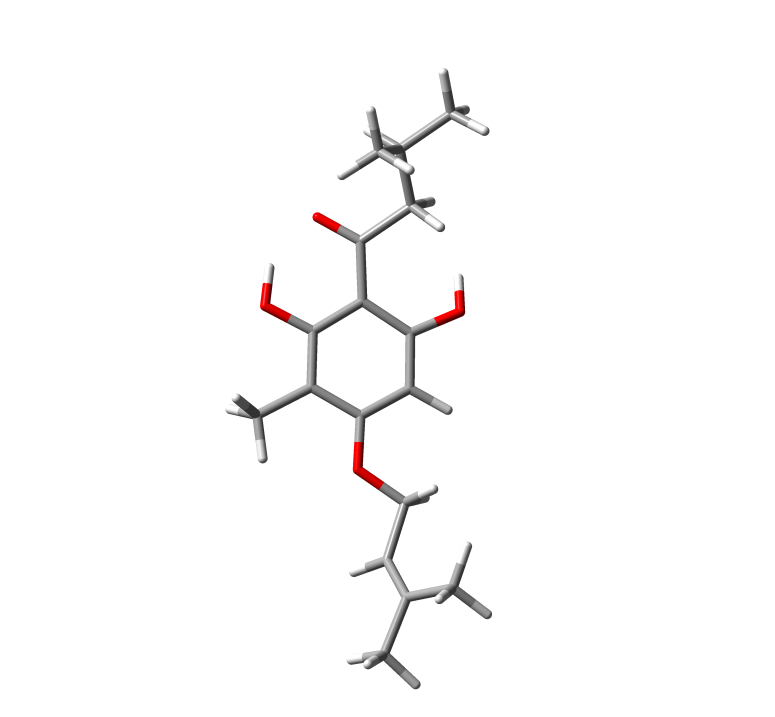 | **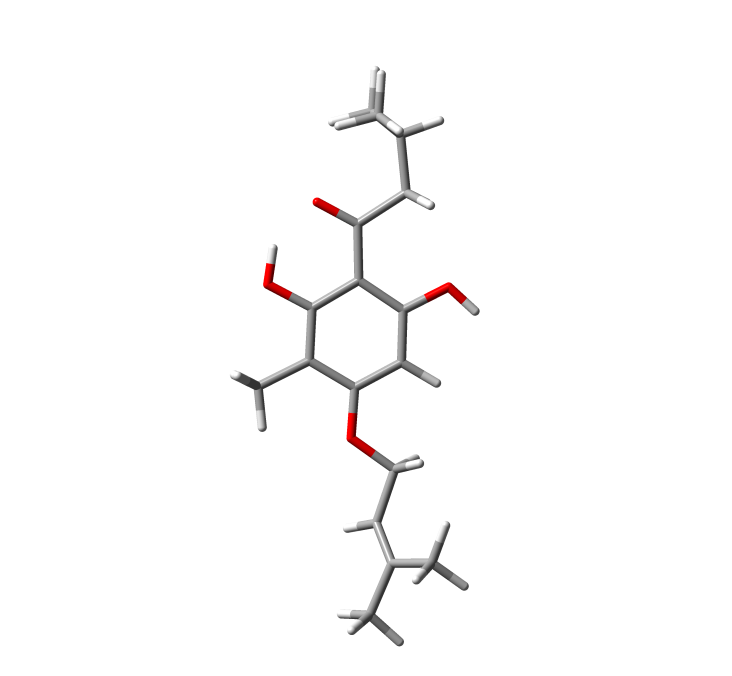** | **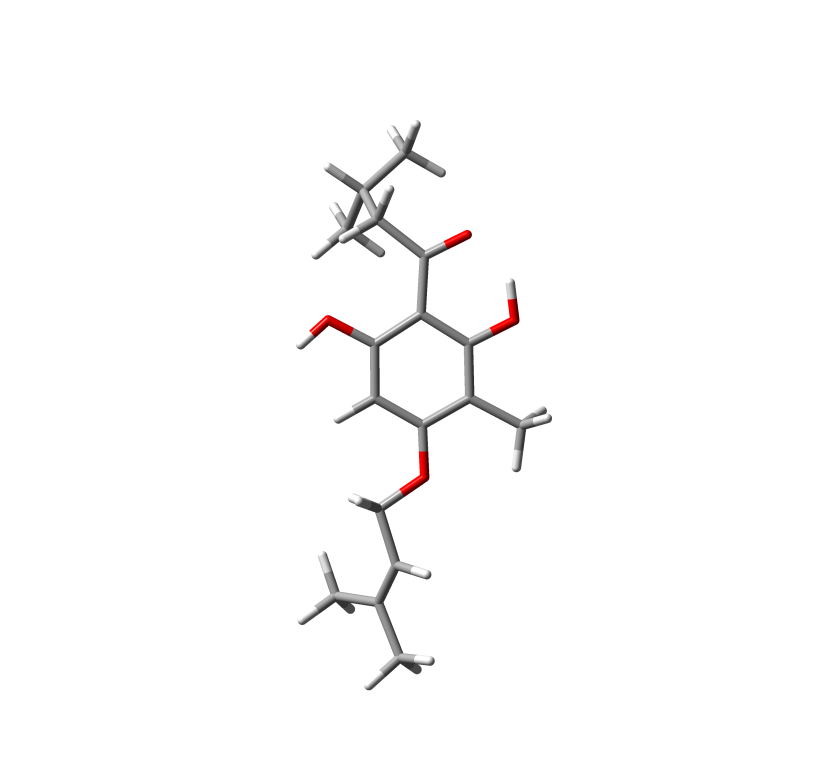** | **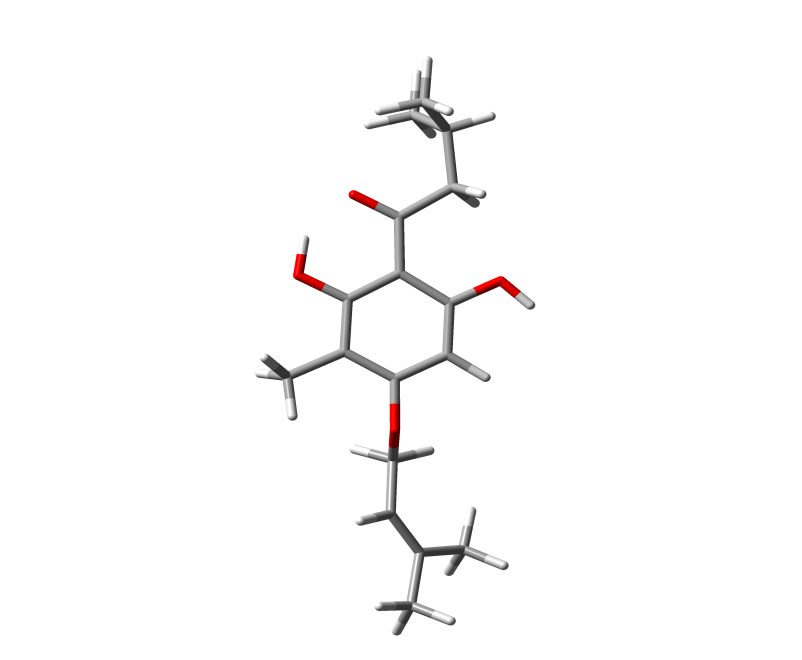** |
| U6-d-w-e-u | U6-d-w-f | U6-d-w-h | U6-d-y-f |
| **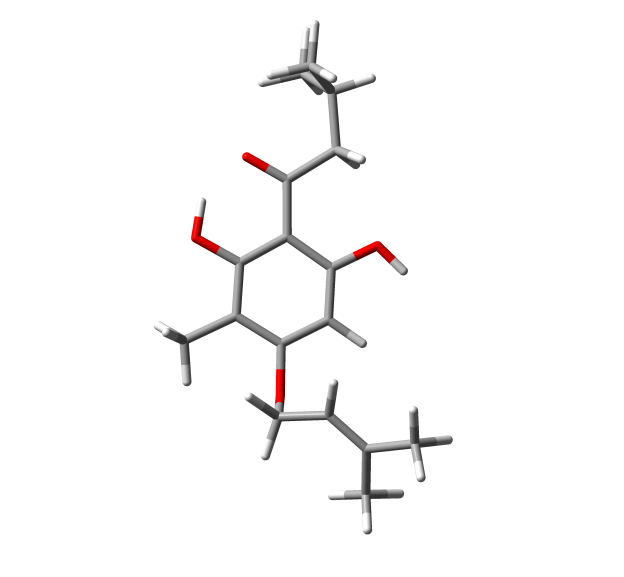** | **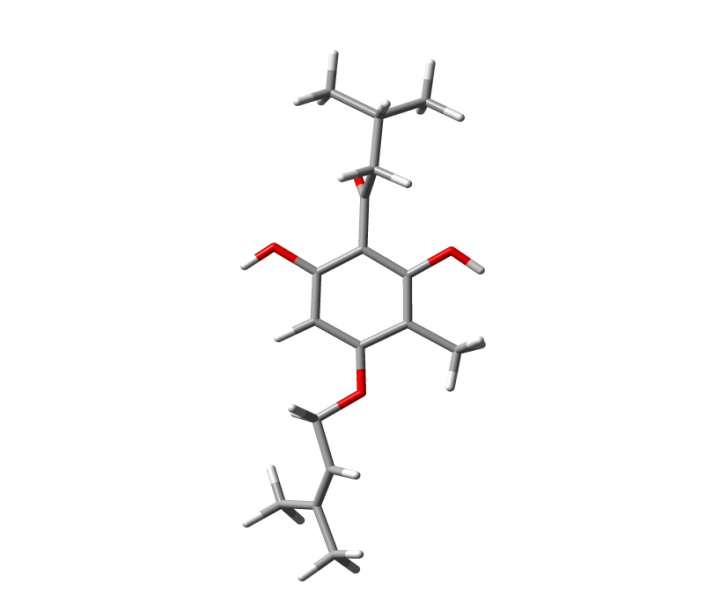** |  |  |
| U6-d-m-f | U6-w-f |  |  |
| **Calculated conformers of antiarone J** | | | |
| **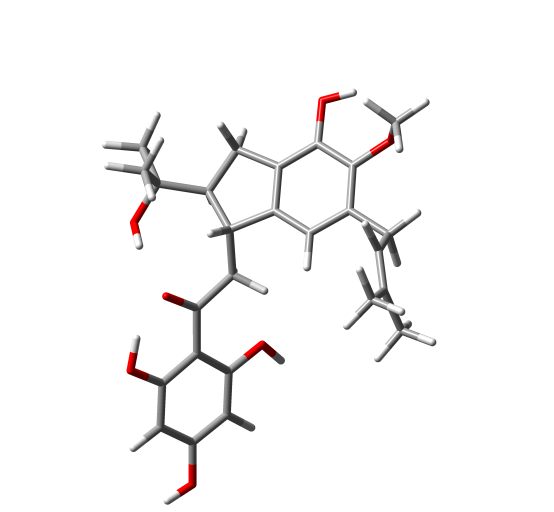** | **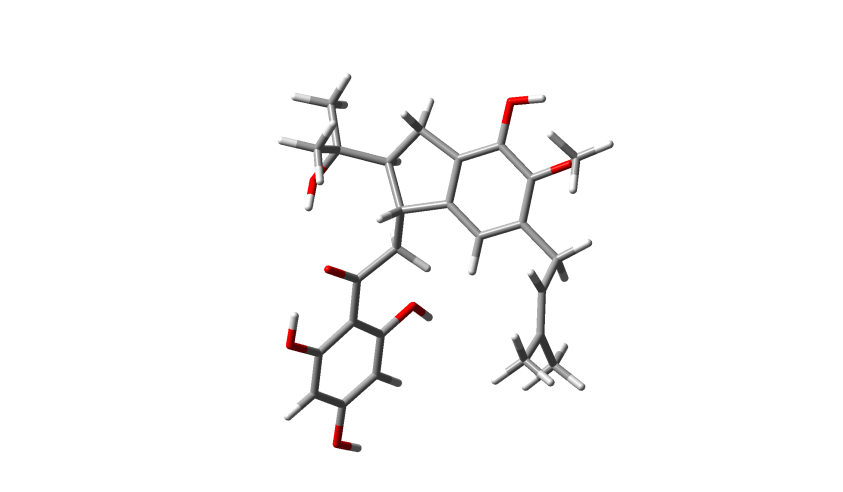** | **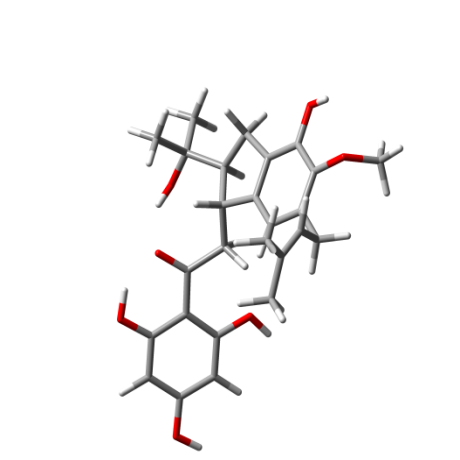** | **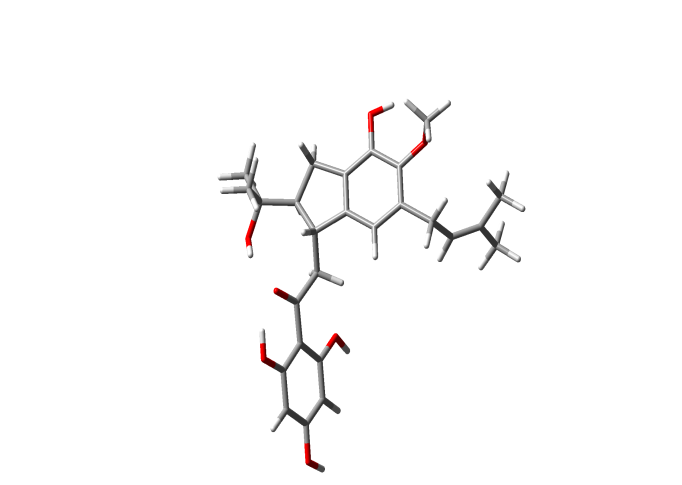** |
| U7-d-r-ᴧ-χ-α-p | U7-d-w-ᴧ-χ-α-p | U7-d-w-ᴧ-χ-α-q | U7-d-w-ᴧ-χ-β-p |
| **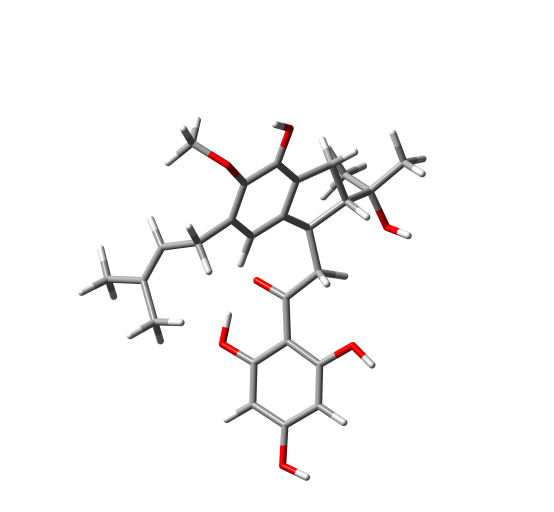** | **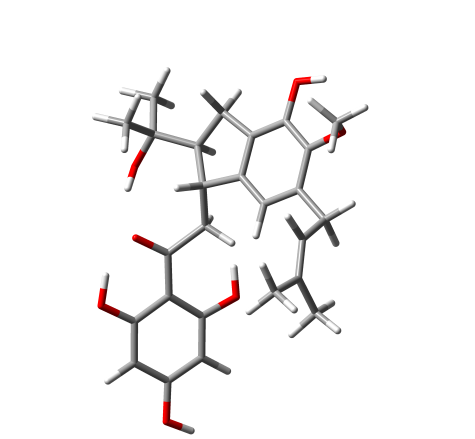** | **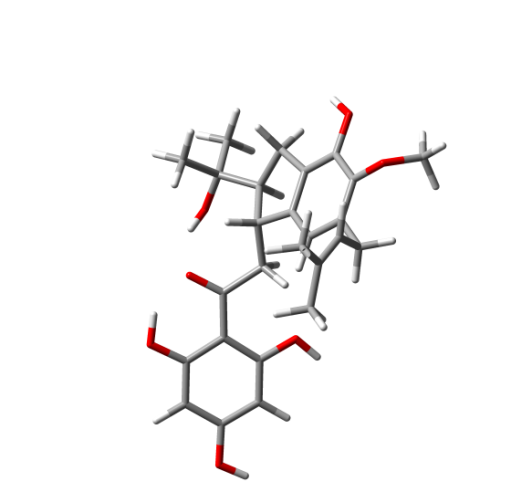** | **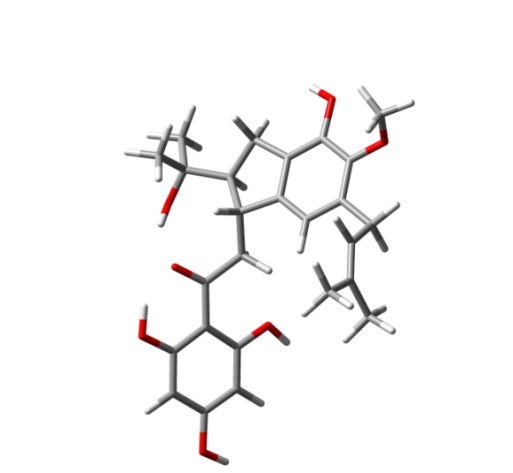** |
| U7-d-w-χ-α-p | U7-d-w-ᴧ-χ-α-p-u | U7-d-w-ᴧ-λ-α-q | U7-d-w-ᴧ-λ-α-p |
| **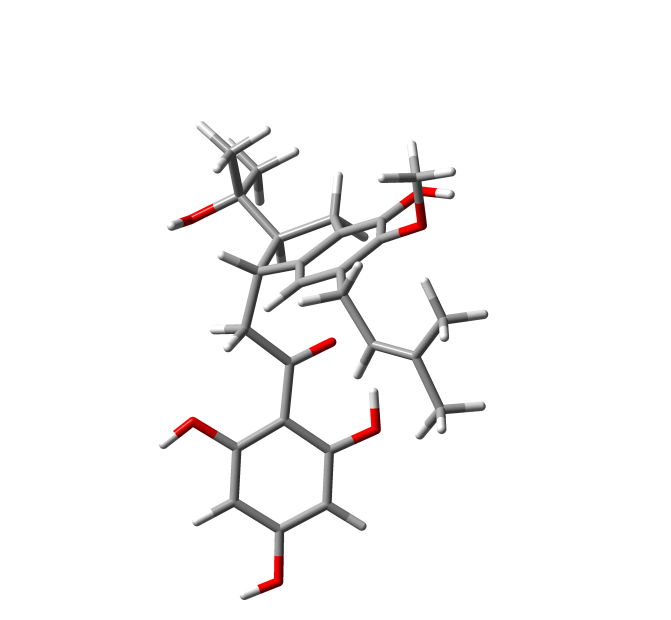** | **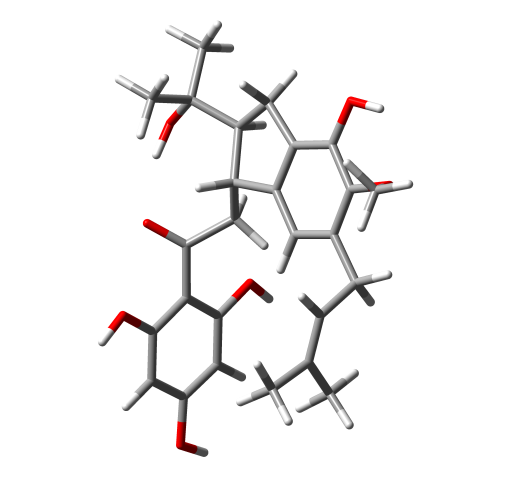** |  |  |
| U7-d-w-γ-χ-p | U7-w-ᴧ-χ-α-p |  |  |
| **Calculated conformers of iriflophenone4-glucoside** | | | |
| **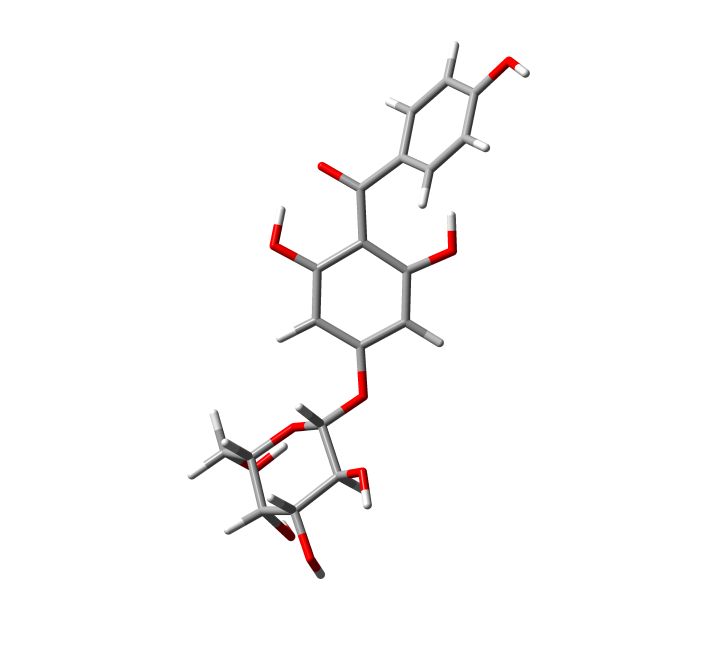** | **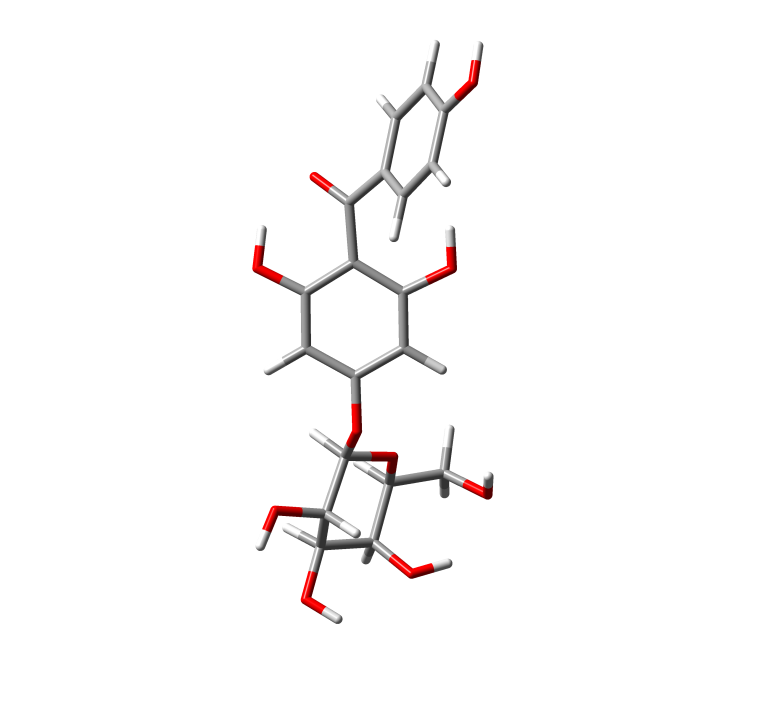** | **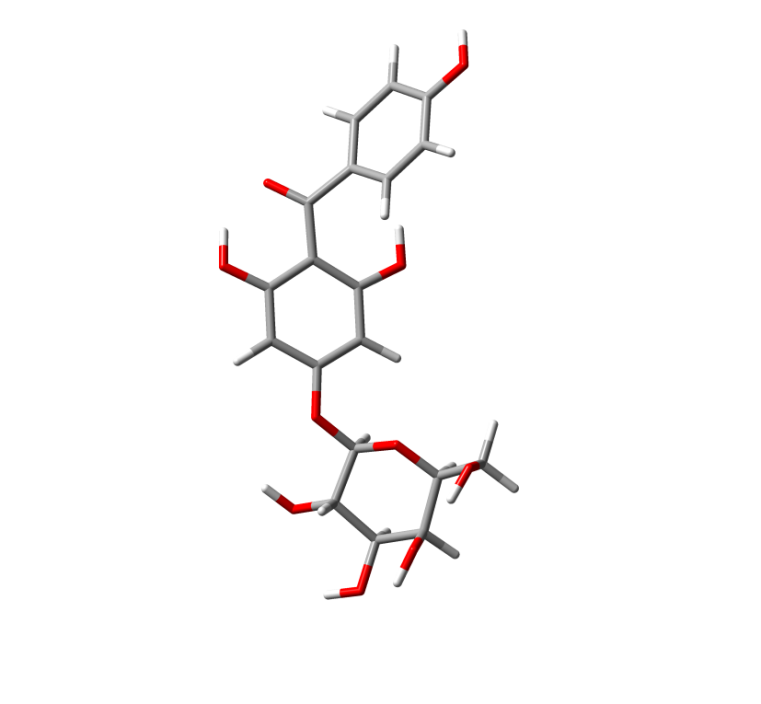** | **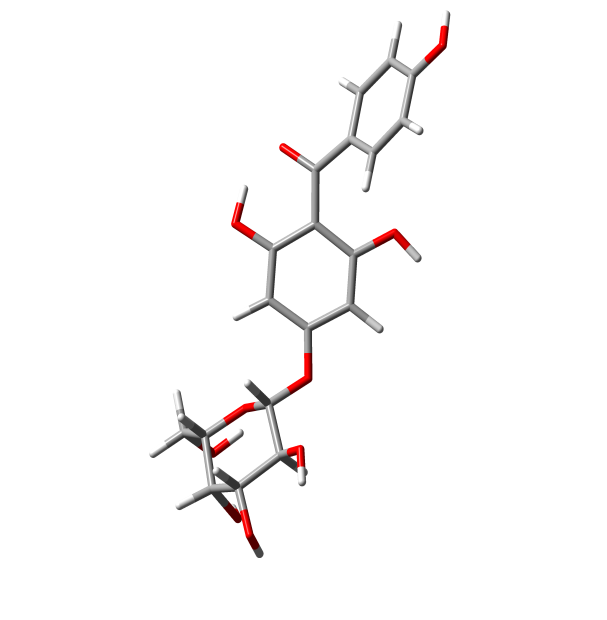** |
| U8-ƞ-d-u-y-κ-ω | U8-ƞ-d-u-y-κ-t | U8-ƞ-d-u-w-μ-t | U8-d-y-κ-ω |
|  |  |  |  |
| **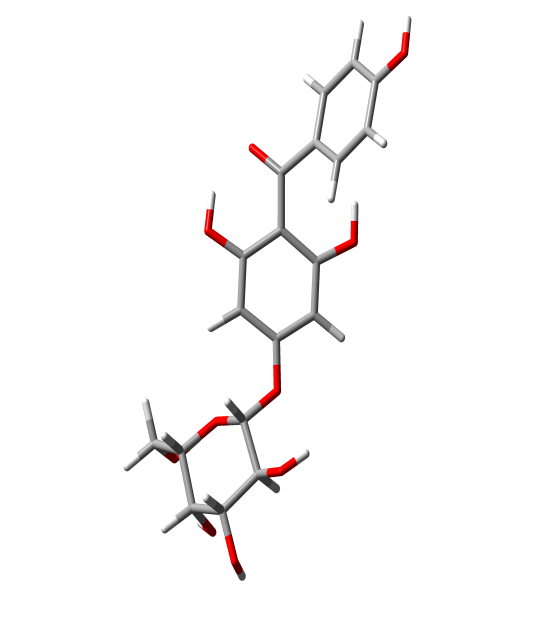** | **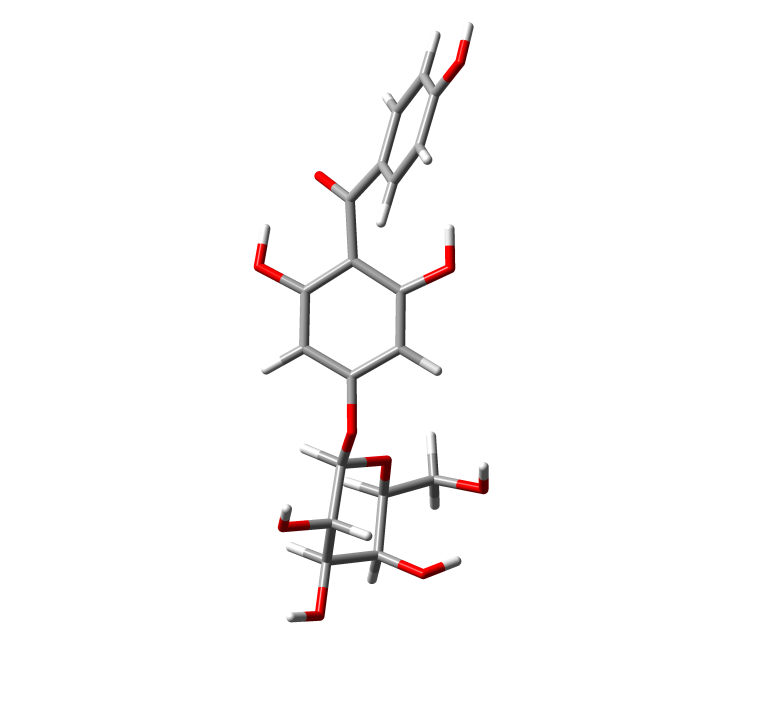** | **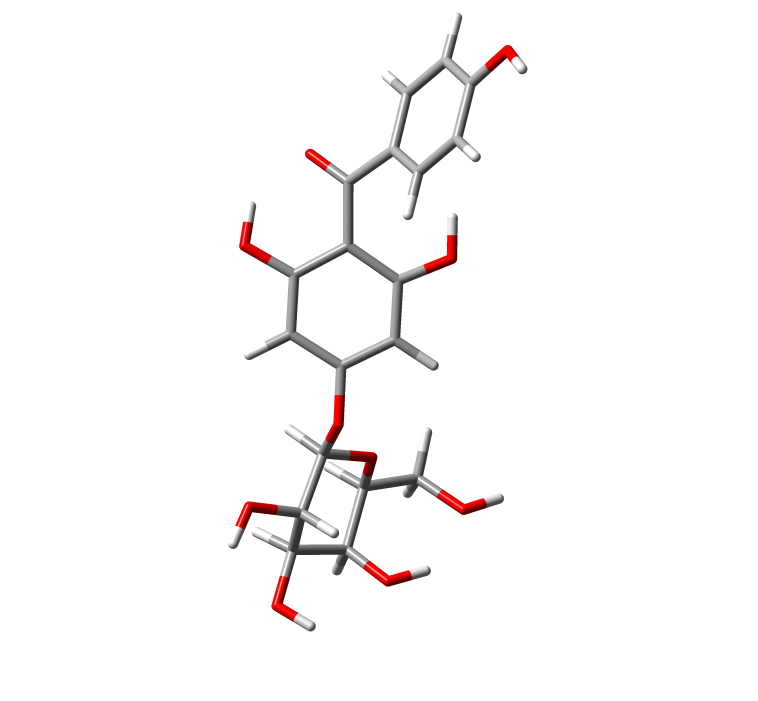** | **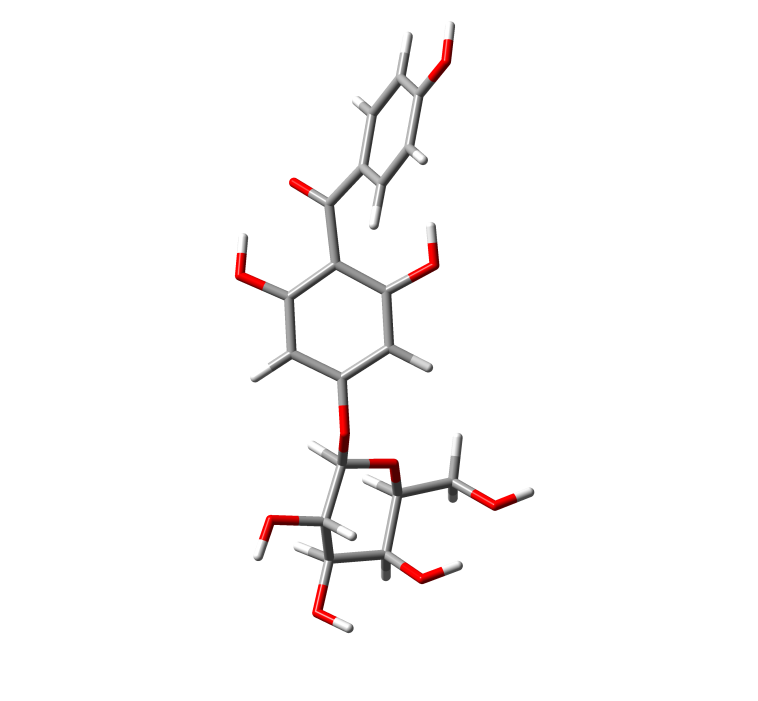** |
| U8-ƞ-d-u-r-ξ-t | U8-ƞ-d-u-y-ς-t | U8-ƞ-d-u-y-δ-ω | U8-ƞ-d-u-y-δ-t |
| **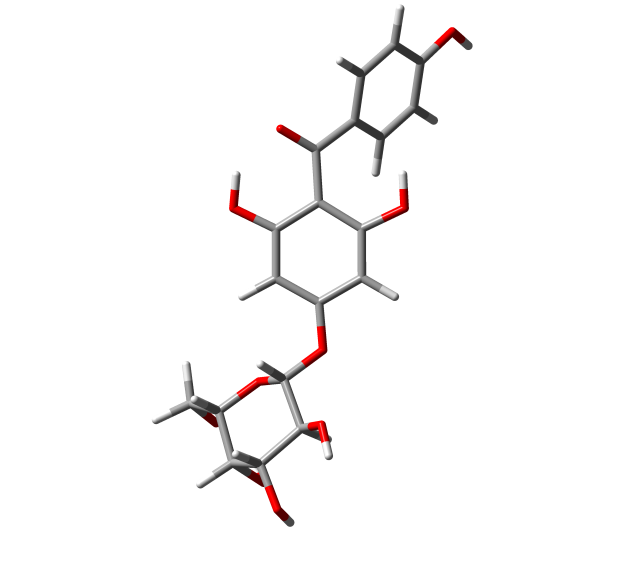** | **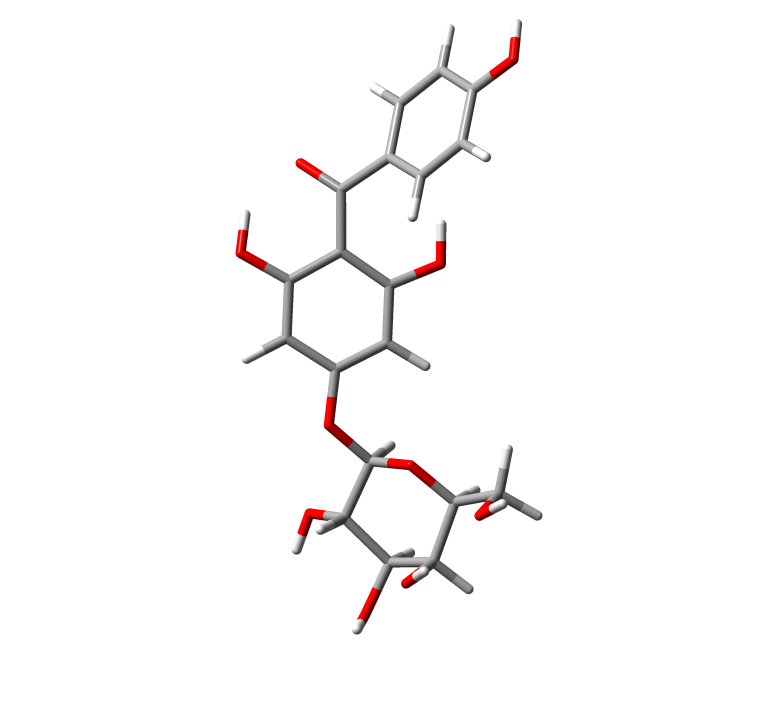** | **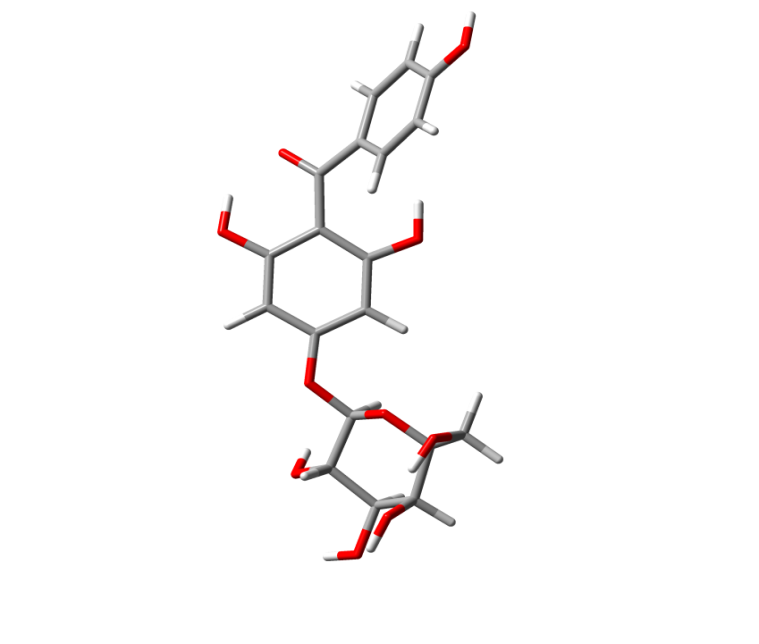** | **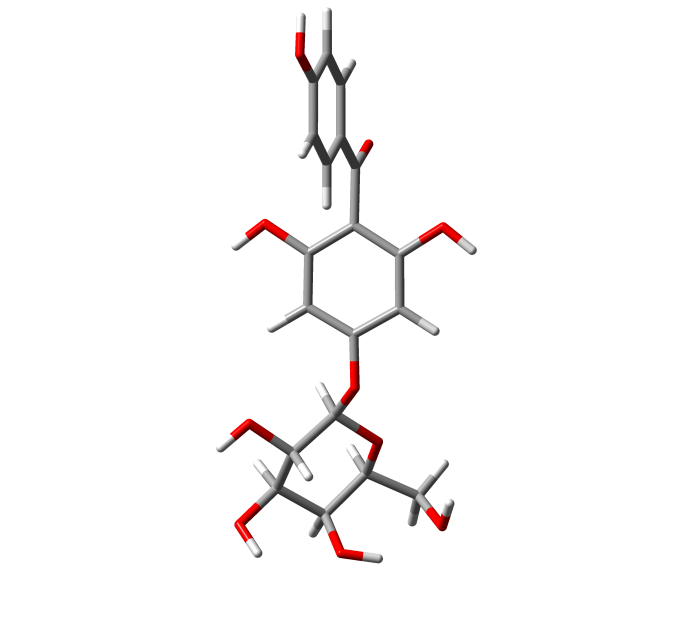** |
| U8-ƞ-d-u-r-δ-n | U8-ƞ-d-u-w-δ-t | U8-ƞ-d-u-w-τ-t | U8-y-κ-ω |
