# Supplementary material for: Computable properties of selected monomeric acylphloroglucinols with anticancer and/or antimalarial activities and first-approximation docking study
Source: J Mol Model. 2025 Mar 12;31(4):113. doi: 10.1007/s00894-025-06299-7 (PMC11903629; doi:10.1007/s00894-025-06299-7)
Supplement: Supplementary file 4 — (DOCX 55.4 KB) [file 894_2025_6299_MOESM4_ESM.docx]

**Figure S4**

**Graphical comparison of the dipole moments of the calculated conformers of the considered ACPL molecules *in vacuo*. I.**

DFT/B3LYP/6-31+G(d,p), HF/6-31G(d,p) and MP2/6-31G(d,p) results from full optimisation calculations, respectively denoted as DFT, HF and MP2 in the figures’ legends.

Each conformer is denoted by a number on the x axis; the correspondence between numbers and conformers is shown in tables before each diagram, where the conformers are denoted with the acronyms indicating their geometric characteristics through the symbols listed in Table 2.

**a) Comparison for thouvenol A(U1)**

Numbers denoting the conformers of thouvenol on the x axis.

| # | Conformers | # | Conformers | # | Conformers |
| --- | --- | --- | --- | --- | --- |
| 1 | U1-d-r-a | 3 | U1-d-u-r-a | 5 | U1-r-a |
| 2 | U1-d-w-a | 4 | U1-d-u-w-a |  |  |

**b) Comparison for myristicyclin A (U2)**

Numbers denoting the conformers of myristicyclin A on the x axis.

| # | Conformers | # | Conformers | # | Conformers |
| --- | --- | --- | --- | --- | --- |
| 1 | U2-d-v-a | 3 | U2-s-v-u-a | 5 | U2-x-a |
| 2 | U2-s-v-a | 4 | U2-d-x-a |  |  |

**c) Comparison for myristicyclin B (U3)**

Numbers denoting the conformers of myristicyclin B on the x axis.

| # | Conformers | # | Conformers | # | Conformers |
| --- | --- | --- | --- | --- | --- |
| 1 | U3-s-x-w-a | 3 | U3-s-x-w-b | 5 | U3-z-x-w |
| 2 | U3-s-v-w-a | 4 | U3-s-x-r-a | 6 | U3-v-w-a |

**d) Comparison for knipholone (U4)**

Numbers denoting the conformers of knipholone on the x axis.

| # | Conformers | # | Conformers | # | Conformers |
| --- | --- | --- | --- | --- | --- |
| 1 | U4-d-ε-r-x-j | 3 | U4-d-ε-r-v-j | 5 | U4-d-w-v-k |
| 2 | U4-d-w-x-j | 4 | U4-d-ε-r-x-k | 6 | U4-w-v-k |

**e) Comparison for knipholoneanthrone (U5)**

Numbers denoting the conformers of knipholoneanthrone on the x axis.

| # | Conformers | # | Conformers | # | Conformers |
| --- | --- | --- | --- | --- | --- |
| 1 | U5-d-r-x-j | 3 | U5-d-r-v-j | 5 | U5-r-x-j |
| 2 | U5-d-w-x-j | 4 | U5-d-r-x-k | 6 | U5-d-w-v-k |

**f) Comparison for 1-(2,6-dihydroxy-3-methyl-4-((3-methylbut-2-en-1-yl)oxy)phenyl)- 3-methylbutan-1-one (U6)**

Numbers denoting the conformers of 1-(2,6-dihydroxy-3-methyl-4-((3-methylbut-2-en-1-yl)oxy)phenyl)-3-methylbutan-1-one on the x axis.

| # | Conformers | # | Conformers | # | Conformers | # | Conformers | # | Conformers |
| --- | --- | --- | --- | --- | --- | --- | --- | --- | --- |
| 1 | U6-d-w-e | 3 | U6-d-w-c | 5 | U6-d-w-e-u | 7 | U6-d-w-h | 9 | U6-d-m-f |
| 2 | U6-d-w-g | 4 | U6-s-w-f | 6 | U6-d-w-f | 8 | U6-d-y-f | 10 | U6-w-f |

**g) Comparison for antiarone J (U7)**

Numbers denoting the conformers of antiarone J on the x axis.

| # | Conformers | # | Conformers | # | Conformers | # | Conformers |
| --- | --- | --- | --- | --- | --- | --- | --- |
| 1 | U8-ƞ-d-u-w-σ-t | 4 | U7-d-w-ᴧ-χ-β-p | 7 | U7-d-w-ᴧ-λ-α-q | 10 | U7-w-ᴧ-χ-α-p |
| 2 | U8-ƞ-d-u-w-τ-t | 5 | U7-d-w-χ-α-p | 8 | U7-d-w-ᴧ-λ-α-p |  |  |
| 3 | U8-y-κ-ω | 6 | U7-d-w-ᴧ-χ-α-p-u | 9 | U7-d-w-γ-χ-p |  |  |

**h) Comparison for iriflophenone4-glucoside (U8)**

Numbers denoting the conformers of iriflophenone4-glucoside on the x axis.

| # | Conformers | # | Conformers | # | Conformers | # | Conformers |
| --- | --- | --- | --- | --- | --- | --- | --- |
| 1 | U8-ƞ-d-u-y-κ-ω | 4 | U8-d-y-κ-ω | 7 | U8-ƞ-d-u-y-δ-ω | 10 | U8-ƞ-d-u-w-δ-t |
| 2 | U8-ƞ-d-u-y-κ-t | 5 | U8-ƞ-d-u-r-ξ-t | 8 | U8-ƞ-d-u-y-δ-t | 11 | U8-ƞ-d-u-w-τ-t |
| 3 | U8-ƞ-d-u-w-μ-t | 6 | U8-ƞ-d-u-y-ς-t | 9 | U8-ƞ-d-u-r-δ-n | 12 | U8-y-κ-ω |
