# Supplementary material for: Computable properties of selected monomeric acylphloroglucinols with anticancer and/or antimalarial activities and first-approximation docking study
Source: J Mol Model. 2025 Mar 12;31(4):113. doi: 10.1007/s00894-025-06299-7 (PMC11903629; doi:10.1007/s00894-025-06299-7)
Supplement: Supplementary file 7 — (DOCX 59.6 KB) [file 894_2025_6299_MOESM7_ESM.docx]

**Figure S7**

**Graphical comparison of the solvation free energy (ΔG_solv_) of the calculated conformers of the considered ACPL molecules in chloroform, acetonitrile and water (respectively denoted as chlrf, actn, and aq in the figures’ legends).**

HF/6-31G(d,p), and DFT/B3LYP/6-31+G(d,p) results from full optimisation PCM calculations, respectively denoted as HF and DFT in the figures’ legends.

Each conformer is denoted by a number on the x axis; the correspondence between numbers and conformers is shown in tables before each diagram, where the conformers are denoted with the acronyms indicating their geometric characteristics through the symbols listed in Table 2.

**a) Comparison for thouvenol A (U1)**

Numbers denoting the conformers of thouvenol A on the x axis.

| # | Conformers | # | Conformers | # | Conformers |
| --- | --- | --- | --- | --- | --- |
| 1 | U1-d-r-a | 3 | U1-d-u-r-a | 5 | U1-r-a |
| 2 | U1-d-w-a | 4 | U1-d-u-w-a |  |  |

**b) Comparison for myristicyclin A (U2)**

Numbers denoting the conformers of myristicyclin A on the x axis.

| # | Conformers | # | Conformers | # | Conformers |
| --- | --- | --- | --- | --- | --- |
| 1 | U2-d-v-a | 3 | U2-s-v-u-a | 5 | U2-x-a |
| 2 | U2-s-v-a | 4 | U2-d-x-a |  |  |

**c) Comparison for myristicyclin B (U3)**

Numbers denoting the conformers of myristicyclin B on the x axis.

| # | Conformers | # | Conformers | # | Conformers |
| --- | --- | --- | --- | --- | --- |
| 1 | U3-s-x-w-a | 3 | U3-s-x-w-b | 5 | U3-z-x-w |
| 2 | U3-s-v-w-a | 4 | U3-s-x-r-a | 6 | U3-v-w-a |

**d) Comparison for knipholone (U4)**

Numbers denoting the conformers of knipholone on the x axis.

| # | Conformers | # | Conformers | # | Conformers |
| --- | --- | --- | --- | --- | --- |
| 1 | U4-d-ε-r-x-j | 3 | U4-d-ε-r-v-j | 5 | U4-d-w-v-k |
| 2 | U4-d-w-x-j | 4 | U4-d-ε-r-x-k | 6 | U4-w-v-k |

**e) Comparison for knipholoneanthrone (U5)**

Numbers denoting the conformers of knipholoneanthrone on the x axis.

| # | Conformers | # | Conformers | # | Conformers |
| --- | --- | --- | --- | --- | --- |
| 1 | U5-d-r-x-j | 3 | U5-d-r-v-j | 5 | U5-r-x-j |
| 2 | U5-d-w-x-j | 4 | U5-d-r-x-k | 6 | U5-d-w-v-k |

**f) Comparison for 1-(2,6-dihydroxy-3-methyl-4-((3-methylbut-2-en-1-yl)oxy)phenyl)- 3-methylbutan-1-one (U6)**

Numbers denoting the conformers of 1-(2,6-dihydroxy-3-methyl-4-((3-methylbut-2-en-1-yl)oxy)phenyl)-3-methylbutan-1-one on the x axis.

| # | Conformers | # | Conformers | # | Conformers | # | Conformers | # | Conformers |
| --- | --- | --- | --- | --- | --- | --- | --- | --- | --- |
| 1 | U6-d-w-e | 3 | U6-d-w-c | 5 | U6-d-w-e-u | 7 | U6-d-w-h | 9 | U6-d-m-f |
| 2 | U6-d-w-g | 4 | U6-s-w-f | 6 | U6-d-w-f | 8 | U6-d-y-f | 10 | U6-w-f |

**g) Comparison for antiarone J (U7)**

Numbers denoting the conformers of antiarone J on the x axis.

| # | Conformers | # | Conformers | # | Conformers | # | Conformers |
| --- | --- | --- | --- | --- | --- | --- | --- |
| 1 | U7-d-r-ᴧ-χ-α-p | 4 | U7-d-w-ᴧ-χ-β-p | 7 | U7-d-w-ᴧ-λ-α-q | 10 | U7-w-ᴧ-χ-α-p |
| 2 | U7-d-w-ᴧ-χ-α-p | 5 | U7-d-w-χ-α-p | 8 | U7-d-w-ᴧ-λ-α-p |  |  |
| 3 | U7-d-w-ᴧ-χ-α-q | 6 | U7-d-w-ᴧ-χ-α-p-u | 9 | U7-d-w-γ-χ-p |  |  |

**h) Comparison for iriflophenone4-glucoside (U8)**

Numbers denoting the conformers of iriflophenone4-glucoside on the x axis.

| # | Conformers | # | Conformers | # | Conformers | # | Conformers |
| --- | --- | --- | --- | --- | --- | --- | --- |
| 1 | U8-ƞ-d-u-y-κ-ω | 4 | U8-d-y-κ-ω | 7 | U8-ƞ-d-u-y-δ-ω | 10 | U8-ƞ-d-u-w-δ-t |
| 2 | U8-ƞ-d-u-y-κ-t | 5 | U8-ƞ-d-u-r-ξ-t | 8 | U8-ƞ-d-u-y-δ-t | 11 | U8-ƞ-d-u-w-τ-t |
| 3 | U8-ƞ-d-u-w-μ-t | 6 | U8-ƞ-d-u-y-ς-t | 9 | U8-ƞ-d-u-r-δ-n | 12 | U8-y-κ-ω |
