# Supplementary material for: Computable properties of selected monomeric acylphloroglucinols with anticancer and/or antimalarial activities and first-approximation docking study
Source: J Mol Model. 2025 Mar 12;31(4):113. doi: 10.1007/s00894-025-06299-7 (PMC11903629; doi:10.1007/s00894-025-06299-7)
Supplement: Supplementary file 8 — (DOCX 112 KB) [file 894_2025_6299_MOESM8_ESM.docx]

**Figure S8**

**Graphical comparison of the lengths of the O−H⋅⋅⋅X intramolecular hydrogen bonds (IHBs) in the calculated conformers of the considered ACPL molecules *in vacuo* and in chloroform, acetonitrile and water (respectively denoted as vac, chlrf, actn, and aq in the figures’ legends).**

HF/6-31G(d,p), and DFT/B3LYP/6-31+G(d,p) results from full optimisation PCM calculations, respectively denoted as HF, and DFT in the figures’ legends.

The various IHBs are considered individually, across the molecules and conformers in which they are present.

Each conformer is denoted by a number on the x axis; the correspondence between numbers and conformers is shown in tables before each diagram, where the conformers are denoted with the acronyms indicating their geometric characteristics through the symbols listed in Table 2.

**a) Comparison for thouvenol A (U1), H15⋅⋅⋅O14 IHB**

Numbers denoting the conformers of thouvenol A on the x axis.

| # | Conformers | # | Conformers |
| --- | --- | --- | --- |
| 1 | U1-d-r-a | 3 | U1-d-u-r-a |
| 2 | U1-d-w-a | 4 | U1-d-u-w-a |

**b) Comparison for myristicyclin A (U1) and** **knipholone (U4), H15⋅⋅⋅O14 IHB**

Numbers denoting the conformers of myristicyclin A and knipholone on the x axis.

| # | Conformers | # | Conformers | # | Conformers |
| --- | --- | --- | --- | --- | --- |
| 1 | U2-d-v-a | 3 | U4-d-ε-r-x-j | 5 | U4-d-ε-r-v-j |
| 2 | U2-d-x-a | 4 | U4-d-w-x-j | 6 | U4-d-w-v-k |

**c) Comparison for knipholoneanthrone (U5), H15⋅⋅⋅O14 IHB**

Numbers denoting the conformers of knipholoneanthrone on the x axis.

| # | Conformers | # | Conformers | # | Conformers |
| --- | --- | --- | --- | --- | --- |
| 1 | U5-d-r-x-j | 3 | U5-d-r-v-j | 5 | U5-d-w-v-k |
| 2 | U5-d-w-x-j | 4 | U5-d-r-x-k |  |  |

**d) Comparison for 1-(2,6-dihydroxy-3-methyl-4-((3-methylbut-2-en-1-yl)oxy)phenyl)- 3-methylbutan-1-one (U6)**, **H15⋅⋅⋅O14 IHB**

Numbers denoting the conformers of 1-(2,6-dihydroxy-3-methyl-4-((3-methylbut-2-en-1-yl)oxy)phenyl)-3-methylbutan-1-one (U6) on the x axis.

| # | Conformers | # | Conformers | # | Conformers | # | Conformers |
| --- | --- | --- | --- | --- | --- | --- | --- |
| 1 | U6-d-w-e | 3 | U6-d-w-c | 5 | U6-d-w-f | 7 | U6-d-y-f |
| 2 | U6-d-w-g | 4 | U6-d-w-e-u | 6 | U6-d-w-h | 8 | U6-d-m-f |

**e) Comparison for antiarone J (U7), H15⋅⋅⋅O14 IHB**

Numbers denoting the conformers of antiarone J on the x axis.

| # | Conformers | # | Conformers | # | Conformers |
| --- | --- | --- | --- | --- | --- |
| 1 | U7-d-r-ᴧ-χ-α-p | 4 | U7-d-w-ᴧ-χ-β-p | 7 | U7-d-w-ᴧ-λ-α-q |
| 2 | U7-d-w-ᴧ-χ-α-p | 5 | U7-d-w-χ-α-p | 8 | U7-d-w-ᴧ-λ-α-p |
| 3 | U7-d-w-ᴧ-χ-α-q | 6 | U7-d-w-ᴧ-χ-α-p-u | 9 | U7-d-w-γ-χ-p |

**f) Comparison for iriflophenone4-glucoside (U8) H15⋅⋅⋅O14 IHB**

Numbers denoting the conformers of iriflophenone4-glucoside on the x axis.

| # | Conformers | # | Conformers | # | Conformers | # | Conformers |
| --- | --- | --- | --- | --- | --- | --- | --- |
| 1 | U8-ƞ-d-u-y-κ-ω | 4 | U8-d-y-κ-ω | 7 | U8-ƞ-d-u-y-δ-ω | 10 | U8-ƞ-d-u-w-δ-t |
| 2 | U8-ƞ-d-u-y-κ-t | 5 | U8-ƞ-d-u-r-ξ-t | 8 | U8-ƞ-d-u-y-δ-t | 11 | U8-ƞ-d-u-w-τ-t |
| 3 | U8-ƞ-d-u-w-μ-t | 6 | U8-ƞ-d-u-y-ς-t | 9 | U8-ƞ-d-u-r-δ-n |  |  |

# g) Comparison for myristicyclin B (U3), H17⋅⋅⋅O14 IHB

Numbers denoting the conformers of myristicyclin B on the x axis.

| # | Conformers | # | Conformers |
| --- | --- | --- | --- |
| 1 | U3-s-x-w-a | 3 | U3-s-x-w-b |
| 2 | U3-s-v-w-a | 4 | U3-s-x-r-a |

**h) Comparison for myristicyclin A (U1) and 1-(2,6-dihydroxy-3-methyl-4-((3- methylbut-2-en-1-yl)oxy)phenyl)-3-methylbutan-1-one (U6), H17⋅⋅⋅O14 IHB**

Numbers denoting the conformers of myristicyclin A (U1) and 1-(2,6-dihydroxy-3-methyl-4-((3-methylbut-2-en-1-yl)oxy)phenyl)-3-methylbutan-1-one (U6) on the x axis.

| # | Conformers | # | Conformers |
| --- | --- | --- | --- |
| 1 | U2-s-v-a | 3 | U6-s-w-f |
| 2 | U2-s-v-u-a |  |  |

**i) Comparison for knipholone (U4) and knipholoneanthrone (U5),** **H23···O32 IHB**

Numbers denoting the conformers of knipholone (U4) and knipholoneanthrone on the x axis.

| # | Conformers | # | Conformers | # | Conformers | # | Conformers |
| --- | --- | --- | --- | --- | --- | --- | --- |
| 1 | U4-d-ε-r-x-j | 3 | U4-d-ε-r-v-j | 5 | U5-d-w-x-j | 7 | U5-r-x-j |
| 2 | U4-d-w-x-j | 4 | U5-d-r-x-j | 6 | U5-d-r-v-j |  |  |

**j) Comparison for knipholone (U4) and knipholoneanthrone (U5), H26···O32 IHB**

Numbers denoting the conformers of knipholone (U4) and knipholoneanthrone (U5) on the x axis.

| # | Conformers | # | Conformers |  |  |
| --- | --- | --- | --- | --- | --- |
| 1 | U4-d-ε-r-x-j | 3 | U5-d-r-x-j | 5 | U5-d-r-x-k |
| 2 | U4-d-w-x-j | 4 | U5-d-w-x-j | 6 | U5-r-x-j |

**k) Comparison for antiarone J (U7),** **H26···O14 IHB**

Numbers denoting the conformers of antiarone J on the x axis.

| # | Conformers | # | Conformers |  |  |
| --- | --- | --- | --- | --- | --- |
| 1 | U7-d-r-ᴧ-χ-α-p | 3 | U7-d-w-ᴧ-χ-α-q | 5 | U7-d-w-ᴧ-χ-α-p-u |
| 2 | U7-d-w-ᴧ-χ-α-p | 4 | U7-d-w-ᴧ-χ-β-p | 6 | U7-d-w-χ-α-p |

***l*) Comparison for iriflophenone4-glucoside (U8),**  **H26···O10 IHB**

Numbers denoting the conformers of iriflophenone4-glucoside (U8) on the x axis.

| # | Conformers | # | Conformers |
| --- | --- | --- | --- |
| 1 | U8-ƞ-d-u-w-μ-t | 3 | U8-ƞ-d-u-y-ς-t |
| 2 | U8-ƞ-d-u-r-ξ-t |  |  |

**m) Comparison for iriflophenone4-glucoside (U8),** **H26···O28 IHB**

Numbers denoting the conformers of iriflophenone4-glucoside (U8) on the x axis.

| # | Conformers | # | Conformers | # | Conformers |
| --- | --- | --- | --- | --- | --- |
| 1 | U8-ƞ-d-u-y-κ-t | 3 | U8-ƞ-d-u-y-δ-t | 5 | U8-ƞ-d-u-w-δ-t |
| 2 | U8-ƞ-d-u-y-δ-ω | 4 | U8-ƞ-d-u-r-δ-n |  |  |

**n) Comparison for iriflophenone4-glucoside (U8),** **H28···O25 IHB**

Numbers denoting the conformers of iriflophenone4-glucoside (U8) on the x axis.

| # | Conformers | # | Conformers |
| --- | --- | --- | --- |
| 1 | U8-ƞ-d-u-w-τ-t | 3 | U8-ƞ-d-u-y-ς-t |
| 2 | U8-ƞ-d-u-w-μ-t |  |  |

# o) Comparison for iriflophenone4-glucoside (U8), H28···O29,IHB

Numbers denoting the conformers of iriflophenone4-glucoside (U8) on the x axis.

| # | Conformers | # | Conformers | # | Conformers |
| --- | --- | --- | --- | --- | --- |
| 1 | U8-ƞ-d-u-r-ξ-t | 3 | U8-ƞ-d-u-y-δ-ω | 5 | U8-ƞ-d-u-r-δ-n |
| 2 | U8-ƞ-d-u-y-κ-t | 4 | U8-ƞ-d-u-y-δ-t | 6 | U8-ƞ-d-u-w-δ-t |

**p) Comparison for iriflophenone4-glucoside (U8),** **H30···O27 IHB**

Numbers denoting the conformers of iriflophenone4-glucoside (U8) on the x axis.

| # | Conformers | # | Conformers |
| --- | --- | --- | --- |
| 1 | U8-ƞ-d-u-w-τ-t | 2 | U8-ƞ-d-u-w-μ-t |

**q) Comparison for iriflophenone4-glucoside (U8)**, **H30···O31 IHB**

Numbers denoting the conformers of iriflophenone4-glucoside (U8) on the x axis.

| # | Conformers | # | Conformers | # | Conformers | # | Conformers |
| --- | --- | --- | --- | --- | --- | --- | --- |
| 1 | U8-ƞ-d-u-y-κ-t | 3 | U8-ƞ-d-u-y-ς-t | 5 | U8-ƞ-d-u-y-δ-t | 7 | U8-ƞ-d-u-w-δ-t |
| 2 | U8-ƞ-d-u-r-ξ-t | 4 | U8-ƞ-d-u-y-δ-ω | 6 | U8-ƞ-d-u-r-δ-n |  |  |

**r) Comparison for iriflophenone4-glucoside (U8),** **H32···O29 IHB**

Numbers denoting the conformers of iriflophenone4-glucoside (U8) on the x axis.

| # | Conformers | # | Conformers | # | Conformers |
| --- | --- | --- | --- | --- | --- |
| 1 | U8-ƞ-d-u-r-ξ-t | 3 | U8-ƞ-d-u-y-κ-t | 5 | U8-ƞ-d-u-w-τ-t |
| 2 | U8-ƞ-d-u-y-ς-t | 4 | U8-ƞ-d-u-w-μ-t |  |  |

**s) Comparison for iriflophenone4-glucoside (U8),** **H15···π** **IHB** (H15···C13 distance)

Numbers denoting the conformers of Iriflophenone4-glucoside on the x axis.

| # | Conformers | # | Conformers | # | Conformers | # | Conformers |
| --- | --- | --- | --- | --- | --- | --- | --- |
| 1 | U8-ƞ-d-u-y-κ-ω | 4 | U8-ƞ-d-u-r-ξ-t | 7 | U8-ƞ-d-u-y-δ-t | 10 | U8-ƞ-d-u-w-τ-t |
| 2 | U8-ƞ-d-u-y-κ-t | 5 | U8-ƞ-d-u-y-ς-t | 8 | U8-ƞ-d-u-r-δ-n |  |  |
| 3 | U8-ƞ-d-u-w-μ-t | 6 | U8-ƞ-d-u-y-δ-ω | 9 | U8-ƞ-d-u-w-δ-t |  |  |
