# Supplementary material for: Computable properties of selected monomeric acylphloroglucinols with anticancer and/or antimalarial activities and first-approximation docking study
Source: J Mol Model. 2025 Mar 12;31(4):113. doi: 10.1007/s00894-025-06299-7 (PMC11903629; doi:10.1007/s00894-025-06299-7)

**Figure S11**

**Structures of the receptors selected for docking studies, together with their co-crystallized ligands.**

The binding region or site is enclosed by a red circle. In instances where there are multiple binding sites, letters are used to distinguish them.

1. **Epidermal Growth Factor receptor (EGFR) and co-crystallised ligand. An anticancer target.**


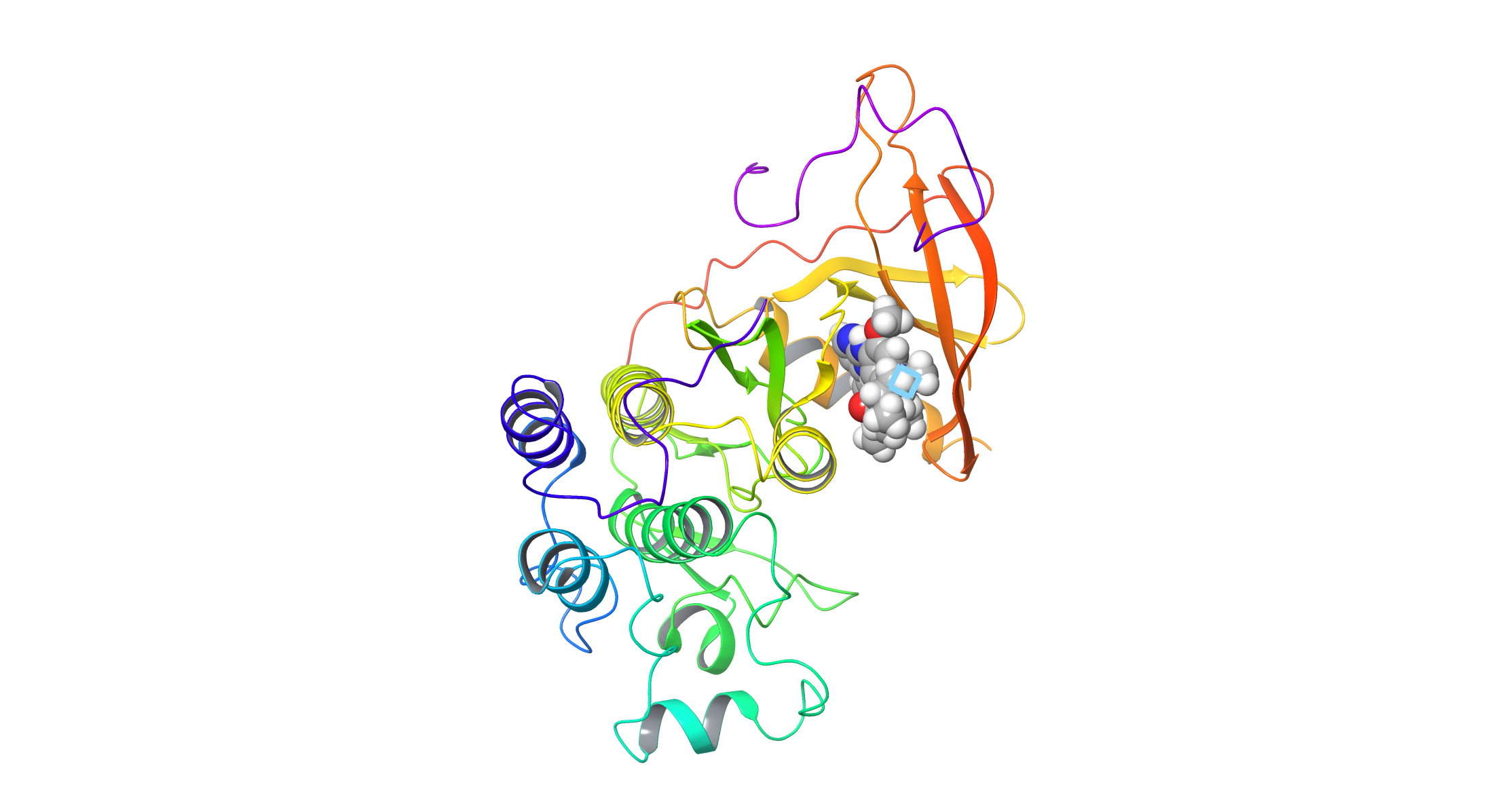


1. **Janus kinase protein (JAK3) and co-crystallised ligand. An anticancer target.**


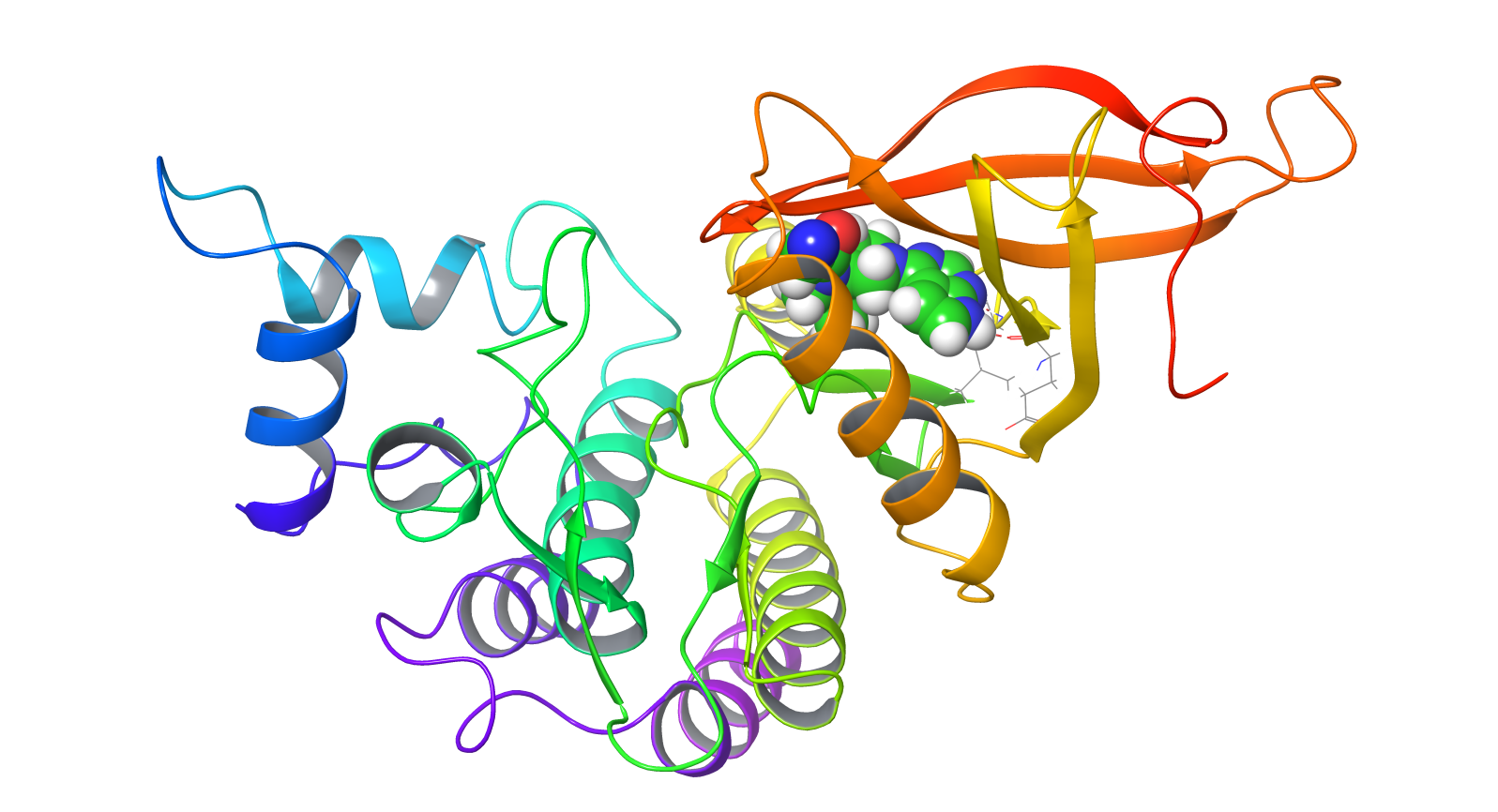


\

1. **Topoisomerase I (Topo I) protein and co-crystallised ligand. An anticancer target.**


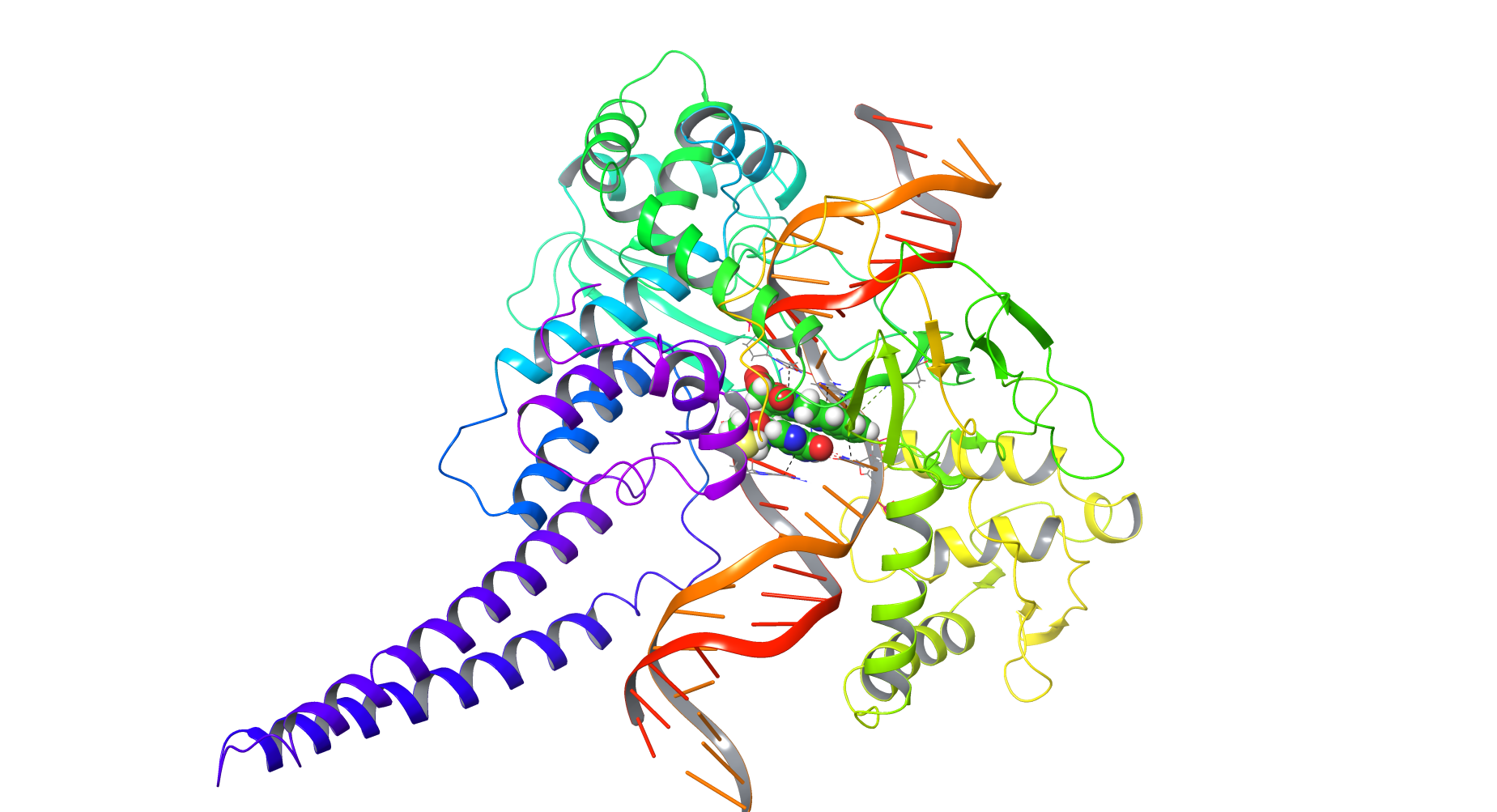


1. **Phosphatidylinositol 3-kinase (PI3K) protein and co-crystallised ligands. An anticancer target.**


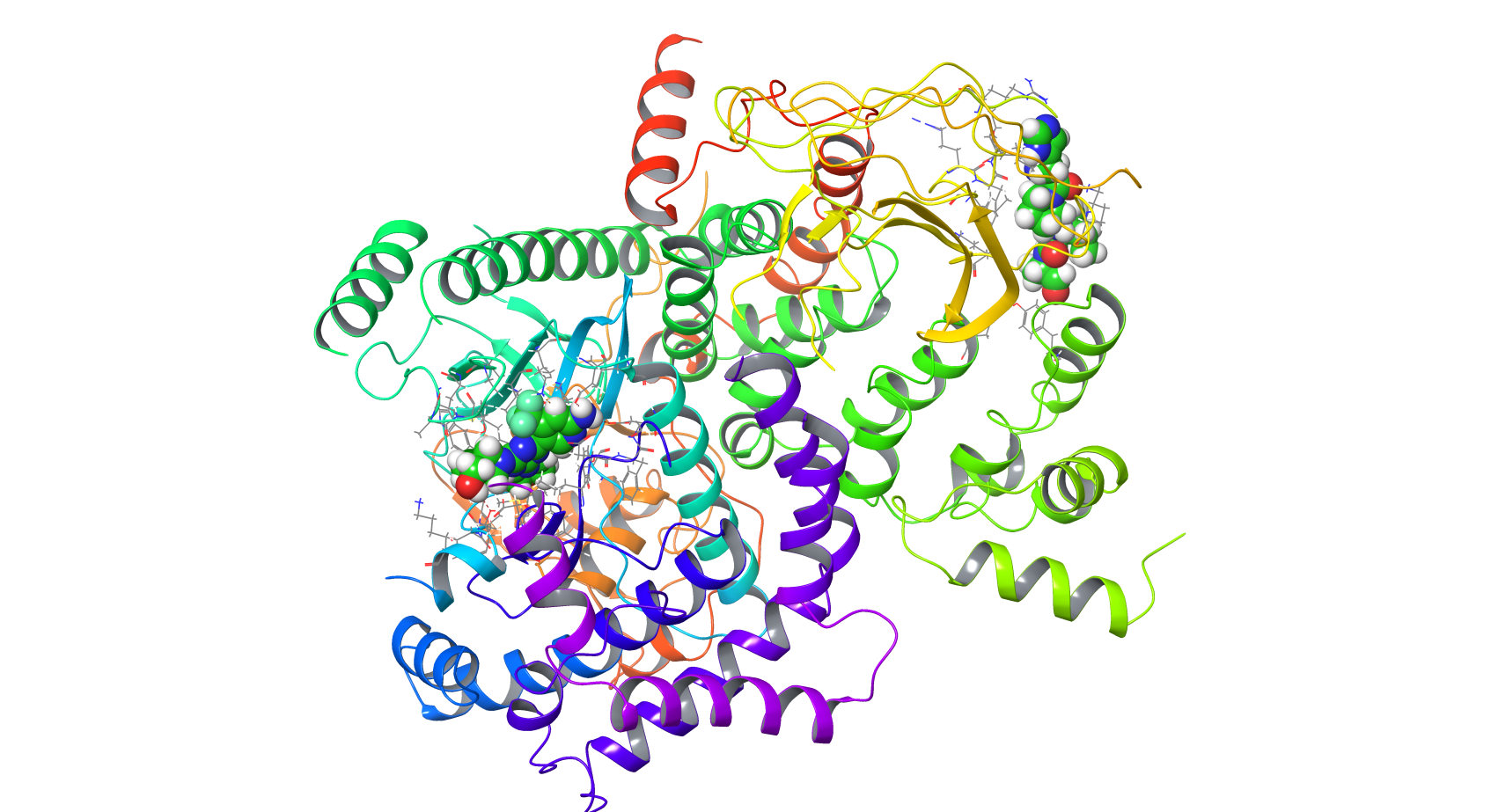


1. **A mutant of BRAF gene (BRAFV600E) and co-crystallised ligands. An anticancer target.**


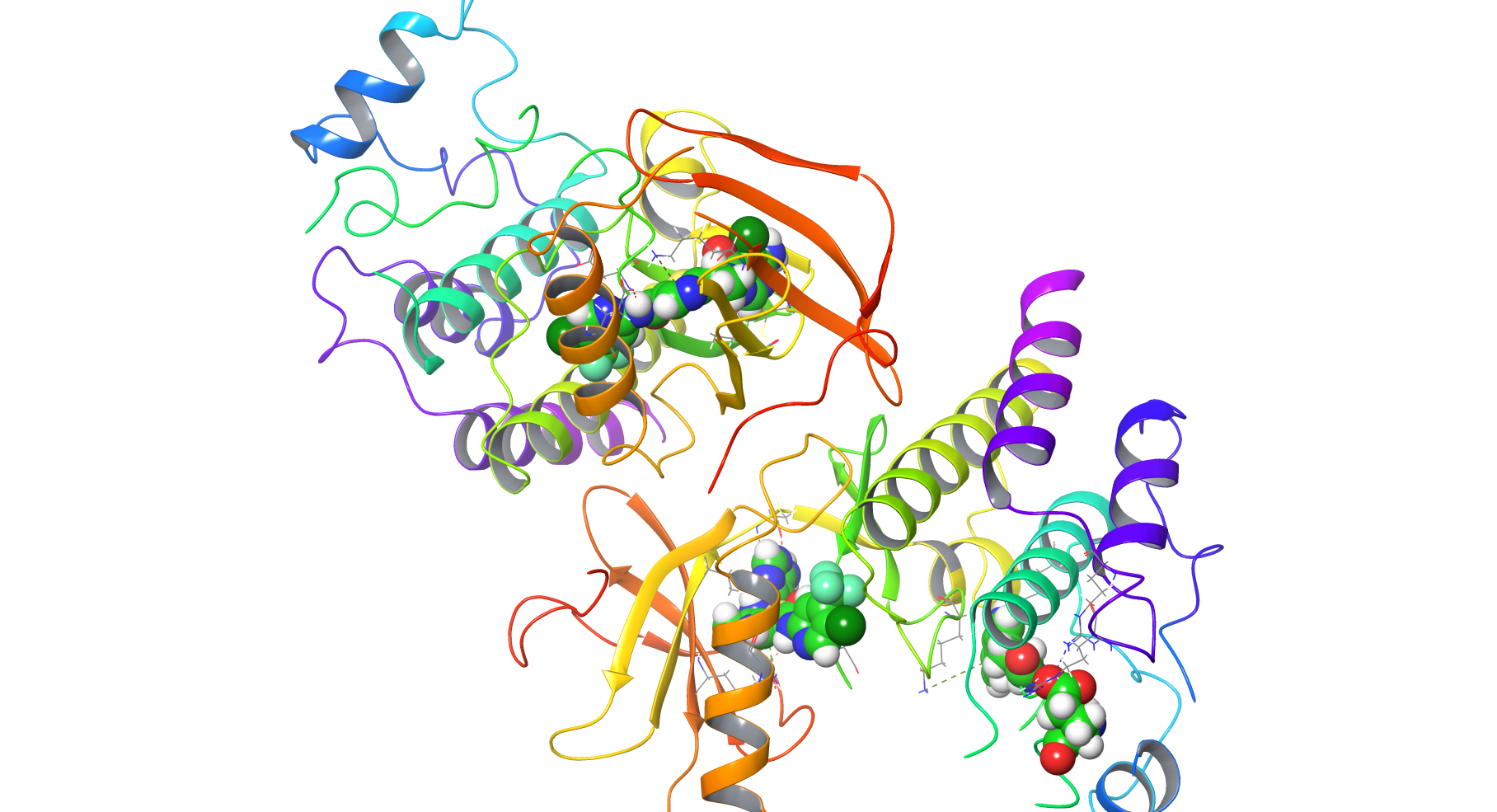


1. **Heat shock protein 90 (HSP90) and co-crystallised ligands. An anticancer target.**


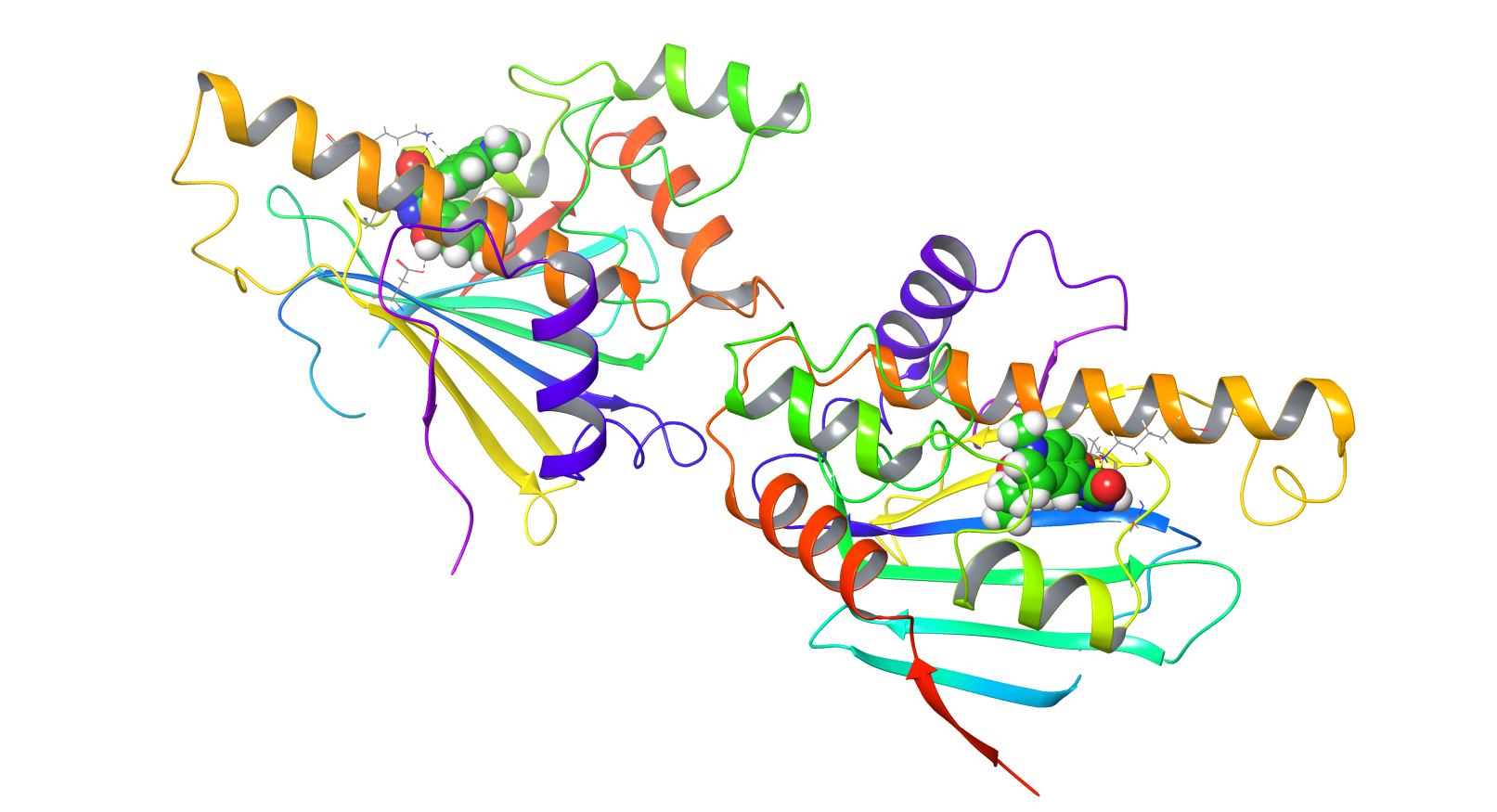


1. **Human epidermal growth factor receptor 2 (HER2) and co-crystallised ligands. An anticancer target.**


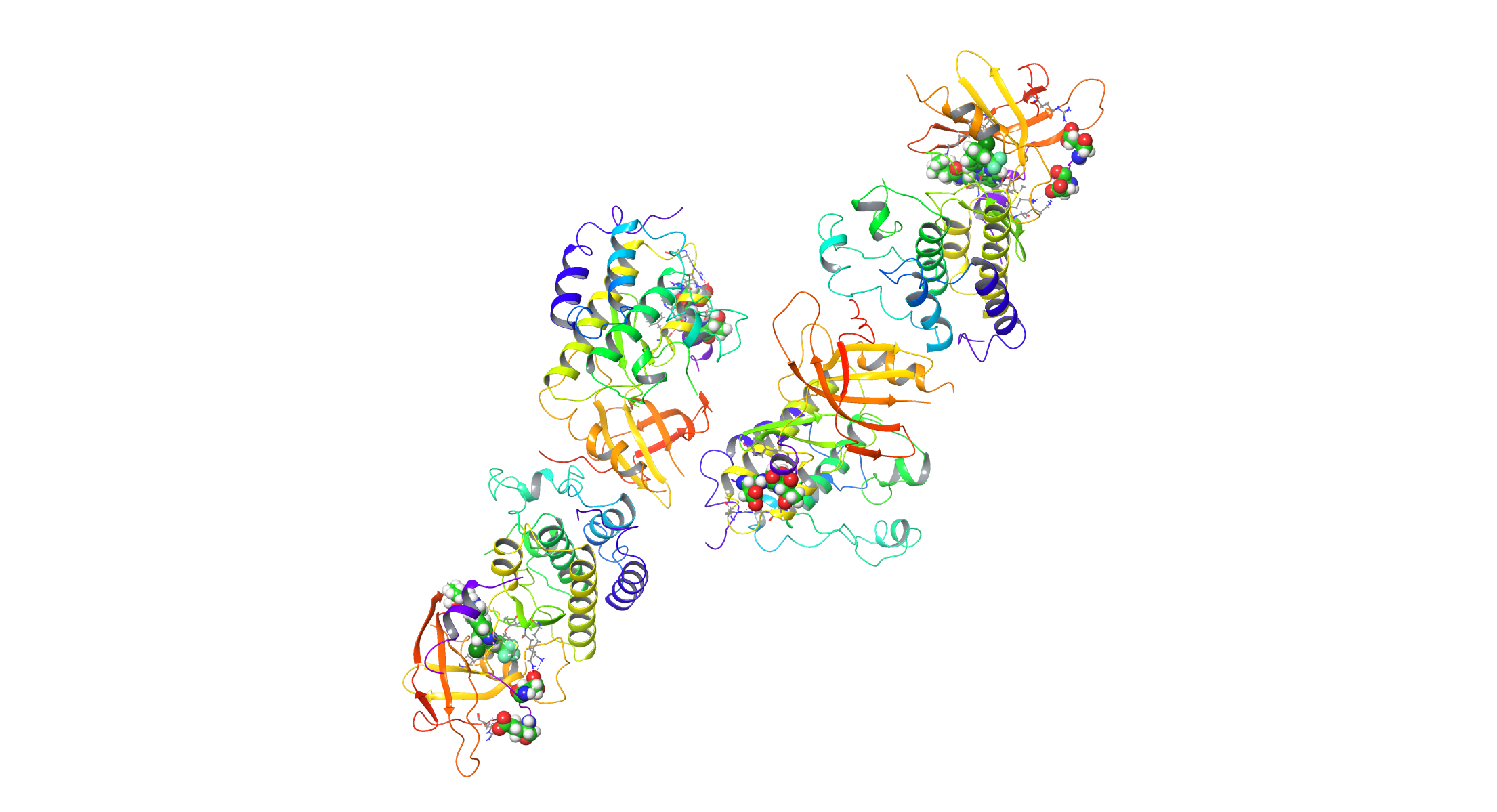


1. **Cyclin-dependent kinase 2 (CDK2) protein and co-crystallised ligand. An anticancer target.**


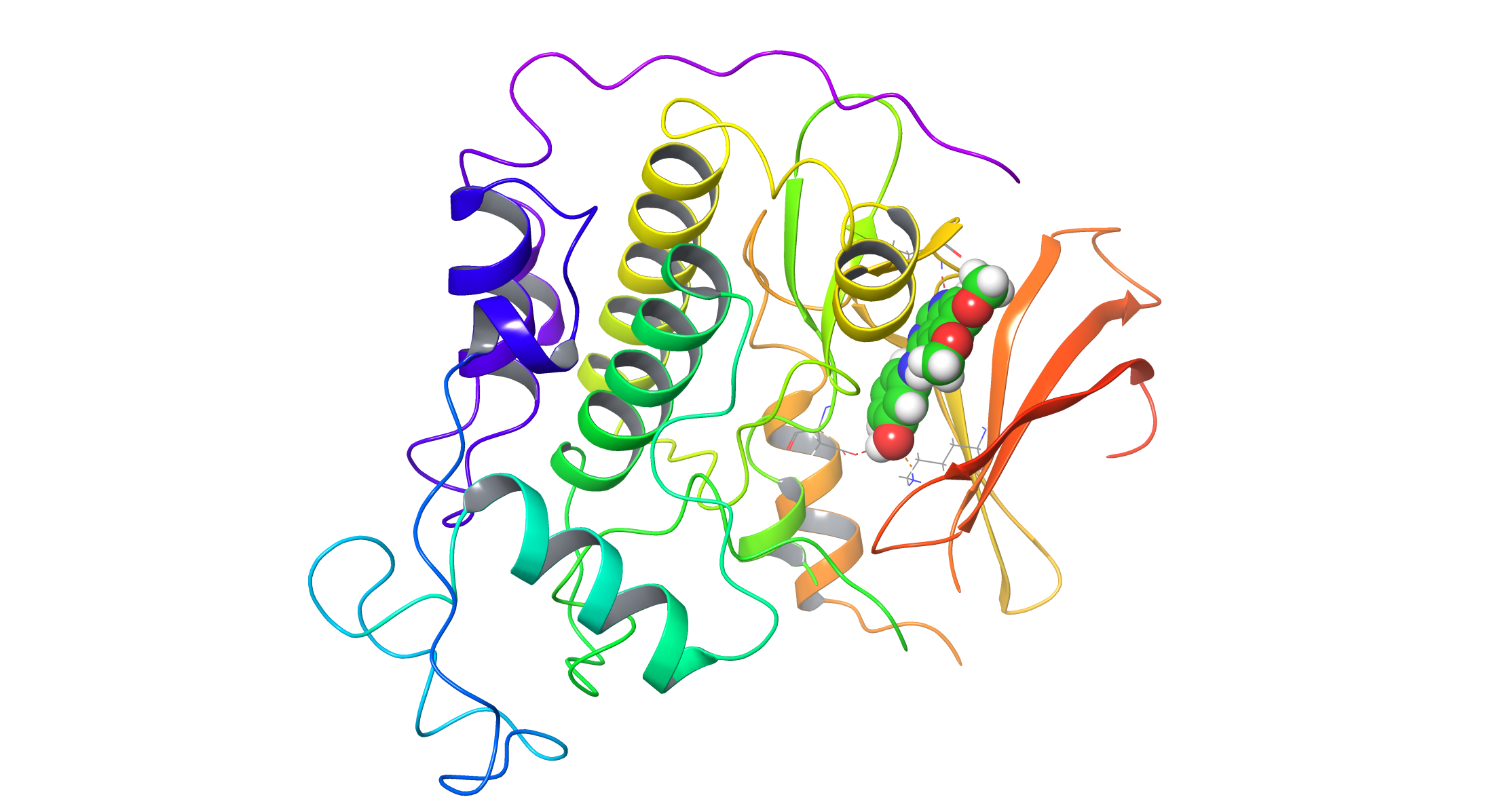


1. ***Plasmodium falciparum* lactate dehydrogenase (PFLDH) and co-crystallised ligand. An antimalarial target.**


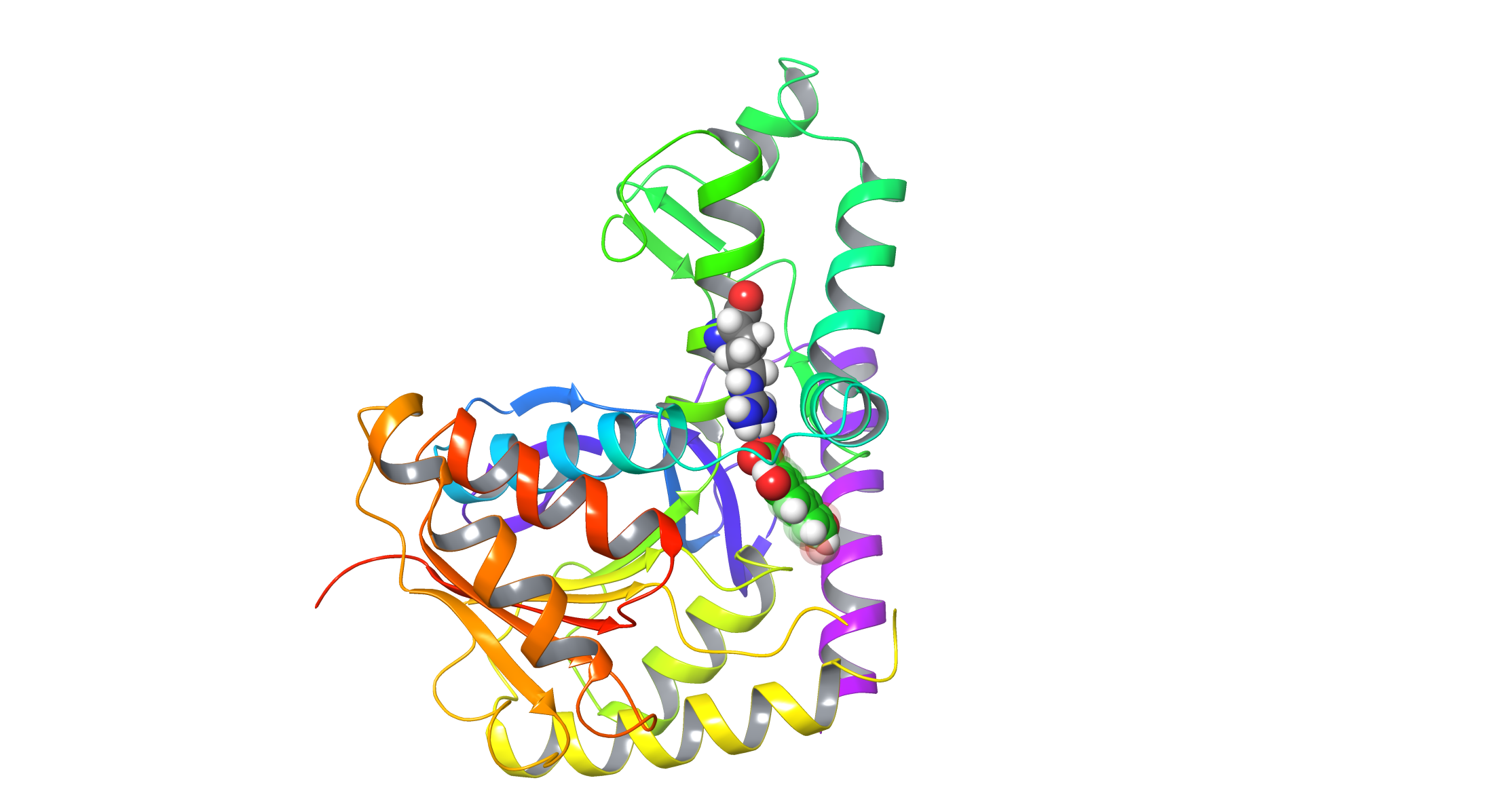


1. ***Plasmodium falciparum* malate dehydrogenase (PfMDH) and co-crystallised ligands. An antimalarial target.**


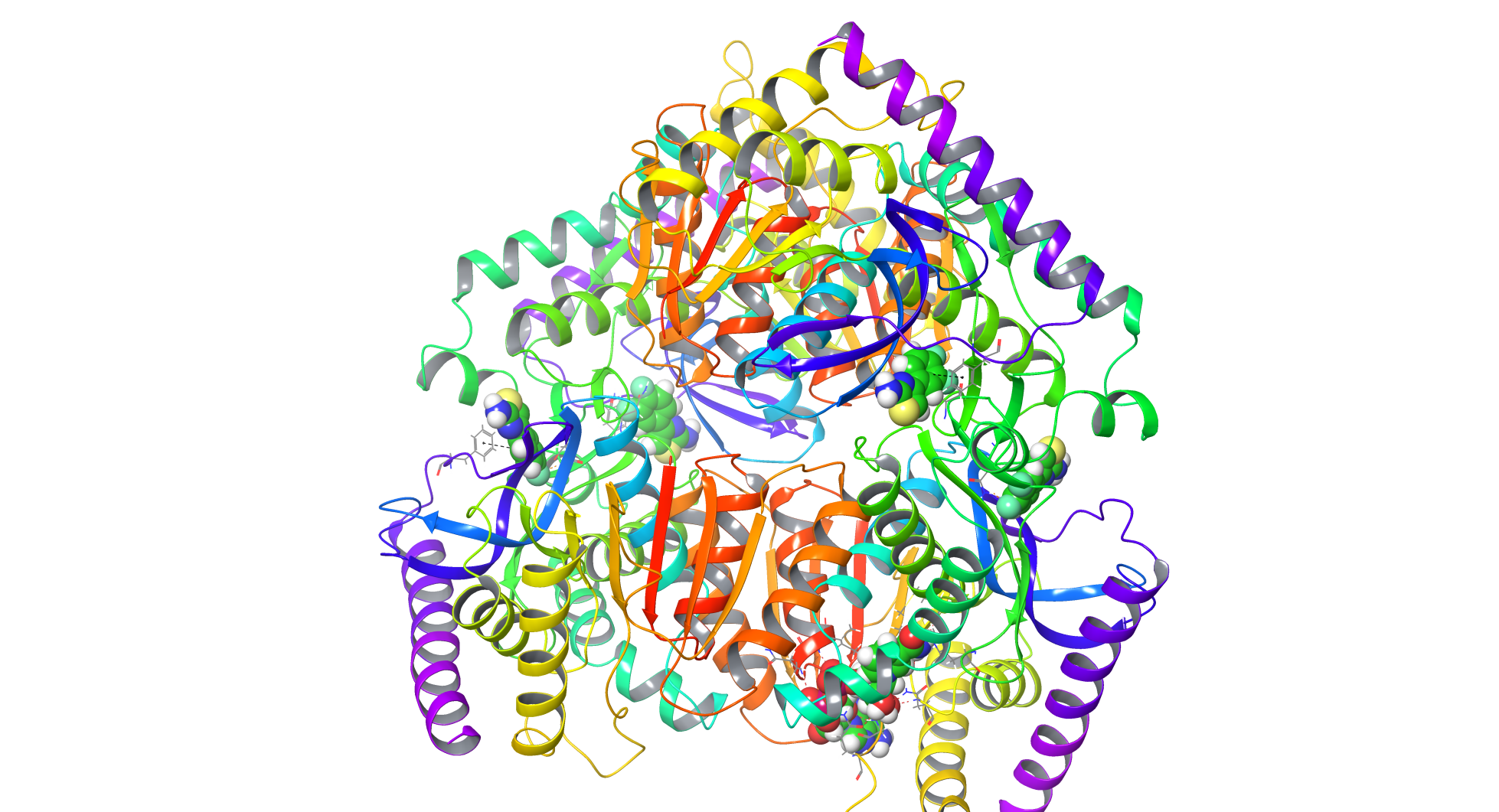


1. ***Plasmodium falciparum* phosphoethanolamine-methyltransferase (PfPMT) and co-crystallised ligand.** **An antimalarial target.**


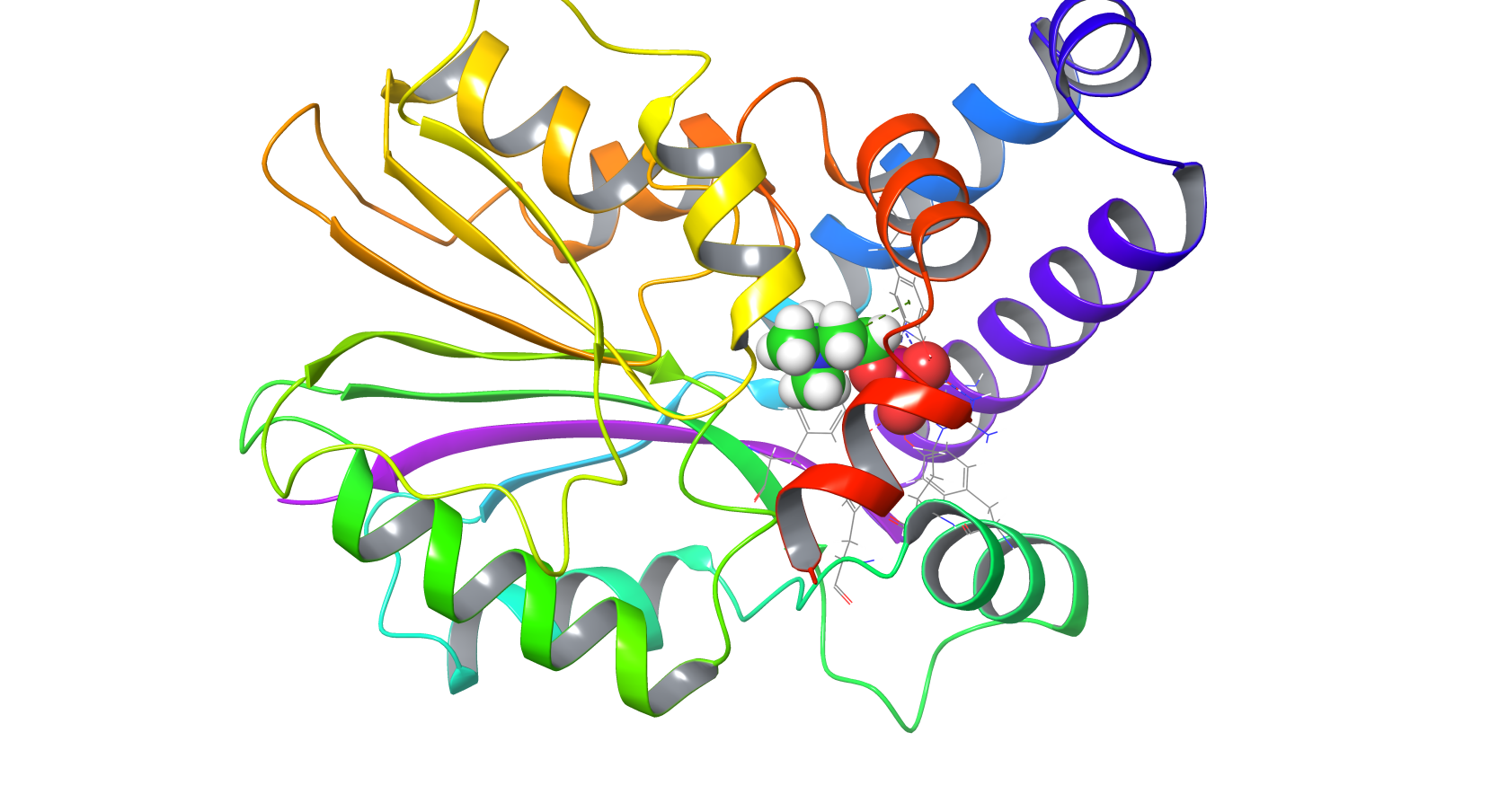

Supplement: Supplementary file 11 — (DOCX 7.30 MB) [file 894_2025_6299_MOESM11_ESM.docx]
