# Supplementary material for: Computable properties of selected monomeric acylphloroglucinols with anticancer and/or antimalarial activities and first-approximation docking study
Source: J Mol Model. 2025 Mar 12;31(4):113. doi: 10.1007/s00894-025-06299-7 (PMC11903629; doi:10.1007/s00894-025-06299-7)
Supplement: Supplementary file 12 — (DOCX 3.58 MB) [file 894_2025_6299_MOESM12_ESM.docx]

**Figure S12**

**Graphical representation of the main interactions in the molecule-target complexes, with the targets being biomolecules associated with various forms of cancer.**

All the images are obtained from docking simulation in GLIDE.

In each figure, the image on the left shows the docking pose of the protein-ligand complex and the image on the right shows the interactions between the ligand and the active site of the protein, highlighting the residues within this site. Dashed coloured segments denote the hydrogen bonds (“aromatic hydrogen bond” stands for O−H⋅⋅⋅π hydrogen bond); the meaning of the colours is specified under each figure. Short descriptions and the PDB IDs of the proteins are provided in Table 9.


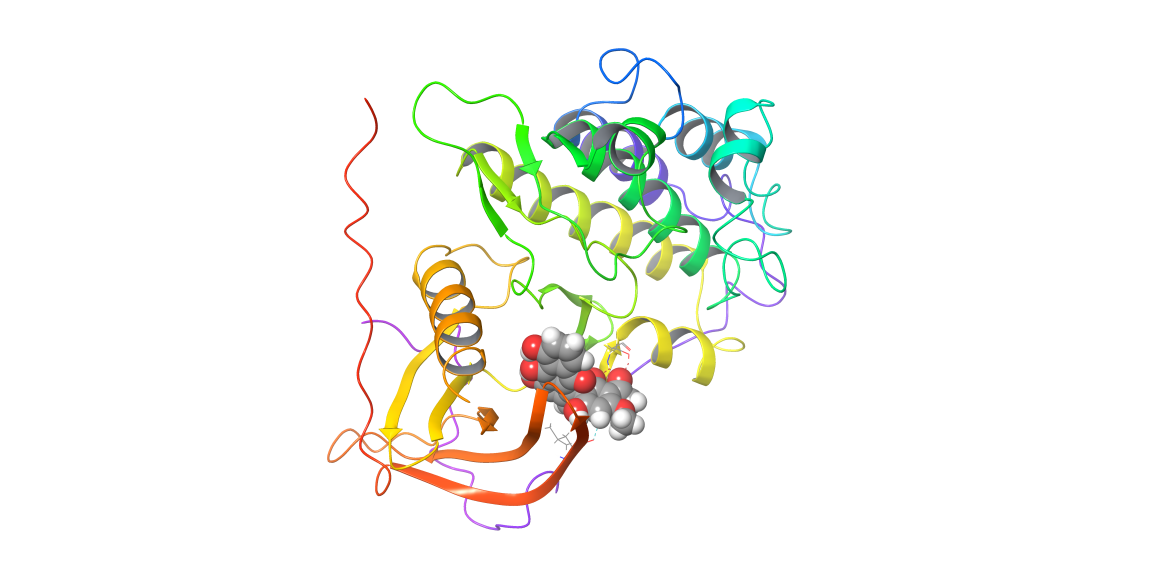

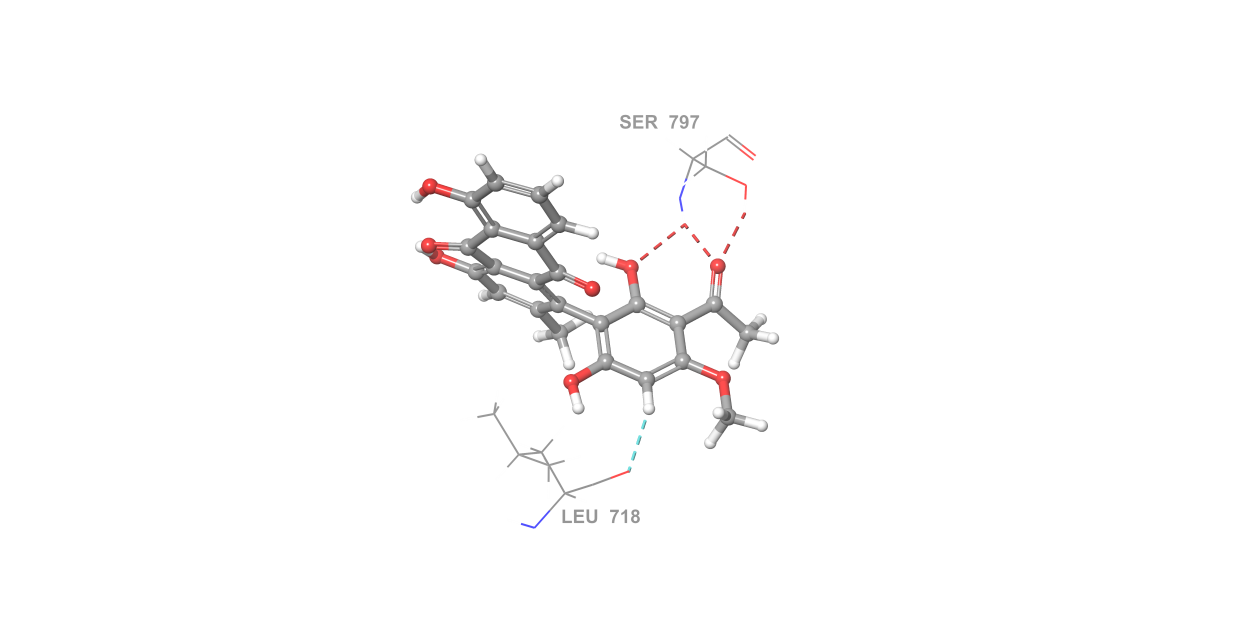


1. Graphical 3D representation of the main interactions in the EGFR-U4 complex, with EGFR (PDB: 6LUD) being a lung cancer target. In the image on the right, red-dashed segments denote hydrogen bonds, and light blue-dashed segments denote aromatic hydrogen bonds.


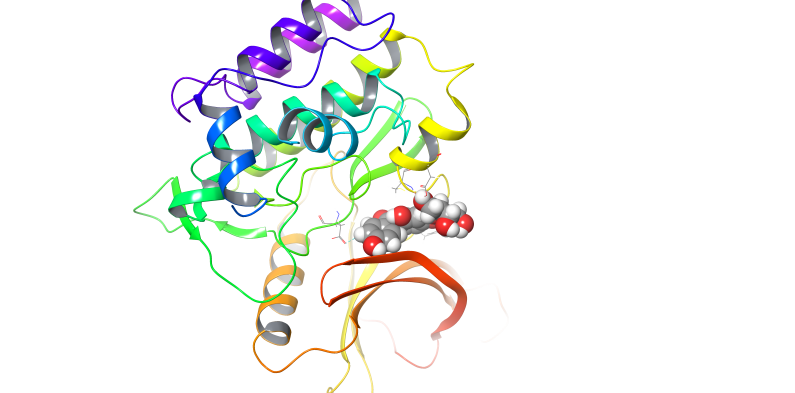

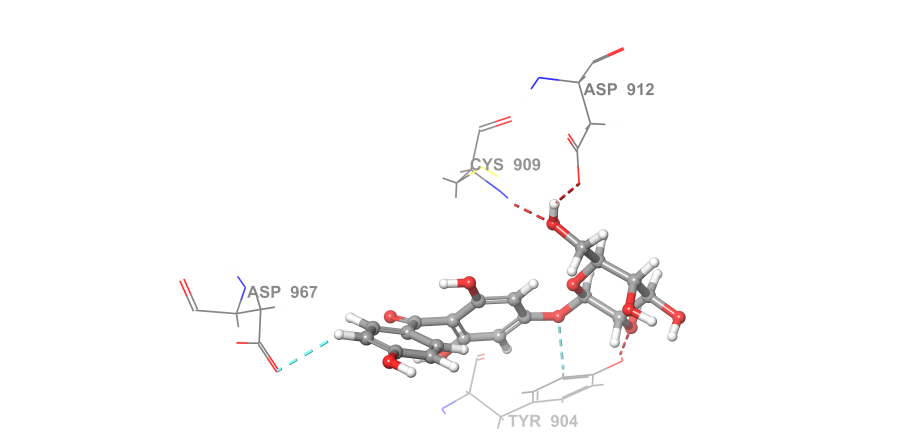


1. Graphical 3D representation of the main interactions in the JAK3-U8 complex, with JAK3 (PDB: 7C3N) being a lung cancer target. In the image on the right, red-dashed segments denote hydrogen bonds, and light blue-dashed segments denote aromatic hydrogen bonds.


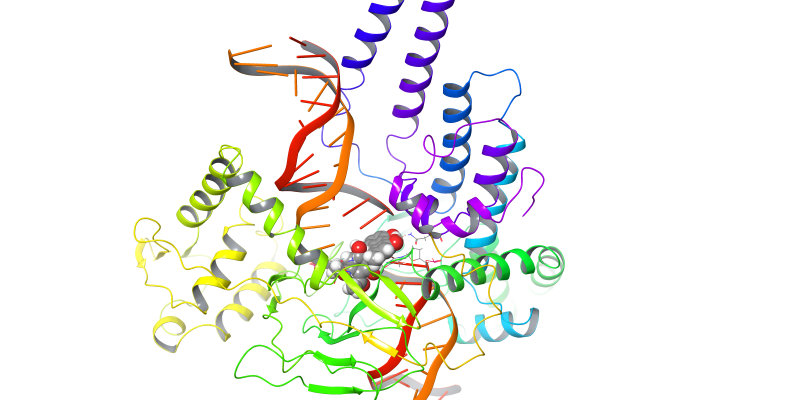

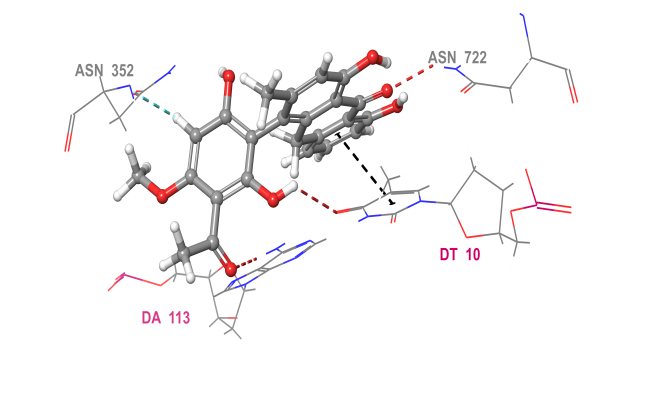


1. Graphical 3D representation of the main interactions in the Topo I-U5 complex, with Topo I (PDB: IT8I) being a lung cancer target. In the image on the right, red-dashed segments denote hydrogen bonds, light blue-dashed segments denote aromatic hydrogen bonds and black-dashed segments denote π-π stacking interactions.


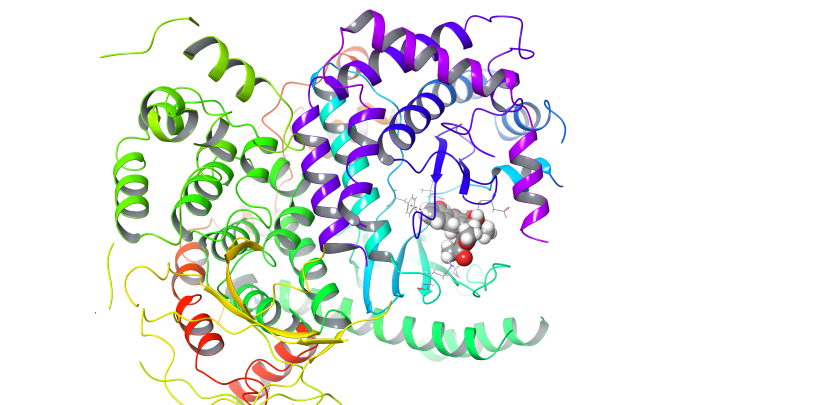

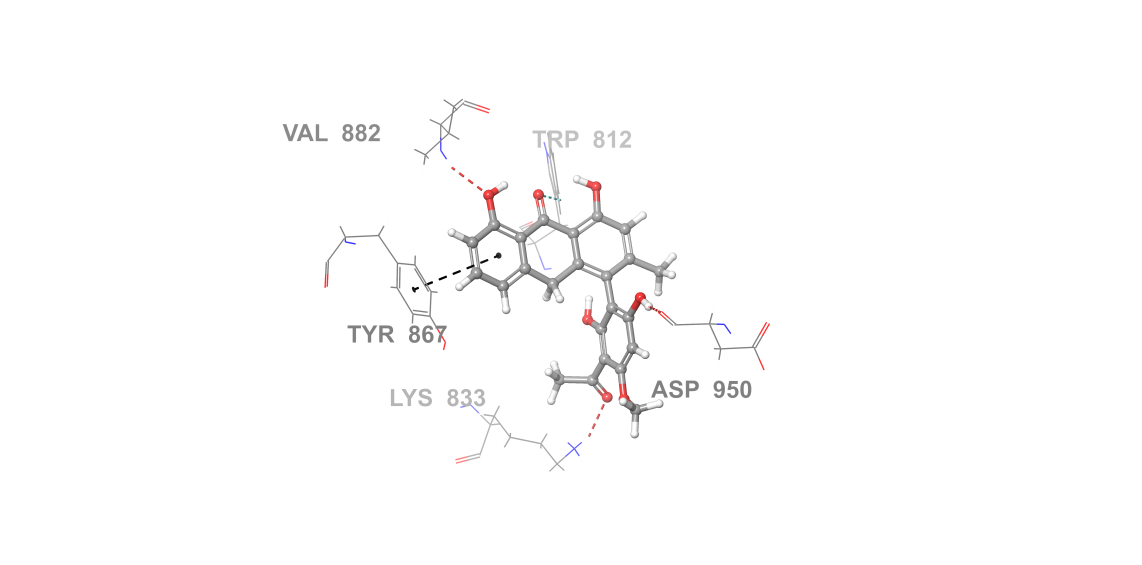


1. Graphical 3D representation of the main interactions in the P13K-U5 complex (within the binding site pocket B1), with P13K (PDB: 5JHB) being a lung cancer target. In the image on the right, red-dashed segments denote hydrogen bonds, light blue-dashed segments denote aromatic hydrogen bonds and black-dashed segments denote π-π stacking interactions.


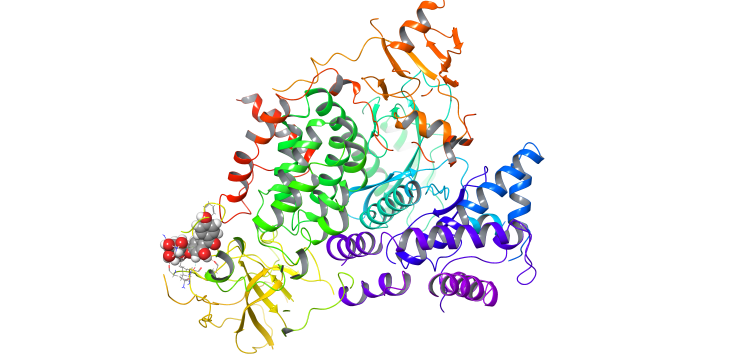

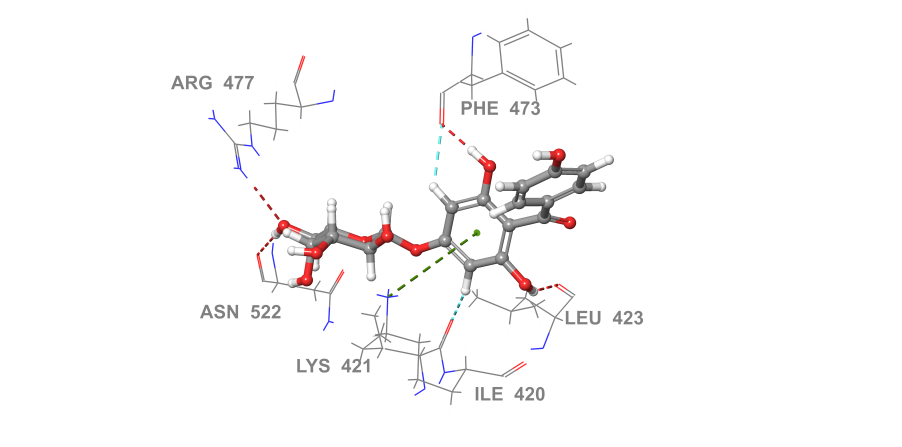


1. Graphical 3D representation of the main interactions in the P13K-U8 complex (within the binding site pocket B2), with P13K (PDB: 5JHB) being a lung cancer target. In the image on the right, red-dashed segments denote hydrogen bonds, light blue-dashed segments denote aromatic hydrogen bonds and green-dashed segments denote π-cation interactions.


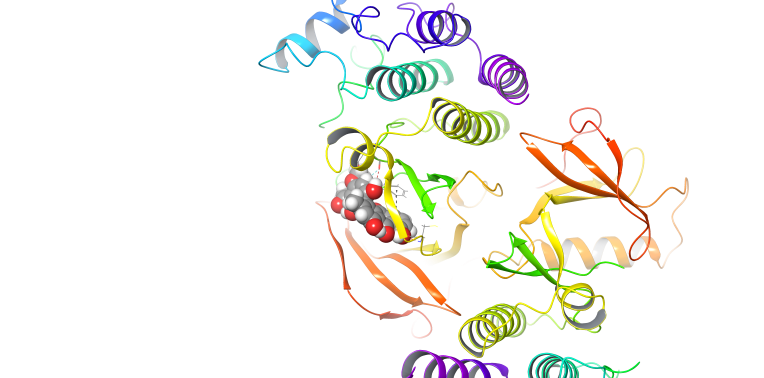

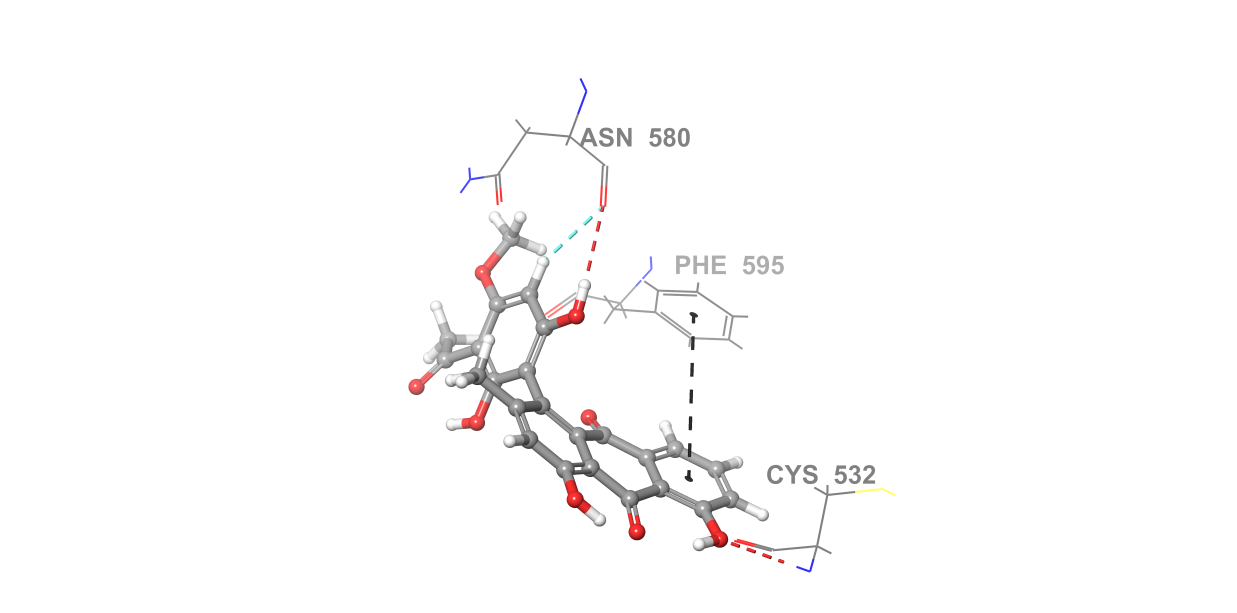


1. Graphical 3D representation of the main interactions in the BRAF V600b-U4 complex (within the binding site pocket C1), with BRAF V600b (PDB: 6V34) being a lung cancer target. In the image on the right, red-dashed segments denote hydrogen bonds, light blue-dashed segments denote aromatic hydrogen bonds and black-dashed segments denote π-π stacking interactions.


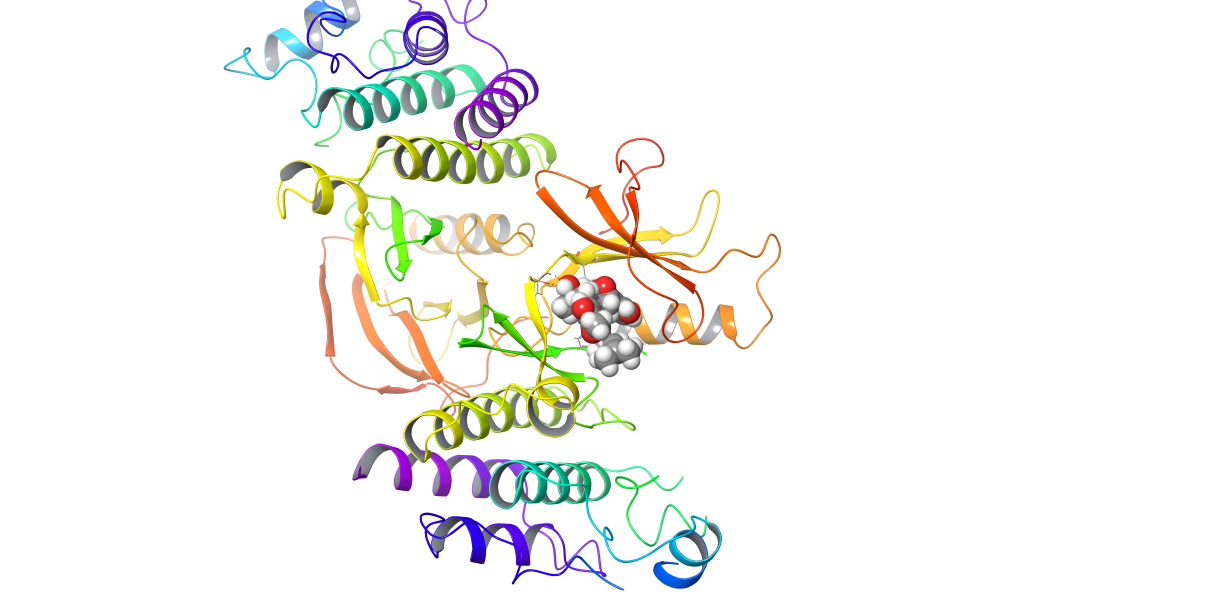

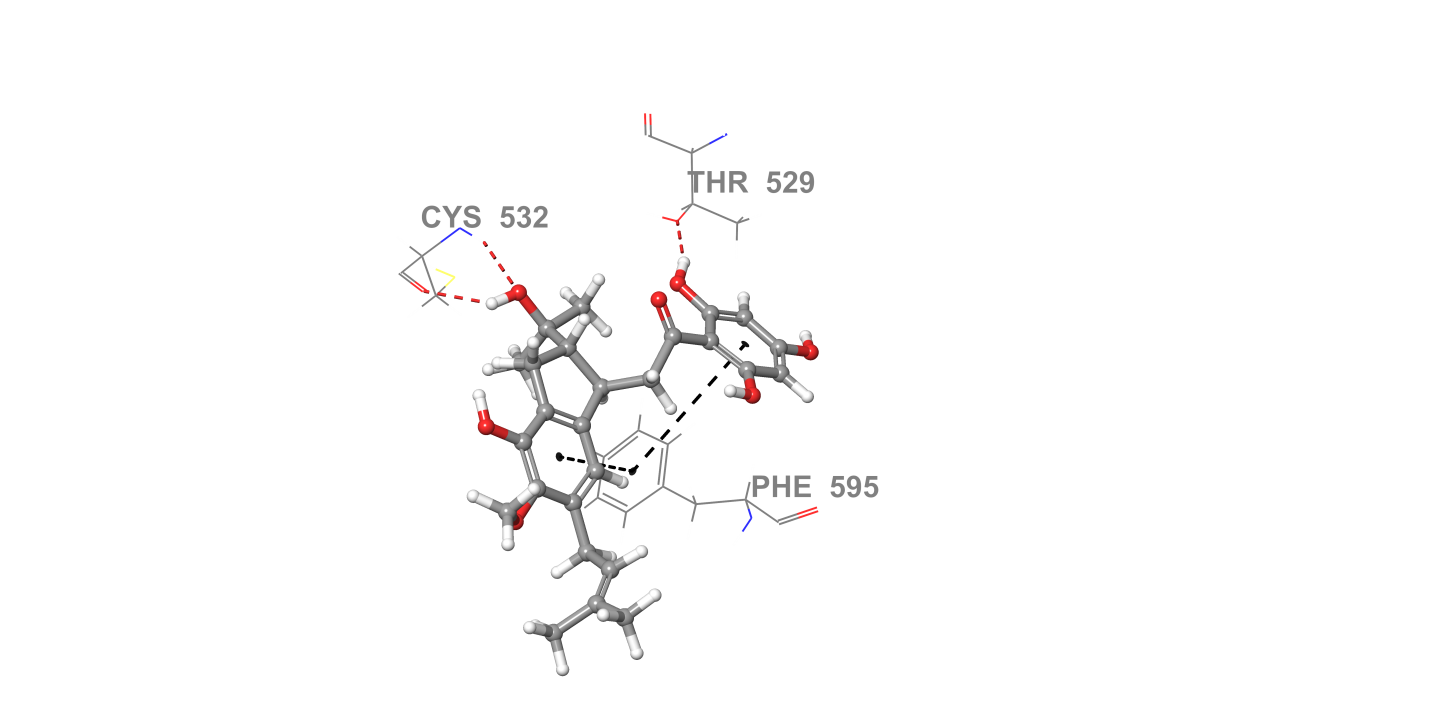


1. Graphical 3D representation of the main interactions in the BRAF V600b-U7 complex (within the binding site pocket C2), with BRAF V600b (PDB: 6V34) being a lung cancer target. In the image on the right, red-dashed segments denote hydrogen bonds and black-dashed segments denote π-π stacking interactions.


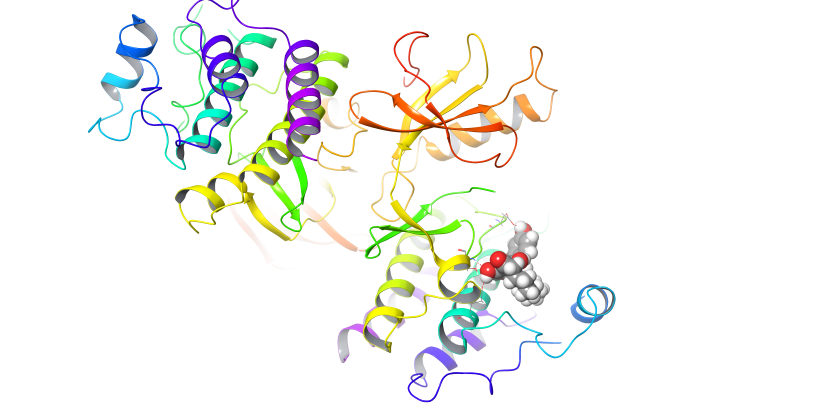

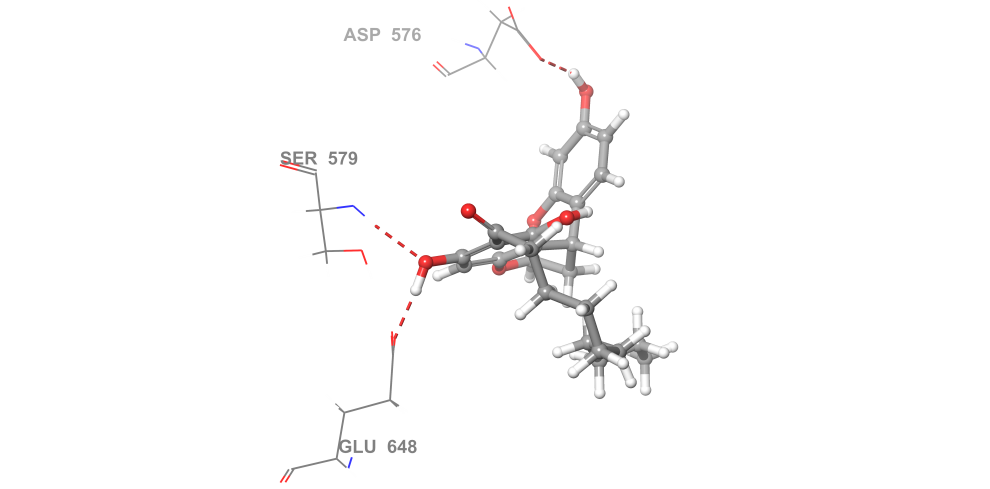


1. Graphical 3D representation of the main interactions in the BRAF V600b-U2 complex (within the binding site pocket C3), with BRAF V600b (PDB: 6V34) being a lung cancer target. In the image on the right, red-dashed segments denote hydrogen bonds.


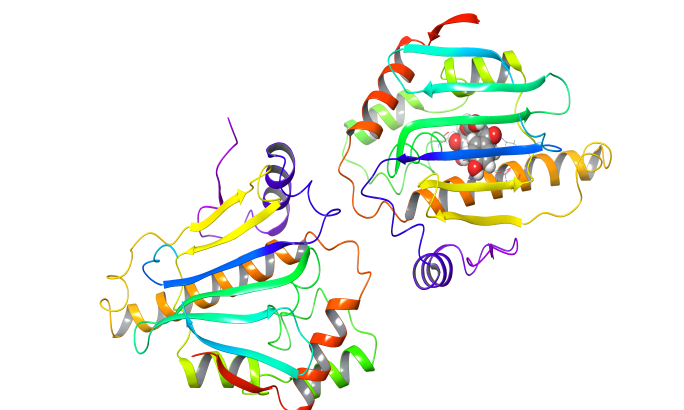

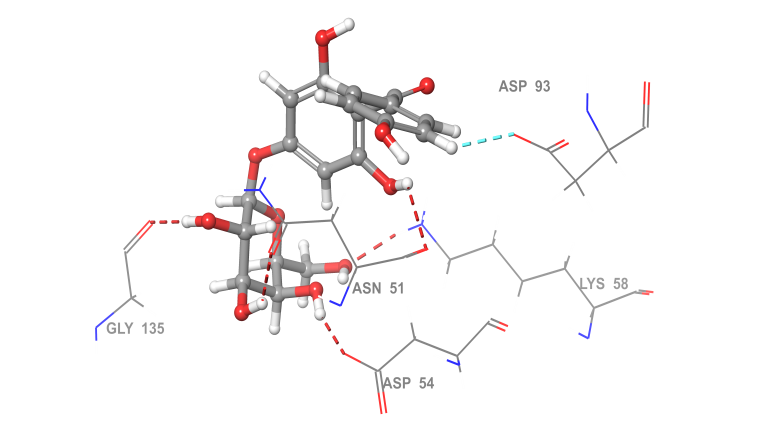


1. Graphical 3D representation of the main interactions in the H5P90-U8 complex (within the binding site pocket G1), with H5P90 (PDB: 3TUH) being a breast cancer target. In the image on the right, red-dashed segments denote hydrogen bonds and light blue-dashed segments denote aromatic hydrogen bonds.


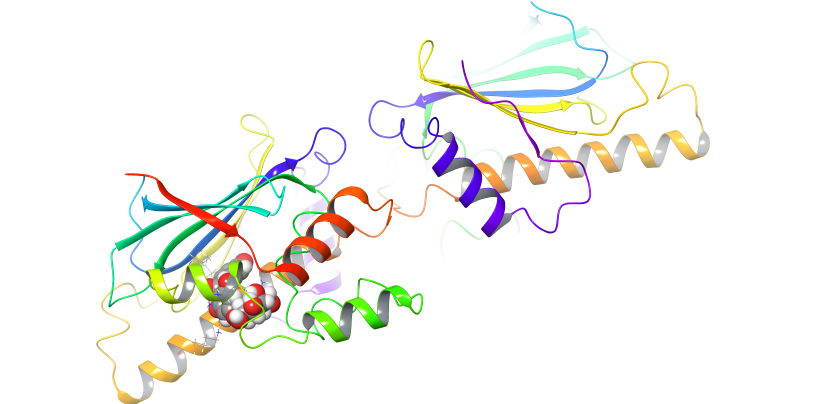

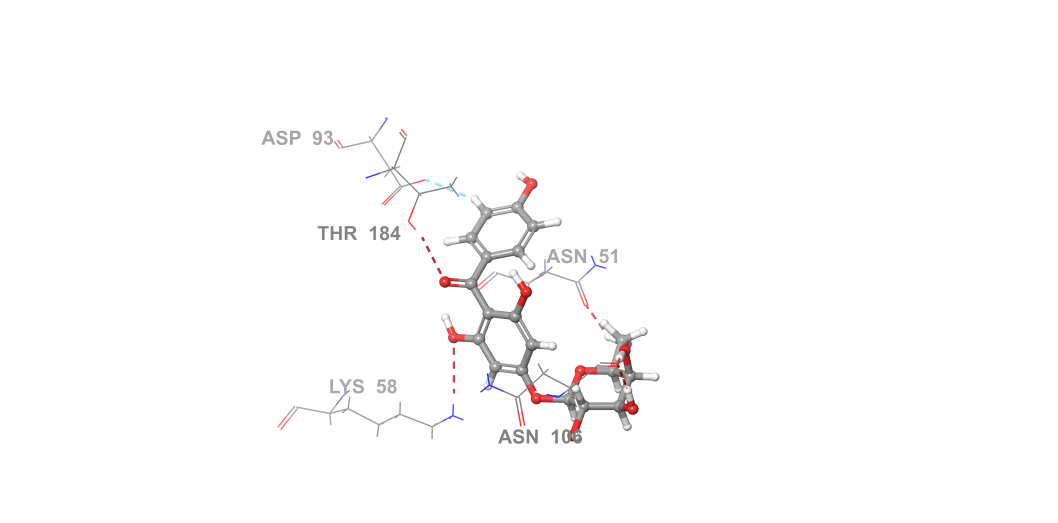


1. Graphical 3D representation of the main interactions in the H5P90-U8 complex (within the binding site pocket G2), with H5P90 (PDB: 3TUH) being a breast cancer target. In the image on the right, red-dashed segments denote hydrogen bonds and light blue-dashed segments denote aromatic hydrogen bonds.


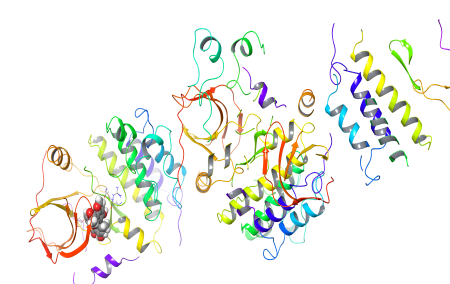

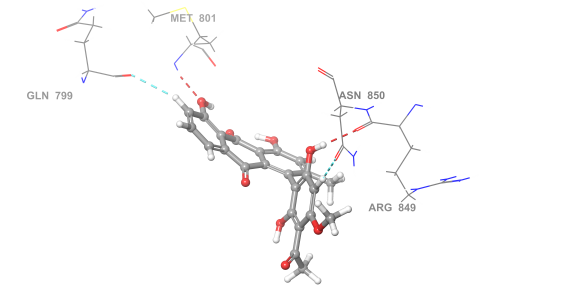


1. Graphical 3D representation of the main interactions in the HER2-U4 complex (within the binding site pocket H1), with HER2 (PDB: 3RCD) being a breast cancer target. In the image on the right, red-dashed segments denote hydrogen bonds and light blue-dashed segments denote aromatic hydrogen bonds.


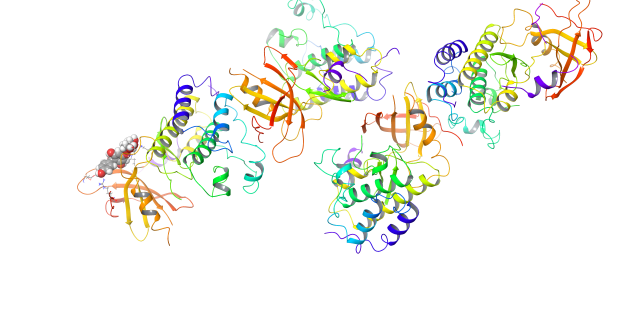

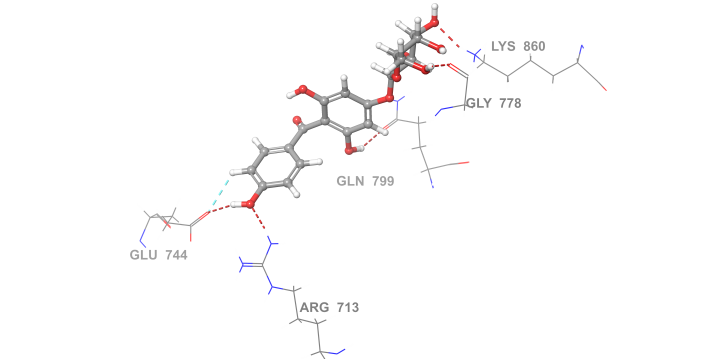


1. Graphical 3D representation of the main interactions in the HER2-U8 complex (within the binding site pocket H2), with HER2 (PDB: 3RCD) being a breast cancer target. In the image on the right, red-dashed segments denote hydrogen bonds and light blue-dashed segments denote aromatic hydrogen bonds.


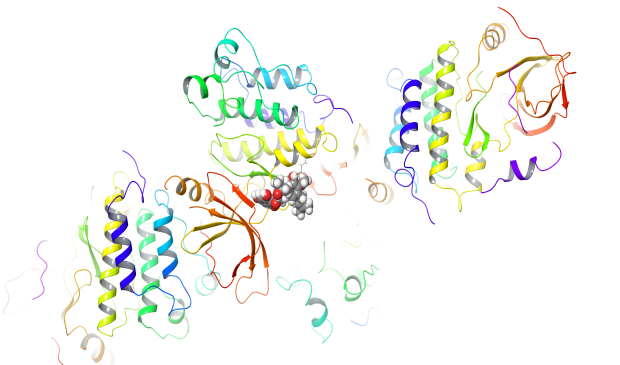

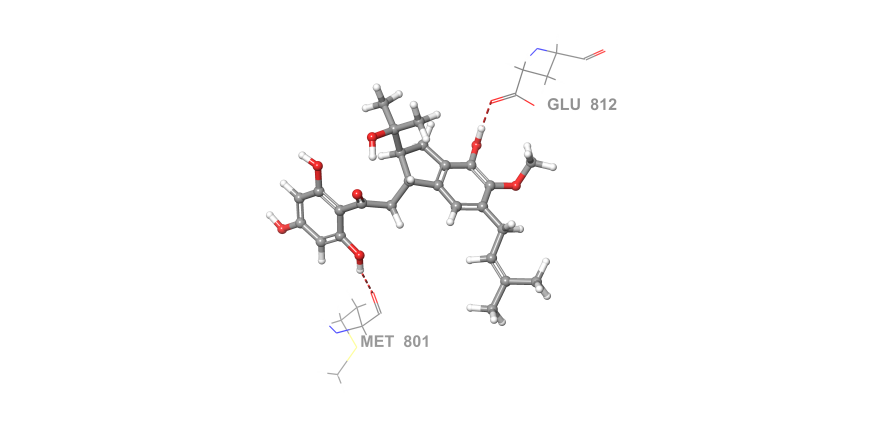


1. Graphical 3D representation of the main interactions in the HER2-U7 complex (within the binding site pocket H3), with HER2 (PDB: 3RCD) being a breast cancer target. In the image on the right, red-dashed segments denote hydrogen bonds.


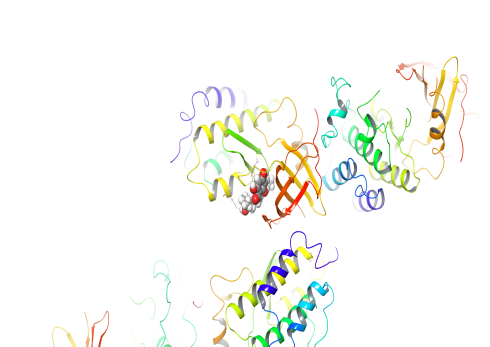

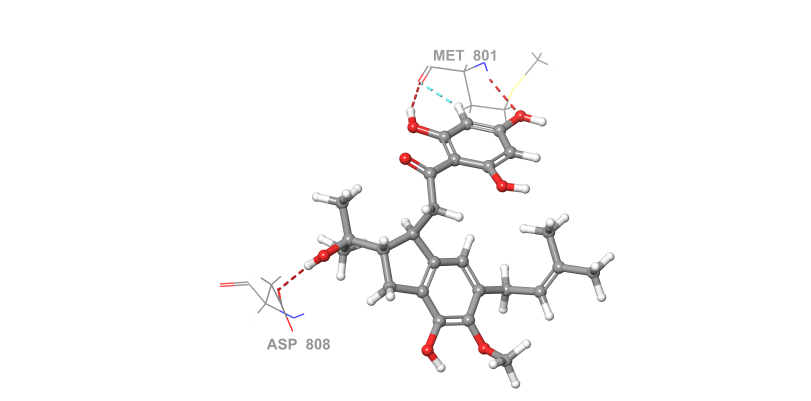


1. Graphical 3D representation of the main interactions in the HER2-U7 complex (within the binding site pocket H4), with HER2 (PDB: 3RCD) being a breast cancer target. In the image on the right, red-dashed segments denote hydrogen bonds and light blue-dashed segments denote aromatic hydrogen bonds.


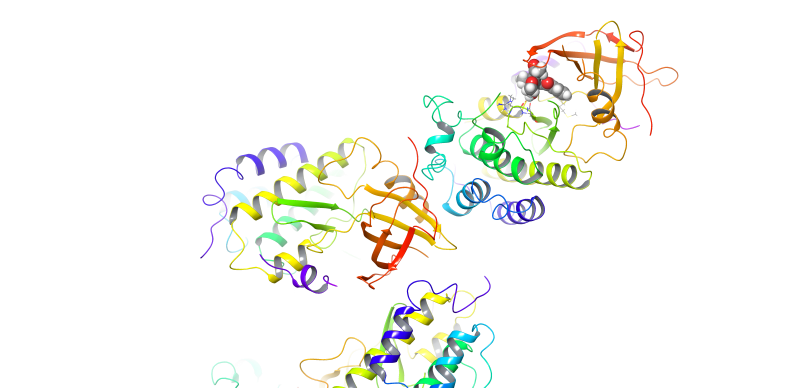

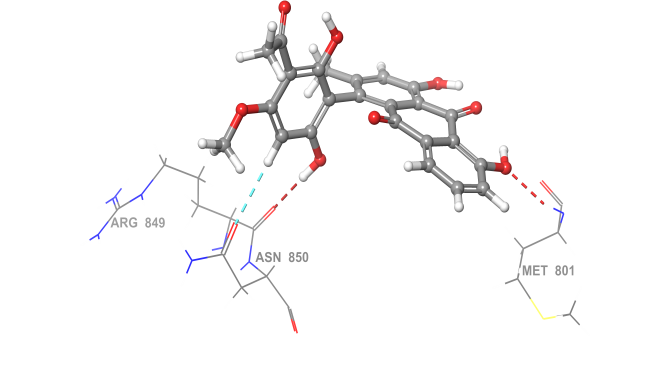


1. Graphical 3D representation of the main interactions in the HER2-U4 complex (within the binding site pocket H5), with HER2 (PDB: 3RCD) being a breast cancer target. In the image on the right, red-dashed segments denote hydrogen bonds and light blue-dashed segments denote aromatic hydrogen bonds.


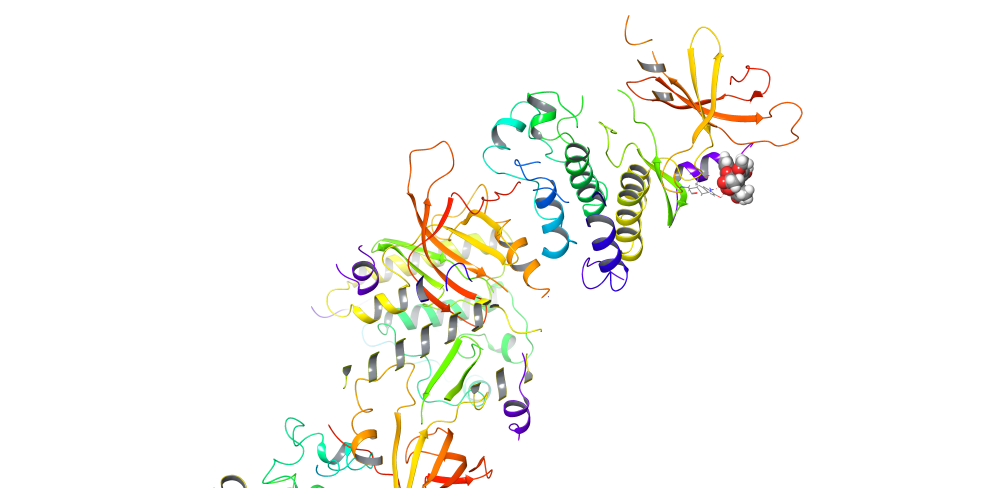

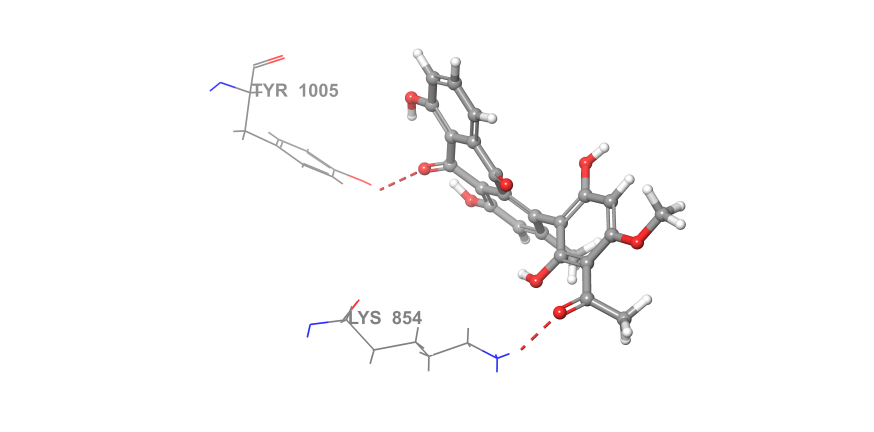


1. Graphical 3D representation of the main interactions in the HER2-U4 complex (within the binding site pocket H6), with HER2 (PDB: 3RCD) being a breast cancer target. In the image on the right, red-dashed segments denote hydrogen bonds.


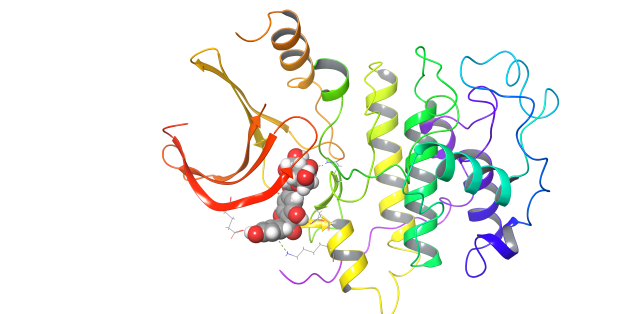

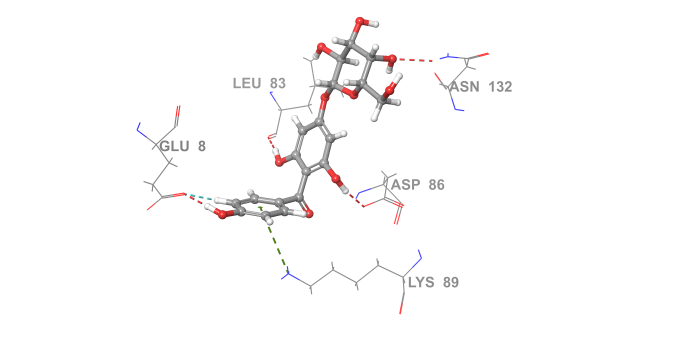


1. Graphical 3D representation of the main interactions in the CDK-2-U8 complex, with CDK-2 (PDB: 1DI8) being a lung cancer target. In the image on the right, red-dashed segments denote hydrogen bonds, light blue-dashed segments denote aromatic hydrogen bonds and green-dashed segments denote π-cation interactions.
