# Supplementary material for: Computable properties of selected monomeric acylphloroglucinols with anticancer and/or antimalarial activities and first-approximation docking study
Source: J Mol Model. 2025 Mar 12;31(4):113. doi: 10.1007/s00894-025-06299-7 (PMC11903629; doi:10.1007/s00894-025-06299-7)
Supplement: Supplementary file 13 — (DOCX 2.17 MB) [file 894_2025_6299_MOESM13_ESM.docx]

**Figure S13**

**Graphical representation of the main interactions in the molecule-target complexes, with the targets being proteins associated with malaria.**

All the images are obtained from docking simulation in GLIDE.

In each figure, the image on the left shows the docking pose of the protein-ligand complex and the image on the right shows the interactions between the ligand and the active site of the protein, highlighting the residues within this site. Dashed coloured segments denote the hydrogen bonds; the meaning of the colours is specified under each figure. Short descriptions and the PDB IDs of the proteins are provided in Table 9.


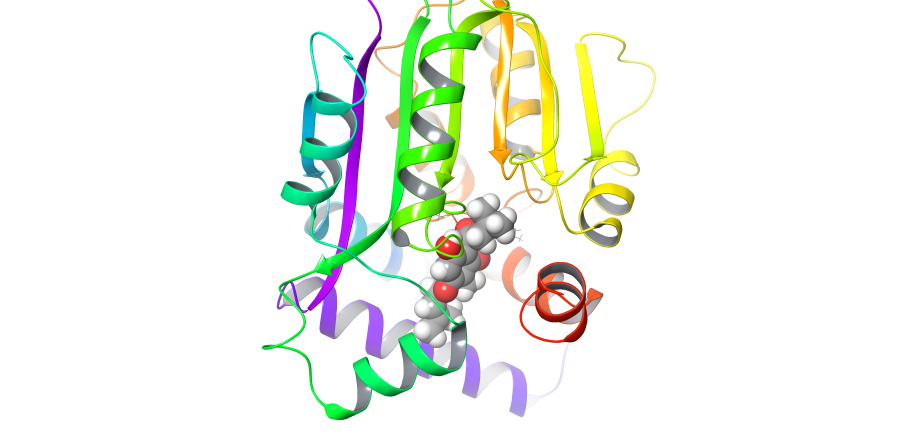

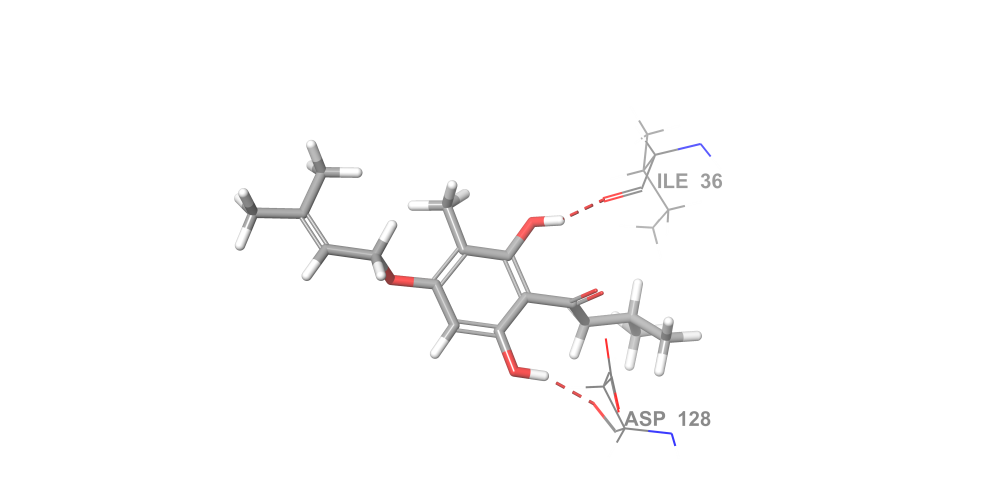


1. Graphical 3D representation of the main interactions in the PFPMT-U6 complex, with PFPMT (PDB: 3UJ9) being an antimalarial target. In the image on the right, red-dashed segments denote hydrogen bonds.


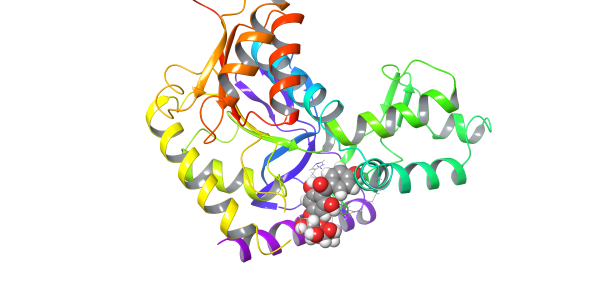

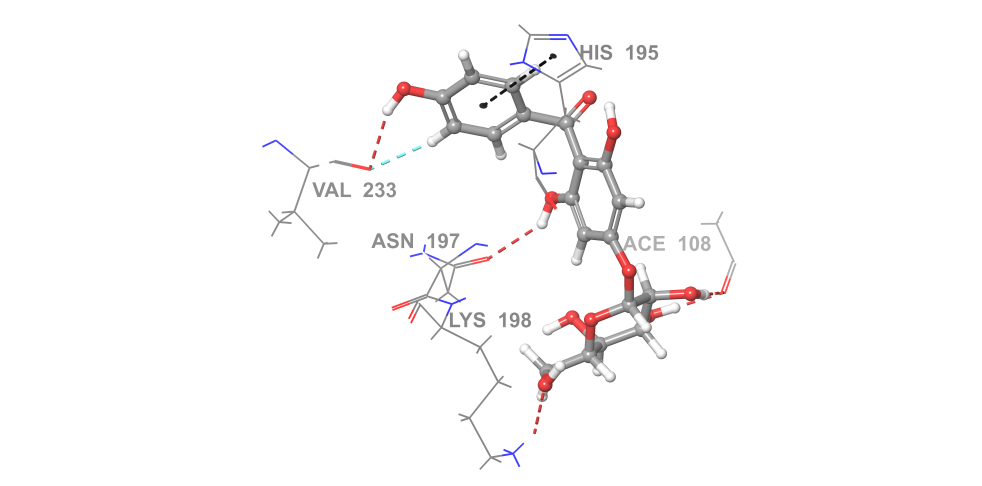


1. Graphical 3D representation of the main interactions in the PFLDH-U8 complex, with PFLDH (PDB: 1U5A) being an antimalarial target. In the image on the right, red-dashed segments denote hydrogen bonds, light blue-dashed segments denote aromatic hydrogen bonds and black-dashed segments denote π-π stacking interactions.


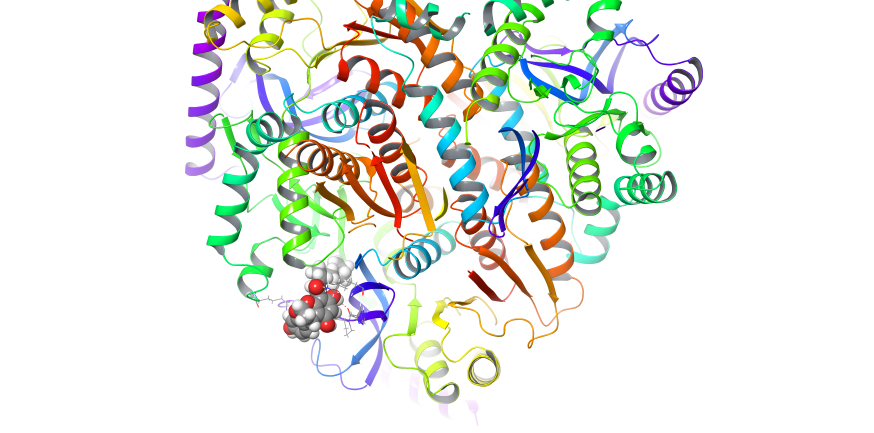

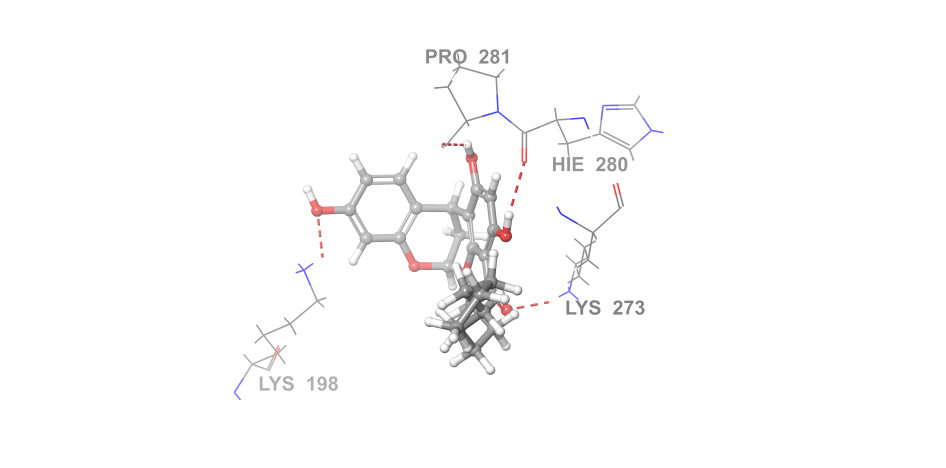


1. Graphical 3D representation of the main interactions in the PFMDH-U2 complex (within the binding site pocket M1), with PFMDH (PDB: 7C3N) being an antimalarial target. In the image on the right, red-dashed segments denote hydrogen bonds.


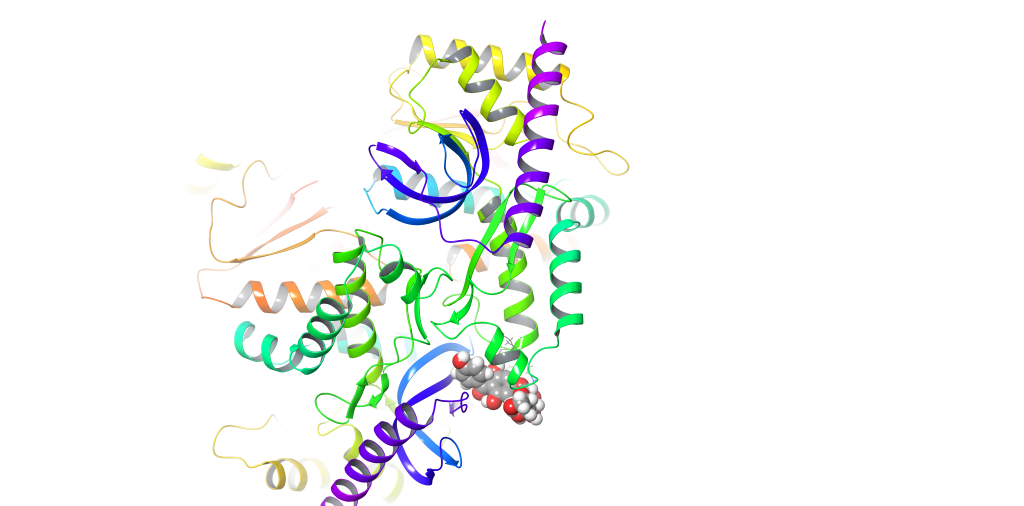

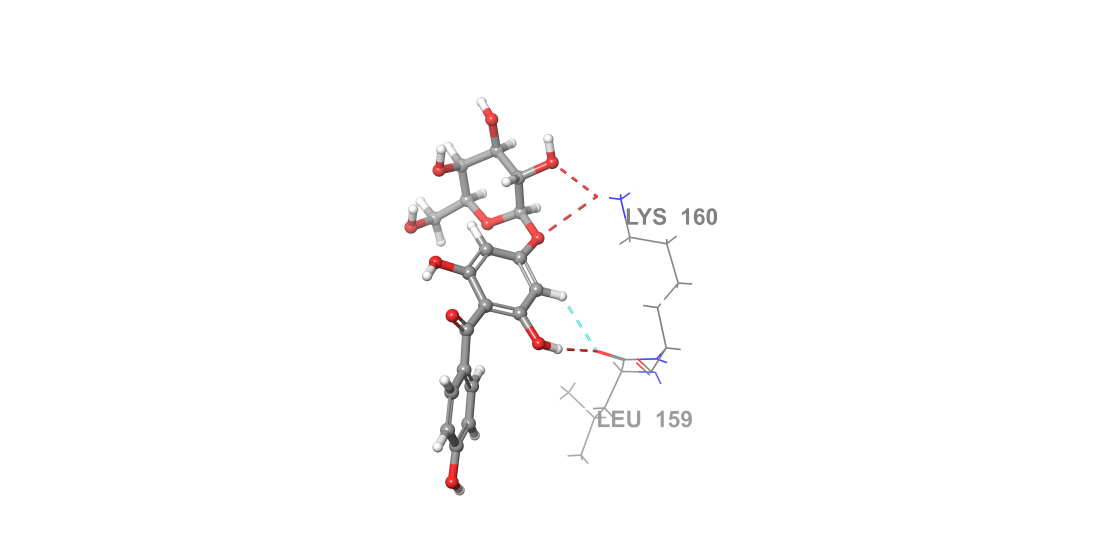


1. Graphical 3D representation of the main interactions in the PFMDH-U8 complex (within the binding site pocket M2), with PFMDH (PDB: 6R8G) being an antimalarial target. In the image on the right, red-dashed segments denote hydrogen bonds and light blue-dashed segments denote aromatic hydrogen bonds.


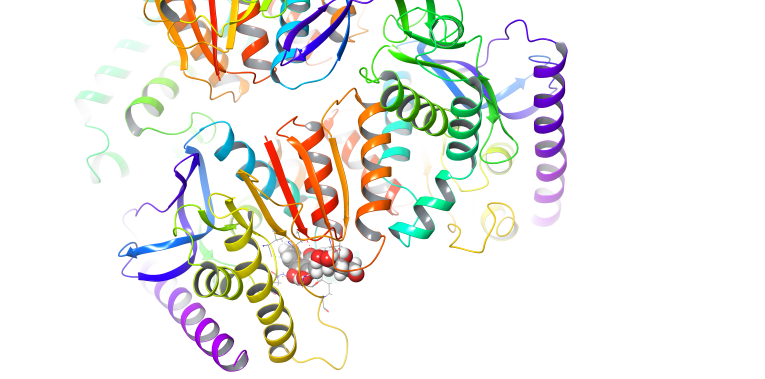

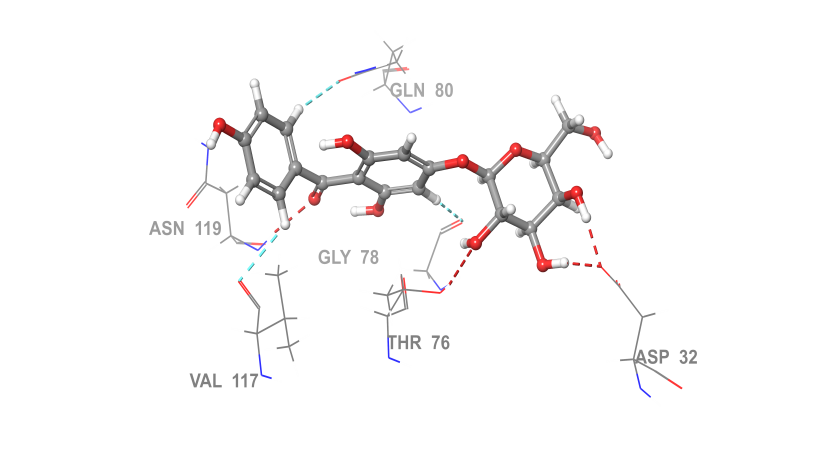


1. Graphical 3D representation of the main interactions in the PFMDH-U8 complex (within the binding site pocket M3), with PFMDH (PDB: 6R8G) being an antimalarial target. In the image on the right, red-dashed segments denote hydrogen bonds and light blue-dashed segments denote aromatic hydrogen bonds.


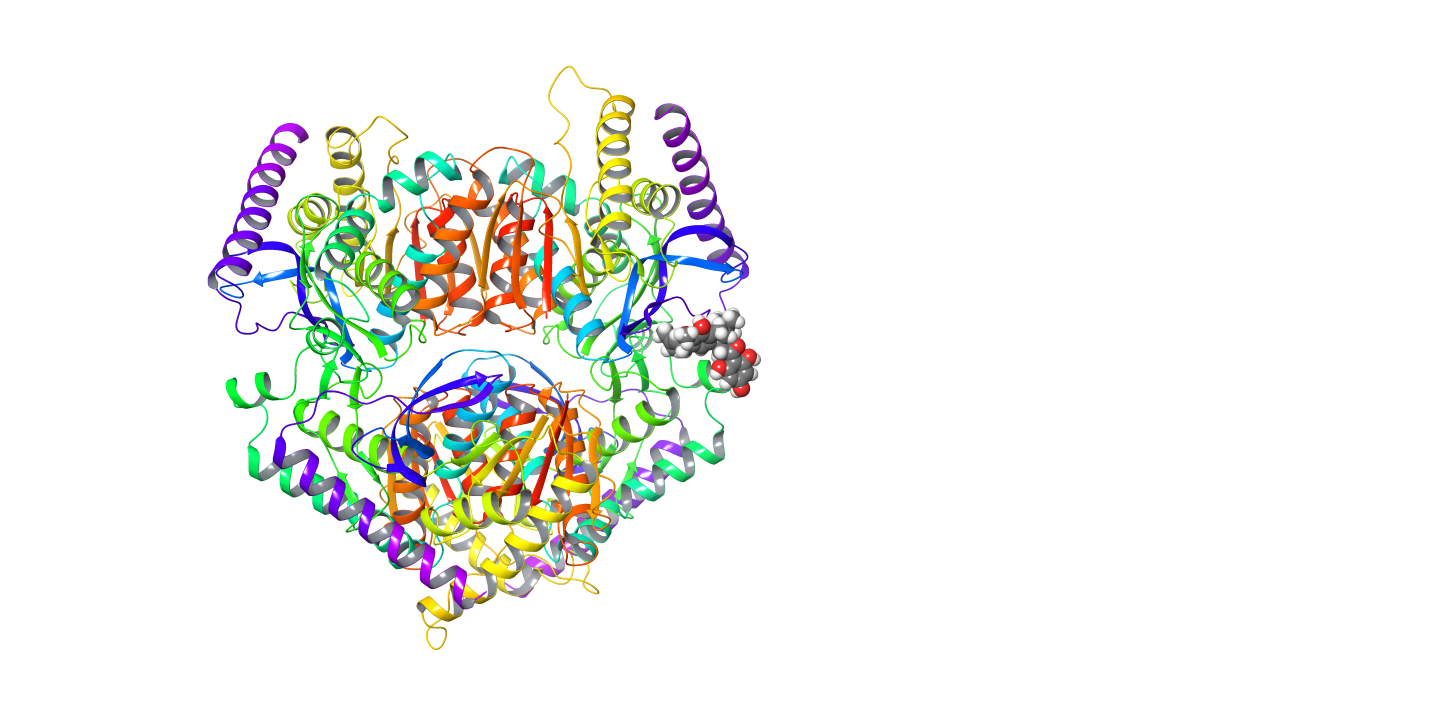

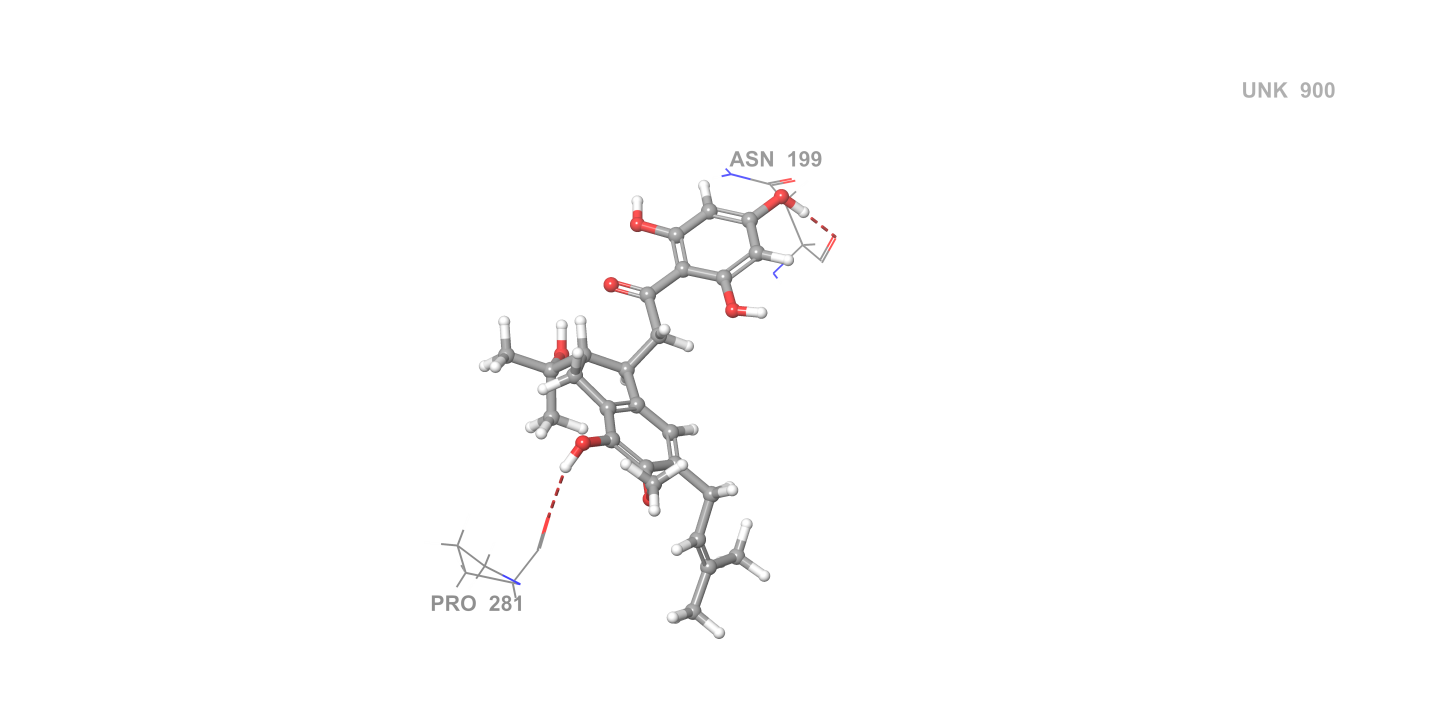


1. Graphical 3D representation of the main interactions in the PFMDH-U7 complex (within the binding site pocket M4), with PFMDH (PDB: 6R8G) being an antimalarial target. In the image on the right, red-dashed segments denote hydrogen bonds.


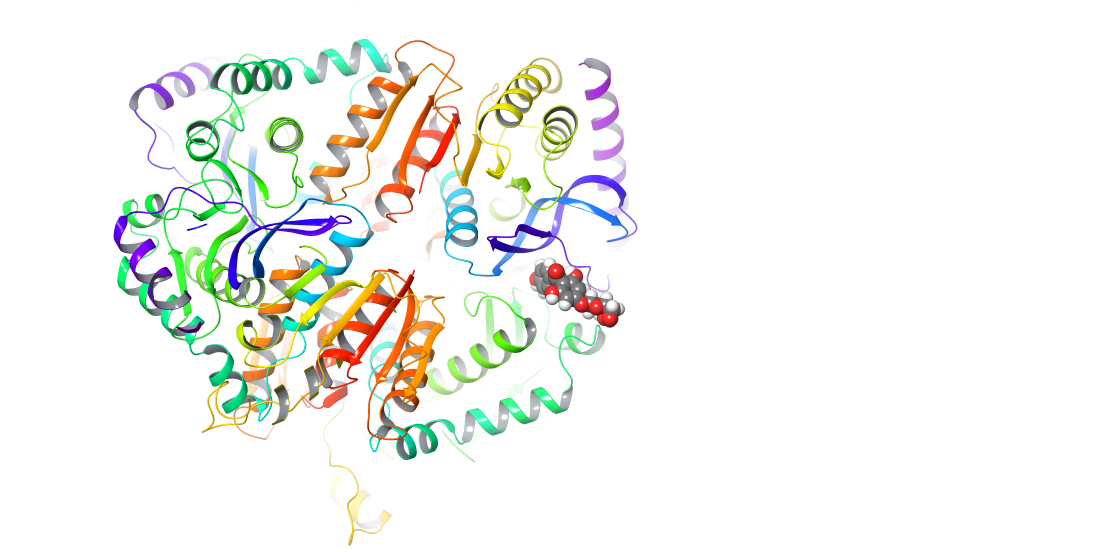

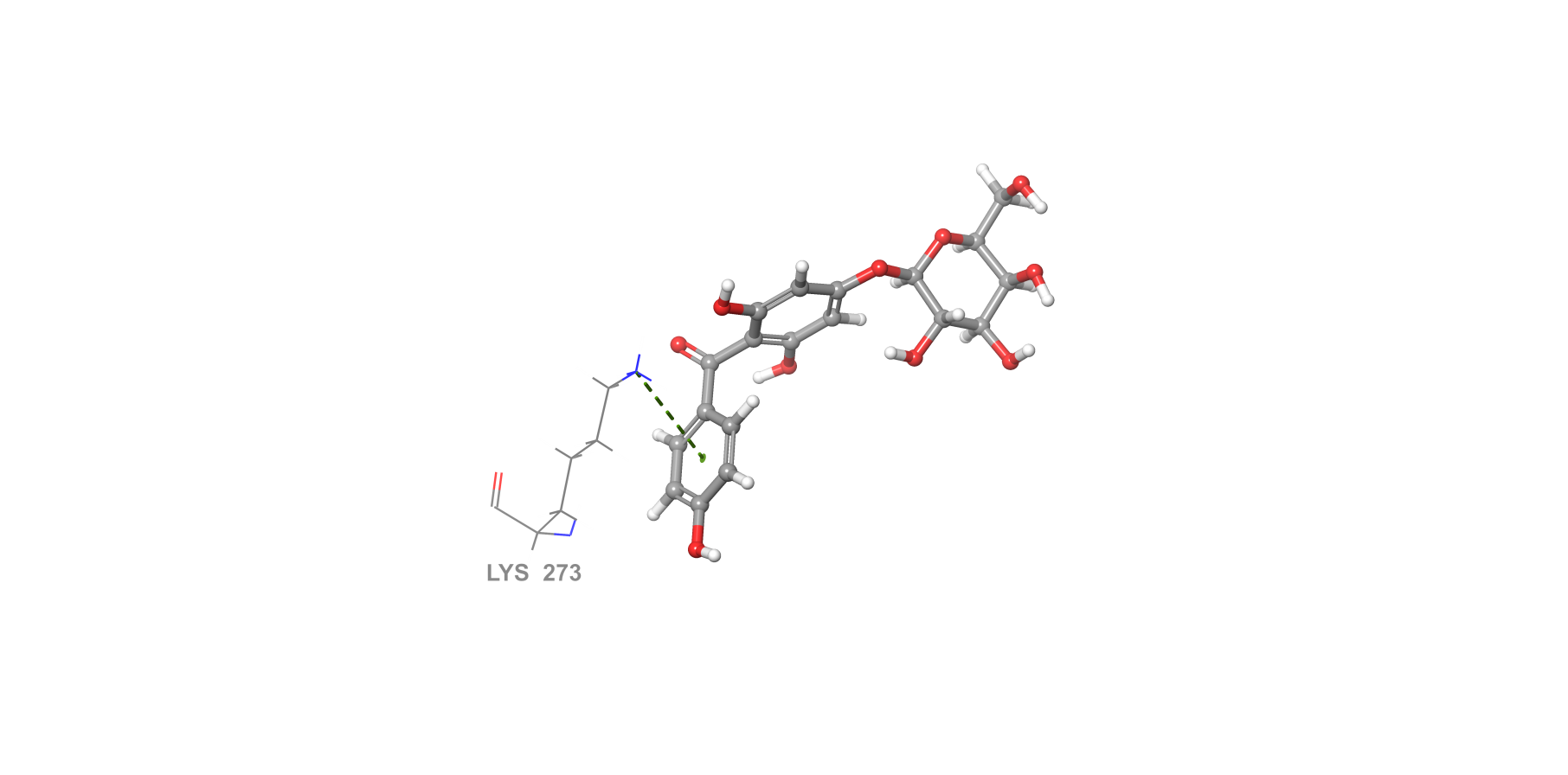


1. Graphical 3D representation of the main interactions in the PFMDH-U8 complex (within the binding site pocket M5), with PFMDH (PDB: 6R8G) being an antimalarial target. In the image on the right, red-dashed segments denote π-cation interactions.
