# Supplementary material for: Computable properties of selected monomeric acylphloroglucinols with anticancer and/or antimalarial activities and first-approximation docking study
Source: J Mol Model. 2025 Mar 12;31(4):113. doi: 10.1007/s00894-025-06299-7 (PMC11903629; doi:10.1007/s00894-025-06299-7)
Supplement: Supplementary file 14 — (DOCX 1.87 MB) [file 894_2025_6299_MOESM14_ESM.docx]

**Figure S14**

**Graphical comparison of the main interactions with an anticancer and an antimalarial target for molecules showing both activities**

All the images are obtained from docking simulation in GLIDE.

In each figure, the image denoted as ‘a’ shows the lowest energy conformer of the considered molecule *in vacuo*, the image denoted as ‘b’ shows the interactions between the ligand and the active site of the protein associated with cancer and the image denoted as ‘c’ shows the interactions between the ligand and the active site of the protein associated with malaria. The images highlight the residues within this site. The information under each figure specifies the nature of the protein and its active site used for the docking. Dashed coloured segments denote the hydrogen bonds; the meaning of the colours is specified under each figure. Short descriptions and the PDB IDs of the proteins are provided in Table 9.

**
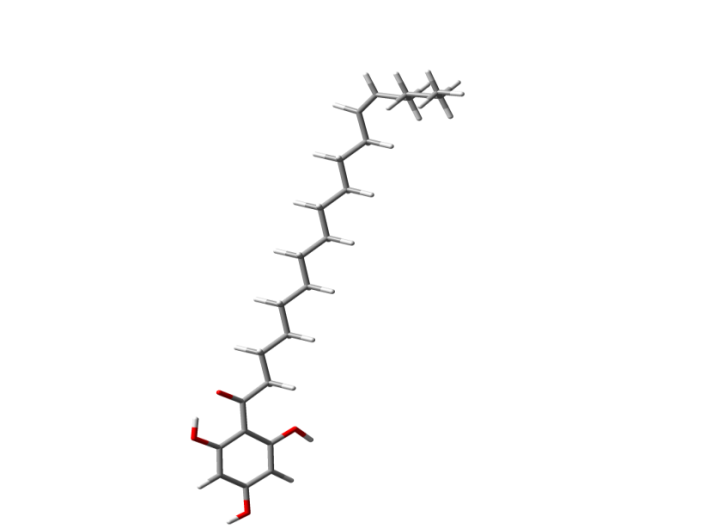
**
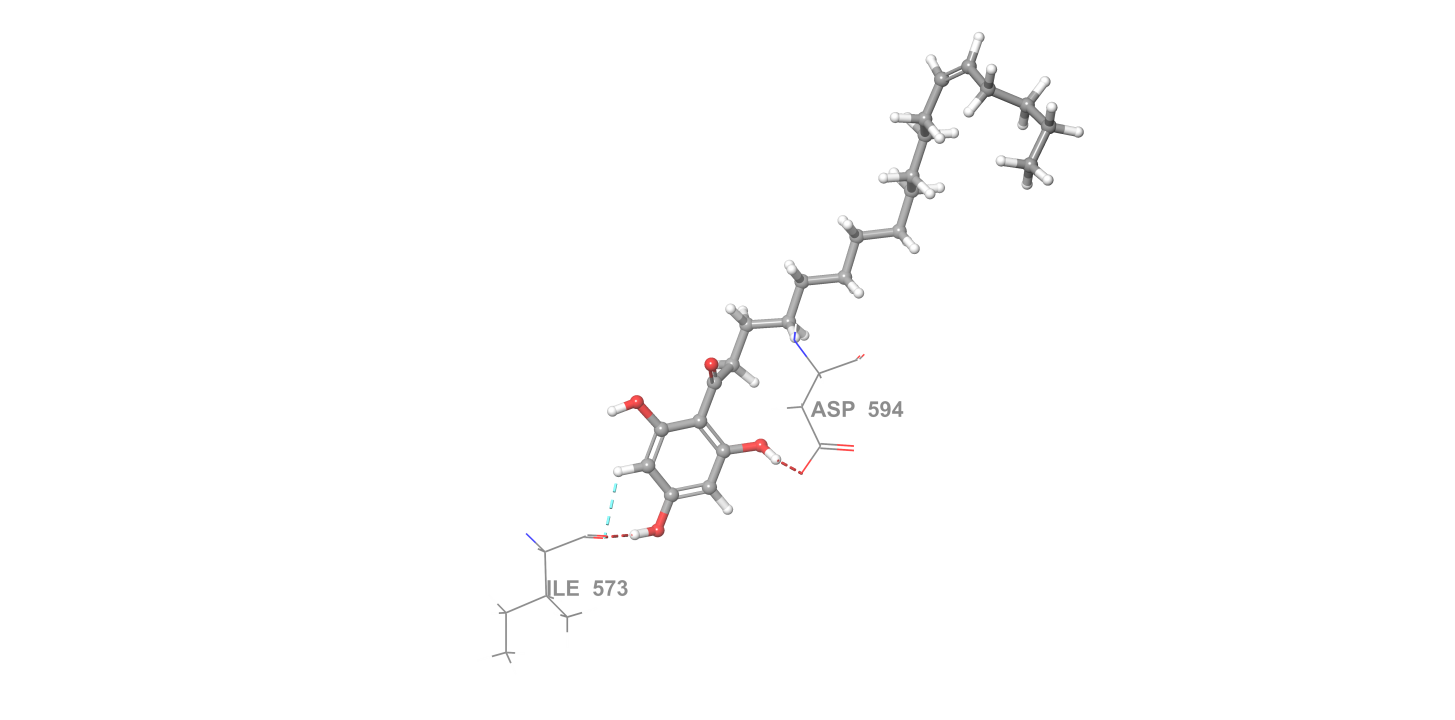

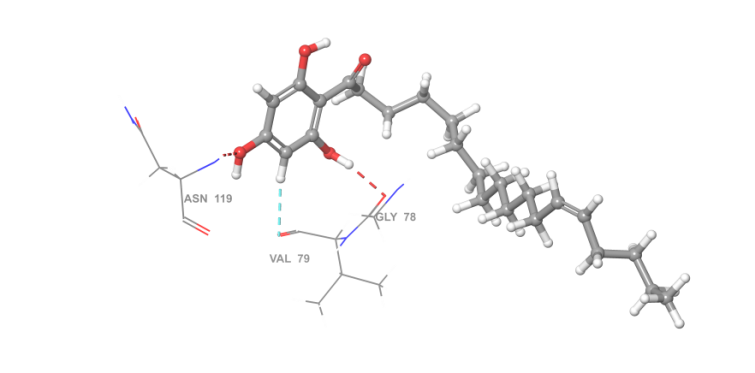


a b c

**a) Results for thouvenol A (U1).**

Interactions with the C2 active site of BRAF V600b (associated with cancer), and the M3 active site of PFMDH (associated with malaria).

**
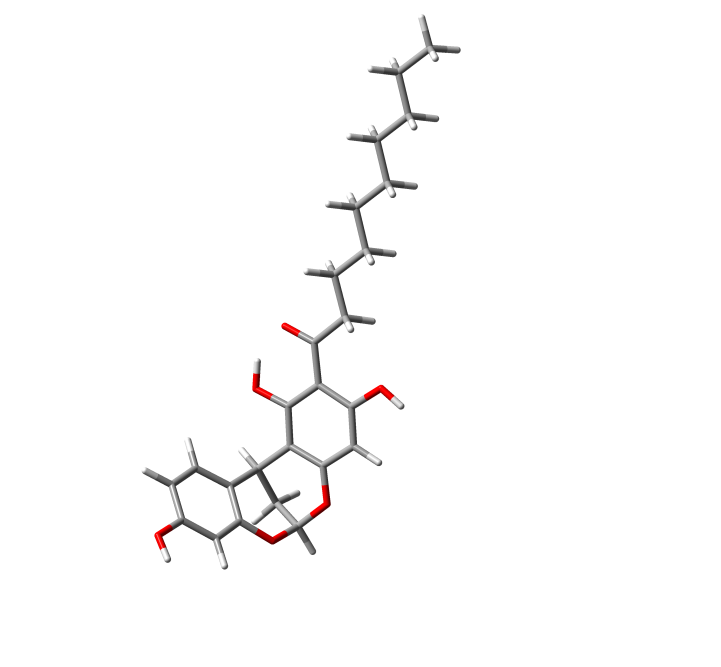
**

a


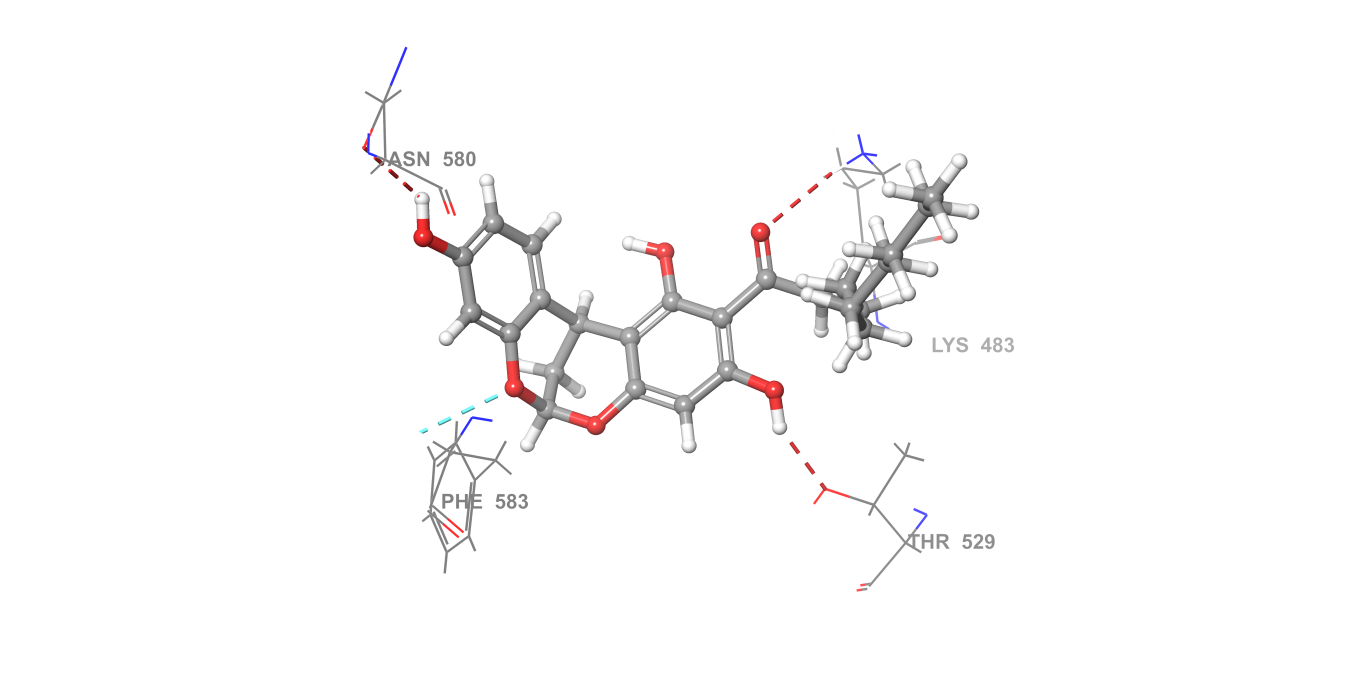

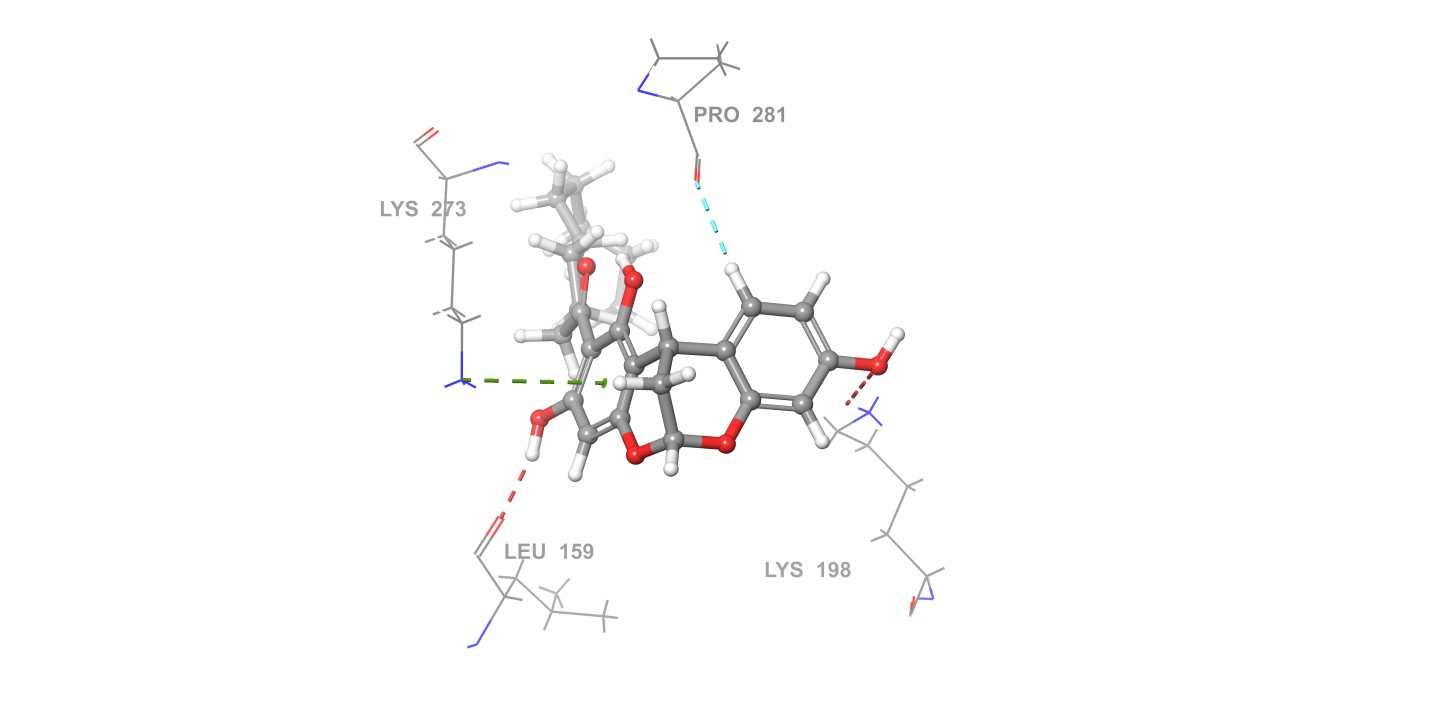


b c

**b) Results for myristicyclin A (U2)**

Interactions with the C2 active site of BRAF V600b (associated with cancer), and the M2 active site of PFMDH (associated with malaria).

**
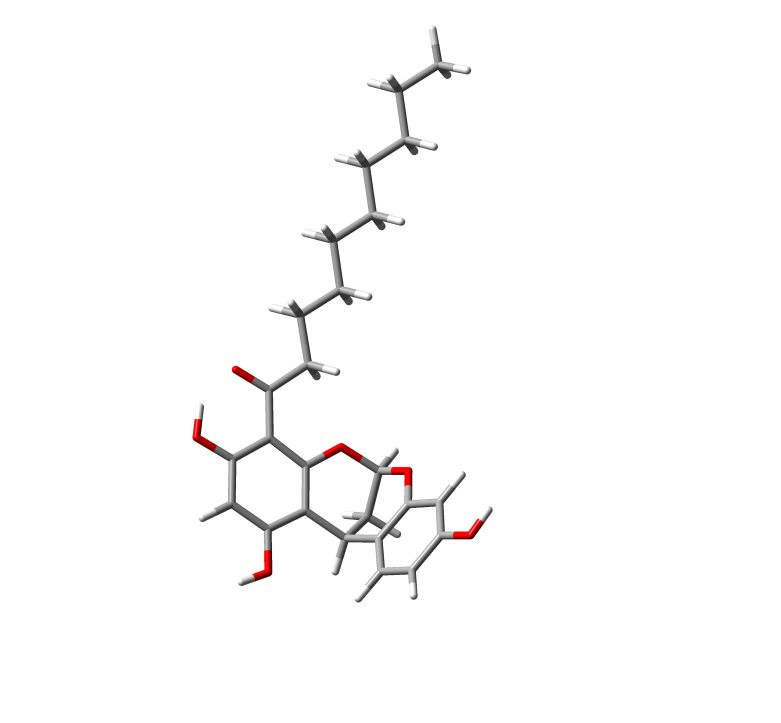
**

a


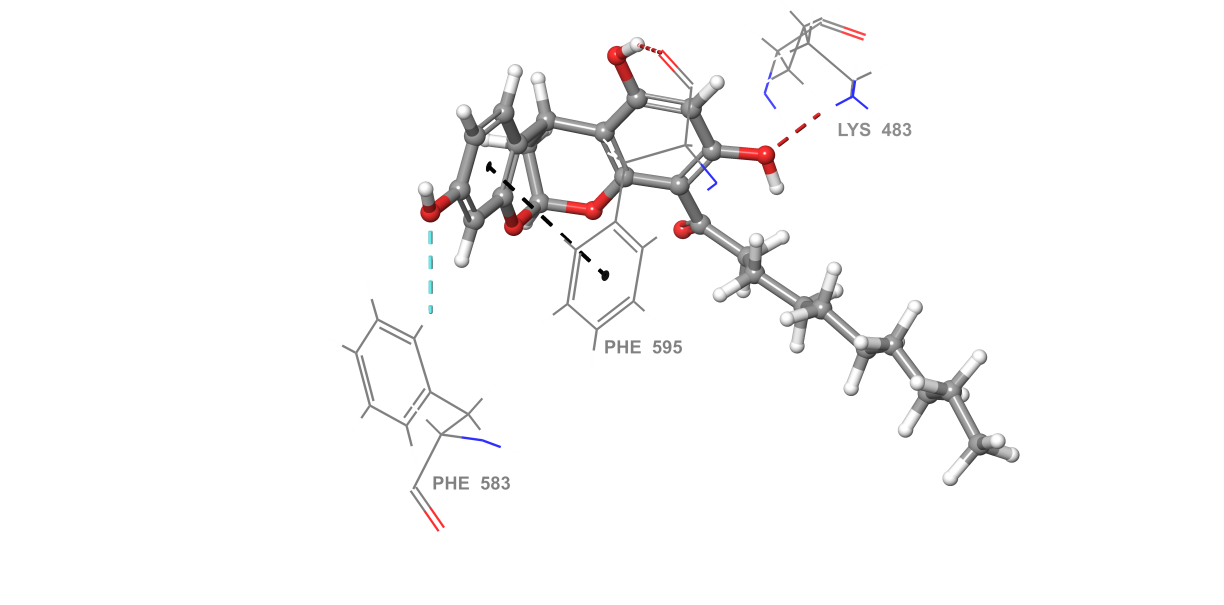

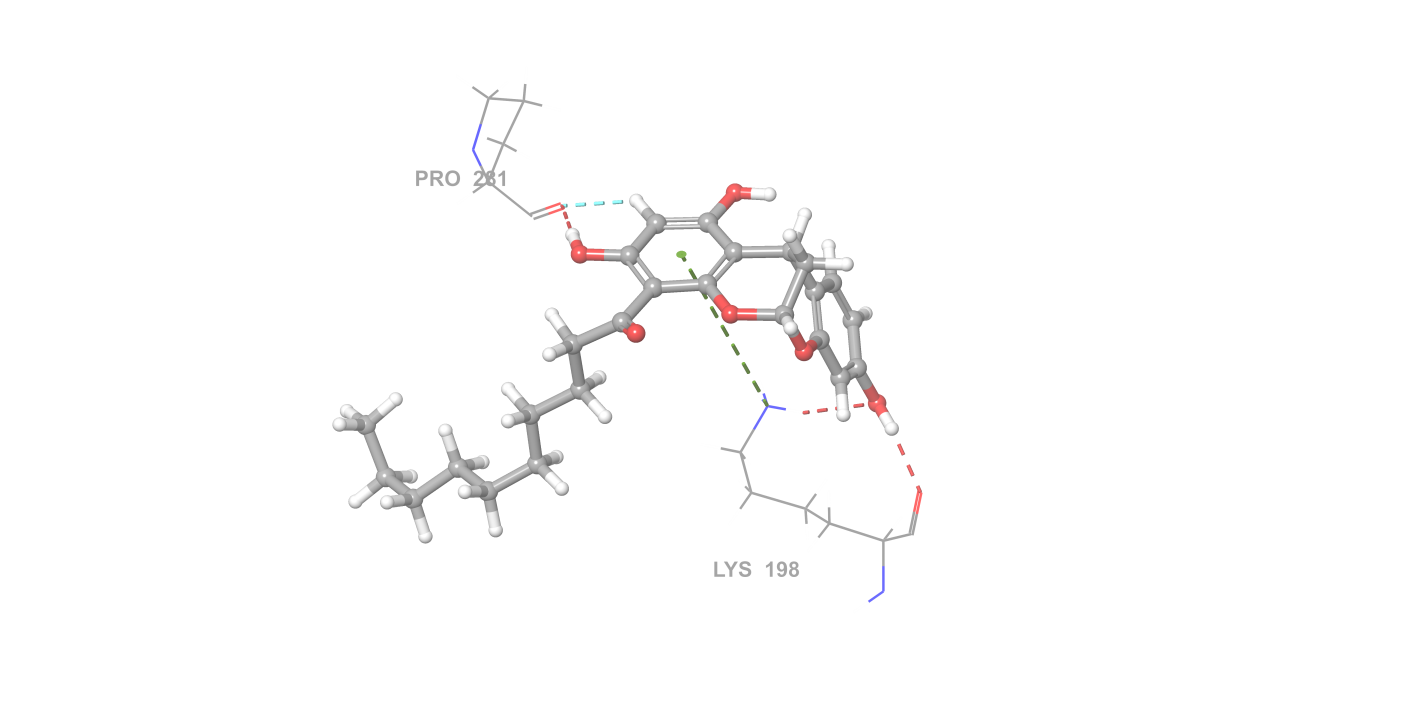


b c

**c) Results for myristicyclin B (U3)**

Interactions with the C1active site of BRAF V600b (associated with cancer), and the M2 active site of PFMDH (associated with malaria).

**
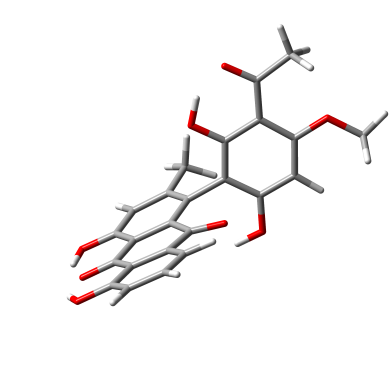
**

a


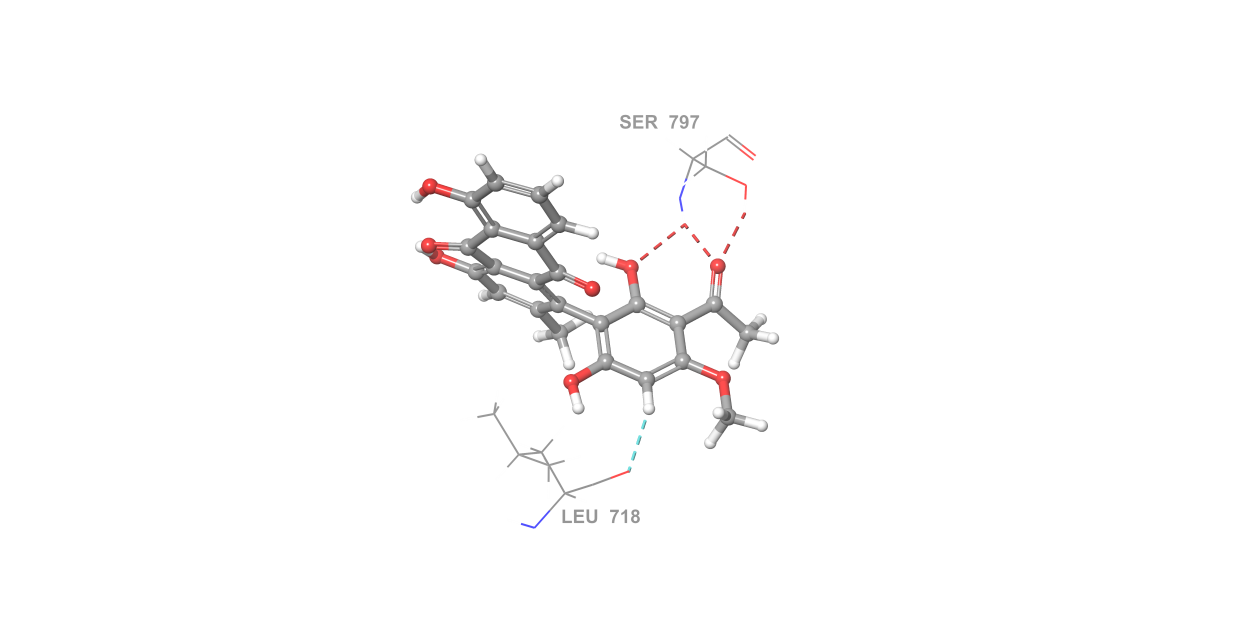

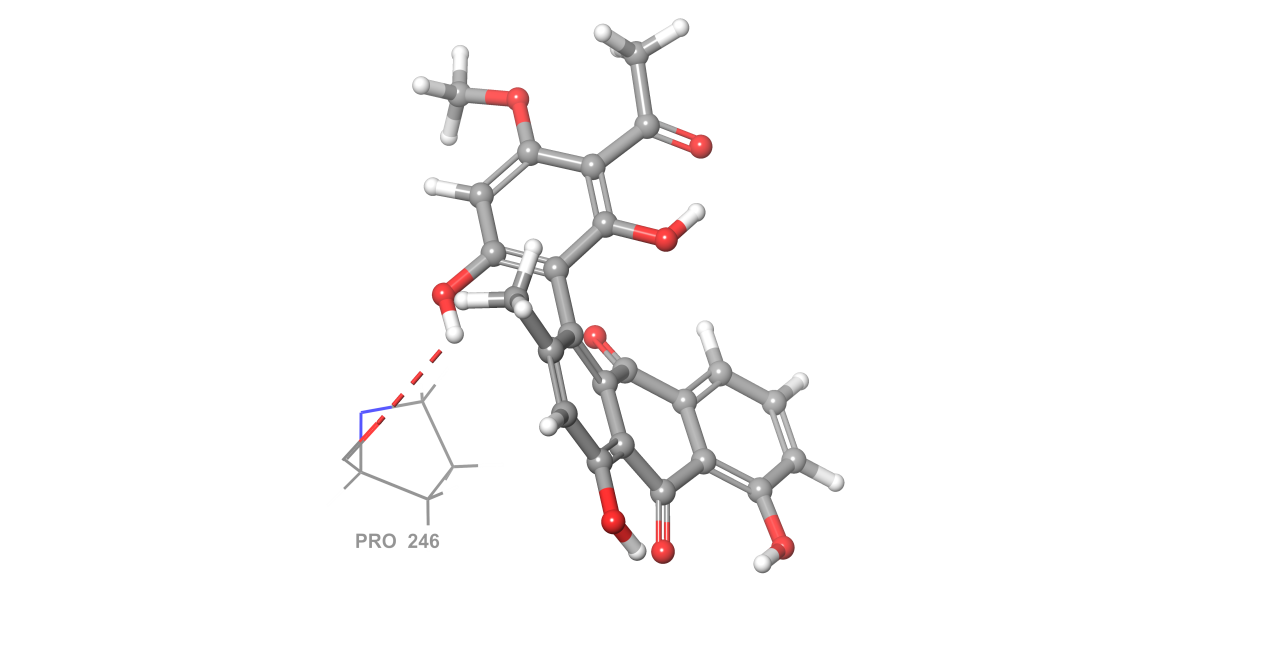


b c

**d) Results for knipholone (U4)**

Interactions with the C1 active site of BRAF V600b (associated with cancer), and the active site of PFLDH (associated with malaria).

**
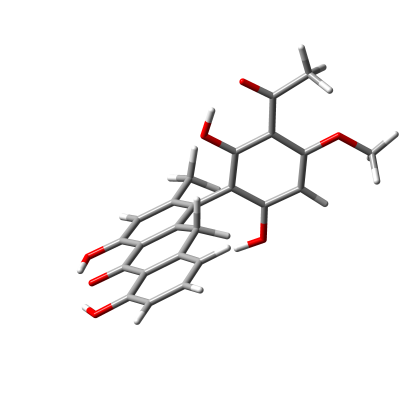
**

a


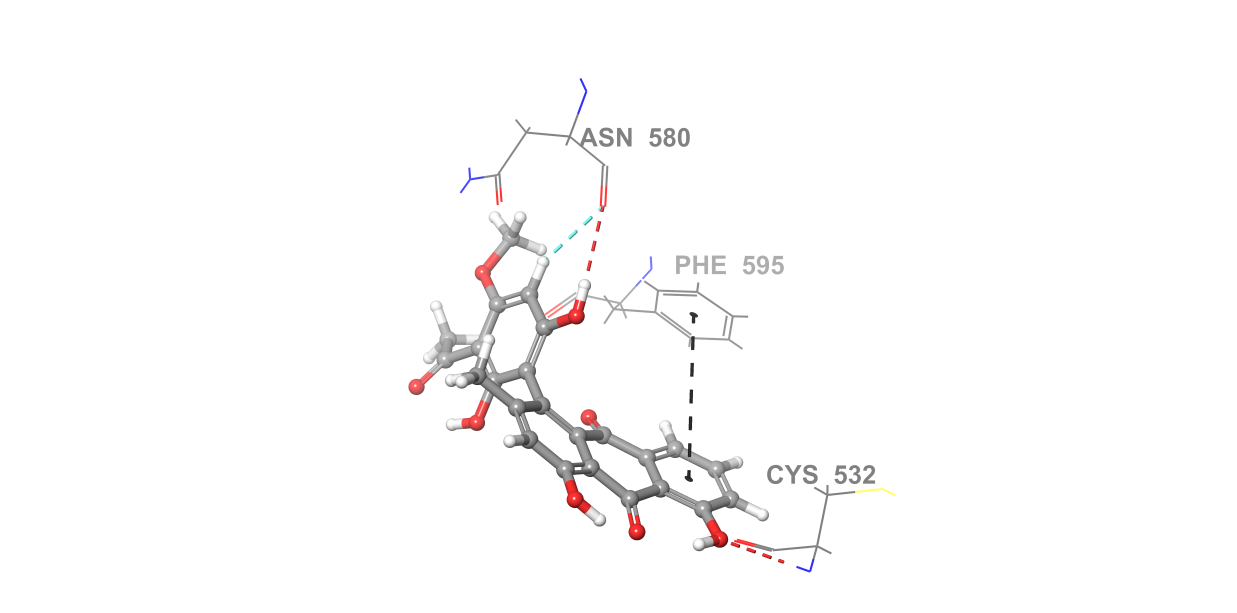

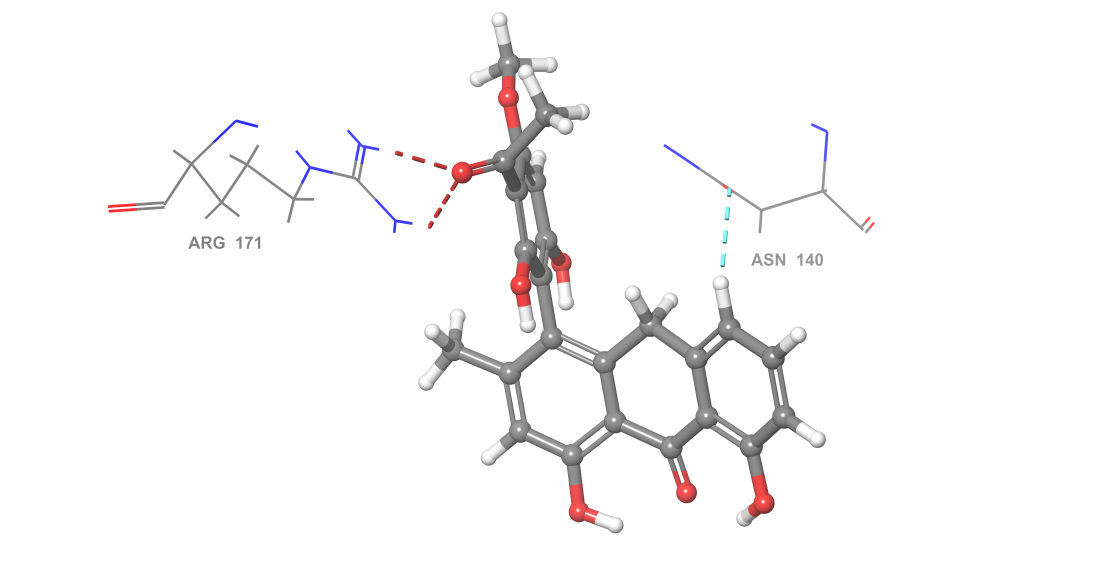


b c

**e) Results for knipholoneanthrone (U5)**

Interactions with the C1 active site of BRAF V600b (associated with cancer), and the active site of PFLDH (associated with malaria).

**
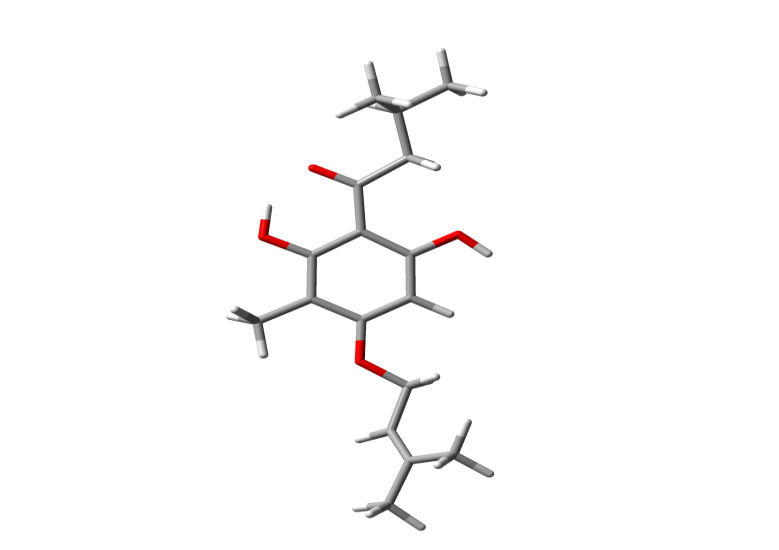
**

a


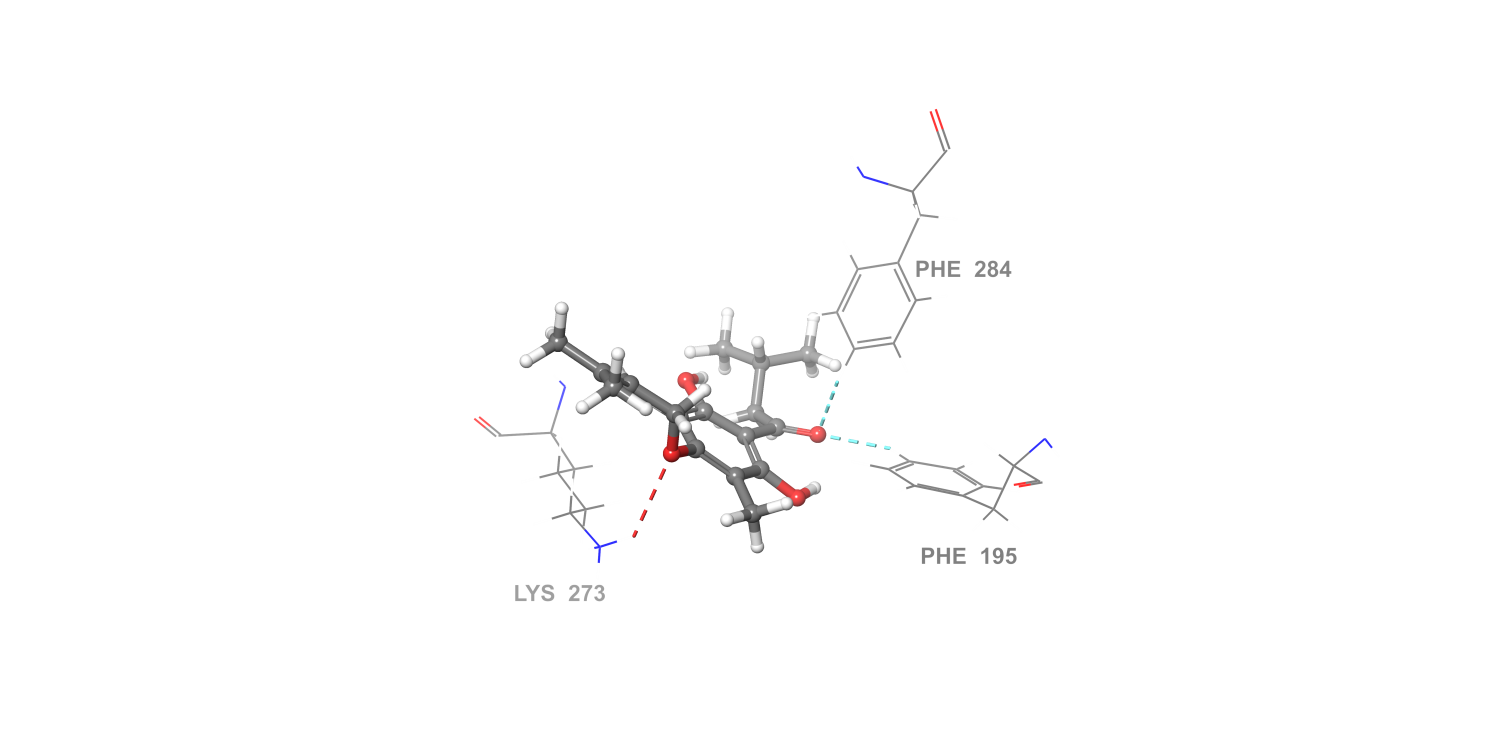

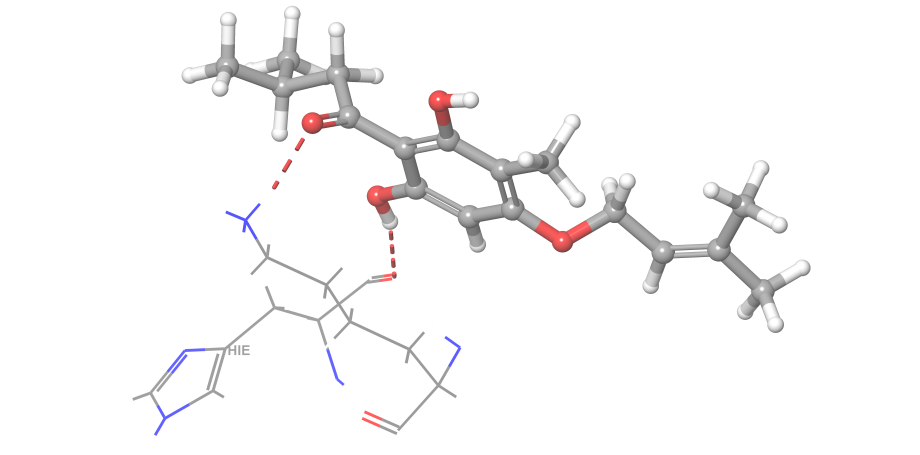


b c

**f) Results for 1-(2,6-dihydroxy-3-methyl-4-((3-methylbut-2-en-1-yl)oxy)phenyl)-3-methylbutan-1-one (U6)**

Interactions with the C1 active site of BRAF V600b (associated with cancer), and the M1 active site of PFMDH (associated with malaria).

**
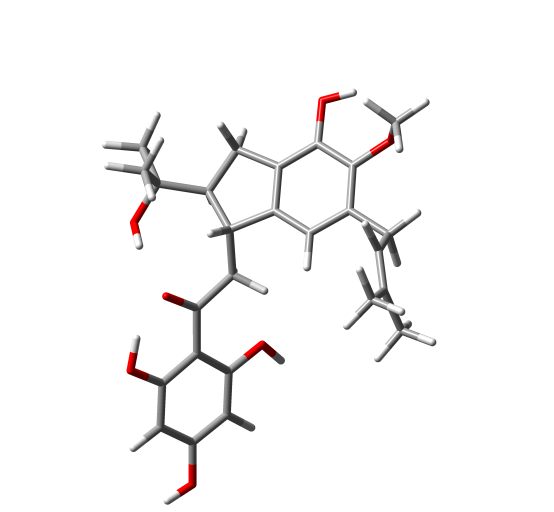
**

a


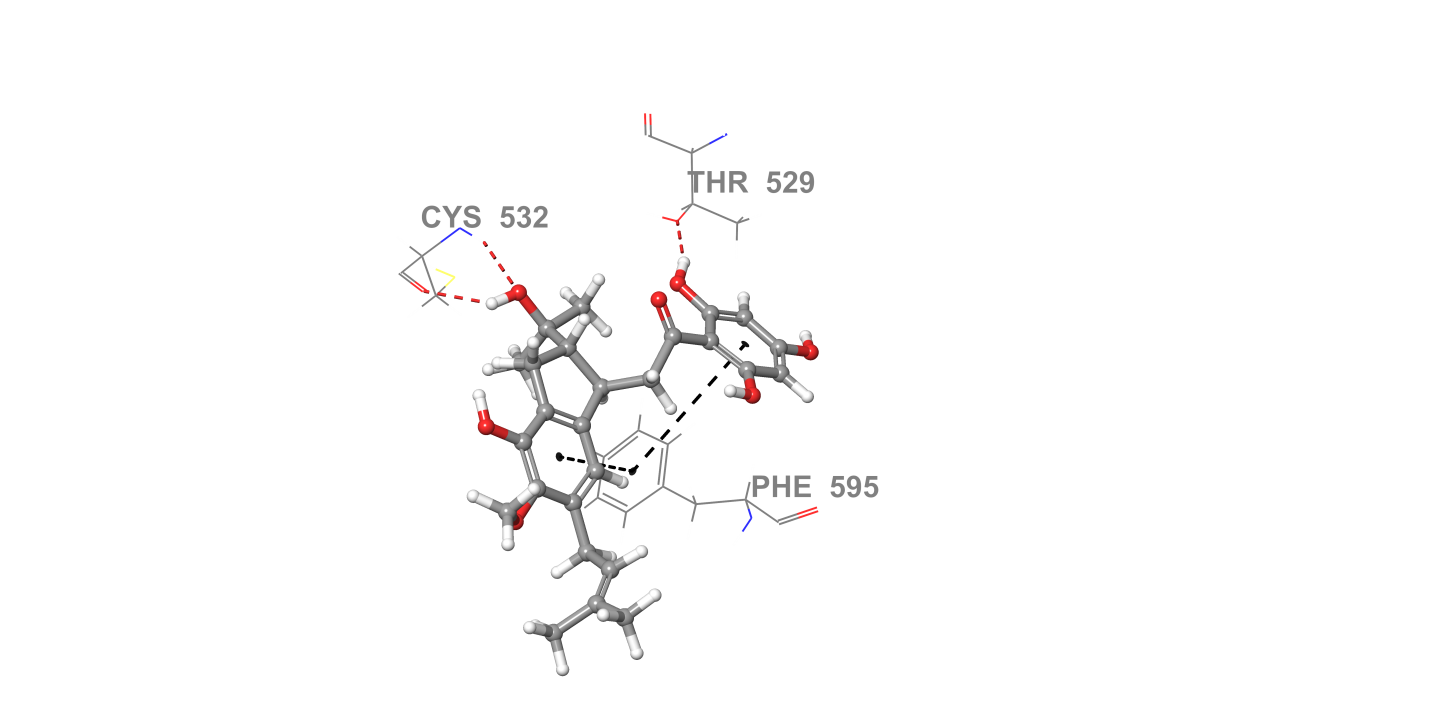

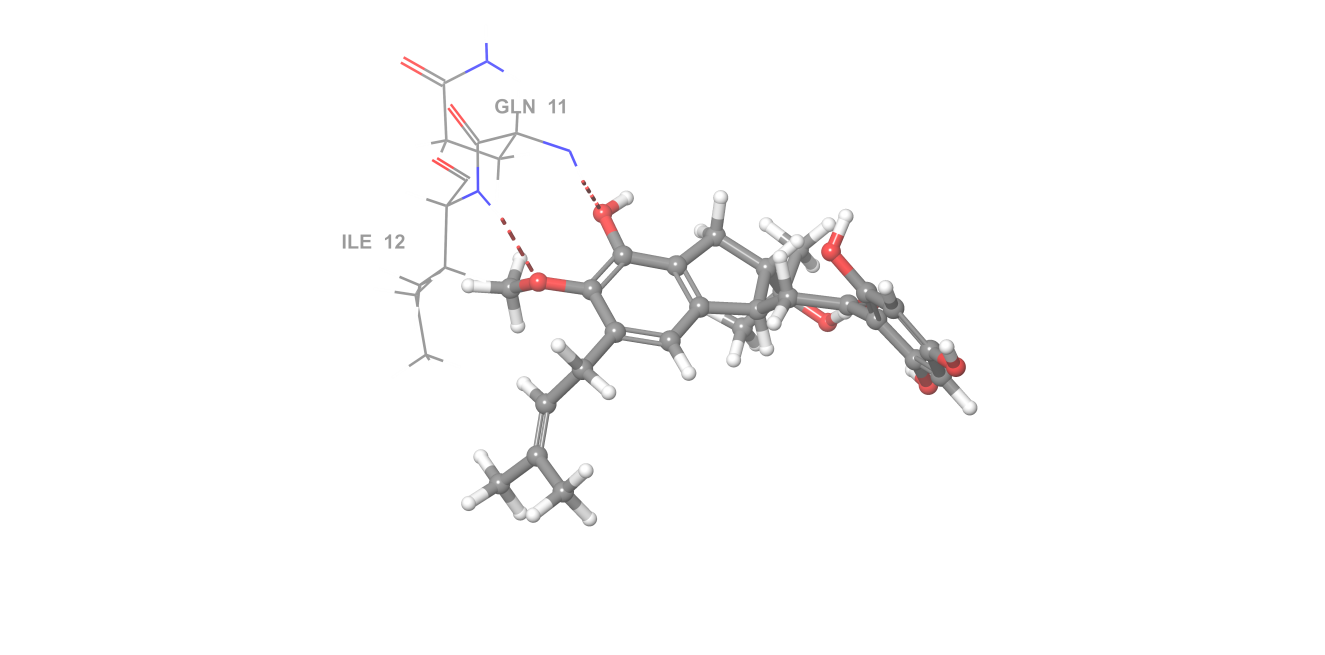


b c

**g) Results for antiarone J (U7)**

Interactions with the C1 active site of BRAF V600b (associated with cancer), and the M3 active site of PFMDH (associated with malaria).

**
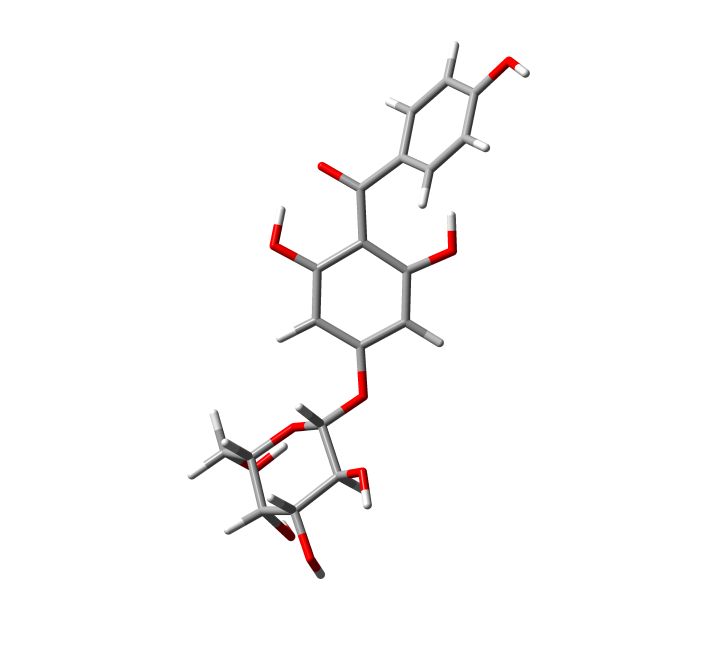
**

a


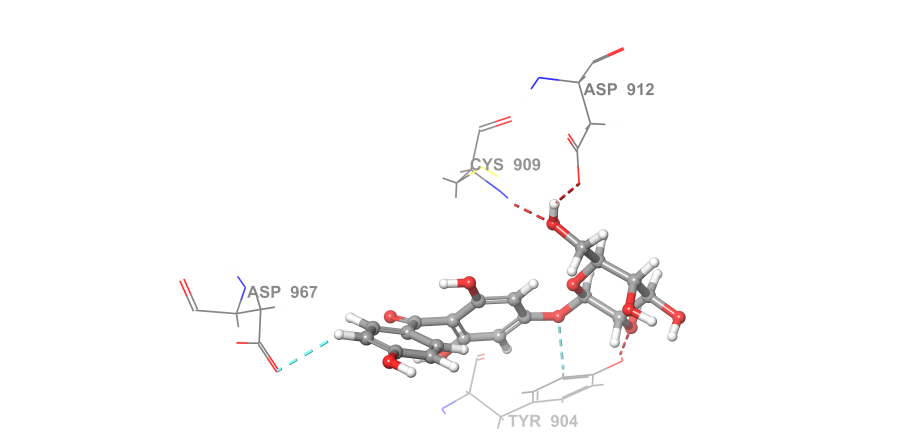

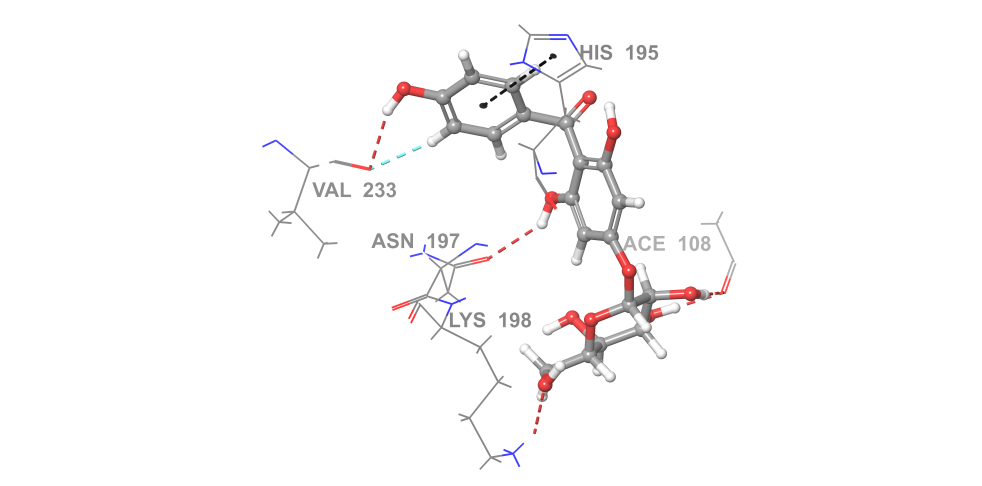


b c

**h) Results for iriflophenone4-glucoside (U8)**

Interactions with the H1 active site of HER2 (associated with cancer), and the active site of PFLDH (associated with malaria).
