# Supplementary material for: Computable properties of selected monomeric acylphloroglucinols with anticancer and/or antimalarial activities and first-approximation docking study
Source: J Mol Model. 2025 Mar 12;31(4):113. doi: 10.1007/s00894-025-06299-7 (PMC11903629; doi:10.1007/s00894-025-06299-7)
Supplement: Supplementary file 15 — (DCOX 24.1 KB) [file 894_2025_6299_MOESM15_ESM.docx]

**Table S1.**

**Relative energies *in vacuo* of the calculated conformers of the ACPL molecules considered in this work.**

Results from full optimisation calculations HF/6-31G(d,p), DFT/B3LYP/6-31+G(d,p) and MP2/ 6-31G(d,p) calculations, respectively denoted as DFT, HF and MP2 in the columns’ headings. For each molecule, the conformers are listed in order of increasing relative energies in the DFT results.

| Molecules and conformers | Relative energy (kcal mol^-1^) | | |
| --- | --- | --- | --- |
|  | DFT | HF | MP2 |
| **U1** |  |  |  |
| U1-d-r-a | 0.000 | 0.000 | 0.000 |
| U1-d-w-a | 1.380 | 1.516 | 1.402 |
| U1-d-u-r-a | 2.879 | 4.373 | 2.820 |
| U1-d-u-w-a | 3.229 | 4.785 | 3.189 |
| U1-r-a | 14.717 | 15.476 | 12.423 |
|  |  |  |  |
| **U2** |  |  |  |
| U2-d-v-a | 0.000 | 0.041 | 0.051 |
| U2-s-v-a | 0.080 | 0.000 | 0.000 |
| U2-s-v-u-a | 4.178 | 5.019 | 3.165 |
| U2-d-x-a | 4.327 | 5.822 | 4.096 |
| U2-x-a | 16.643 | 14.228 | 13.458 |
|  |  |  |  |
| **U3** |  |  |  |
| U3-s-x-w-a | 0.000 | 0.000 | 3.334 |
| U3-s-v-w-a | 0.030 | 0.135 | 3.491 |
| U3-s-x-w-b | 0.534 | 1.024 | 0.000 |
| U3-s-x-r-a | 3.135 | 3.994 | 5.772 |
| U3-z-x-w | 13.628 | 10.884 | 14.957 |
| U3-v-w-a | 13.739 | 11.128 | 15.203 |
|  |  |  |  |
| **U4** |  |  |  |
| U4-d-ε-r-x-j | 0.000 | 0.000 | 0.000 |
| U4-d-w-x-j | 2.091 | 2.669 | 3.410 |
| U4-d-ε-r-v-j | 12.259 | 10.941 | 11.408 |
| U4-d-ε-r-x-k | 12.923 | 11.510 | 12.342 |
| U4-d-w-v-k | 29.374 | 26.088 | 27.939 |
| U4-w-v-k | 42.418 | 36.168 | 37.840 |
|  |  |  |  |
| **U5** |  |  |  |
| U5-d-r-x-j | 0.000 | 0.000 | 0.000 |
| U5-d-w-x-j | 3.873 | 4.058 | 4.286 |
| U5-d-r-v-j | 12.880 | 11.948 | 11.683 |
| U5-d-r-x-k | 13.489 | 12.498 | 12.124 |
| U5-r-x-j | 13.528 | 10.372 | 10.671 |
| U5-d-w-v-k | 31.954 | 29.280 | 28.820 |
|  |  |  |  |
| **U6** |  |  |  |
| U6-d-w-e | 0.000 | 0.000 | 0.461 |
| U6-d-w-g | 0.418 | 0.889 | 0.000 |
| U6-d-w-c | 0.443 | 0.887 | 0.000 |
| U6-s-w-f | 1.133 | 1.064 | 1.320 |
| U6-d-w-e-u | 1.939 | 3.007 | 1.929 |
| U6-d-w-f | 1.950 | 1.775 | 1.821 |
| U6-d-w-h | 2.946 | 3.662 | 1.862 |
| U6-d-y-f | 5.042 | 3.512 | 3.671 |
| U6-d-m-f | 5.605 | 4.632 | 2.814 |
| U6-w-f | 15.142 | 11.821 | 12.619 |
|  |  |  |  |
| **U7** |  |  |  |
| U7-d-r-ᴧ-χ-α-p | 0.000 | 0.000 | 0.000 |
| U7-d-w-ᴧ-χ-α-p | 1.325 | 1.466 | 1.321 |
| U7-d-w-ᴧ-χ-α-q | 1.561 | 1.589 | 2.252 |
| U7-d-w-ᴧ-χ-β-p | 1.845 | 1.815 | 0.150 |
| U7-d-w-χ-α-p | 2.839 | 2.088 | 1.293 |
| U7-d-w-ᴧ-χ-α-p-u | 3.061 | 3.925 | 1.743 |
| U7-d-w-ᴧ-λ-α-q | 4.390 | 4.757 | 7.354 |
| U7-d-w-ᴧ-λ-α-p | 4.430 | 5.015 | 4.779 |
| U7-d-w-γ-χ-p | 5.497 | 4.989 | 5.216 |
| U7-w-ᴧ-χ-α-p | 14.314 | 11.687 | 9.434 |
|  |  |  |  |
| **U8** |  |  |  |
| U8-ƞ-d-u-y-κ-ω | 0.000 | 0.951 | 0.019 |
| U8-ƞ-d-u-y-κ-t | 0.000 | 1.038 | 0.000 |
| U8-ƞ-d-u-w-μ-t | 1.213 | 0.000 | 1.416 |
| U8-d-y-κ-ω | 1.378 | 1.607 | 3.948 |
| U8-ƞ-d-u-r-ξ-t | 1.335 | 0.071 | 0.781 |
| U8-ƞ-d-u-y-ς-t | 2.343 | 3.011 | 4.258 |
| U8-ƞ-d-u-y-δ-ω | 2.907 | 4.323 | 4.556 |
| U8-ƞ-d-u-y-δ-t | 2.961 | 4.441 | 4.493 |
| U8-ƞ-d-u-r-δ-n | 3.079 | 2.399 | 4.007 |
| U8-ƞ-d-u-w-δ-t | 3.664 | 2.972 | 4.493 |
| U8-ƞ-s-u-w-τ-t | 5.166 | 4.714 | 5.541 |
| U8-y-κ-ω | 11.949 | 7.493 | 10.096 |
