# Supplementary material for: Computable properties of selected monomeric acylphloroglucinols with anticancer and/or antimalarial activities and first-approximation docking study
Source: J Mol Model. 2025 Mar 12;31(4):113. doi: 10.1007/s00894-025-06299-7 (PMC11903629; doi:10.1007/s00894-025-06299-7)
Supplement: Supplementary file 16 — (DOCX 23.4 KB) [file 894_2025_6299_MOESM16_ESM.docx]

**Table S2.**

**Relative energy corrected for ZPE (sum of electronic and zero-point energies, ΔE_corrected_, kcal mol^-^**^1^**), ZPE correction to the electronic energy (ZPE_corr_, kcal mol^-1^), relative Gibbs free energies (sum of electronic and thermal free energy, ΔG_corrected_) and corresponding thermal corrections (G_corr_), for the calculated conformers of the considered ACPL molecules.**

DFT/B3LYP/6-31+G(d,p) results *in vacuo*.

The corrected energies and corresponding corrections are from harmonic-approximation frequency calculations. For each molecule, the conformers are listed in order of increasing relative energies in the DFT results.

| Molecules and conformers | ΔE_corrected_ | ZPE_corr_ | ΔG_corrected_ | G_corr_ |
| --- | --- | --- | --- | --- |
| **U1** |  |  |  |  |
| U1-d-r-a | 0.000 | 365.26 | 0.000 | 320.57 |
| U1-d-w-a | 1.155 | 365.04 | 0.858 | 320.05 |
| U1-d-u-r-a | 2.914 | 365.30 | 2.759 | 320.45 |
| U1-d-u-w-a | 3.502 | 365.11 | 6.164 | 323.08 |
| U1-r-a | 13.742 | 364.29 | 13.218 | 319.07 |
|  |  |  |  |  |
| **U2** |  |  |  |  |
| U2-d-v-a | 0.000 | 320.72 | 0.000 | 281.11 |
| U2-s-v-a | 0.131 | 320.69 | 0.171 | 281.17 |
| U2-s-v-u-a | 4.146 | 320.77 | 4.234 | 281.20 |
| U2-d-x-a | 0.131 | 320.77 | 0.169 | 281.20 |
| U2-x-a | 15.609 | 319.69 | 13.995 | 278.46 |
|  |  |  |  |  |
| **U3** |  |  |  |  |
| U3-s-x-w-a | 0.023 | 320.67 | 0.279 | 280.46 |
| U3-s-v-w-a | 0.000 | 320.62 | 0.000 | 280.16 |
| U3-s-x-w-b | 1.044 | 321.16 | 1.817 | 281.47 |
| U3-s-x-r-a | 2.971 | 320.48 | 3.229 | 280.28 |
| U3-z-x-w | 12.853 | 319.87 | 12.572 | 279.13 |
| U3-v-w-a | 12.912 | 319.82 | 12.590 | 279.04 |
|  |  |  |  |  |
| **U4** |  |  |  |  |
| U4-d-ε-r-x-j | 0.000 | 236.20 | 0.000 | 199.65 |
| U4-d-w-x-j | 1.952 | 236.06 | 1.817 | 199.37 |
| U4-d-ε-r-v-j | 11.829 | 235.77 | 11.606 | 198.99 |
| U4-d-ε-r-x-k | 12.381 | 235.66 | 11.994 | 198.72 |
| U4-d-w-v-k | 28.190 | 235.02 | 27.465 | 197.74 |
| U4-w-v-k | 40.397 | 234.18 | 38.696 | 195.93 |
|  |  |  |  |  |
| **U5** |  |  |  |  |
| U5-d-r-x-j | 0.000 | 247.93 | 0.000 | 212.11 |
| U5-d-w-x-j | 3.657 | 247.71 | 3.545 | 211.78 |
| U5-d-r-v-j | 12.361 | 247.41 | 11.927 | 211.16 |
| U5-d-r-x-k | 12.989 | 247.43 | 12.356 | 210.97 |
| U5-r-x-j | 12.558 | 246.96 | 11.120 | 209.70 |
| U5-d-w-v-k | 30.774 | 246.75 | 29.891 | 210.04 |
|  |  |  |  |  |
| **U6** |  |  |  |  |
| U6-d-w-e | 0.000 | 238.83 | 0.000 | 204.37 |
| U6-d-w-g | 0.725 | 239.13 | 0.540 | 204.49 |
| U6-d-w-c | 0.680 | 239.06 | 0.169 | 204.09 |
| U6-s-w-f | 1.339 | 239.03 | 1.999 | 205.23 |
| U6-d-w-e-u | 2.163 | 239.05 | 2.341 | 204.77 |
| U6-d-w-f | 1.985 | 238.86 | 1.955 | 204.37 |
| U6-d-w-h | 3.324 | 239.21 | 3.302 | 204.72 |
| U6-d-y-f | 4.972 | 238.76 | 4.787 | 204.11 |
| U6-d-m-f | 5.858 | 239.08 | 5.966 | 204.73 |
| U6-w-f | 14.401 | 238.09 | 13.943 | 203.17 |
|  |  |  |  |  |
| **U7** |  |  |  |  |
| U7-d-r-ᴧ-χ-α-p | 0.000 | 338.40 | 0.000 | 296.26 |
| U7-d-w-ᴧ-χ-α-p | 1.189 | 338.26 | 1.129 | 296.06 |
| U7-d-w-ᴧ-χ-α-q | 1.524 | 338.36 | 1.464 | 296.16 |
| U7-d-w-ᴧ-χ-β-p | 1.761 | 338.32 | 1.669 | 296.08 |
| U7-d-w-χ-α-p | 2.196 | 337.76 | 1.748 | 295.17 |
| U7-d-w-ᴧ-χ-α-p-u | 3.011 | 338.35 | 2.846 | 296.04 |
| U7-d-w-ᴧ-λ-α-q | 4.084 | 338.09 | 3.843 | 295.71 |
| U7-d-w-ᴧ-λ-α-p | 4.009 | 337.98 | 3.473 | 295.30 |
| U7-d-w-γ-χ-p | 4.762 | 337.66 | 4.357 | 295.12 |
| U7-w-ᴧ-χ-α-p | 13.635 | 337.72 | 12.570 | 294.51 |
|  |  |  |  |  |
| **U8** |  |  |  |  |
| U8-ƞ-d-u-y-κ-ω | 0.000 | 239.73 | 0.000 | 203.68 |
| U8-ƞ-d-u-y-κ-t | 0.033 | 239.73 | 0.021 | 203.66 |
| U8-ƞ-d-u-w-μ-t | 1.040 | 239.52 | 1.177 | 203.60 |
| U8-d-y-κ-ω | 1.273 | 239.63 | 1.188 | 203.49 |
| U8-ƞ-d-u-r-ξ-t | 1.264 | 239.63 | 1.294 | 203.60 |
| U8-ƞ-d-u-y-ς-t | 2.169 | 239.52 | 2.272 | 203.57 |
| U8-ƞ-d-u-y-δ-ω | 2.368 | 239.16 | 1.950 | 202.68 |
| U8-ƞ-d-u-y-δ-t | 2.418 | 239.15 | 2.002 | 202.68 |
| U8-ƞ-d-u-r-δ-n | 2.588 | 239.21 | 2.272 | 202.83 |
| U8-ƞ-d-u-w-δ-t | 3.171 | 239.20 | 2.816 | 202.79 |
| U8-ƞ-s-u-w-τ-t | 4.646 | 239.18 | 4.536 | 203.01 |
| U8-y-κ-ω | 11.072 | 238.86 | 9.653 | 201.38 |
