# Supplementary material for: Computable properties of selected monomeric acylphloroglucinols with anticancer and/or antimalarial activities and first-approximation docking study
Source: J Mol Model. 2025 Mar 12;31(4):113. doi: 10.1007/s00894-025-06299-7 (PMC11903629; doi:10.1007/s00894-025-06299-7)
Supplement: Supplementary file 17 — (DOCX 23.3 KB) [file 894_2025_6299_MOESM17_ESM.docx]

**Table S3.**

**Relative energy corrected for ZPE (sum of electronic and zero-point energies, ΔE_corrected_, kcal mol^-^**^1^**), ZPE correction to the electronic energy (ZPE_corr_, kcal mol^-1^), relative Gibbs free energies (sum of electronic and thermal free energy, ΔG_corrected_) and corresponding thermal correction (G_corr_), for the conformers of considered simple ACPLs.**

HF/6-31G(d,p) results *in vacuo*.

The corrected energies and corresponding corrections are from harmonic-approximation frequency calculations. For each molecule, the conformers are listed in order of increasing relative energies in the DFT results.

| Molecules and conformers | ΔE_corrected_ | ZPE_corr_ | ΔG_corrected_ | G_corr_ |
| --- | --- | --- | --- | --- |
| **U1** |  |  |  |  |
| U1-d-r-a | 0.000 | 391.52 | 0.000 | 347.59 |
| U1-d-w-a | 1.384 | 391.39 | 1.318 | 347.40 |
| U1-d-u-r-a | 4.365 | 391.51 | 4.313 | 347.53 |
| U1-d-u-w-a | 4.765 | 391.50 | 4.682 | 347.49 |
| U1-r-a | 14.775 | 390.82 | 15.749 | 347.87 |
|  |  |  |  |  |
| **U2** |  |  |  |  |
| U2-d-v-a | 0.008 | 344.75 | 0.011 | 305.90 |
| U2-s-v-a | 0.000 | 344.52 | 0.000 | 305.16 |
| U2-s-v-u-a | 4.759 | 344.74 | 4.255 | 305.83 |
| U2-d-x-a | 5.775 | 344.79 | 5.721 | 305.93 |
| U2-x-a | 12.848 | 343.41 | 11.423 | 303.12 |
|  |  |  |  |  |
| **U3** |  |  |  |  |
| U3-s-x-w-a | 0.000 | 344.77 | 0.000 | 305.76 |
| U3-s-v-w-a | 0.860 | 344.73 | 0.105 | 305.73 |
| U3-s-x-w-b | 2.162 | 345.14 | 1.089 | 305.82 |
| U3-s-x-r-a | 4.491 | 344.50 | 3.312 | 305.07 |
| U3-z-x-w | 10.471 | 343.59 | 8.675 | 303.55 |
| U3-v-w-a | 10.675 | 343.55 | 8.933 | 303.56 |
|  |  |  |  |  |
| **U4** |  |  |  |  |
| U4-d-ε-r-x-j | 0.000 | 255.20 | 0.000 | 219.64 |
| U4-d-w-x-j | 2.506 | 255.03 | 2.167 | 219.14 |
| U4-d-ε-r-v-j | 10.356 | 254.61 | 10.056 | 218.76 |
| U4-d-ε-r-x-k | 10.836 | 254.52 | 10.820 | 218.95 |
| U4-d-w-v-k | 24.590 | 253.70 | 23.910 | 217.46 |
| U4-w-v-k | 33.544 | 252.57 | 31.901 | 215.37 |
|  |  |  |  |  |
| **U5** |  |  |  |  |
| U5-d-r-x-j | 0.000 | 267.48 | 0.000 | 232.10 |
| U5-d-w-x-j | 3.743 | 267.17 | 3.416 | 231.45 |
| U5-d-r-v-j | 11.445 | 266.98 | 11.116 | 231.26 |
| U5-d-r-x-k | 11.978 | 266.96 | 11.585 | 231.18 |
| U5-r-x-j | 9.189 | 266.30 | 8.101 | 229.82 |
| U5-d-w-v-k | 27.826 | 266.03 | 27.420 | 230.24 |
|  |  |  |  |  |
| **U6** |  |  |  |  |
| U6-d-w-e | 0.000 | 256.58 | 0.000 | 222.95 |
| U6-d-w-g | 1.156 | 256.85 | 0.937 | 223.00 |
| U6-d-w-c | 1.153 | 256.85 | 0.937 | 223.00 |
| U6-s-w-f | 1.303 | 256.82 | 1.522 | 223.41 |
| U6-d-w-e-u | 3.068 | 256.64 | 3.074 | 223.02 |
| U6-d-w-f | 1.869 | 256.68 | 1.835 | 223.01 |
| U6-d-w-h | 4.025 | 256.95 | 3.511 | 222.80 |
| U6-d-y-f | 3.436 | 256.51 | 3.114 | 222.55 |
| U6-d-m-f | 4.776 | 256.73 | 4.359 | 222.67 |
| U6-w-f | 10.796 | 255.56 | 9.915 | 221.04 |
|  |  |  |  |  |
| **U7** |  |  |  |  |
| U7-d-r-ᴧ-χ-α-p | 0.000 | 364.27 | 0.000 | 323.15 |
| U7-d-w-ᴧ-χ-α-p | 1.337 | 364.14 | 1.298 | 322.98 |
| U7-d-w-ᴧ-χ-α-q | 1.487 | 364.16 | 1.426 | 322.99 |
| U7-d-w-ᴧ-χ-β-p | 1.705 | 364.16 | 1.754 | 323.09 |
| U7-d-w-χ-α-p | 1.633 | 363.81 | 2.025 | 323.09 |
| U7-d-w-ᴧ-χ-α-p-u | 3.987 | 364.33 | 4.120 | 323.35 |
| U7-d-w-ᴧ-λ-α-q | 4.340 | 363.85 | 4.283 | 322.68 |
| U7-d-w-ᴧ-λ-α-p | 4.527 | 363.78 | 4.064 | 322.20 |
| U7-d-w-γ-χ-p | 4.386 | 363.66 | 4.364 | 322.53 |
| U7-w-ᴧ-χ-α-p | 10.912 | 363.49 | 10.033 | 321.50 |
|  |  |  |  |  |
| **U8** |  |  |  |  |
| U8-ƞ-d-u-y-κ-ω | 0.968 | 259.99 | 0.000 | 223.91 |
| U8-ƞ-d-u-y-κ-t | 1.040 | 259.98 | 0.079 | 223.90 |
| U8-ƞ-d-u-w-μ-t | 0.000 | 259.98 | 0.139 | 225.00 |
| U8-d-y-κ-ω | 1.397 | 259.77 | 0.136 | 223.39 |
| U8-ƞ-d-u-r-ξ-t | 0.171 | 260.07 | 0.324 | 225.11 |
| U8-ƞ-d-u-y-ς-t | 2.886 | 259.85 | 1.958 | 223.81 |
| U8-ƞ-d-u-y-δ-ω | 3.783 | 259.44 | 2.583 | 223.12 |
| U8-ƞ-d-u-y-δ-t | 3.876 | 259.41 | 2.631 | 223.05 |
| U8-ƞ-d-u-r-δ-n | 2.046 | 259.62 | 1.845 | 224.30 |
| U8-ƞ-d-u-w-δ-t | 2.588 | 259.59 | 2.358 | 224.24 |
| U8-ƞ-s-u-w-τ-t | 4.069 | 259.33 | 4.297 | 224.44 |
| U8-y-κ-ω | 6.552 | 259.03 | 4.168 | 221.53 |
