# Supplementary material for: Computable properties of selected monomeric acylphloroglucinols with anticancer and/or antimalarial activities and first-approximation docking study
Source: J Mol Model. 2025 Mar 12;31(4):113. doi: 10.1007/s00894-025-06299-7 (PMC11903629; doi:10.1007/s00894-025-06299-7)
Supplement: Supplementary file 18 — (DOCX 56.9 KB) [file 894_2025_6299_MOESM18_ESM.docx]

**Table S4.**

**Parameters of the intramolecular hydrogen bonds in the calculated conformers of the considered ACPL molecules.**

DFT/B3LYP/6-31+G(d,p), HF/6-31G(d,p) and MP2/6-31G(d,p) results *in vacuo*, from full optimisation calculations, respectively denoted as DFT, HF and MP2 in the columns’ headings.

The various IHBs are considered individually, across the molecules and conformers in which they are present. The molecules are denoted with the symbols listed in table 1, and the conformers with the symbols listed in table 2. For each molecule, the conformers are listed in order of increasing relative energies in the DFT results.

| Molecules and conformers | DFT | | | HF | | | MP2 | | |
| --- | --- | --- | --- | --- | --- | --- | --- | --- | --- |
|  | OH···O  (Å) | O···O  (Å) | OĤO  (°) | OH···O  (Å) | O···O  (Å) | OĤO  (°) | OH···O  (Å) | O···O  (Å) | OĤO  (°) |
| H15···O14 | | | | | | | | | |
| **U1** |  |  |  |  |  |  |  |  |  |
| U1-d-r-a | 1.563 | 2.486 | 150.2 | 1.692 | 2.532 | 144.3 | 1.615 | 2.523 | 149.9 |
| U1-d-w-a | 1.576 | 2.490 | 149.5 | 1.714 | 2.545 | 143.4 | 1.643 | 2.540 | 148.7 |
| U1-d-u-r-a | 1.576 | 2.491 | 149.5 | 1.714 | 2.545 | 143.4 | 1.643 | 2.540 | 148.7 |
| U1-d-u-w-a | 1.584 | 2.496 | 149.3 | 1.719 | 2.549 | 143.4 | 1.652 | 2.546 | 148.6 |
|  |  |  |  |  |  |  |  |  |  |
| **U2** |  |  |  |  |  |  |  |  |  |
| U2-d-v-a | 1.545 | 2.475 | 151.0 | 1.672 | 2.520 | 145.2 | 1.599 | 2.513 | 150.5 |
| U2-d-x-a | 1.546 | 2.476 | 150.9 | 1.673 | 2.521 | 145.1 | 1.600 | 2.514 | 150.5 |
|  |  |  |  |  |  |  |  |  |  |
| **U4** |  |  |  |  |  |  |  |  |  |
| U4-d-ε-r-x-j | 1.539 | 2.472 | 151.1 | 1.665 | 2.516 | 145.7 | 1.590 | 2.508 | 151.0 |
| U4-d-w-x-j | 1.547 | 2.476 | 150.8 | 1.671 | 2.520 | 145.4 | 1.601 | 2.515 | 150.5 |
| U4-d-ε-r-v-j | 1.541 | 2.473 | 151.1 | 1.666 | 2.517 | 145.7 | 1.591 | 2.509 | 151.0 |
| U4-d-w-v-k | 1.546 | 2.476 | 151.0 | 1.671 | 2.521 | 145.6 | 1.599 | 2.514 | 150.8 |
|  |  |  |  |  |  |  |  |  |  |
| **U5** |  |  |  |  |  |  |  |  |  |
| U5-d-r-x-j | 1.536 | 2.470 | 151.2 | 1.666 | 2.516 | 145.4 | 1.590 | 2.507 | 150.7 |
| U5-d-w-x-j | 1.541 | 2.473 | 151.1 | 1.670 | 2.518 | 145.3 | 1.595 | 2.510 | 150.7 |
| U5-d-r-v-j | 1.538 | 2.471 | 151.2 | 1.667 | 2.517 | 145.4 | 1.592 | 2.509 | 150.7 |
| U5-d-r-x-k | 1.537 | 2.470 | 151.1 | 1.667 | 2.516 | 145.4 | 1.591 | 2.508 | 150.6 |
| U5-d-w-v-k | 1.546 | 2.476 | 150.9 | 1.671 | 2.519 | 145.3 | 1.598 | 2.512 | 150.6 |
|  |  |  |  |  |  |  |  |  |  |
| **U6** |  |  |  |  |  |  |  |  |  |
| U6-d-w-e | 1.566 | 2.489 | 150.5 | 1.691 | 2.534 | 144.9 | 1.615 | 2.525 | 150.4 |
| U6-d-w-g | 1.553 | 2.480 | 150.9 | 1.681 | 2.527 | 145.2 | 1.610 | 2.522 | 150.6 |
| U6-d-w-c | 1.554 | 2.481 | 150.9 | 1.681 | 2.527 | 145.2 | 1.611 | 2.522 | 150.6 |
| U6-d-w-e-u | 1.585 | 2.498 | 149.7 | 1.722 | 2.555 | 143.8 | 1.649 | 2.547 | 149.2 |
| U6-d-w-f | 1.561 | 2.484 | 150.6 | 1.682 | 2.526 | 145.0 | 1.608 | 2.517 | 150.4 |
| U6-d-w-h | 1.538 | 2.468 | 151.1 | 1.663 | 2.511 | 145.4 | 1.612 | 2.521 | 150.2 |
| U6-d-y-f | 1.556 | 2.480 | 150.5 | 1.680 | 2.521 | 144.6 | 1.608 | 2.515 | 150.0 |
| U6-d-m-f | 1.556 | 2.480 | 150.6 | 1.679 | 2.522 | 144.8 | 1.607 | 2.515 | 150.1 |
|  |  |  |  |  |  |  |  |  |  |
| **U7** |  |  |  |  |  |  |  |  |  |
| U7-d-r-ᴧ-χ-α-p | 1.575 | 2.490 | 149.4 | 1.703 | 2.537 | 143.6 | 1.655 | 2.547 | 148.1 |
| U7-d-w-ᴧ-χ-α-p | 1.583 | 2.495 | 149.2 | 1.710 | 2.542 | 143.5 | 1.665 | 2.555 | 147.9 |
| U7-d-w-ᴧ-χ-α-q | 1.584 | 2.495 | 149.2 | 1.709 | 2.541 | 143.5 | 1.680 | 2.565 | 147.3 |
| U7-d-w-ᴧ-χ-β-p | 1.583 | 2.495 | 149.3 | 1.710 | 2.542 | 143.5 | 1.612 | 2.516 | 149.5 |
| U7-d-w-χ-α-p | 1.565 | 2.486 | 150.1 | 1.714 | 2.547 | 143.6 | 1.619 | 2.525 | 149.6 |
| U7-d-w-ᴧ-χ-α-p-u | 1.601 | 2.504 | 148.5 | 1.746 | 2.568 | 142.5 | 1.683 | 2.570 | 147.7 |
| U7-d-w-ᴧ-λ-α-q | 1.586 | 2.497 | 149.1 | 1.711 | 2.543 | 143.4 | 1.651 | 2.543 | 148.3 |
| U7-d-w-ᴧ-λ-α-p | 1.584 | 2.496 | 149.2 | 1.711 | 2.543 | 143.5 | 1.666 | 2.555 | 147.8 |
| U7-d-w-γ-χ-p | 1.572 | 2.490 | 149.8 | 1.704 | 2.539 | 143.8 | 1.633 | 2.534 | 149.2 |
|  |  |  |  |  |  |  |  |  |  |
| **U8** |  |  |  |  |  |  |  |  |  |
| U8-ƞ-d-u-y-κ-ω | 1.633 | 2.534 | 148.2 | 1.836 | 2.641 | 140.7 | 1.729 | 2.607 | 146.9 |
| U8-ƞ-d-u-y-κ-t | 1.632 | 2.533 | 148.2 | 1.835 | 2.640 | 140.7 | 1.728 | 2.606 | 146.9 |
| U8-ƞ-d-u-w-μ-t | 1.653 | 2.547 | 147.6 | 1.828 | 2.636 | 141.0 | 1.728 | 2.606 | 146.9 |
| U8-d-y-κ-ω | 1.632 | 2.533 | 148.2 | 1.812 | 2.623 | 141.3 | 1.715 | 2.596 | 147.2 |
| U8-ƞ-d-u-r-ξ-t | 1.627 | 2.529 | 148.2 | 1.856 | 2.657 | 140.3 | 1.751 | 2.624 | 146.2 |
| U8-ƞ-d-u-y-ς-t | 1.633 | 2.533 | 148.1 | 1.831 | 2.637 | 140.7 | 1.731 | 2.607 | 146.6 |
| U8-ƞ-d-u-y-δ-ω | 1.631 | 2.533 | 148.3 | 1.839 | 2.644 | 140.6 | 1.729 | 2.607 | 146.9 |
| U8-ƞ-d-u-y-δ-t | 1.631 | 2.533 | 148.4 | 1.838 | 2.643 | 140.7 | 1.728 | 2.606 | 147.0 |
| U8-ƞ-d-u-r-δ-n | 1.634 | 2.535 | 148.2 | 1.808 | 2.621 | 141.5 | 1.712 | 2.595 | 147.4 |
| U8-ƞ-d-u-w-δ-t | 1.652 | 2.546 | 147.7 | 1.826 | 2.635 | 141.1 | 1.728 | 2.606 | 147.0 |
| U8-ƞ-d-u-w-τ-t | 1.656 | 2.549 | 147.5 | 1.831 | 2.638 | 141.0 | 1.730 | 2.608 | 146.9 |
| H17···O14 | | | | | | | | | |
| **U2** |  |  |  |  |  |  |  |  |  |
| U2-s-v-a | 1.570 | 2.489 | 149.8 | 1.696 | 2.532 | 143.9 | 1.630 | 2.532 | 149.4 |
| U2-s-v-u-a | 1.588 | 2.497 | 149.0 | 1.733 | 2.558 | 142.7 | 1.661 | 2.551 | 148.1 |
|  |  |  |  |  |  |  |  |  |  |
| **U3** |  |  |  |  |  |  |  |  |  |
| U3-s-x-w-a | 1.558 | 2.480 | 150.0 | 1.692 | 2.529 | 143.9 | 1.615 | 2.521 | 149.6 |
| U3-s-v-w-a | 1.557 | 2.480 | 150.1 | 1.691 | 2.529 | 143.9 | 1.615 | 2.521 | 149.6 |
| U3-s-x-w-b | 1.545 | 2.472 | 150.3 | 1.681 | 2.521 | 144.2 | 1.667 | 2.560 | 148.1 |
| U3-s-x-r-a | 1.565 | 2.484 | 149.8 | 1.697 | 2.532 | 143.8 | 1.629 | 2.529 | 149.1 |
|  |  |  |  |  |  |  |  |  |  |
| **U6** |  |  |  |  |  |  |  |  |  |
| U6-s-w-f | 1.563 | 2.484 | 150.0 | 1.691 | 2.528 | 144.0 | 1.614 | 2.519 | 149.6 |
| H23···O32 | | | | | | | | | |
| **U4** |  |  |  |  |  |  |  |  |  |
| U4-d-ε-r-x-j | 1.654 | 2.542 | 146.7 | 1.779 | 2.590 | 141.1 | 1.707 | 2.585 | 146.6 |
| U4-d-w-x-j | 1.647 | 2.537 | 146.9 | 1.766 | 2.579 | 141.3 | 1.682 | 2.565 | 147.2 |
| U4-d-ε-r-v-j | 1.615 | 2.520 | 148.2 | 1.785 | 2.598 | 141.3 | 1.687 | 2.576 | 147.7 |
|  |  |  |  |  |  |  |  |  |  |
| **U5** |  |  |  |  |  |  |  |  |  |
| U5-d-r-x-j | 1.657 | 2.549 | 147.3 | 1.758 | 2.578 | 142.2 | 1.695 | 2.579 | 147.3 |
| U5-d-w-x-j | 1.660 | 2.551 | 147.3 | 1.760 | 2.581 | 142.3 | 1.697 | 2.581 | 147.4 |
| U5-d-r-v-j | 1.609 | 2.520 | 149.1 | 1.747 | 2.573 | 142.7 | 1.687 | 2.579 | 148.3 |
| U5-r-x-j | 1.649 | 2.543 | 147.4 | 1.752 | 2.573 | 142.2 | 1.691 | 2.575 | 147.3 |
| H26···O32 | | | | | | | | | |
| **U4** |  |  |  |  |  |  |  |  |  |
| U4-d-ε-r-x-j | 1.694 | 2.575 | 146.2 | 1.814 | 2.622 | 140.9 | 1.727 | 2.604 | 146.6 |
| U4-d-w-x-j | 1.684 | 2.568 | 146.5 | 1.800 | 2.611 | 141.2 | 1.712 | 2.592 | 146.9 |
|  |  |  |  |  |  |  |  |  |  |
| **U5** |  |  |  |  |  |  |  |  |  |
| U5-d-r-x-j | 1.675 | 2.562 | 147.0 | 1.776 | 2.594 | 142.1 | 1.707 | 2.588 | 147.1 |
| U5-d-w-x-j | 1.665 | 2.557 | 147.4 | 1.767 | 2.589 | 142.5 | 1.699 | 2.583 | 147.5 |
| U5-d-r-x-k | 1.627 | 2.534 | 148.8 | 1.769 | 2.592 | 142.5 | 1.704 | 2.593 | 148.0 |
| U5-r-x-j | 1.682 | 2.566 | 146.6 | 1.783 | 2.598 | 141.8 | 1.714 | 2.592 | 146.8 |
| H26···O14 | | | | | | | | | |
| **U7** |  |  |  |  |  |  |  |  |  |
| U7-d-r-ᴧ-χ-α-p | 1.930 | 2.862 | 159.8 | 2.069 | 2.943 | 153.1 | 2.074 | 3.009 | 161.0 |
| U7-d-w-ᴧ-χ-α-p | 1.938 | 2.868 | 159.6 | 2.077 | 2.949 | 152.8 | 2.079 | 3.014 | 160.8 |
| U7-d-w-ᴧ-χ-α-q | 1.943 | 2.871 | 159.1 | 2.074 | 2.945 | 152.7 | 2.106 | 3.050 | 163.1 |
| U7-d-w-ᴧ-χ-β-p | 1.937 | 2.865 | 159.1 | 2.077 | 2.949 | 152.8 | 2.253 | 3.178 | 158.7 |
| U7-d-w-ᴧ-χ-α-p-u | 1.979 | 2.901 | 157.6 | 2.128 | 2.998 | 152.4 | 2.838 | 3.684 | 146.4 |
| U7-d-w-χ-α-p | 1.923 | 2.856 | 160.1 | 2.057 | 2.932 | 153.3 | 1.963 | 2.897 | 161.0 |
| H26···O10 | | | | | | | | | |
| **U8** |  |  |  |  |  |  |  |  |  |
| U8-ƞ-d-u-w-μ-t | 2.535 | 2.852 | 99.1 | 2.514 | 2.812 | 98.4 | 2.439 | 2.815 | 102.7 |
| U8-ƞ-d-u-r-ξ-t | 2.554 | 2.850 | 97.7 | 2.535 | 2.805 | 96.6 | 2.415 | 2.799 | 103.2 |
| U8-ƞ-d-u-y-ς-t | 2.632 | 2.883 | 95.1 | 2.593 | 2.842 | 95.4 | 2.521 | 2.847 | 99.6 |
| H26···O27 | | | | | | | | | |
| **U8** |  |  |  |  |  |  |  |  |  |
| U8-ƞ-d-u-y-κ-t | 2.416 | 2.873 | 108.4 | 2.444 | 2.852 | 105.9 | 2.335 | 2.844 | 112.1 |
| U8-ƞ-d-u-y-δ-ω | 2.429 | 2.880 | 108.0 | 2.451 | 2.857 | 105.8 | 2.351 | 2.854 | 111.6 |
| U8-ƞ-d-u-y-δ-t | 2.430 | 2.881 | 108.0 | 2.452 | 2.857 | 105.8 | 2.350 | 2.854 | 111.6 |
| U8-ƞ-d-u-r-δ-n | 2.424 | 2.877 | 108.2 | 2.441 | 2.852 | 106.1 | 2.348 | 2.853 | 111.8 |
| U8-ƞ-d-u-w-δ-t | 2.428 | 2.879 | 108.0 | 2.446 | 2.853 | 105.8 | 2.351 | 2.854 | 111.6 |
| H28···O25 | | | | | | | | | |
| **U8** |  |  |  |  |  |  |  |  |  |
| U8-ƞ-d-u-w-τ-t | 2.371 | 2.856 | 110.3 | 2.374 | 2.819 | 108.4 | 2.295 | 2.829 | 113.8 |
| U8-ƞ-d-u-w-μ-t | 2.402 | 2.843 | 107.2 | 2.408 | 2.815 | 105.7 | 2.340 | 2.823 | 110.1 |
| U8-ƞ-d-u-y-ς-t | 2.353 | 2.815 | 108.5 | 2.371 | 2.798 | 107.1 | 2.306 | 2.804 | 111.1 |
| H28···O29 | | | | | | | | | |
| **U8** |  |  |  |  |  |  |  |  |  |
| U8-ƞ-d-u-r-ξ-t | 2.183 | 2.697 | 111.7 | 2.209 | 2.679 | 109.6 | 2.134 | 2.675 | 113.7 |
| U8-ƞ-d-u-y-κ-t | 2.209 | 2.724 | 111.9 | 2.217 | 2.699 | 110.6 | 2.159 | 2.700 | 113.7 |
| U8-ƞ-d-u-y-δ-ω | 2.204 | 2.718 | 111.8 | 2.213 | 2.696 | 110.5 | 2.159 | 2.698 | 113.6 |
| U8-ƞ-d-u-y-δ-t | 2.204 | 2.718 | 111.8 | 2.214 | 2.695 | 110.5 | 2.158 | 2.697 | 113.6 |
| U8-ƞ-d-u-r-δ-n | 2.202 | 2.717 | 111.8 | 2.213 | 2.695 | 110.5 | 2.156 | 2.696 | 113.7 |
| U8-ƞ-d-u-w-δ-t | 2.203 | 2.717 | 111.8 | 2.214 | 2.695 | 110.5 | 2.158 | 2.697 | 113.6 |
| H30···O27 | | | | | | | | | |
| **U8** |  |  |  |  |  |  |  |  |  |
| U8-ƞ-d-u-w-τ-t | 2.219 | 2.722 | 111.0 | 2.233 | 2.697 | 109.2 | 2.151 | 2.695 | 114.0 |
| U8-ƞ-d-u-w-μ-t | 2.202 | 2.710 | 111.3 | 2.227 | 2.691 | 109.2 | 2.135 | 2.684 | 114.3 |
| H30···O31 | | | | | | | | | |
| **U8** |  |  |  |  |  |  |  |  |  |
| U8-ƞ-d-u-y-κ-t | 1.935 | 2.781 | 143.8 | 2.022 | 2.798 | 138.0 | 1.891 | 2.746 | 145.1 |
| U8-ƞ-d-u-r-ξ-t | 1.944 | 2.785 | 143.2 | 2.025 | 2.796 | 137.5 | 1.899 | 2.746 | 144.1 |
| U8-ƞ-d-u-y-ς-t | 1.977 | 2.811 | 142.4 | 2.052 | 2.815 | 136.7 | 1.932 | 2.773 | 143.4 |
| U8-ƞ-d-u-y-δ-ω | 1.898 | 2.721 | 140.7 | 1.948 | 2.735 | 139.1 | 1.863 | 2.700 | 142.7 |
| U8-ƞ-d-u-y-δ-t | 1.898 | 2.721 | 140.7 | 1.948 | 2.735 | 139.1 | 1.863 | 2.699 | 142.6 |
| U8-ƞ-d-u-r-δ-n | 1.899 | 2.722 | 140.8 | 1.947 | 2.733 | 139.0 | 1.861 | 2.698 | 142.6 |
| U8-ƞ-d-u-w-δ-t | 1.906 | 2.726 | 140.5 | 1.948 | 2.733 | 138.9 | 1.863 | 2.699 | 142.6 |
| H32···O29 | | | | | | | | | |
| **U8** |  |  |  |  |  |  |  |  |  |
| U8-ƞ-d-u-r-ξ-t | 2.481 | 2.814 | 99.9 | 2.429 | 2.769 | 101.0 | 2.269 | 2.721 | 107.3 |
| U8-ƞ-d-u-y-ς-t | 2.469 | 2.816 | 100.8 | 2.409 | 2.769 | 102.3 | 2.251 | 2.721 | 108.7 |
| U8-ƞ-d-u-y-κ-t | 2.617 | 2.359 | 64.4 | 2.409 | 2.759 | 101.6 | 2.242 | 2.704 | 108.0 |
| U8-ƞ-d-u-w-μ-t | 1.992 | 2.783 | 137.1 | 2.036 | 2.788 | 135.3 | 1.946 | 2.752 | 139.2 |
| U8-ƞ-d-u-w-τ-t | 1.997 | 2.787 | 137.1 | 2.037 | 2.789 | 135.3 | 1.950 | 2.756 | 139.2 |
| H17···π (C13) | | | | | | | | | |
| **U8** |  |  |  |  |  |  |  |  |  |
| U8-ƞ-d-u-y-κ-ω | 2.184 | 2.982 | 138.8 | 2.301 | 3.020 | 132.5 | 2.147 | 2.941 | 138.3 |
| U8-ƞ-d-u-y-κ-t | 2.183 | 2.982 | 139.0 | 2.638 | 2.374 | 64.1 | 2.144 | 2.940 | 138.5 |
| U8-ƞ-d-u-w-μ-t | 2.181 | 2.982 | 139.1 | 2.526 | 2.308 | 66.0 | 2.148 | 2.944 | 138.5 |
| U8-ƞ-d-u-r-ξ-t | 2.183 | 2.983 | 139.0 | 2.522 | 2.308 | 66.2 | 2.152 | 2.945 | 138.1 |
| U8-ƞ-d-u-y-ς-t | 2.183 | 2.983 | 138.9 | 2.572 | 2.342 | 65.6 | 2.158 | 2.946 | 137.6 |
| U8-ƞ-d-u-y-δ-ω | 2.183 | 2.981 | 138.8 | 2.594 | 2.355 | 65.2 | 2.146 | 2.939 | 138.2 |
| U8-ƞ-d-u-y-δ-t | 2.181 | 2.981 | 139.0 | 2.596 | 2.356 | 65.2 | 2.144 | 2.938 | 138.3 |
| U8-ƞ-d-u-r-δ-n | 2.193 | 2.985 | 138.1 | 2.515 | 2.305 | 66.3 | 2.161 | 2.942 | 136.9 |
| U8-ƞ-d-u-w-δ-t | 2.172 | 2.975 | 139.3 | 2.519 | 2.305 | 66.2 | 2.144 | 2.938 | 138.3 |
| U8-ƞ-d-u-w-τ-t | 2.180 | 2.982 | 139.2 | 2.520 | 2.306 | 66.2 | 2.147 | 2.944 | 138.6 |
