# Supplementary material for: Computable properties of selected monomeric acylphloroglucinols with anticancer and/or antimalarial activities and first-approximation docking study
Source: J Mol Model. 2025 Mar 12;31(4):113. doi: 10.1007/s00894-025-06299-7 (PMC11903629; doi:10.1007/s00894-025-06299-7)
Supplement: Supplementary file 19 — (DOCX 17.7 KB) [file 894_2025_6299_MOESM19_ESM.docx]

**Table S5.**

**Energy increase on removal of the first intramolecular hydrogen bond (IHB) from the lowest energy conformer of the ACPL molecules considered in this work.**

Results from full optimisation calculations HF/6-31G(d,p), DFT/B3LYP/6-31+G(d,p) and MP2/ 6-31G(d,p) calculations, respectively denoted as DFT, HF and MP2 in the columns’ headings.

The energy difference is taken as « energy of the lowest energy conformer resulting from the removal of the first IHB minus energy of the lowest energy conformer with the IHB».

| Molecules and conformers | | Energy difference (kcal mol^-1^) | | |
| --- | --- | --- | --- | --- |
| With the IHB | Without the IHB | DFT | HF | MP2 |
| **U1** |  |  |  |  |
| U1-d-r-a | U1-r-a | 14.717 | 15.476 | 12.423 |
|  |  |  |  |  |
| **U2** |  |  |  |  |
| U2-d-v-a | U2-x-a | 16.643 | 14.187 | 13.407 |
|  |  |  |  |  |
| **U3** |  |  |  |  |
| U3-s-x-w-a | U3-v-w-a | 13.739 | 11.128 | 11.869 |
|  |  |  |  |  |
| **U4** |  |  |  |  |
| U4-d-w-v-k | U4-w-v-k | 13.044 | 10.080 | 9.901 |
|  |  |  |  |  |
| **U5** |  |  |  |  |
| U5-d-r-x-j | U5-r-x-j | 13.528 | 10.372 | 10.671 |
|  |  |  |  |  |
| **U6** |  |  |  |  |
| U6-d-w-e | U6-w-f | 15.142 | 11.821 | 12.158 |
|  |  |  |  |  |
| **U7** |  |  |  |  |
| U7-d-r-ᴧ-χ-α-p | U7-w-ᴧ-χ-α-p | 14.314 | 11.687 | 9.434 |
|  |  |  |  |  |
| **U8** |  |  |  |  |
| U8-ƞ-d-u-y-κ-ω | U8-y-κ-ω | 11.949 | 6.542 | 10.077 |
