# Supplementary material for: Computable properties of selected monomeric acylphloroglucinols with anticancer and/or antimalarial activities and first-approximation docking study
Source: J Mol Model. 2025 Mar 12;31(4):113. doi: 10.1007/s00894-025-06299-7 (PMC11903629; doi:10.1007/s00894-025-06299-7)
Supplement: Supplementary file 21 — (DOCX 31.5 KB) [file 894_2025_6299_MOESM21_ESM.docx]

**Table S7**

**Vibrational frequencies (harmonic approximation, stretching mode) of the O–H bonds of the calculated conformers of the considered ACPL molecules.**

HF/6-31G(d,p) results *in vacuo*. The molecules are denoted with the symbols listed in table 1, and the conformers with the symbols listed in table 2. For each molecule, the conformers are listed in order of increasing relative energies in the DFT results. The computed frequencies have been scaled by 0.8992.

# a) Vibrational frequencies of the OH groups in the calculated conformers of thouvenol A

| Molecules and conformers | Vibrational frequency (cm^-1^) | | |
| --- | --- | --- | --- |
|  | O12–H17 | O10–H16 | O8–H15 |
| U1-d-r-a | 3462.62 | 3762.96 | 3763.33 |
| U1-d-w-a | 3478.61 | 3771.08 | 3767.41 |
| U1-d-u-r-a | 3512.63 | 3763.89 | 3828.25 |
| U1-d-u-w-a | 3529.02 | 3766.69 | 3830.18 |
| U1-r-a | 3769.38 | 3768.14 | 3767.43 |

# b) Vibrational frequencies of the OH groups in the calculated conformers of myristicyclin A

| Molecules and conformers | Vibrational frequency (cm^-1^) | | |
| --- | --- | --- | --- |
|  | O12–H17 | O10–H16 | O22–H23 |
| U2-d-v-a | 3430.12 | 3763.45 | 3773.82 |
| U2-s-v-a | 3809.69 | 3488.91 | 3773.17 |
| U2-s-v-u-a | 3821.06 | 3548.27 | 3774.30 |
| U2-d-x-a | 3431.96 | 3763.80 | 3773.03 |
| U2-x-a | 3772.45 | 3805.77 | 3771.58 |

# c) Vibrational frequencies of the OH groups in the calculated conformers of myristicyclin B

| Molecules and conformers | Vibrational frequency (cm^-1^) | | |
| --- | --- | --- | --- |
|  | O12–H17 | O10–H16 | O22–H23 |
| U3-s-x-w-a | 3471.68 | 3763.83 | 3772.60 |
| U3-s-v-w-a | 3470.23 | 3763.88 | 3773.83 |
| U3-s-x-w-b | 3450.47 | 3763.50 | 3772.67 |
| U3-s-x-r-a | 3490.61 | 3811.83 | 3771.42 |
| U3-z-x-w | 3777.04 | 3773.49 | 3772.37 |
| U3-v-w-a | 3777.31 | 3773.39 | 3774.20 |

# d) Vibrational frequencies of the OH groups in the calculated conformers of knipholone

| Molecules and conformers | Vibrational frequency (cm^-1^) | | | |
| --- | --- | --- | --- | --- |
|  | O12–H17 | O10–H16 | O22–H23 | O25–H26 |
| U4-d-ε-r-x-j | 3411.74 | 3747.99 | 3580.36 | 3604.60 |
| U4-d-w-x-j | 3431.19 | 3772.61 | 3579.23 | 3595.93 |
| U4-d-ε-r-v-j | 3416.02 | 3743.82 | 3568.40 | 3767.59 |
| U4-d-ε-r-x-k | 3412.00 | 3721.73 | 3765.20 | 3584.72 |
| U4-d-w-v-k | 3432.18 | 3773.32 | 3772.01 | 3772.42 |
| U4-w-v-k | 3748.54 | 3775.42 | 3748.54 | 3771.43 |

# e) Vibrational frequencies of the OH groups in the calculated conformers of knipholoneanthrone

| Molecules and conformers | Vibrational frequency (cm^-1^) | | | |
| --- | --- | --- | --- | --- |
|  | O12–H17 | O10–H16 | O22–H23 | O25–H26 |
| U5-d-r-x-j | 3414.14 | 3715.78 | 3556.26 | 3579.88 |
| U5-d-w-x-j | 3429.18 | 3769.88 | 3560.10 | 3573.51 |
| U5-d-r-v-j | 3418.79 | 3711.00 | 3525.11 | 3766.39 |
| U5-d-r-x-k | 3414.55 | 3716.96 | 3764.82 | 3548.20 |
| U5-r-x-j | 3740.02 | 3736.96 | 3545.41 | 3591.22 |
| U5-d-w-v-k | 3434.41 | 3771.12 | 3772.59 | 3772.36 |

# f) Vibrational frequencies of the OH groups in the calculated conformers of 1-(2,6-dihydroxy-3-methyl-4-((3-methylbut-2-en-1-yl)oxy)phenyl)-3-methylbutan-1-one

| Molecules and conformers | Vibrational frequency (cm^-1^) | |
| --- | --- | --- |
|  | O12–H17 | O8–H15 |
| U6-d-w-e | 3482.44 | 3769.28 |
| U6-d-w-g | 3460.44 | 3770.53 |
| U6-d-w-c | 3460.69 | 3770.55 |
| U6-s-w-f | 3784.45 | 3480.39 |
| U6-d-w-e-u | 3536.53 | 3563.62 |
| U6-d-w-f | 3476.70 | 3769.04 |
| U6-d-w-h | 3445.02 | 3770.07 |
| U6-d-y-f | 3472.76 | 3765.46 |
| U6-d-m-f | 3473.72 | 3765.35 |
| U6-w-f | 3788.60 | 3778.00 |

# g) Vibrational frequencies of the OH groups in the calculated conformers of antiarone J

| Molecules and conformers | Vibrational frequency (cm^-1^) | | | | |
| --- | --- | --- | --- | --- | --- |
|  | O12–H17 | O10–H16 | O8–H15 | O22–H23 | O25–H26 |
| U7-d-r-ᴧ-χ-α-p | 3498.80 | 3761.97 | 3762.30 | 3737.89 | 3735.59 |
| U7-d-w-ᴧ-χ-α-p | 3516.48 | 3770.05 | 3766.40 | 3737.93 | 3735.01 |
| U7-d-w-ᴧ-χ-α-q | 3516.27 | 3770.09 | 3766.41 | 3739.36 | 3735.56 |
| U7-d-w-ᴧ-χ-β-p | 3516.76 | 3769.99 | 3766.21 | 3738.55 | 3734.94 |
| U7-d-w-χ-α-p | 3761.97 | 3770.92 | 3767.85 | 3737.39 | 3747.35 |
| U7-d-w-ᴧ-χ-α-p-u | 3563.62 | 3765.81 | 3808.32 | 3736.46 | 3718.09 |
| U7-d-w-ᴧ-λ-α-q | 3520.48 | 3769.82 | 3765.88 | 3782.11 | 3732.46 |
| U7-d-w-ᴧ-λ-α-p | 3520.15 | 3769.76 | 3765.85 | 3782.16 | 3732.07 |
| U7-d-w-γ-χ-p | 3494.53 | 3771.15 | 3767.16 | 3738.96 | 3773.08 |
| U7-w-ᴧ-χ-α-p | 3771.80 | 3770.35 | 3770.92 | 3738.31 | 3690.18 |

# h) Vibrational frequencies of the OH groups in the calculated conformers of iriflophenone4-glucoside

| Molecules and conformers | Vibrational frequency (cm^-1^) | | | | | | |
| --- | --- | --- | --- | --- | --- | --- | --- |
|  | O12–H17 | O8–H15 | O22–H23 | O25–H26 | O27–H28 | O29–H30 | O31–H32 |
| U8-ƞ-d-u-y-κ-ω | 3621.54 | 3768.92 | 3769.28 | 3768.92 | 3722.59 | 3712.65 | 3735.85 |
| U8-ƞ-d-u-y-κ-t | 3625.06 | 3764.12 | 3767.40 | 3744.41 | 3722.11 | 3709.44 | 3734.21 |
| U8-ƞ-d-u-w-μ-t | 3616.76 | 3760.53 | 3767.53 | 3752.92 | 3741.87 | 3733.06 | 3743.66 |
| U8-d-y-κ-ω | 3621.54 | 3768.92 | 3769.28 | 3744.18 | 3722.59 | 3621.54 | 3735.85 |
| U8-ƞ-d-u-r-ξ-t | 3599.34 | 3763.38 | 3767.25 | 3761.34 | 3734.09 | 3714.25 | 3735.58 |
| U8-ƞ-d-u-y-ς-t | 3618.94 | 3762.56 | 3766.96 | 3758.32 | 3745.78 | 3727.53 | 3737.47 |
| U8-ƞ-d-u-y-δ-ω | 3628.42 | 3765.10 | 3767.05 | 3745.26 | 3722.25 | 3719.68 | 3778.70 |
| U8-ƞ-d-u-y-δ-t | 3629.11 | 3766.08 | 3767.84 | 3745.45 | 3719.76 | 3722.26 | 3778.94 |
| U8-ƞ-d-u-r-δ-n | 3597.97 | 3766.79 | 3768.40 | 3744.66 | 3721.70 | 3718.98 | 3779.11 |
| U8-ƞ-d-u-w-δ-t | 3620.06 | 3763.58 | 3767.62 | 3745.35 | 3721.94 | 3719.51 | 3778.90 |
| U8-ƞ-d-u-w-τ-t | 3620.21 | 3760.06 | 3767.54 | 3754.85 | 3734.64 | 3732.07 | 3743.18 |
| U8-y-κ-ω | 3775.56 | 3772.14 | 3770.07 | 3742.62 | 3721.88 | 3711.86 | 3735.01 |
