# Supplementary material for: Computable properties of selected monomeric acylphloroglucinols with anticancer and/or antimalarial activities and first-approximation docking study
Source: J Mol Model. 2025 Mar 12;31(4):113. doi: 10.1007/s00894-025-06299-7 (PMC11903629; doi:10.1007/s00894-025-06299-7)
Supplement: Supplementary file 22 — (DOCX 29.6 KB) [file 894_2025_6299_MOESM22_ESM.docx]

**Table S8**

**Red-shifts in the calculated vibrational frequencies (harmonic approximation) of the O–H bonds that act as IHB donors in the calculated conformers of the considered ACPL molecules.**

DFT/B3LYP/6-31+G(d,p) results *in vacuo*. The molecules are denoted with the symbols listed in table 1, and the conformers with the symbols listed in table 2. For each molecule, the conformers are listed in order of increasing relative energies in the DFT results.

# a) Red-shifts in the calculated vibrational frequencies of the O12–H17 bonds forming H17⋅⋅⋅O14 IHB in the calculated conformers of considered ACPL molecules.

| Donor OH | Molecules and conformers | IHB considered | Redshift (cm^-1^) |
| --- | --- | --- | --- |
|  | U1 |  |  |
| O12–H17 | U1-d-r-a | H17⋅⋅⋅O14 | 755.20 |
| O12–H17 | U1-d-w-a | H17⋅⋅⋅O14 | 719.56 |
| O12–H17 | U1-d-u-r-a | H17⋅⋅⋅O14 | 674.92 |
| O12–H17 | U1-d-u-w-a | H17⋅⋅⋅O14 | 674.99 |
|  |  |  | |
|  | U2 |  |  |
| O8–H15 | U2-d-v-a | H15⋅⋅⋅O14 | 806.20 |
| O12–H17 | U2-s-v-a | H17⋅⋅⋅O14 | 701.82 |
| O12–H17 | U2-s-v-u-a | H17⋅⋅⋅O14 | 802.49 |
| O8–H15 | U2-d-x-a | H15⋅⋅⋅O14 | 799.89 |
|  |  |  | |
|  | U3 |  |  |
| O12–H17 | U3-s-x-w-a | H17⋅⋅⋅O14 | 754.58 |
| O12–H17 | U3-s-v-w-a | H17⋅⋅⋅O14 | 761.40 |
| O12–H17 | U3-s-x-w-b | H17⋅⋅⋅O14 | 804.83 |
| O12–H17 | U3-s-x-r-a | H17⋅⋅⋅O14 | 721.40 |
|  |  |  | |
|  | U4 |  |  |
| O8–H15 | U4-d-ε-r-x-j | H15⋅⋅⋅O14 | 783.14 |
| O8–H15 | U4-d-w-x-j | H15⋅⋅⋅O14 | 748.68 |
| O8–H15 | U4-d-ε-r-v-j | H15⋅⋅⋅O14 | 775.54 |
| O8–H15 | U4-d-ε-r-x-k | H15⋅⋅⋅O14 | 786.17 |
| O8–H15 | U4-d-w-v-k | H15⋅⋅⋅O14 | 747.69 |
|  |  |  |  |
|  | U5 |  |  |
| O8–H15 | U5-d-r-x-j | H15⋅⋅⋅O14 | 779.67 |
| O8–H15 | U5-d-w-x-j | H15⋅⋅⋅O14 | 749.03 |
| O8–H15 | U5-d-r-v-j | H15⋅⋅⋅O14 | 769.47 |
| O8–H15 | U5-d-r-x-k | H15⋅⋅⋅O14 | 773.50 |
| O8–H15 | U5-d-w-v-k | H15⋅⋅⋅O14 | 730.52 |
|  |  |  |  |
|  | U6 |  |  |
| O8–H15 | U6-d-w-e | H15⋅⋅⋅O14 | 705.90 |
| O8–H15 | U6-d-w-g | H15⋅⋅⋅O14 | 760.32 |
| O8–H15 | U6-d-w-c | H15⋅⋅⋅O14 | 758.33 |
| O12–H17 | U6-s-w-f | H17⋅⋅⋅O14 | 725.83 |
| O8–H15 | U6-d-w-e-u | H15⋅⋅⋅O14 | 629.13 |
| O8–H15 | U6-d-w-f | H15⋅⋅⋅O14 | 714.23 |
| O8–H15 | U6-d-w-h | H15⋅⋅⋅O14 | 789.35 |
| O8–H15 | U6-d-y-f | H15⋅⋅⋅O14 | 727.20 |
| O8–H15 | U6-d-m-f | H15⋅⋅⋅O14 | 730.73 |
|  |  |  |  |
|  | U7 |  |  |
| O12–H17 | U7-d-r-ᴧ-χ-α-p | H17⋅⋅⋅O14 | 673.24 |
| O12–H17 | U7-d-w-ᴧ-χ-α-p | H17⋅⋅⋅O14 | 643.19 |
| O12–H17 | U7-d-w-ᴧ-χ-α-q | H17⋅⋅⋅O14 | 640.96 |
| O12–H17 | U7-d-w-ᴧ-χ-β-p | H17⋅⋅⋅O14 | 640.50 |
| O12–H17 | U7-d-w-χ-α-p | H17⋅⋅⋅O14 | 724.94 |
| O12–H17 | U7-d-w-ᴧ-χ-α-p-u | H17⋅⋅⋅O14 | 575.69 |
| O12–H17 | U7-d-w-ᴧ-λ-α-q | H17⋅⋅⋅O14 | 632.81 |
| O12–H17 | U7-d-w-ᴧ-λ-α-p | H17⋅⋅⋅O14 | 635.08 |
| O12–H17 | U7-d-w-γ-χ-p | H17⋅⋅⋅O14 | 698.61 |
|  |  |  |  |
|  | U8 |  |  |
| O12–H17 | U8-ƞ-d-u-y-κ-ω | H17⋅⋅⋅O14 | 575.74 |
| O12–H17 | U8-ƞ-d-u-y-κ-t | H17⋅⋅⋅O14 | 575.72 |
| O12–H17 | U8-ƞ-d-u-w-μ-t | H17⋅⋅⋅O14 | 524.45 |
| O12–H17 | U8-d-y-κ-ω | H17⋅⋅⋅O14 | 598.79 |
| O12–H17 | U8-ƞ-d-u-r-ξ-t | H17⋅⋅⋅O14 | 576.34 |
| O12–H17 | U8-ƞ-d-u-y-ς-t | H17⋅⋅⋅O14 | 573.63 |
| O12–H17 | U8-ƞ-d-u-y-δ-ω | H17⋅⋅⋅O14 | 579.37 |
| O12–H17 | U8-ƞ-d-u-y-δ-t | H17⋅⋅⋅O14 | 579.39 |
| O12–H17 | U8-ƞ-d-u-r-δ-n | H17⋅⋅⋅O14 | 572.33 |
| O12–H17 | U8-ƞ-d-u-w-δ-t | H17⋅⋅⋅O14 | 521.20 |
| O12–H17 | U8-ƞ-d-u-w-τ-t | H17⋅⋅⋅O14 | 515.89 |

# b) Red-shifts in the calculated vibrational frequencies of the O22–H23 and O25–H26 bonds that act as IHB donors in the calculated conformers of considered ACPL molecules.

| Molecules and conformers | O22–H23 | | O25–H26 | |
| --- | --- | --- | --- | --- |
|  | IHB considered | Redshift (cm^-1^) | IHB considered | Redshift (cm^-1^) |
| U4 |  |  |  |  |
| U4-d-ε-r-x-j | H23···O32 | 482.45 | H26···O32 | 415.40 |
| U4-d-w-x-j | H23···O32 | 488.07 | H26···O32 | 432.28 |
| U4-d-ε-r-v-j | H23···O32 | 603.90 |  |  |
| U4-d-ε-r-x-k |  |  | H26···O32 | 541.73 |
|  |  |  |  |  |
| U5 |  |  |  |  |
| U5-d-r-x-j | H23···O32 | 490.07 | H26···O32 | 438.51 |
| U5-d-w-x-j | H23···O32 | 449.35 | H26···O32 | 488.32 |
| U5-d-r-v-j |  | 635.64 |  |  |
| U5-d-r-x-k |  |  | H26···O32 | 589.70 |
| U5-r-x-j | H23···O32 | 506.38 | H26···O32 | 508.10 |
|  |  |  |  |  |
| U7 |  |  |  |  |
| U7-d-r-ᴧ-χ-α-p | H23···O24 | 83.71 | H26···O14 | 90.30 |
| U7-d-w-ᴧ-χ-α-p | H23···O24 | 83.60 | H26···O14 | 89.16 |
| U7-d-w-ᴧ-χ-α-q | H23···O24 | 79.70 | H26···O14 | 88.46 |
| U7-d-w-ᴧ-χ-β-p | H23···O24 | 80.01 | H26···O14 | 88.60 |
| U7-d-w-χ-α-p | H23···O24 | 85.82 |  |  |
| U7-d-w-ᴧ-χ-α-p-u | H23···O24 | 86.26 | H26···O14 | 86.41 |
| U7-d-w-ᴧ-λ-α-q |  |  | H26···O14 | 99.20 |
| U7-d-w-ᴧ-λ-α-p |  |  |  | 104.20 |
| U7-d-w-γ-χ-p | H23···O24 | 79.46 |  |  |
| U7-w-ᴧ-χ-α-p | H23···O24 | 84.38 | H26···O14 | 147.25 |
