# Supplementary material for: Computable properties of selected monomeric acylphloroglucinols with anticancer and/or antimalarial activities and first-approximation docking study
Source: J Mol Model. 2025 Mar 12;31(4):113. doi: 10.1007/s00894-025-06299-7 (PMC11903629; doi:10.1007/s00894-025-06299-7)
Supplement: Supplementary file 23 — (DOCX 28.5 KB) [file 894_2025_6299_MOESM23_ESM.docx]

**Table S9**

**Red-shifts in the calculated vibrational frequencies (harmonic approximation) of the O–H bonds that act as IHB donors in the calculated conformers of the considered ACPL molecules.**

HF/6-31G(d,p) results *in vacuo*. The molecules are denoted with the symbols listed in table 1, and the conformers with the symbols listed in table 2.

# a) Red-shifts in the calculated vibrational frequencies of the O12–H17 bonds forming H17⋅⋅⋅O14 IHB in the calculated conformers of considered ACPL molecules.

| Donor OH | Molecules and conformers | IHB considered | Redshift (cm^-1^) |
| --- | --- | --- | --- |
|  | U1 |  |  |
| O12–H17 | U1-d-r-a | H17⋅⋅⋅O14 | 306.78 |
| O12–H17 | U1-d-w-a | H17⋅⋅⋅O14 | 290.79 |
| O12–H17 | U1-d-u-r-a | H17⋅⋅⋅O14 | 256.77 |
| O12–H17 | U1-d-u-w-a | H17⋅⋅⋅O14 | 240.38 |
|  |  |  | |
|  | U2 |  |  |
| O8–H15 | U2-d-v-a | H15⋅⋅⋅O14 | 342.33 |
| O12–H17 | U2-s-v-a | H17⋅⋅⋅O14 | 295.69 |
| O12–H17 | U2-s-v-u-a | H17⋅⋅⋅O14 | 236.34 |
| O8–H15 | U2-d-x-a | H15⋅⋅⋅O14 | 340.49 |
|  |  |  | |
|  | U3 |  |  |
| O12–H17 | U3-s-x-w-a | H17⋅⋅⋅O14 | 305.55 |
| O12–H17 | U3-s-v-w-a | H17⋅⋅⋅O14 | 307.00 |
| O12–H17 | U3-s-x-w-b | H17⋅⋅⋅O14 | 326.76 |
| O12–H17 | U3-s-x-r-a | H17⋅⋅⋅O14 | 286.62 |
|  |  |  | |
|  | U4 |  |  |
| O8–H15 | U4-d-ε-r-x-j | H15⋅⋅⋅O14 | 336.80 |
| O8–H15 | U4-d-w-x-j | H15⋅⋅⋅O14 | 317.35 |
| O8–H15 | U4-d-ε-r-v-j | H15⋅⋅⋅O14 | 332.52 |
| O8–H15 | U4-d-ε-r-x-k | H15⋅⋅⋅O14 | 336.54 |
| O8–H15 | U4-d-w-v-k | H15⋅⋅⋅O14 | 316.36 |
|  |  |  |  |
|  | U5 |  |  |
| O8–H15 | U5-d-r-x-j | H15⋅⋅⋅O14 | 325.88 |
| O8–H15 | U5-d-w-x-j | H15⋅⋅⋅O14 | 310.84 |
| O8–H15 | U5-d-r-v-j | H15⋅⋅⋅O14 | 321.23 |
| O8–H15 | U5-d-r-x-k | H15⋅⋅⋅O14 | 325.47 |
| O8–H15 | U5-d-w-v-k | H15⋅⋅⋅O14 | 305.61 |
|  |  |  |  |
|  | U6 |  |  |
| O8–H15 | U6-d-w-e | H15⋅⋅⋅O14 | 304.09 |
| O8–H15 | U6-d-w-g | H15⋅⋅⋅O14 | 326.09 |
| O8–H15 | U6-d-w-c | H15⋅⋅⋅O14 | 325.84 |
| O12–H17 | U6-s-w-f | H17⋅⋅⋅O14 | 293.25 |
| O8–H15 | U6-d-w-e-u | H15⋅⋅⋅O14 | 250.00 |
| O8–H15 | U6-d-w-f | H15⋅⋅⋅O14 | 309.83 |
| O8–H15 | U6-d-w-h | H15⋅⋅⋅O14 | 341.51 |
| O8–H15 | U6-d-y-f | H15⋅⋅⋅O14 | 313.77 |
| O8–H15 | U6-d-m-f | H15⋅⋅⋅O14 | 312.81 |
|  |  |  |  |
|  | U7 |  |  |
| O12–H17 | U7-d-r-ᴧ-χ-α-p | H17⋅⋅⋅O14 | 268.08 |
| O12–H17 | U7-d-w-ᴧ-χ-α-p | H17⋅⋅⋅O14 | 250.40 |
| O12–H17 | U7-d-w-ᴧ-χ-α-q | H17⋅⋅⋅O14 | 250.61 |
| O12–H17 | U7-d-w-ᴧ-χ-β-p | H17⋅⋅⋅O14 | 250.12 |
| O12–H17 | U7-d-w-χ-α-p | H17⋅⋅⋅O14 | 271.39 |
| O12–H17 | U7-d-w-ᴧ-χ-α-p-u | H17⋅⋅⋅O14 | 203.26 |
| O12–H17 | U7-d-w-ᴧ-λ-α-q | H17⋅⋅⋅O14 | 246.40 |
| O12–H17 | U7-d-w-ᴧ-λ-α-p | H17⋅⋅⋅O14 | 246.73 |
| O12–H17 | U7-d-w-γ-χ-p | H17⋅⋅⋅O14 | 272.35 |
|  |  |  |  |
|  | U8 |  |  |
| O12–H17 | U8-ƞ-d-u-y-κ-ω | H17⋅⋅⋅O14 | 154.02 |
| O12–H17 | U8-ƞ-d-u-y-κ-t | H17⋅⋅⋅O14 | 150.50 |
| O12–H17 | U8-ƞ-d-u-w-μ-t | H17⋅⋅⋅O14 | 158.80 |
| O12–H17 | U8-d-y-κ-ω | H17⋅⋅⋅O14 | 154.02 |
| O12–H17 | U8-ƞ-d-u-r-ξ-t | H17⋅⋅⋅O14 | 176.22 |
| O12–H17 | U8-ƞ-d-u-y-ς-t | H17⋅⋅⋅O14 | 156.62 |
| O12–H17 | U8-ƞ-d-u-y-δ-ω | H17⋅⋅⋅O14 | 147.14 |
| O12–H17 | U8-ƞ-d-u-y-δ-t | H17⋅⋅⋅O14 | 146.45 |
| O12–H17 | U8-ƞ-d-u-r-δ-n | H17⋅⋅⋅O14 | 177.59 |
| O12–H17 | U8-ƞ-d-u-w-δ-t | H17⋅⋅⋅O14 | 155.50 |
| O12–H17 | U8-ƞ-d-u-w-τ-t | H17⋅⋅⋅O14 | 155.35 |

# b) Red-shifts in the calculated vibrational frequencies of the O22–H23 and O25–H26 bonds that act as IHB donors in the calculated conformers of considered ACPL molecules.

| Molecules and conformers | O22–H23 | | O25–H26 | |
| --- | --- | --- | --- | --- |
|  | IHB considered | Redshift (cm^-1^) | IHB considered | Redshift (cm^-1^) |
| U4 |  |  |  |  |
| U4-d-ε-r-x-j | H23···O32 | 179.91 | H26···O32 | 164.91 |
| U4-d-w-x-j | H23···O32 | 181.04 | H26···O32 | 173.58 |
| U4-d-ε-r-v-j | H23···O32 | 191.87 |  |  |
| U4-d-ε-r-x-k |  |  | H26···O32 | 184.79 |
|  |  |  |  |  |
| U5 |  |  |  |  |
| U5-d-r-x-j | H23···O32 | 212.45 | H26···O32 | 189.49 |
| U5-d-w-x-j | H23···O32 | 208.61 | H26···O32 | 195.86 |
| U5-d-r-v-j |  | 243.60 |  |  |
| U5-d-r-x-k |  |  | H26···O32 | 221.17 |
| U5-r-x-j | H23···O32 | 223.30 | H26···O32 | 178.15 |
|  |  |  |  |  |
| U7 |  |  |  |  |
| U7-d-r-ᴧ-χ-α-p | H23···O24 | 44.24 | H26···O14 | 37.49 |
| U7-d-w-ᴧ-χ-α-p | H23···O24 | 44.20 | H26···O14 | 38.07 |
| U7-d-w-ᴧ-χ-α-q | H23···O24 | 42.77 | H26···O14 | 37.52 |
| U7-d-w-ᴧ-χ-β-p | H23···O24 | 43.58 | H26···O14 | 38.14 |
| U7-d-w-χ-α-p | H23···O24 | 44.74 |  |  |
| U7-d-w-ᴧ-χ-α-p-u | H23···O24 | 45.67 | H26···O14 | 54.99 |
| U7-d-w-ᴧ-λ-α-q |  |  | H26···O14 | 40.62 |
| U7-d-w-ᴧ-λ-α-p |  |  |  | 41.01 |
| U7-d-w-γ-χ-p | H23···O24 | 43.17 |  |  |
| U7-w-ᴧ-χ-α-p | H23···O24 | 43.82 | H26···O14 | 82.90 |
