# Supplementary material for: Computable properties of selected monomeric acylphloroglucinols with anticancer and/or antimalarial activities and first-approximation docking study
Source: J Mol Model. 2025 Mar 12;31(4):113. doi: 10.1007/s00894-025-06299-7 (PMC11903629; doi:10.1007/s00894-025-06299-7)
Supplement: Supplementary file 25 — (DOCX 21.9 KB) [file 894_2025_6299_MOESM25_ESM.docx]

**Table S11.**

**Dipole moments of the calculated conformers of the considered ACPL molecules.**

DFT/B3LYP/6-31+G(d,p), HF/6-31G(d,p) and MP2/6-31G(d,p) results *in vacuo*, from full optimisation calculations, respectively denoted as DFT, HF and MP2 in the columns’ headings. For each molecule, the conformers are listed in order of increasing relative energies in the DFT results.

| Molecules and conformers | Dipole moment (debye) | | |
| --- | --- | --- | --- |
|  | DFT | HF | MP2 |
| **U1** |  |  |  |
| U1-d-r-a | 3.786 | 3.618 | 3.845 |
| U1-d-w-a | 6.135 | 6.073 | 6.380 |
| U1-d-u-r-a | 1.612 | 1.353 | 1.465 |
| U1-d-u-w-a | 4.304 | 4.205 | 4.395 |
| U1-r-a | 4.625 | 5.253 | 5.142 |
|  |  |  |  |
| **U2** |  |  |  |
| U2-d-v-a | 1.845 | 1.770 | 2.058 |
| U2-s-v-a | 6.671 | 6.774 | 7.278 |
| U2-s-v-u-a | 5.847 | 5.860 | 6.211 |
| U2-d-x-a | 4.113 | 4.021 | 4.203 |
| U2-x-a | 4.724 | 4.793 | 5.956 |
|  |  |  |  |
| **U3** |  |  |  |
| U3-s-x-w-a | 3.693 | 3.474 | 3.842 |
| U3-s-v-w-a | 4.696 | 4.764 | 5.098 |
| U3-s-x-w-b | 3.927 | 3.686 | 4.019 |
| U3-s-x-r-a | 6.253 | 6.154 | 6.558 |
| U3-z-x-w | 3.385 | 3.896 | 4.323 |
| U3-v-w-a | 6.024 | 6.650 | 7.088 |
|  |  |  |  |
| **U4** |  |  |  |
| U4-d-ε-r-x-j | 4.430 | 4.289 | 5.392 |
| U4-d-w-x-j | 7.767 | 7.666 | 8.549 |
| U4-d-ε-r-v-j | 4.391 | 4.304 | 5.979 |
| U4-d-ε-r-x-k | 5.254 | 4.986 | 6.628 |
| U4-d-w-v-k | 5.324 | 5.532 | 5.947 |
| U4-w-v-k | 3.099 | 2.702 | 3.975 |
|  |  |  |  |
| **U5** |  |  |  |
| U5-d-r-x-j | 7.600 | 7.381 | 8.572 |
| U5-d-w-x-j | 11.072 | 10.949 | 12.255 |
| U5-d-r-v-j | 7.335 | 7.483 | 8.507 |
| U5-d-r-x-k | 8.327 | 8.157 | 9.415 |
| U5-r-x-j | 3.404 | 3.702 | 4.139 |
| U5-d-w-v-k | 10.501 | 10.777 | 11.995 |
|  |  |  |  |
| **U6** |  |  |  |
| U6-d-w-e | 6.654 | 6.528 | 6.969 |
| U6-d-w-g | 6.978 | 6.780 | 7.252 |
| U6-d-w-c | 6.951 | 6.757 | 7.222 |
| U6-s-w-f | 5.466 | 4.927 | 5.293 |
| U6-d-w-e-u | 4.002 | 3.996 | 4.379 |
| U6-d-w-f | 6.760 | 6.610 | 7.046 |
| U6-d-w-h | 6.837 | 6.680 | 7.099 |
| U6-d-y-f | 5.139 | 5.184 | 5.520 |
| U6-d-m-f | 5.254 | 5.384 | 5.568 |
| U6-w-f | 6.266 | 6.284 | 6.705 |
|  |  |  |  |
| **U7** |  |  |  |
| U7-d-r-ᴧ-χ-α-p | 5.061 | 4.556 | 5.213 |
| U7-d-w-ᴧ-χ-α-p | 6.764 | 6.064 | 6.349 |
| U7-d-w-ᴧ-χ-α-q | 7.808 | 7.152 | 7.978 |
| U7-d-w-ᴧ-χ-β-p | 6.757 | 6.001 | 8.394 |
| U7-d-w-χ-α-p | 4.956 | 5.185 | 5.674 |
| U7-d-w-ᴧ-χ-α-p-u | 4.908 | 3.829 | 2.243 |
| U7-d-w-ᴧ-λ-α-q | 6.203 | 5.847 | 6.130 |
| U7-d-w-ᴧ-λ-α-p | 4.792 | 3.882 | 3.961 |
| U7-d-w-γ-χ-p | 5.954 | 6.061 | 6.210 |
| U7-w-ᴧ-χ-α-p | 7.287 | 7.092 | 8.809 |
|  |  |  |  |
| **U8** |  |  |  |
| U8-ƞ-d-u-y-κ-ω | 1.830 | 3.624 | 7.320 |
| U8-ƞ-d-u-y-κ-t | 2.939 | 5.022 | 7.196 |
| U8-ƞ-d-u-w-μ-t | 4.729 | 4.347 | 4.825 |
| U8-d-y-κ-ω | 1.454 | 4.414 | 5.182 |
| U8-ƞ-d-u-r-ξ-t | 5.659 | 5.406 | 5.901 |
| U8-ƞ-d-u-y-ς-t | 6.050 | 6.317 | 6.885 |
| U8-ƞ-d-u-y-δ-ω | 1.191 | 5.762 | 10.001 |
| U8-ƞ-d-u-y-δ-t | 3.597 | 7.100 | 9.339 |
| U8-ƞ-d-u-r-δ-n | 5.054 | 4.897 | 4.707 |
| U8-ƞ-d-u-w-δ-t | 8.507 | 8.297 | 9.338 |
| U8-ƞ-s-u-w-τ-t | 6.398 | 6.075 | 6.726 |
| U8-y-κ-ω | 1.610 | 2.012 | 3.278 |
