# Supplementary material for: Computable properties of selected monomeric acylphloroglucinols with anticancer and/or antimalarial activities and first-approximation docking study
Source: J Mol Model. 2025 Mar 12;31(4):113. doi: 10.1007/s00894-025-06299-7 (PMC11903629; doi:10.1007/s00894-025-06299-7)
Supplement: Supplementary file 26 — (DOCX 23.7 KB) [file 894_2025_6299_MOESM26_ESM.docx]

**Table S12.**

**Relative energies of the calculated conformers of the considered ACPL molecules *in vacuo*, in chloroform, in acetonitrile and in water (respectively denoted as vac, chlrf, actn and aq in the column headings).**

DFT/B3LYP/6-31+G(d,p results from full optimisation calculations. For each molecule, the conformers are listed in order of increasing relative energies in the DFT results *in vacuo*.

| Molecules and conformers | Relative energy (kcal mol^-1^) | | | |
| --- | --- | --- | --- | --- |
|  | vac | chlrf | actn | aq |
| **U1** |  |  |  |  |
| U1-d-r-a | 0.000 | 0.000 | 0.000 | 0.000 |
| U1-d-w-a | 1.380 | 0.635 | 0.250 | 0.219 |
| U1-d-u-r-a | 2.879 | 2.963 | 2.997 | 3.001 |
| U1-d-u-w-a | 3.229 | 3.124 | 3.070 | 3.065 |
| U1-r-a | 14.717 | 11.173 | 10.125 | 10.034 |
|  |  |  |  |  |
| **U2** |  |  |  |  |
| U2-d-v-a | 0.000 | 0.000 | 0.000 | 0.000 |
| U2-s-v-a | 0.080 | 3.107 | 2.641 | 3.495 |
| U2-s-v-u-a | 4.178 | 3.775 | 3.520 | 3.495 |
| U2-d-x-a | 4.327 | 0.086 | 0.089 | 0.089 |
| U2-x-a | 16.643 | 12.939 | 11.883 | 11.789 |
|  |  |  |  |  |
| **U3** |  |  |  |  |
| U3-s-x-w-a | 0.000 | 0.000 | 0.676 | 0.000 |
| U3-s-v-w-a | 0.030 | 0.000 | 0.000 | 0.000 |
| U3-s-x-w-b | 0.534 | 0.422 | 1.058 | 0.379 |
| U3-s-x-r-a | 3.135 | 2.460 | 2.860 | 2.157 |
| U3-z-x-w | 13.628 | 10.352 | 10.081 | 9.322 |
| U3-v-w-a | 13.739 | 10.401 | 9.429 | 9.344 |
|  |  |  |  |  |
| **U4** |  |  |  |  |
| U4-d-ε-r-x-j | 0.000 | 0.000 | 0.000 | 0.000 |
| U4-d-w-x-j | 2.091 | 0.774 | 0.317 | 0.275 |
| U4-d-ε-r-v-j | 12.259 | 9.247 | 8.376 | 8.300 |
| U4-d-ε-r-x-k | 12.923 | 9.975 | 9.095 | 9.018 |
| U4-d-w-v-k | 29.374 | 21.234 | 18.742 | 18.522 |
| U4-w-v-k | 42.418 | 32.315 | 29.324 | 29.065 |
|  |  |  |  |  |
| **U5** |  |  |  |  |
| U5-d-r-x-j | 0.000 | 0.000 | 0.000 | 0.000 |
| U5-d-w-x-j | 3.873 | 1.720 | 1.062 | 1.004 |
| U5-d-r-v-j | 12.880 | 9.938 | 9.054 | 8.977 |
| U5-d-r-x-k | 13.489 | 10.321 | 9.382 | 9.301 |
| U5-r-x-j | 13.528 | 11.145 | 10.472 | 10.417 |
| U5-d-w-v-k | 31.954 | 23.058 | 20.328 | 20.088 |
|  |  |  |  |  |
| **U6** |  |  |  |  |
| U6-d-w-e | 0.000 | 0.000 | 0.000 | 0.000 |
| U6-d-w-g | 0.418 | 0.114 | 0.009 | 0.000 |
| U6-d-w-c | 0.443 | 0.141 | 0.036 | 0.027 |
| U6-s-w-f | 1.133 | 2.143 | 2.496 | 2.529 |
| U6-d-w-e-u | 1.939 | 2.438 | 2.655 | 2.677 |
| U6-d-w-f | 1.950 | 2.177 | 2.240 | 2.245 |
| U6-d-w-h | 2.946 | 2.830 | 2.776 | 2.771 |
| U6-d-y-f | 5.042 | 2.187 | 2.240 | 2.254 |
| U6-d-m-f | 5.605 | 6.403 | 6.705 | 6.734 |
| U6-w-f | 15.142 | 12.733 | 12.081 | 12.025 |
|  |  |  |  |  |
| **U7** |  |  |  |  |
| U7-d-r-ᴧ-χ-α-p | 0.000 | 0.000 | 0.000 | 0.000 |
| U7-d-w-ᴧ-χ-α-p | 1.325 | 0.547 | 0.220 | 0.192 |
| U7-d-w-ᴧ-χ-α-q | 1.561 | 1.903 | 0.348 | 0.008 |
| U7-d-w-ᴧ-χ-β-p | 1.845 | 0.905 | 0.496 | 0.461 |
| U7-d-w-χ-α-p | 2.839 | 2.957 | 2.643 | 2.617 |
| U7-d-w-ᴧ-χ-α-p-u | 3.061 | 4.848 | 5.095 | 5.120 |
| U7-d-w-ᴧ-λ-α-q | 4.390 | 4.075 | 2.315 | 2.252 |
| U7-d-w-ᴧ-λ-α-p | 4.430 | 4.291 | 2.704 | 2.653 |
| U7-d-w-γ-χ-p | 5.497 | 5.086 | 4.571 | 4.525 |
| U7-w-ᴧ-χ-α-p | 14.314 | 12.086 | 11.108 | 11.025 |
|  |  |  |  |  |
| **U8** |  |  |  |  |
| U8-ƞ-d-u-y-κ-ω | 0.000 | 0.000 | 0.000 | 0.000 |
| U8-ƞ-d-u-y-κ-t | 0.000 | 0.012 | 0.005 | 0.006 |
| U8-ƞ-d-u-w-μ-t | 1.213 | 0.577 | 0.279 | 0.253 |
| U8-d-y-κ-ω | 1.378 | 0.617 | 0.402 | 0.383 |
| U8-ƞ-d-u-r-ξ-t | 1.335 | 0.427 | 0.173 | 0.153 |
| U8-ƞ-d-u-y-ς-t | 2.343 | 1.247 | 0.875 | 0.844 |
| U8-ƞ-d-u-y-δ-ω | 2.907 | 1.335 | 0.782 | 0.735 |
| U8-ƞ-d-u-y-δ-t | 2.961 | 1.352 | 0.788 | 0.739 |
| U8-ƞ-d-u-r-δ-n | 3.079 | 1.451 | 0.890 | 0.842 |
| U8-ƞ-d-u-w-δ-t | 3.664 | 1.747 | 1.038 | 0.974 |
| U8-ƞ-s-u-w-τ-t | 5.166 | 2.805 | 1.858 | 1.771 |
| U8-y-κ-ω | 11.949 | 8.164 | 5.577 | 5.453 |
