# Supplementary material for: Computable properties of selected monomeric acylphloroglucinols with anticancer and/or antimalarial activities and first-approximation docking study
Source: J Mol Model. 2025 Mar 12;31(4):113. doi: 10.1007/s00894-025-06299-7 (PMC11903629; doi:10.1007/s00894-025-06299-7)
Supplement: Supplementary file 27 — (DOCX 22.9 KB) [file 894_2025_6299_MOESM27_ESM.docx]

**Table S13**

**Relative energies of the calculated conformers of the considered ACPL molecules *in vacuo*, in chloroform, in acetonitrile and in water (respectively denoted as vac, chlrf, actn and aq in the column headings).**

HF/6-31G(d,p) results from full optimisation calculations. For each molecule, the conformers are listed in order of increasing relative energies in the DFT results *in vacuo*.

| Molecules and conformers | Relative energy (kcal mol^-1^) | | | |
| --- | --- | --- | --- | --- |
|  | vac | chlrf | actn | aq |
| **U1** |  |  |  |  |
| U1-d-r-a | 0.000 | 0.000 | 0.000 | 0.000 |
| U1-d-w-a | 1.516 | 0.623 | 0.281 | 0.249 |
| U1-d-u-r-a | 4.373 | 4.220 | 4.189 | 4.186 |
| U1-d-u-w-a | 4.785 | 4.417 | 4.287 | 4.275 |
| U1-r-a | 15.476 | 8.215 | 7.092 | 6.994 |
|  |  |  |  |  |
| **U2** |  |  |  |  |
| U2-d-v-a | 0.041 | 0.000 | 0.000 | 0.000 |
| U2-s-v-a | 0.000 | 0.045 | 0.047 | 0.022 |
| U2-s-v-u-a | 5.019 | 3.275 | 3.229 | 3.770 |
| U2-d-x-a | 5.822 | 4.572 | 4.540 | 4.907 |
| U2-x-a | 14.228 | 9.001 | 8.905 | 10.121 |
|  |  |  |  |  |
| **U3** |  |  |  |  |
| U3-s-x-w-a | 0.000 | 0.045 | 0.051 | 0.000 |
| U3-s-v-w-a | 0.135 | 0.000 | 0.000 | 0.011 |
| U3-s-x-w-b | 1.024 | 0.944 | 0.946 | 0.935 |
| U3-s-x-r-a | 3.994 | 2.869 | 2.846 | 3.142 |
| U3-z-x-w | 10.884 | 6.219 | 6.134 | 7.214 |
| U3-v-w-a | 11.128 | 6.210 | 6.117 | 7.286 |
|  |  |  |  |  |
| **U4** |  |  |  |  |
| U4-d-ε-r-x-j | 0.000 | 0.000 | 0.000 | 0.000 |
| U4-d-w-x-j | 2.669 | 0.518 | 0.473 | 1.030 |
| U4-d-ε-r-v-j | 10.941 | 6.856 | 6.779 | 7.742 |
| U4-d-ε-r-x-k | 11.510 | 7.495 | 7.418 | 8.389 |
| U4-d-w-v-k | 26.088 | 14.693 | 14.467 | 17.284 |
| U4-w-v-k | 36.168 | 22.076 | 21.805 | 25.211 |
|  |  |  |  |  |
| **U5** |  |  |  |  |
| U5-d-r-x-j | 0.000 | 0.000 | 0.000 | 0.000 |
| U5-d-w-x-j | 4.058 | 1.000 | 0.937 | 1.717 |
| U5-d-r-v-j | 11.948 | 7.881 | 7.804 | 8.777 |
| U5-d-r-x-k | 12.498 | 8.237 | 8.156 | 9.173 |
| U5-r-x-j | 10.372 | 7.187 | 7.128 | 7.879 |
| U5-d-w-v-k | 29.280 | 16.845 | 16.599 | 19.661 |
|  |  |  |  |  |
| **U6** |  |  |  |  |
| U6-d-w-e | 0.000 | 0.000 | 0.000 | 0.000 |
| U6-d-w-g | 0.889 | 0.543 | 0.535 | 0.624 |
| U6-d-w-c | 0.887 | 0.547 | 0.540 | 0.625 |
| U6-s-w-f | 1.064 | 2.789 | 2.826 | 2.369 |
| U6-d-w-e-u | 3.007 | 3.422 | 3.435 | 3.282 |
| U6-d-w-f | 1.775 | 2.141 | 2.147 | 2.067 |
| U6-d-w-h | 3.662 | 3.502 | 3.498 | 3.546 |
| U6-d-y-f | 3.512 | 4.385 | 2.147 | 4.185 |
| U6-d-m-f | 4.632 | 5.800 | 5.826 | 5.505 |
| U6-w-f | 11.821 | 8.587 | 8.529 | 9.269 |
|  |  |  |  |  |
| **U7** |  |  |  |  |
| U7-d-r-ᴧ-χ-α-p | 0.000 | 0.204 | 0.256 | 0.000 |
| U7-d-w-ᴧ-χ-α-p | 1.466 | 0.493 | 0.515 | 0.616 |
| U7-d-w-ᴧ-χ-α-q | 1.589 | 0.399 | 0.415 | 0.589 |
| U7-d-w-ᴧ-χ-β-p | 1.815 | 0.500 | 0.517 | 0.699 |
| U7-d-w-χ-α-p | 2.088 | 0.000 | 0.000 | 0.381 |
| U7-d-w-ᴧ-χ-α-p-u | 3.925 | 4.361 | 4.416 | 4.125 |
| U7-d-w-ᴧ-λ-α-q | 4.757 | 2.419 | 2.412 | 2.875 |
| U7-d-w-ᴧ-λ-α-p | 5.015 | 2.788 | 2.785 | 3.204 |
| U7-d-w-γ-χ-p | 4.989 | 2.086 | 2.065 | 2.694 |
| U7-w-ᴧ-χ-α-p | 11.687 | 6.943 | 6.898 | 7.858 |
|  |  |  |  |  |
| **U8** |  |  |  |  |
| U8-ƞ-d-u-y-κ-ω | 0.951 | 0.000 | 0.009 | 0.460 |
| U8-ƞ-d-u-y-κ-t | 1.038 | 0.039 | 0.046 | 0.512 |
| U8-ƞ-d-u-w-μ-t | 0.000 | 0.020 | 0.000 | 0.788 |
| U8-d-y-κ-ω | 1.607 | 1.342 | 1.324 | 0.000 |
| U8-ƞ-d-u-r-ξ-t | 0.071 | 0.179 | 0.171 | 0.839 |
| U8-ƞ-d-u-y-ς-t | 3.011 | 2.819 | 2.801 | 3.574 |
| U8-ƞ-d-u-y-δ-ω | 4.323 | 1.159 | 1.116 | 2.190 |
| U8-ƞ-d-u-y-δ-t | 4.441 | 1.197 | 1.152 | 2.248 |
| U8-ƞ-d-u-r-δ-n | 2.399 | 1.190 | 1.145 | 2.243 |
| U8-ƞ-d-u-w-δ-t | 2.972 | 1.299 | 1.242 | 2.482 |
| U8-ƞ-s-u-w-τ-t | 4.714 | 1.964 | 1.879 | 3.447 |
| U8-y-κ-ω | 7.493 | 3.531 | 3.439 | 5.156 |
