# Supplementary material for: Computable properties of selected monomeric acylphloroglucinols with anticancer and/or antimalarial activities and first-approximation docking study
Source: J Mol Model. 2025 Mar 12;31(4):113. doi: 10.1007/s00894-025-06299-7 (PMC11903629; doi:10.1007/s00894-025-06299-7)
Supplement: Supplementary file 28 — (DOCX 29.8 KB) [file 894_2025_6299_MOESM28_ESM.docx]

**Table S14.**

**Solvation free energy (ΔG_solv_) and its electrostatic (G_el_) and dispersion components for the calculated conformers of the considered ACPL molecules in chloroform, acetonitrile and water (respectively denoted as chlrf, actn, aq in the column headings).**

DFT/B3LYP/6-31+G(d,p) results from full optimisation calculations. For each molecule, the conformers are listed in order of increasing relative energies in the DFT results *in vacuo*.

| Molecules and conformers | ΔG_solv_ (kcal mol^-1^) | | | G_el_ (kcal mol^-1^) | | | Dispersion (kcal mol^-1^) | | |
| --- | --- | --- | --- | --- | --- | --- | --- | --- | --- |
|  | chlrf | actn | aq | chlrf | actn | aq | chlrf | actn | aq |
| **U1** |  |  |  |  |  |  |  |  |  |
| U1-d-r-a | 7.10 | 14.78 | 0.95 | -11.23 | -13.92 | -22.04 | -39.84 | -39.15 | -55.54 |
| U1-d-w-a | 6.39 | 13.62 | -0.26 | -11.96 | -15.07 | -23.25 | -39.83 | -39.15 | -55.52 |
| U1-d-u-r-a | 6.74 | 14.13 | 0.04 | -11.22 | -14.13 | -22.45 | -39.82 | -39.20 | -55.61 |
| U1-d-u-w-a | 6.54 | 13.86 | -0.22 | -11.47 | -14.44 | -22.76 | -39.85 | -39.19 | -55.60 |
| U1-r-a | 4.15 | 10.85 | -5.57 | -15.13 | -18.79 | -29.66 | -39.51 | -38.95 | -55.27 |
|  |  |  |  |  |  |  |  |  |  |
| **U2** |  |  |  |  |  |  |  |  |  |
| U2-d-v-a | 2.49 | 9.03 | -6.05 | -12.63 | -15.61 | -25.23 | -37.73 | -37.03 | -52.40 |
| U2-s-v-a | 1.08 | 6.53 | -7.51 | -13.97 | -17.93 | -26.61 | -37.52 | -36.91 | -52.17 |
| U2-s-v-u-a | 1.94 | 7.79 | -7.51 | -13.17 | -16.75 | -26.61 | -37.46 | -36.83 | -52.17 |
| U2-d-x-a | 2.49 | 9.04 | -6.08 | -12.65 | -15.62 | -25.28 | -37.72 | -37.02 | -52.39 |
| U2-x-a | -0.64 | 4.73 | -12.96 | -17.23 | -21.43 | -33.89 | -37.09 | -36.50 | -51.79 |
|  |  |  |  |  |  |  |  |  |  |
| **U3** |  |  |  |  |  |  |  |  |  |
| U3-s-x-w-a | 2.38 | 8.59 | -6.52 | -13.00 | -16.09 | -25.81 | -36.93 | -36.35 | -51.56 |
| U3-s-v-w-a | 2.31 | 8.48 | -6.61 | -13.04 | -16.17 | -25.89 | -36.96 | -36.37 | -51.56 |
| U3-s-x-w-b | 3.58 | 9.68 | -5.15 | -13.11 | -16.21 | -25.90 | -35.61 | -35.18 | -50.13 |
| U3-s-x-r-a | 1.34 | 6.84 | -8.49 | -14.05 | -17.90 | -27.84 | -36.88 | -36.31 | -51.52 |
| U3-z-x-w | 0.61 | 6.11 | -11.35 | -16.76 | -20.72 | -33.09 | -36.38 | -35.98 | -51.15 |
| U3-v-w-a | 0.55 | 6.03 | -11.42 | -16.85 | -20.87 | -33.26 | -36.39 | -35.98 | -51.12 |
|  |  |  |  |  |  |  |  |  |  |
| **U4** |  |  |  |  |  |  |  |  |  |
| U4-d-ε-r-x-j | -1.06 | 3.80 | -12.36 | -12.72 | -15.86 | -27.40 | -32.40 | -31.86 | -45.20 |
| U4-d-w-x-j | -2.35 | 2.00 | -14.61 | -14.10 | -17.75 | -29.75 | -32.45 | -31.94 | -45.29 |
| U4-d-ε-r-v-j | -3.88 | 0.22 | -18.58 | -16.17 | -20.12 | -34.37 | -32.28 | -31.81 | -45.18 |
| U4-d-ε-r-x-k | -3.89 | 0.18 | -18.63 | -16.22 | -20.20 | -34.47 | -32.24 | -31.77 | -45.12 |
| U4-d-w-v-k | -8.28 | -5.69 | -28.25 | -21.57 | -27.14 | -45.29 | -32.00 | -31.59 | -44.92 |
| U4-w-v-k | -9.72 | -7.40 | -31.96 | -24.04 | -30.00 | -50.39 | -31.52 | -31.11 | -44.29 |
|  |  |  |  |  |  |  |  |  |  |
| **U5** |  |  |  |  |  |  |  |  |  |
| U5-d-r-x-j | 0.41 | 5.09 | -9.39 | -12.13 | -15.33 | -25.27 | -32.23 | -31.74 | -45.01 |
| U5-d-w-x-j | -1.66 | 2.41 | -12.60 | -14.21 | -18.22 | -28.64 | -32.32 | -31.88 | -45.28 |
| U5-d-r-v-j | -2.65 | 1.39 | -16.00 | -15.40 | -19.40 | -32.28 | -32.19 | -31.76 | -45.05 |
| U5-d-r-x-k | -2.87 | 1.17 | -16.19 | -15.66 | -19.68 | -32.51 | -32.16 | -31.72 | -45.05 |
| U5-r-x-j | -1.76 | 2.67 | -14.13 | -15.20 | -18.79 | -31.19 | -31.75 | -31.33 | -44.54 |
| U5-d-w-v-k | -8.11 | -6.06 | -27.36 | -21.28 | -27.26 | -44.03 | -31.96 | -31.60 | -44.96 |
|  |  |  |  |  |  |  |  |  |  |
| **U6** |  |  |  |  |  |  |  |  |  |
| U6-d-w-e | 2.20 | 6.35 | -4.08 | -9.27 | -12.08 | -18.33 | -28.00 | -27.63 | -39.28 |
| U6-d-w-g | 2.40 | 6.53 | -3.73 | -9.65 | -12.50 | -18.76 | -27.55 | -27.19 | -38.66 |
| U6-d-w-c | 2.27 | 6.40 | -3.89 | -9.63 | -12.49 | -18.77 | -27.54 | -27.18 | -38.63 |
| U6-s-w-f | 2.86 | 7.47 | -2.34 | -8.33 | -10.65 | -16.26 | -28.02 | -27.65 | -39.28 |
| U6-d-w-e-u | 2.41 | 6.75 | -3.66 | -9.00 | -11.62 | -17.89 | -28.00 | -27.60 | -39.20 |
| U6-d-w-f | 2.46 | 6.68 | -3.42 | -8.98 | -11.76 | -17.72 | -28.03 | -27.64 | -39.24 |
| U6-d-w-h | 2.73 | 6.89 | -3.22 | -9.40 | -12.24 | -18.43 | -27.50 | -27.13 | -38.53 |
| U6-d-y-f | 2.52 | 6.68 | -3.41 | -9.01 | -11.76 | -17.72 | -27.95 | -27.64 | -39.23 |
| U6-d-m-f | 3.62 | 8.36 | -1.73 | -8.50 | -10.74 | -16.88 | -27.46 | -27.10 | -38.51 |
| U6-w-f | 0.69 | 4.42 | -7.96 | -12.09 | -15.42 | -23.90 | -27.46 | -27.15 | -38.63 |
|  |  |  |  |  |  |  |  |  |  |
| **U7** |  |  |  |  |  |  |  |  |  |
| U7-d-r-ᴧ-χ-α-p | 2.01 | 7.34 | -10.86 | -17.02 | -21.27 | -34.42 | -36.45 | -36.23 | -51.72 |
| U7-d-w-ᴧ-χ-α-p | 1.33 | 6.18 | -12.12 | -17.69 | -22.41 | -35.68 | -36.45 | -36.23 | -51.71 |
| U7-d-w-ᴧ-χ-α-q | 0.73 | 5.69 | -12.13 | -17.65 | -22.65 | -35.58 | -36.95 | -36.31 | -51.77 |
| U7-d-w-ᴧ-χ-β-p | 1.34 | 6.12 | -12.27 | -17.91 | -22.79 | -36.31 | -36.24 | -35.97 | -51.28 |
| U7-d-w-χ-α-p | 2.38 | 7.46 | -11.35 | -17.93 | -22.55 | -36.77 | -35.48 | -35.18 | -50.20 |
| U7-d-w-ᴧ-χ-α-p-u | 1.86 | 6.83 | -11.96 | -16.54 | -20.83 | -34.46 | -36.39 | -36.46 | -52.02 |
| U7-d-w-ᴧ-λ-α-q | -0.03 | 4.59 | -14.67 | -18.26 | -23.77 | -37.94 | -37.09 | -36.45 | -51.99 |
| U7-d-w-ᴧ-λ-α-p | 0.77 | 5.21 | -13.91 | -18.20 | -23.32 | -37.39 | -36.29 | -36.13 | -51.61 |
| U7-d-w-γ-χ-p | 1.91 | 6.42 | -13.27 | -18.36 | -23.45 | -38.37 | -35.71 | -35.58 | -50.87 |
| U7-w-ᴧ-χ-α-p | 0.02 | 4.39 | -16.64 | -20.55 | -25.72 | -42.12 | -35.31 | -35.22 | -50.35 |
|  |  |  |  |  |  |  |  |  |  |
| **U8** |  |  |  |  |  |  |  |  |  |
| U8-ƞ-d-u-y-κ-ω | -8.06 | -4.12 | -27.64 | -19.67 | -23.63 | -42.55 | -31.80 | -31.21 | -44.19 |
| U8-ƞ-d-u-y-κ-t | -8.04 | -4.15 | -27.64 | -19.66 | -23.66 | -42.53 | -31.79 | -31.21 | -44.22 |
| U8-ƞ-d-u-w-μ-t | -8.19 | -4.63 | -28.42 | -20.09 | -24.47 | -43.61 | -31.77 | -31.23 | -44.32 |
| U8-d-y-κ-ω | -8.76 | -5.03 | -29.12 | -20.66 | -24.88 | -44.38 | -31.82 | -31.27 | -44.31 |
| U8-ƞ-d-u-r-ξ-t | -8.72 | -5.05 | -29.10 | -20.46 | -24.64 | -44.10 | -31.71 | -31.17 | -44.16 |
| U8-ƞ-d-u-y-ς-t | -8.76 | -5.16 | -29.73 | -20.73 | -25.02 | -45.00 | -31.68 | -31.15 | -44.16 |
| U8-ƞ-d-u-y-δ-ω | -9.59 | -6.38 | -30.29 | -20.97 | -25.67 | -44.94 | -31.99 | -31.38 | -44.42 |
| U8-ƞ-d-u-y-δ-t | -9.58 | -6.42 | -30.30 | -20.96 | -25.71 | -44.92 | -31.98 | -31.38 | -44.44 |
| U8-ƞ-d-u-r-δ-n | -9.29 | -5.98 | -29.90 | -21.10 | -25.77 | -45.08 | -31.90 | -31.31 | -44.39 |
| U8-ƞ-d-u-w-δ-t | -9.46 | -6.47 | -30.41 | -21.29 | -26.23 | -45.54 | -31.85 | -31.30 | -44.39 |
| U8-ƞ-s-u-w-τ-t | -9.72 | -7.30 | -31.96 | -21.71 | -27.24 | -47.28 | -31.73 | -31.19 | -44.26 |
| U8-y-κ-ω | -11.21 | -8.90 | -35.36 | -24.42 | -29.88 | -51.95 | -31.38 | -30.95 | -43.89 |
