# Supplementary material for: Computable properties of selected monomeric acylphloroglucinols with anticancer and/or antimalarial activities and first-approximation docking study
Source: J Mol Model. 2025 Mar 12;31(4):113. doi: 10.1007/s00894-025-06299-7 (PMC11903629; doi:10.1007/s00894-025-06299-7)
Supplement: Supplementary file 29 — (DOCX 30.3 KB) [file 894_2025_6299_MOESM29_ESM.docx]

**Table S15.**

**Solvation free energy (ΔG_solv_) and its electrostatic (G_el_) and dispersion components for the calculated conformers of the considered ACPL molecules in chloroform, acetonitrile and water (respectively denoted as chlrf, actn, aq in the column headings).**

HF/6-31G(d,p) results from full optimisation calculations. For each molecule, the conformers are listed in order of increasing relative energies in the DFT results *in vacuo*.

| Molecules and conformers | ΔG_solv_ (kcal mol^-1^) | | | G_el_ (kcal mol^-1^) | | | Dispersion (kcal mol^-1^) | | |
| --- | --- | --- | --- | --- | --- | --- | --- | --- | --- |
|  | chlrf | actn | aq | chlrf | actn | aq | chlrf | actn | aq |
| **U1** |  |  |  |  |  |  |  |  |  |
| U1-d-r-a | -3.22 | 3.76 | -11.09 | -5.46 | -7.76 | -17.08 | -42.63 | -42.03 | -59.67 |
| U1-d-w-a | -4.00 | 2.57 | -12.47 | -6.25 | -8.95 | -18.45 | -42.63 | -42.02 | -59.67 |
| U1-d-u-r-a | -2.47 | 4.64 | -9.82 | -4.95 | -7.10 | -16.04 | -42.38 | -41.78 | -59.30 |
| U1-d-u-w-a | -2.66 | 4.35 | -10.10 | -5.12 | -7.37 | -16.28 | -42.39 | -41.79 | -59.33 |
| U1-r-a | -5.46 | 0.22 | -7.67 | -8.69 | -12.38 | -31.12 | -42.39 | -41.88 | -55.26 |
|  |  |  |  |  |  |  |  |  |  |
| **U2** |  |  |  |  |  |  |  |  |  |
| U2-d-v-a | -4.10 | 2.02 | -15.74 | -6.61 | -9.43 | -22.14 | -40.78 | -40.21 | -57.10 |
| U2-s-v-a | -4.82 | 0.56 | -17.67 | -7.53 | -11.05 | -24.25 | -40.52 | -39.97 | -56.77 |
| U2-s-v-u-a | -3.76 | 1.99 | -15.65 | -6.78 | -9.88 | -22.50 | -40.24 | -39.76 | -56.52 |
| U2-d-x-a | 1.83 | 8.39 | -8.80 | -12.54 | -15.35 | -26.88 | -37.78 | -37.10 | -52.58 |
| U2-x-a | -1.37 | 3.86 | -16.04 | -17.41 | -21.60 | -36.08 | -37.02 | -36.50 | -51.88 |
|  |  |  |  |  |  |  |  |  |  |
| **U3** |  |  |  |  |  |  |  |  |  |
| U3-s-x-w-a | -3.53 | 2.38 | -15.17 | -6.79 | -9.72 | -22.37 | -39.95 | -39.53 | -56.25 |
| U3-s-v-w-a | 1.96 | 8.19 | -8.92 | -12.94 | -15.87 | -27.44 | -36.93 | -36.40 | -51.68 |
| U3-s-x-w-b | -2.13 | 3.65 | -13.46 | -6.80 | -9.67 | -22.24 | -38.37 | -38.12 | -54.34 |
| U3-s-x-r-a | -3.80 | 1.68 | -16.40 | -7.21 | -10.55 | -23.76 | -39.73 | -39.30 | -55.94 |
| U3-z-x-w | -5.39 | -0.59 | -20.98 | -9.76 | -13.87 | -29.64 | -39.66 | -39.35 | -56.10 |
| U3-v-w-a | -0.39 | 5.03 | -14.54 | -17.11 | -21.05 | -35.36 | -36.41 | -35.99 | -51.22 |
|  |  |  |  |  |  |  |  |  |  |
| **U4** |  |  |  |  |  |  |  |  |  |
| U4-d-ε-r-x-j | 1.68 | 7.39 | -5.50 | -4.72 | -6.76 | -15.54 | -32.68 | -32.16 | -45.61 |
| U4-d-w-x-j | -0.12 | 4.87 | -9.08 | -17.11 | -9.25 | -19.12 | -32.88 | -32.37 | -45.93 |
| U4-d-ε-r-v-j | -0.81 | 3.85 | -12.20 | -7.31 | -10.43 | -22.43 | -32.81 | -32.33 | -45.89 |
| U4-d-ε-r-x-k | -0.82 | 3.79 | -12.08 | -7.32 | -10.51 | -22.34 | -32.78 | -32.29 | -45.84 |
| U4-d-w-v-k | -5.29 | -6.16 | -23.00 | -12.12 | -27.32 | -33.85 | -32.98 | -31.50 | -46.30 |
| U4-w-v-k | -10.48 | -7.88 | -35.90 | -24.63 | -30.25 | -54.12 | -31.39 | -30.98 | -44.09 |
|  |  |  |  |  |  |  |  |  |  |
| **U5** |  |  |  |  |  |  |  |  |  |
| U5-d-r-x-j | 1.32 | 7.10 | -6.20 | -4.44 | -6.32 | -15.39 | -32.95 | -32.54 | -46.24 |
| U5-d-w-x-j | -2.34 | 1.76 | -15.84 | -14.37 | -18.13 | -31.06 | -32.33 | -31.84 | -45.18 |
| U5-d-r-v-j | -1.20 | 3.49 | -12.30 | -7.00 | -10.01 | -21.63 | -33.13 | -32.74 | -46.58 |
| U5-d-r-x-k | -1.34 | 3.30 | -12.76 | -7.13 | -10.18 | -22.06 | -33.11 | -32.72 | -46.54 |
| U5-r-x-j | -2.03 | 2.73 | -16.44 | -15.27 | -18.53 | -33.29 | -31.74 | -31.34 | -44.54 |
| U5-d-w-v-k | -6.40 | -4.32 | -24.31 | -12.41 | -18.12 | -34.09 | -33.40 | -33.08 | -47.12 |
|  |  |  |  |  |  |  |  |  |  |
| **U6** |  |  |  |  |  |  |  |  |  |
| U6-d-w-e | -0.64 | 4.05 | -6.70 | -4.11 | -6.02 | -13.22 | -28.84 | -28.49 | -40.51 |
| U6-d-w-g | -0.36 | 4.24 | -6.25 | -4.34 | -6.35 | -13.46 | -28.33 | -27.96 | -39.74 |
| U6-d-w-c | -0.37 | 4.22 | -6.28 | -4.33 | -6.34 | -13.47 | -28.34 | -27.98 | -6.28 |
| U6-s-w-f | 0.54 | 5.90 | -3.51 | -2.74 | -3.95 | -9.77 | -28.79 | -28.40 | -40.33 |
| U6-d-w-e-u | 2.10 | 6.56 | -5.39 | -9.25 | -11.77 | -19.59 | -27.95 | -27.54 | -39.10 |
| U6-d-w-f | -0.72 | 3.36 | -6.61 | -3.95 | -5.83 | -12.85 | -28.94 | -28.57 | -40.60 |
| U6-d-w-h | -0.47 | 4.11 | -6.38 | -4.22 | -6.19 | -13.33 | -28.23 | -27.87 | -39.62 |
| U6-d-y-f | -0.14 | 4.69 | -6.61 | -3.67 | -5.47 | -12.85 | -28.69 | -28.30 | -40.60 |
| U6-d-m-f | 0.16 | 5.03 | -5.22 | -3.51 | -5.16 | -11.97 | -28.39 | -28.07 | -39.89 |
| U6-w-f | -1.28 | 2.82 | -9.56 | -5.66 | -8.29 | -17.42 | -28.52 | -28.18 | -40.07 |
|  |  |  |  |  |  |  |  |  |  |
| **U7** |  |  |  |  |  |  |  |  |  |
| U7-d-r-ᴧ-χ-α-p | 2.16 | 8.16 | -12.90 | -16.11 | -19.54 | -35.33 | -36.54 | -36.37 | -52.02 |
| U7-d-w-ᴧ-χ-α-p | -0.94 | -0.72 | -14.72 | -8.20 | -8.34 | -26.71 | -38.90 | -39.00 | -55.96 |
| U7-d-w-ᴧ-χ-α-q | -1.29 | -1.42 | -15.96 | -8.21 | -8.34 | -27.16 | -39.40 | -39.43 | -56.34 |
| U7-d-w-ᴧ-χ-β-p | -1.20 | -1.33 | -15.59 | -8.18 | -8.29 | -27.03 | -39.35 | -39.35 | -56.16 |
| U7-d-w-χ-α-p | 1.91 | 7.47 | -13.90 | -17.84 | -22.01 | -38.88 | -35.46 | -35.07 | -49.87 |
| U7-d-w-ᴧ-χ-α-p-u | 3.19 | 8.99 | -11.76 | -16.25 | -19.93 | -35.95 | -35.47 | -35.26 | -50.32 |
| U7-d-w-ᴧ-λ-α-q | -1.96 | -2.04 | -17.54 | -8.77 | -8.88 | -28.60 | -39.47 | -39.47 | -56.45 |
| U7-d-w-ᴧ-λ-α-p | -1.65 | -1.40 | -16.53 | -8.85 | -8.97 | -28.45 | -38.94 | -39.02 | -56.01 |
| U7-d-w-γ-χ-p | -0.79 | -0.83 | -17.03 | -8.55 | -8.62 | -29.19 | -38.48 | -38.53 | -55.51 |
| U7-w-ᴧ-χ-α-p | 0.21 | 5.42 | -17.91 | -20.20 | -24.66 | -43.61 | -34.89 | -34.56 | -49.25 |
|  |  |  |  |  |  |  |  |  |  |
| **U8** |  |  |  |  |  |  |  |  |  |
| U8-ƞ-d-u-y-κ-ω | -7.81 | -3.20 | -30.46 | -19.10 | -22.33 | -44.92 | -31.76 | -31.20 | -44.20 |
| U8-ƞ-d-u-y-κ-t | -6.10 | -2.63 | -28.60 | -9.86 | -14.25 | -36.18 | -35.29 | -35.16 | -50.27 |
| U8-ƞ-d-u-w-μ-t | -6.71 | -3.74 | -29.93 | -10.52 | -15.38 | -50.36 | -35.28 | -35.20 | -50.36 |
| U8-d-y-κ-ω | -8.49 | -4.37 | -32.40 | -19.98 | -23.98 | -47.42 | -31.77 | -31.09 | -44.07 |
| U8-ƞ-d-u-r-ξ-t | -6.63 | -3.27 | -29.58 | -10.43 | -14.92 | -37.21 | -35.27 | -35.15 | -50.26 |
| U8-ƞ-d-u-y-ς-t | -8.35 | -2.98 | -29.88 | -20.13 | -14.87 | -37.78 | -31.52 | -35.14 | -50.30 |
| U8-ƞ-d-u-y-δ-ω | -7.74 | -4.95 | -31.59 | -11.35 | -16.44 | -39.01 | -35.50 | -35.35 | -50.54 |
| U8-ƞ-d-u-y-δ-t | -7.80 | -5.14 | -31.66 | -11.41 | -16.62 | -39.07 | -35.50 | -35.36 | -50.54 |
| U8-ƞ-d-u-r-δ-n | -7.86 | -5.00 | -31.67 | -11.47 | -16.48 | -39.08 | -35.50 | -35.35 | -50.53 |
| U8-ƞ-d-u-w-δ-t | -8.00 | -5.45 | -31.94 | -11.75 | -17.04 | -39.48 | -35.37 | -35.26 | -50.44 |
| U8-ƞ-s-u-w-τ-t | -8.39 | -6.46 | -33.68 | -12.27 | -18.17 | -41.39 | -35.31 | -35.24 | -50.43 |
| U8-y-κ-ω | -10.76 | -7.12 | -37.08 | -23.57 | -28.02 | -53.54 | -31.44 | -30.86 | -43.83 |
