# Supplementary material for: Computable properties of selected monomeric acylphloroglucinols with anticancer and/or antimalarial activities and first-approximation docking study
Source: J Mol Model. 2025 Mar 12;31(4):113. doi: 10.1007/s00894-025-06299-7 (PMC11903629; doi:10.1007/s00894-025-06299-7)
Supplement: Supplementary file 30 — (DOCX 57.1 KB) [file 894_2025_6299_MOESM30_ESM.docx]

**Table S16.**

**Parameters of the intramolecular hydrogen bonds in the calculated conformers of the considered ACPL molecules in chloroform, acetonitrile and water (respectively denoted as chlrf, actn, and aq in the column headings).**

DFT/B3LYP/6-31+G(d,p) results from full optimisation PCM calculations.

The various IHBs are considered individually, across the molecules and conformers in which they are present. The molecules are denoted with the symbols listed in table 1, and the conformers with the symbols listed in table 2. For each molecule, the conformers are listed in order of increasing relative energies in the DFT results *in vacuo*.

| Molecules and conformers | chlrf | | | actn | | | aq | | |
| --- | --- | --- | --- | --- | --- | --- | --- | --- | --- |
|  | OH···O  (Å) | O···O  (Å) | OĤO  (°) | OH···O  (Å) | O···O  (Å) | OĤO  (°) | OH···O  (Å) | O···O  (Å) | OĤO  (°) |
| H15···O14 | | | | | | | | | |
| **U1** |  |  |  |  |  |  |  |  |  |
| U1-d-r-a | 1.552 | 2.481 | 151.0 | 1.548 | 2.479 | 151.4 | 1.547 | 2.479 | 151.4 |
| U1-d-w-a | 1.558 | 2.481 | 150.5 | 1.552 | 2.478 | 150.8 | 1.552 | 2.477 | 150.7 |
| U1-d-u-r-a | 1.719 | 2.549 | 143.4 | 1.719 | 2.549 | 143.4 | 1.719 | 2.549 | 143.4 |
| U1-d-u-w-a | 1.558 | 2.482 | 150.5 | 1.553 | 2.479 | 150.8 | 1.553 | 2.479 | 150.8 |
|  |  |  |  |  |  |  |  |  |  |
| **U2** |  |  |  |  |  |  |  |  |  |
| U2-d-v-a | 1.538 | 2.473 | 151.6 | 1.536 | 2.472 | 151.8 | 1.536 | 2.472 | 151.9 |
| U2-d-x-a | 1.539 | 2.473 | 151.6 | 1.536 | 2.472 | 151.8 | 1.535 | 2.472 | 151.9 |
|  |  |  |  |  |  |  |  |  |  |
| **U4** |  |  |  |  |  |  |  |  |  |
| U4-d-ε-r-x-j | 1.533 | 2.470 | 151.8 | 1.530 | 2.469 | 152.1 | 1.530 | 2.469 | 152.1 |
| U4-d-w-x-j | 1.537 | 2.473 | 151.7 | 1.533 | 2.471 | 152.0 | 1.533 | 2.471 | 152.0 |
| U4-d-ε-r-v-j | 1.533 | 2.470 | 151.8 | 1.530 | 2.469 | 152.1 | 1.529 | 2.469 | 152.1 |
| U4-d-w-v-k | 1.535 | 2.472 | 151.9 | 1.531 | 2.470 | 152.2 | 1.531 | 2.470 | 152.2 |
|  |  |  |  |  |  |  |  |  |  |
| **U5** |  |  |  |  |  |  |  |  |  |
| U5-d-r-x-j | 1.529 | 2.468 | 151.8 | 1.528 | 2.468 | 152.1 | 1.528 | 2.468 | 152.1 |
| U5-d-w-x-j | 1.531 | 2.469 | 151.9 | 1.527 | 2.467 | 152.3 | 1.527 | 2.467 | 152.3 |
| U5-d-r-v-j | 1.532 | 2.469 | 151.8 | 1.529 | 2.468 | 152.1 | 1.528 | 2.468 | 152.1 |
| U5-d-r-x-k | 1.531 | 2.469 | 151.8 | 1.528 | 2.468 | 152.1 | 1.528 | 2.468 | 152.1 |
| U5-d-w-v-k | 1.535 | 2.471 | 151.8 | 1.530 | 2.469 | 152.2 | 1.530 | 2.469 | 152.2 |
|  |  |  |  |  |  |  |  |  |  |
| **U6** |  |  |  |  |  |  |  |  |  |
| U6-d-w-e | 1.552 | 2.482 | 151.5 | 1.546 | 2.479 | 151.9 | 1.546 | 2.479 | 151.9 |
| U6-d-w-g | 1.541 | 2.475 | 151.8 | 1.537 | 2.473 | 152.1 | 1.536 | 2.473 | 152.2 |
| U6-d-w-c | 1.543 | 2.476 | 151.8 | 1.538 | 2.474 | 152.1 | 1.537 | 2.474 | 152.1 |
| U6-d-w-e-u | 1.564 | 2.487 | 150.8 | 1.557 | 2.483 | 151.2 | 1.556 | 2.483 | 151.3 |
| U6-d-w-f | 1.545 | 2.477 | 151.7 | 1.539 | 2.474 | 152.1 | 1.538 | 2.474 | 152.1 |
| U6-d-w-h | 1.525 | 2.463 | 152.1 | 1.520 | 2.460 | 152.4 | 1.519 | 2.460 | 152.5 |
| U6-d-y-f | 1.573 | 2.490 | 149.9 | 1.539 | 2.474 | 152.1 | 1.574 | 2.491 | 149.9 |
| U6-d-m-f | 1.556 | 2.483 | 150.8 | 1.539 | 2.472 | 151.8 | 1.550 | 2.480 | 151.2 |
|  |  |  |  |  |  |  |  |  |  |
| **U7** |  |  |  |  |  |  |  |  |  |
| U7-d-r-ᴧ-χ-α-p | 1.574 | 2.490 | 149.7 | 1.572 | 2.489 | 150.0 | 1.571 | 2.489 | 150.0 |
| U7-d-w-ᴧ-χ-α-p | 1.578 | 2.493 | 149.6 | 1.574 | 2.491 | 149.9 | 1.573 | 2.491 | 150.0 |
| U7-d-w-ᴧ-χ-α-q | 1.573 | 2.490 | 149.8 | 1.574 | 2.491 | 149.9 | 1.573 | 2.490 | 149.9 |
| U7-d-w-ᴧ-χ-β-p | 1.556 | 2.483 | 150.8 | 1.574 | 2.491 | 149.9 | 1.574 | 2.491 | 150.0 |
| U7-d-w-χ-α-p | 1.555 | 2.482 | 150.9 | 1.550 | 2.480 | 151.2 | 1.550 | 2.480 | 151.2 |
| U7-d-w-ᴧ-χ-α-p-u | 1.580 | 2.491 | 149.3 | 1.571 | 2.485 | 149.7 | 1.570 | 2.485 | 149.7 |
| U7-d-w-ᴧ-λ-α-q | 1.574 | 2.490 | 149.8 | 1.575 | 2.491 | 149.9 | 1.574 | 2.491 | 149.9 |
| U7-d-w-ᴧ-λ-α-p | 1.536 | 2.472 | 152.2 | 1.574 | 2.491 | 149.9 | 1.538 | 2.474 | 152.2 |
| U7-d-w-γ-χ-p | 1.543 | 2.474 | 151.5 | 1.550 | 2.480 | 151.2 | 1.538 | 2.472 | 151.8 |
|  |  |  |  |  |  |  |  |  |  |
| **U8** |  |  |  |  |  |  |  |  |  |
| U8-ƞ-d-u-y-κ-ω | 1.621 | 2.528 | 149.0 | 1.618 | 2.527 | 149.2 | 1.617 | 2.526 | 149.2 |
| U8-ƞ-d-u-y-κ-t | 1.620 | 2.527 | 149.0 | 1.616 | 2.526 | 149.2 | 1.616 | 2.525 | 149.2 |
| U8-ƞ-d-u-w-μ-t | 1.639 | 2.540 | 148.4 | 1.633 | 2.537 | 148.7 | 1.632 | 2.536 | 148.7 |
| U8-d-y-κ-ω | 1.619 | 2.526 | 148.9 | 1.616 | 2.525 | 149.1 | 1.616 | 2.525 | 149.1 |
| U8-ƞ-d-u-r-ξ-t | 1.620 | 2.527 | 148.9 | 1.615 | 2.524 | 149.2 | 1.615 | 2.524 | 149.3 |
| U8-ƞ-d-u-y-ς-t | 1.620 | 2.527 | 148.9 | 1.616 | 2.525 | 149.2 | 1.616 | 2.525 | 149.2 |
| U8-ƞ-d-u-y-δ-ω | 1.620 | 2.528 | 149.0 | 1.617 | 2.526 | 149.2 | 1.617 | 2.526 | 149.2 |
| U8-ƞ-d-u-y-δ-t | 1.619 | 2.527 | 149.0 | 1.616 | 2.525 | 149.2 | 1.616 | 2.525 | 149.3 |
| U8-ƞ-d-u-r-δ-n | 1.627 | 2.532 | 148.8 | 1.623 | 2.531 | 149.0 | 1.623 | 2.530 | 149.0 |
| U8-ƞ-d-u-w-δ-t | 1.638 | 2.539 | 148.5 | 1.632 | 2.536 | 148.8 | 1.632 | 2.536 | 148.8 |
| U8-ƞ-d-u-w-τ-t | 1.639 | 2.540 | 148.4 | 1.632 | 2.536 | 148.7 | 1.632 | 2.536 | 148.8 |
| H17···O14 | | | | | | | | | |
| **U2** |  |  |  |  |  |  |  |  |  |
| U2-s-v-a | 1.554 | 2.480 | 150.7 | 1.548 | 2.477 | 151.1 | 1.548 | 2.532 | 143.9 |
| U2-s-v-u-a | 1.567 | 2.486 | 150.1 | 1.559 | 2.482 | 150.5 | 1.559 | 2.482 | 150.5 |
|  |  |  |  |  |  |  |  |  |  |
| **U3** |  |  |  |  |  |  |  |  |  |
| U3-s-x-w-a | 1.546 | 2.475 | 150.9 | 1.542 | 2.473 | 151.3 | 1.541 | 2.473 | 151.3 |
| U3-s-v-w-a | 1.546 | 2.475 | 150.9 | 1.542 | 2.474 | 151.3 | 1.542 | 2.473 | 151.3 |
| U3-s-x-w-b | 1.536 | 2.468 | 151.1 | 1.533 | 2.467 | 151.4 | 1.532 | 2.467 | 151.4 |
| U3-s-x-r-a | 1.550 | 2.477 | 150.8 | 1.545 | 2.475 | 151.2 | 1.544 | 2.475 | 151.2 |
|  |  |  |  |  |  |  |  |  |  |
| **U6** |  |  |  |  |  |  |  |  |  |
| U6-s-w-f | 1.548 | 2.477 | 151.0 | 1.543 | 2.474 | 151.3 | 1.542 | 2.474 | 151.4 |
| H23···O32 | | | | | | | | | |
| **U4** |  |  |  |  |  |  |  |  |  |
| U4-d-ε-r-x-j | 1.644 | 2.536 | 147.1 | 1.640 | 2.533 | 147.2 | 1.640 | 2.533 | 147.2 |
| U4-d-w-x-j | 1.639 | 2.532 | 147.2 | 1.637 | 2.531 | 147.3 | 1.636 | 2.531 | 147.4 |
| U4-d-ε-r-v-j | 1.602 | 2.512 | 148.9 | 1.596 | 2.509 | 149.2 | 1.595 | 2.508 | 149.2 |
|  |  |  |  |  |  |  |  |  |  |
| **U5** |  |  |  |  |  |  |  |  |  |
| U5-d-r-x-j | 1.648 | 2.544 | 147.9 | 1.643 | 2.542 | 148.1 | 1.643 | 2.542 | 148.1 |
| U5-d-w-x-j | 1.651 | 2.546 | 147.9 | 1.647 | 2.545 | 148.1 | 1.647 | 2.544 | 148.1 |
| U5-d-r-v-j | 1.591 | 2.511 | 150.1 | 1.583 | 2.506 | 150.5 | 1.582 | 2.505 | 150.6 |
| U5-r-x-j | 1.642 | 2.540 | 147.9 | 1.640 | 2.539 | 148.1 | 1.640 | 2.539 | 148.1 |
| H26···O32 | | | | | | | | | |
| **U4** |  |  |  |  |  |  |  |  |  |
| U4-d-ε-r-x-j | 1.686 | 2.570 | 146.5 | 1.683 | 2.568 | 146.6 | 1.682 | 2.568 | 146.6 |
| U4-d-w-x-j | 1.679 | 2.565 | 146.7 | 1.677 | 2.565 | 146.8 | 1.677 | 2.564 | 146.8 |
|  |  |  |  |  |  |  |  |  |  |
| **U5** |  |  |  |  |  |  |  |  |  |
| U5-d-r-x-j | 1.663 | 2.556 | 147.6 | 1.659 | 2.554 | 147.9 | 1.658 | 2.554 | 147.9 |
| U5-d-w-x-j | 1.657 | 2.553 | 147.9 | 1.654 | 2.551 | 148.1 | 1.654 | 2.551 | 148.1 |
| U5-d-r-x-k | 1.606 | 2.522 | 149.9 | 1.598 | 2.518 | 150.3 | 1.597 | 2.517 | 150.4 |
| U5-r-x-j | 1.669 | 2.559 | 147.3 | 1.663 | 2.556 | 147.6 | 1.663 | 2.556 | 147.7 |
| H26···O14 | | | | | | | | | |
| **U7** |  |  |  |  |  |  |  |  |  |
| U7-d-r-ᴧ-χ-α-p | 1.879 | 2.850 | 173.8 | 1.860 | 2.834 | 176.3 | 1.851 | 2.804 | 165.2 |
| U7-d-w-ᴧ-χ-α-p | 1.879 | 2.849 | 173.7 | 1.858 | 2.832 | 176.5 | 1.856 | 2.831 | 176.7 |
| U7-d-w-ᴧ-χ-α-q | 1.867 | 2.813 | 162.9 | 1.850 | 2.824 | 177.0 | 1.849 | 2.824 | 178.1 |
| U7-d-w-ᴧ-χ-β-p | 1.875 | 2.846 | 174.5 | 1.855 | 2.830 | 177.0 | 1.854 | 2.829 | 177.3 |
| U7-d-w-ᴧ-χ-α-p-u | 1.885 | 2.832 | 163.5 | 1.854 | 2.807 | 165.2 | 1.858 | 2.832 | 176.4 |
| U7-d-w-χ-α-p | 1.863 | 2.811 | 163.5 | 1.850 | 2.825 | 176.7 | 1.849 | 2.824 | 176.9 |
| H26···O10 | | | | | | | | | |
| **U8** |  |  |  |  |  |  |  |  |  |
| U8-ƞ-d-u-w-μ-t | 2.613 | 2.869 | 95.3 | 2.644 | 2.874 | 93.8 | 2.647 | 2.875 | 93.6 |
| U8-ƞ-d-u-r-ξ-t | 2.562 | 2.854 | 97.5 | 2.559 | 2.855 | 97.8 | 2.558 | 2.855 | 97.8 |
| U8-ƞ-d-u-y-ς-t | 2.681 | 2.892 | 92.7 | 2.691 | 2.894 | 92.2 | 2.692 | 2.894 | 92.1 |
| H26···O27 | | | | | | | | | |
| **U8** |  |  |  |  |  |  |  |  |  |
| U8-ƞ-d-u-y-κ-t | 2.434 | 2.882 | 107.8 | 2.437 | 2.883 | 107.7 | 2.437 | 2.883 | 107.7 |
| U8-ƞ-d-u-y-σ-ω | 2.444 | 2.886 | 107.4 | 2.445 | 2.887 | 107.4 | 2.445 | 2.887 | 107.4 |
| U8-ƞ-d-u-y-δ-t | 2.444 | 2.886 | 107.4 | 2.445 | 2.887 | 107.4 | 2.444 | 2.887 | 107.4 |
| U8-ƞ-d-u-r-δ-n | 2.436 | 2.882 | 107.7 | 2.435 | 2.882 | 107.7 | 2.435 | 2.882 | 107.7 |
| U8-ƞ-d-u-w-δ-t | 2.438 | 2.883 | 107.6 | 2.437 | 2.883 | 107.7 | 2.437 | 2.883 | 107.7 |
| H28···O25 | | | | | | | | | |
| **U8** |  |  |  |  |  |  |  |  |  |
| U8-ƞ-d-u-w-τ-t | 2.419 | 2.886 | 109.2 | 2.441 | 2.896 | 108.3 | 2.442 | 2.751 | 138.0 |
| U8-ƞ-d-u-w-μ-t | 2.422 | 2.856 | 106.7 | 2.428 | 2.859 | 106.6 | 2.428 | 2.860 | 106.6 |
| U8-ƞ-d-u-y-ς-t | 2.383 | 2.825 | 107.2 | 2.396 | 2.829 | 106.6 | 2.397 | 2.830 | 106.5 |
| H28···O29 | | | | | | | | | |
| **U8** |  |  |  |  |  |  |  |  |  |
| U8-ƞ-d-u-r-ξ-t | 2.192 | 2.702 | 111.4 | 2.192 | 2.702 | 111.4 | 2.192 | 2.702 | 111.4 |
| U8-ƞ-d-u-y-κ-t | 2.239 | 2.735 | 110.5 | 2.248 | 2.739 | 110.1 | 2.249 | 2.739 | 110.1 |
| U8-ƞ-d-u-y-δ-ω | 2.234 | 2.732 | 110.7 | 2.244 | 2.737 | 110.3 | 2.245 | 2.737 | 110.3 |
| U8-ƞ-d-u-y-δ-t | 2.234 | 2.732 | 110.7 | 2.244 | 2.737 | 110.3 | 2.245 | 2.737 | 110.3 |
| U8-ƞ-d-u-r-δ-n | 2.237 | 2.734 | 110.6 | 2.248 | 2.740 | 110.2 | 2.249 | 2.740 | 110.2 |
| U8-ƞ-d-u-w-δ-t | 2.234 | 2.731 | 110.6 | 2.244 | 2.736 | 110.2 | 2.245 | 2.737 | 110.2 |
| H30···O27 | | | | | | | | | |
| **U8** |  |  |  |  |  |  |  |  |  |
| U8-ƞ-d-u-w-τ-t | 2.249 | 2.731 | 109.5 | 2.261 | 2.733 | 108.8 | 2.262 | 2.733 | 108.7 |
| U8-ƞ-d-u-w-μ-t | 2.239 | 2.724 | 109.7 | 2.255 | 2.729 | 108.9 | 2.256 | 2.729 | 108.9 |
| H30···O31 | | | | | | | | | |
| **U8** |  |  |  |  |  |  |  |  |  |
| U8-ƞ-d-u-y-κ-t | 1.910 | 2.767 | 145.2 | 1.901 | 2.762 | 145.7 | 1.900 | 2.762 | 145.7 |
| U8-ƞ-d-u-r-ξ-t | 1.908 | 2.766 | 145.2 | 1.896 | 2.759 | 145.9 | 1.895 | 2.758 | 146.0 |
| U8-ƞ-d-u-y-ς-t | 1.926 | 2.785 | 145.6 | 1.907 | 2.774 | 146.6 | 1.906 | 2.773 | 146.7 |
| U8-ƞ-d-u-y-δ-ω | 1.871 | 2.711 | 142.6 | 1.866 | 2.709 | 142.9 | 1.866 | 2.709 | 143.0 |
| U8-ƞ-d-u-y-δ-t | 1.871 | 2.711 | 142.6 | 1.866 | 2.709 | 142.9 | 1.865 | 2.709 | 143.0 |
| U8-ƞ-d-u-r-δ-n | 1.875 | 2.713 | 142.5 | 1.870 | 2.711 | 142.7 | 1.869 | 2.711 | 142.7 |
| U8-ƞ-d-u-w-δ-t | 1.879 | 2.716 | 142.3 | 1.872 | 2.713 | 142.7 | 1.872 | 2.713 | 142.7 |
| H32···O29 | | | | | | | | | |
| **U8** |  |  |  |  |  |  |  |  |  |
| U8-ƞ-d-u-r-ξ-t | 2.501 | 2.801 | 98.5 | 2.522 | 2.809 | 97.7 | 2.524 | 2.810 | 97.6 |
| U8-ƞ-d-u-y-ς-t | 2.472 | 2.789 | 99.5 | 2.496 | 2.796 | 98.4 | 2.498 | 2.797 | 98.3 |
| U8-ƞ-d-u-y-κ-t | 2.495 | 2.797 | 98.6 | 2.517 | 2.807 | 97.8 | 2.519 | 2.808 | 97.8 |
| U8-ƞ-d-u-w-μ-t | 2.018 | 2.770 | 135.1 | 2.013 | 2.764 | 134.9 | 2.013 | 2.764 | 134.9 |
| U8-ƞ-d-u-w-τ-t | 2.019 | 2.773 | 135.3 | 2.015 | 2.767 | 135.1 | 2.014 | 2.767 | 135.1 |
| H17···π (C13) | | | | | | | | | |
| **U8** |  |  |  |  |  |  |  |  |  |
| U8-ƞ-d-u-y-κ-ω | 2.170 | 2.969 | 138.7 | 2.168 | 2.967 | 138.6 | 2.168 | 2.967 | 138.6 |
| U8-ƞ-d-u-y-κ-t | 2.297 | 3.009 | 131.7 | 2.300 | 3.009 | 131.4 | 2.300 | 3.009 | 131.4 |
| U8-ƞ-d-u-w-μ-t | 2.297 | 3.010 | 131.8 | 2.302 | 3.009 | 131.2 | 2.303 | 3.009 | 131.1 |
| U8-ƞ-d-u-r-ξ-t | 2.297 | 3.009 | 131.7 | 2.300 | 3.008 | 131.3 | 2.300 | 3.008 | 131.3 |
| U8-ƞ-d-u-y-ς-t | 2.315 | 3.018 | 130.8 | 2.322 | 3.018 | 130.1 | 2.323 | 3.018 | 130.0 |
| U8-ƞ-d-u-y-δ-ω | 2.303 | 3.012 | 131.4 | 2.305 | 3.012 | 131.2 | 2.303 | 3.012 | 131.3 |
| U8-ƞ-d-u-y-δ-t | 2.298 | 3.008 | 131.6 | 2.301 | 3.009 | 131.3 | 2.301 | 3.009 | 131.3 |
| U8-ƞ-d-u-r-δ-n | 2.306 | 3.012 | 131.2 | 2.308 | 3.013 | 131.0 | 2.308 | 3.013 | 131.0 |
| U8-ƞ-d-u-w-δ-t | 2.296 | 3.008 | 131.7 | 2.301 | 3.008 | 131.2 | 2.301 | 3.009 | 131.2 |
| U8-ƞ-d-u-w-τ-t | 2.295 | 3.009 | 132.0 | 2.300 | 3.008 | 131.3 | 2.300 | 3.008 | 131.3 |
