# Supplementary material for: Computable properties of selected monomeric acylphloroglucinols with anticancer and/or antimalarial activities and first-approximation docking study
Source: J Mol Model. 2025 Mar 12;31(4):113. doi: 10.1007/s00894-025-06299-7 (PMC11903629; doi:10.1007/s00894-025-06299-7)
Supplement: Supplementary file 31 — (DOCX 56.1 KB) [file 894_2025_6299_MOESM31_ESM.docx]

**Table S17.**

**Parameters of the intramolecular hydrogen bonds in the calculated conformers of the considered ACPL molecules in chloroform, acetonitrile and water (respectively denoted as chlrf, actn, and aq in the column headings).**

HF/6-31G(d,p) results from full optimisation PCM calculations.

The various IHBs are considered individually, across the molecules and conformers in which they are present. The molecules are denoted with the symbols listed in table 1, and the conformers with the symbols listed in table 2. For each molecule, the conformers are listed in order of increasing relative energies in the DFT results *in vacuo*.

| Molecules and conformers | chlrf | | | actn | | | aq | | |
| --- | --- | --- | --- | --- | --- | --- | --- | --- | --- |
|  | OH···O  (Å) | O···O  (Å) | OĤO  (°) | OH···O  (Å) | O···O  (Å) | OĤO  (°) | OH···O  (Å) | O···O  (Å) | OĤO  (°) |
| H15···O14 | | | | | | | | | |
| **U1** |  |  |  |  |  |  |  |  |  |
| U1-d-r-a | 1.685 | 2.530 | 145.0 | 1.682 | 2.529 | 145.2 | 1.682 | 2.528 | 145.2 |
| U1-d-w-a | 1.687 | 2.531 | 145.0 | 1.683 | 2.530 | 145.3 | 1.683 | 2.529 | 145.3 |
| U1-d-u-r-a | 1.706 | 2.542 | 144.1 | 1.700 | 2.539 | 144.4 | 1.699 | 2.539 | 144.5 |
| U1-d-u-w-a | 1.706 | 2.542 | 144.0 | 1.702 | 2.541 | 144.2 | 1.702 | 2.540 | 144.3 |
|  |  |  |  |  |  |  |  |  |  |
| **U2** |  |  |  |  |  |  |  |  |  |
| U2-d-v-a | 1.669 | 2.519 | 145.6 | 1.667 | 2.519 | 145.8 | 1.667 | 2.519 | 145.8 |
| U2-d-x-a | 1.669 | 2.519 | 145.6 | 1.667 | 2.519 | 145.8 | 1.667 | 2.519 | 145.8 |
|  |  |  |  |  |  |  |  |  |  |
| **U4** |  |  |  |  |  |  |  |  |  |
| U4-d-ε-r-x-j | 1.660 | 2.515 | 146.2 | 1.658 | 2.515 | 146.4 | 1.665 | 2.516 | 145.7 |
| U4-d-w-x-j | 1.664 | 2.518 | 146.2 | 1.661 | 2.517 | 146.4 | 1.671 | 2.520 | 145.4 |
| U4-d-ε-r-v-j | 1.660 | 2.516 | 146.3 | 1.658 | 2.515 | 146.5 | 1.666 | 2.517 | 145.7 |
| U4-d-w-v-k | 1.664 | 2.518 | 146.2 | 1.661 | 2.517 | 146.4 | 1.671 | 2.521 | 145.6 |
|  |  |  |  |  |  |  |  |  |  |
| **U5** |  |  |  |  |  |  |  |  |  |
| U5-d-r-x-j | 1.660 | 2.515 | 146.1 | 1.657 | 2.514 | 146.4 | 1.666 | 2.516 | 145.4 |
| U5-d-w-x-j | 1.662 | 2.516 | 146.2 | 1.658 | 2.515 | 146.5 | 1.658 | 2.515 | 146.5 |
| U5-d-r-v-j | 1.660 | 2.515 | 146.1 | 1.657 | 2.514 | 146.4 | 1.667 | 2.517 | 145.4 |
| U5-d-r-x-k | 1.660 | 2.515 | 146.1 | 1.657 | 2.514 | 146.4 | 1.667 | 2.516 | 145.4 |
| U5-d-w-v-k | 1.662 | 2.516 | 146.2 | 1.658 | 2.515 | 146.6 | 1.671 | 2.519 | 145.3 |
|  |  |  |  |  |  |  |  |  |  |
| **U6** |  |  |  |  |  |  |  |  |  |
| U6-d-w-e | 1.686 | 2.533 | 145.5 | 1.683 | 2.532 | 145.8 | 1.682 | 2.532 | 145.8 |
| U6-d-w-g | 1.674 | 2.525 | 145.9 | 1.671 | 2.524 | 146.2 | 1.671 | 2.524 | 146.2 |
| U6-d-w-c | 1.674 | 2.525 | 145.9 | 1.671 | 2.524 | 146.2 | 1.671 | 2.524 | 146.2 |
| U6-d-w-e-u | 1.713 | 2.551 | 144.5 | 1.708 | 2.548 | 144.8 | 1.707 | 2.548 | 144.8 |
| U6-d-w-f | 1.671 | 2.521 | 145.8 | 1.667 | 2.519 | 146.1 | 1.682 | 2.526 | 145.0 |
| U6-d-w-h | 1.663 | 2.515 | 145.9 | 1.661 | 2.514 | 146.2 | 1.661 | 2.514 | 146.2 |
| U6-d-y-f | 1.671 | 2.518 | 145.3 | 1.667 | 2.517 | 145.6 | 1.666 | 2.519 | 146.2 |
| U6-d-m-f | 1.671 | 2.519 | 145.5 | 1.668 | 2.517 | 145.7 | 1.667 | 2.517 | 145.7 |
|  |  |  |  |  |  |  |  |  |  |
| **U7** |  |  |  |  |  |  |  |  |  |
| U7-d-r-ᴧ-χ-α-p | 1.696 | 2.533 | 144.1 | 1.691 | 2.531 | 144.3 | 1.691 | 2.531 | 144.4 |
| U7-d-w-ᴧ-χ-α-p | 1.700 | 2.537 | 144.1 | 1.693 | 2.533 | 144.4 | 1.693 | 2.532 | 144.4 |
| U7-d-w-ᴧ-χ-α-q | 1.699 | 2.536 | 144.1 | 1.691 | 2.531 | 144.4 | 1.691 | 2.531 | 144.4 |
| U7-d-w-ᴧ-χ-β-p | 1.701 | 2.537 | 144.1 | 1.697 | 2.536 | 144.3 | 1.697 | 2.535 | 144.3 |
| U7-d-w-χ-α-p | 1.702 | 2.542 | 144.4 | 1.699 | 2.540 | 144.6 | 1.699 | 2.540 | 144.6 |
| U7-d-w-ᴧ-χ-α-p-u | 1.740 | 2.565 | 142.8 | 1.736 | 2.563 | 143.0 | 1.735 | 2.563 | 143.0 |
| U7-d-w-ᴧ-λ-α-q | 1.700 | 2.537 | 144.0 | 1.696 | 2.535 | 144.3 | 1.696 | 2.534 | 144.3 |
| U7-d-w-ᴧ-λ-α-p | 1.701 | 2.538 | 144.0 | 1.694 | 2.533 | 144.4 | 1.694 | 2.533 | 144.4 |
| U7-d-w-γ-χ-p | 1.689 | 2.531 | 144.7 | 1.684 | 2.529 | 145.1 | 1.683 | 2.528 | 145.1 |
|  |  |  |  |  |  |  |  |  |  |
| **U8** |  |  |  |  |  |  |  |  |  |
| U8-ƞ-d-u-y-κ-ω | 1.812 | 2.626 | 141.6 | 1.812 | 2.626 | 141.7 | 1.812 | 2.626 | 141.7 |
| U8-ƞ-d-u-y-κ-t | 1.810 | 2.625 | 141.7 | 1.810 | 2.625 | 141.8 | 1.810 | 2.625 | 141.8 |
| U8-ƞ-d-u-w-μ-t | 1.824 | 2.635 | 141.4 | 1.821 | 2.634 | 141.6 | 1.821 | 2.634 | 141.6 |
| U8-d-y-κ-ω | 1.837 | 2.646 | 141.0 | 1.861 | 2.663 | 140.4 | 1.861 | 2.663 | 140.4 |
| U8-ƞ-d-u-r-ξ-t | 1.811 | 2.625 | 141.6 | 1.811 | 2.625 | 141.7 | 1.811 | 2.625 | 141.7 |
| U8-ƞ-d-u-y-ς-t | 1.832 | 2.640 | 141.0 | 1.832 | 2.640 | 141.1 | 1.831 | 2.640 | 141.1 |
| U8-ƞ-d-u-y-δ-ω | 1.811 | 2.625 | 141.7 | 1.811 | 2.626 | 141.8 | 1.811 | 2.626 | 141.8 |
| U8-ƞ-d-u-y-δ-t | 1.809 | 2.624 | 141.7 | 1.809 | 2.625 | 141.8 | 1.809 | 2.625 | 141.8 |
| U8-ƞ-d-u-r-δ-n | 1.812 | 2.626 | 141.6 | 1.812 | 2.627 | 141.7 | 1.812 | 2.627 | 141.7 |
| U8-ƞ-d-u-w-δ-t | 1.822 | 2.634 | 141.5 | 1.819 | 2.633 | 141.7 | 1.819 | 2.633 | 141.7 |
| U8-ƞ-d-u-w-τ-t | 1.823 | 2.635 | 141.5 | 1.819 | 2.632 | 141.6 | 1.819 | 2.632 | 141.7 |
| H17···O14 | | | | | | | | | |
| **U2** |  |  |  |  |  |  |  |  |  |
| U2-s-v-a | 1.686 | 2.527 | 144.5 | 1.682 | 2.526 | 144.8 | 1.682 | 2.525 | 144.8 |
| U2-s-v-u-a | 1.718 | 2.550 | 143.5 | 1.712 | 2.546 | 143.8 | 1.711 | 2.545 | 143.8 |
|  |  |  |  |  |  |  |  |  |  |
| U3 |  |  |  |  |  |  |  |  |  |
| U3-s-x-w-a | 1.683 | 2.525 | 144.6 | 1.680 | 2.524 | 144.8 | 1.680 | 2.524 | 144.9 |
| U3-s-v-w-a | 1.683 | 2.525 | 144.6 | 1.680 | 2.524 | 144.8 | 1.680 | 2.524 | 144.9 |
| U3-s-x-w-b | 1.677 | 2.522 | 144.8 | 1.678 | 2.524 | 145.0 | 1.678 | 2.524 | 145.0 |
| U3-s-x-r-a | 1.686 | 2.527 | 144.5 | 1.683 | 2.526 | 144.8 | 1.682 | 2.525 | 144.8 |
|  |  |  |  |  |  |  |  |  |  |
| U6 |  |  |  |  |  |  |  |  |  |
| U6-s-w-f | 1.681 | 2.524 | 144.6 | 1.678 | 2.522 | 144.9 | 1.678 | 2.522 | 144.9 |
|  |  |  |  |  |  |  |  |  |  |
| H23···O32 | | | | | | | | | |
| U4 |  |  |  |  |  |  |  |  |  |
| U4-d-ε-r-x-j | 1.773 | 2.587 | 141.3 | 1.772 | 2.586 | 141.3 | 1.779 | 2.590 | 141.1 |
| U4-d-w-x-j | 1.767 | 2.581 | 141.4 | 1.767 | 2.581 | 141.4 | 1.766 | 2.579 | 141.3 |
| U4-d-ε-r-v-j | 1.775 | 2.592 | 141.7 | 1.772 | 2.589 | 141.8 | 1.785 | 2.598 | 141.3 |
|  |  |  |  |  |  |  |  |  |  |
| U5 |  |  |  |  |  |  |  |  |  |
| U5-d-r-x-j | 1.752 | 2.576 | 142.6 | 1.750 | 2.575 | 142.8 | 1.758 | 2.578 | 142.2 |
| U5-d-w-x-j | 1.754 | 2.578 | 142.7 | 1.752 | 2.577 | 142.8 | 1.752 | 2.577 | 142.8 |
| U5-d-r-v-j | 1.743 | 2.572 | 143.2 | 1.740 | 2.572 | 143.5 | 1.747 | 2.573 | 142.7 |
| U5-r-x-j | 1.749 | 2.573 | 142.6 | 1.748 | 2.573 | 142.7 | 1.748 | 2.573 | 142.7 |
| H26···O32 | | | | | | | | | |
| U4 |  |  |  |  |  |  |  |  |  |
| U4-d-ε-r-x-j | 1.812 | 2.622 | 141.0 | 1.811 | 2.621 | 141.0 | 1.814 | 2.622 | 140.9 |
| U4-d-w-x-j | 1.803 | 2.614 | 141.2 | 1.803 | 2.615 | 141.2 | 1.800 | 2.611 | 141.2 |
|  |  |  |  |  |  |  |  |  |  |
| U5 |  |  |  |  |  |  |  |  |  |
| U5-d-r-x-j | 1.769 | 2.592 | 142.5 | 1.767 | 2.591 | 142.7 | 1.776 | 2.594 | 142.1 |
| U5-d-w-x-j | 1.764 | 2.588 | 142.8 | 1.762 | 2.588 | 142.9 | 1.762 | 2.588 | 142.9 |
| U5-d-r-x-k | 1.758 | 2.586 | 143.2 | 1.753 | 2.583 | 143.5 | 1.769 | 2.592 | 142.5 |
| U5-r-x-j | 1.774 | 2.594 | 142.3 | 1.770 | 2.592 | 142.5 | 1.770 | 2.592 | 142.5 |
| H26···O14 | | | | | | | | | |
| U7 |  |  |  |  |  |  |  |  |  |
| U7-d-r-ᴧ-χ-α-p | 2.016 | 2.910 | 157.0 | 1.991 | 2.891 | 158.3 | 1.989 | 2.890 | 158.4 |
| U7-d-w-ᴧ-χ-α-p | 2.020 | 2.914 | 157.0 | 1.992 | 2.892 | 158.2 | 1.990 | 2.890 | 158.4 |
| U7-d-w-ᴧ-χ-α-q | 2.015 | 2.910 | 157.1 | 1.990 | 2.889 | 158.0 | 1.988 | 2.888 | 158.1 |
| U7-d-w-ᴧ-χ-β-p | 2.019 | 2.914 | 157.2 | 1.998 | 2.900 | 158.8 | 1.996 | 2.899 | 158.9 |
| U7-d-w-ᴧ-χ-α-p-u | 2.060 | 2.962 | 158.5 | 2.036 | 2.946 | 160.5 | 2.034 | 2.944 | 160.6 |
| U7-d-w-χ-α-p | 2.014 | 2.908 | 157.0 | 1.999 | 2.900 | 158.4 | 1.998 | 2.899 | 158.5 |
| H26···O10 | | | | | | | | | |
| U8 |  |  |  |  |  |  |  |  |  |
| U8-ƞ-d-u-w-μ-t | 2.581 | 2.826 | 95.1 | 2.604 | 2.830 | 93.9 | 2.607 | 2.831 | 93.8 |
| U8-ƞ-d-u-r-ξ-t | 2.540 | 2.813 | 96.8 | 2.538 | 2.815 | 97.1 | 2.537 | 2.815 | 97.1 |
| U8-ƞ-d-u-y-ς-t | 2.633 | 2.853 | 97.6 | 2.641 | 2.856 | 93.3 | 2.642 | 2.856 | 93.3 |
| H26···O27 | | | | | | | | | |
| U8 |  |  |  |  |  |  |  |  |  |
| U8-ƞ-d-u-y-κ-t | 2.445 | 2.852 | 105.8 | 2.447 | 2.854 | 105.8 | 2.447 | 2.854 | 105.8 |
| U8-ƞ-d-u-y-σ-ω | 2.455 | 2.857 | 105.5 | 2.451 | 2.856 | 105.7 | 2.452 | 2.857 | 105.7 |
| U8-ƞ-d-u-y-σ-t | 2.452 | 2.856 | 105.7 | 2.452 | 2.857 | 105.7 | 2.452 | 2.857 | 105.7 |
| U8-ƞ-d-u-r-σ-n | 2.452 | 2.857 | 105.7 | 2.452 | 2.857 | 105.7 | 2.452 | 2.858 | 105.7 |
| U8-ƞ-d-u-w-σ-t | 2.455 | 2.857 | 105.6 | 2.452 | 2.857 | 105.7 | 2.452 | 2.857 | 105.7 |
| H28···O25 | | | | | | | | | |
| U8 |  |  |  |  |  |  |  |  |  |
| U8-ƞ-d-u-w-τ-t | 2.424 | 2.856 | 107.5 | 2.448 | 2.865 | 106.6 | 2.450 | 2.866 | 106.5 |
| U8-ƞ-d-u-w-μ-t | 2.428 | 2.827 | 105.2 | 2.434 | 2.830 | 105.0 | 2.434 | 2.831 | 105.0 |
| U8-ƞ-d-u-y-ς-t | 2.395 | 2.804 | 105.8 | 2.404 | 2.806 | 105.3 | 2.405 | 2.806 | 105.2 |
| H28···O29 | | | | | | | | | |
| U8 |  |  |  |  |  |  |  |  |  |
| U8-ƞ-d-u-r-ξ-t | 2.215 | 2.683 | 109.5 | 2.215 | 2.684 | 109.5 | 2.215 | 2.684 | 109.5 |
| U8-ƞ-d-u-y-κ-t | 2.241 | 2.707 | 109.4 | 2.248 | 2.709 | 109.1 | 2.249 | 2.710 | 109.0 |
| U8-ƞ-d-u-y-σ-ω | 2.238 | 2.705 | 109.5 | 2.245 | 2.708 | 109.2 | 2.246 | 2.709 | 109.1 |
| U8-ƞ-d-u-y-σ-t | 2.238 | 2.705 | 109.4 | 2.245 | 2.708 | 109.2 | 2.246 | 2.708 | 109.1 |
| U8-ƞ-d-u-r-σ-n | 2.240 | 2.707 | 109.4 | 2.249 | 2.711 | 109.1 | 2.249 | 2.711 | 109.1 |
| U8-ƞ-d-u-w-σ-t | 2.241 | 2.707 | 109.4 | 2.247 | 2.710 | 109.2 | 2.248 | 2.710 | 109.1 |
| H30···O27 | | | | | | | | | |
| U8 |  |  |  |  |  |  |  |  |  |
| U8-ƞ-d-u-w-τ-t | 2.261 | 2.705 | 107.8 | 2.272 | 2.706 | 107.1 | 2.273 | 2.706 | 107.0 |
| U8-ƞ-d-u-w-μ-t | 2.258 | 2.701 | 107.8 | 2.269 | 2.704 | 107.2 | 2.270 | 2.705 | 107.2 |
| H30···O31 | | | | | | | | | |
| U8 |  |  |  |  |  |  |  |  |  |
| U8-ƞ-d-u-y-κ-t | 2.012 | 2.794 | 138.6 | 2.009 | 2.793 | 138.9 | 2.009 | 2.793 | 138.9 |
| U8-ƞ-d-u-r-ξ-t | 2.010 | 2.792 | 138.7 | 2.004 | 2.790 | 139.2 | 2.004 | 2.790 | 139.2 |
| U8-ƞ-d-u-y-ς-t | 2.015 | 2.806 | 140.0 | 2.002 | 2.802 | 141.0 | 2.001 | 2.802 | 141.1 |
| U8-ƞ-d-u-y-σ-ω | 1.941 | 2.731 | 139.3 | 1.943 | 2.731 | 139.2 | 1.944 | 2.732 | 139.1 |
| U8-ƞ-d-u-y-σ-t | 1.941 | 2.731 | 139.4 | 1.944 | 2.732 | 139.1 | 1.944 | 2.732 | 139.1 |
| U8-ƞ-d-u-r-σ-n | 1.944 | 2.732 | 139.2 | 1.945 | 2.732 | 139.0 | 1.946 | 2.732 | 139.0 |
| U8-ƞ-d-u-w-σ-t | 1.944 | 2.732 | 139.2 | 1.946 | 2.732 | 139.0 | 1.946 | 2.733 | 138.9 |
| H32···O29 | | | | | | | | | |
| U8 |  |  |  |  |  |  |  |  |  |
| U8-ƞ-d-u-r-ξ-t | 2.501 | 2.801 | 98.5 | 2.522 | 2.809 | 97.7 | 2.524 | 2.810 | 97.6 |
| U8-ƞ-d-u-y-ς-t | 2.472 | 2.789 | 99.5 | 2.496 | 2.796 | 98.4 | 2.498 | 2.797 | 98.3 |
| U8-ƞ-d-u-y-κ-t | 2.495 | 2.797 | 98.6 | 2.517 | 2.807 | 97.8 | 2.519 | 2.808 | 97.8 |
| U8-ƞ-d-u-w-μ-t | 2.018 | 2.770 | 135.1 | 2.013 | 2.764 | 134.9 | 2.013 | 2.764 | 134.9 |
| U8-ƞ-d-u-w-τ-t | 2.019 | 2.773 | 135.3 | 2.015 | 2.767 | 135.1 | 2.014 | 2.767 | 135.1 |
| H17···π (C13) | | | | | | | | | |
| U8 |  |  |  |  |  |  |  |  |  |
| U8-ƞ-d-u-y-κ-ω | 2.301 | 3.012 | 131.6 | 2.303 | 3.012 | 131.4 | 2.303 | 3.012 | 131.4 |
| U8-ƞ-d-u-y-κ-t | 2.300 | 3.009 | 131.4 | 2.297 | 3.009 | 131.7 | 2.300 | 3.018 | 132.8 |
| U8-ƞ-d-u-w-μ-t | 2.303 | 3.009 | 131.1 | 2.297 | 3.010 | 131.8 | 2.303 | 3.014 | 133.6 |
| U8-ƞ-d-u-r-ξ-t | 2.300 | 3.008 | 131.3 | 2.297 | 3.009 | 131.7 | 2.300 | 3.013 | 132.9 |
| U8-ƞ-d-u-y-ς-t | 2.323 | 3.018 | 130.0 | 2.315 | 3.018 | 130.8 | 2.323 | 3.018 | 132.7 |
| U8-ƞ-d-u-y-σ-ω | 2.303 | 3.012 | 131.3 | 2.303 | 3.012 | 131.4 | 2.303 | 3.020 | 132.6 |
| U8-ƞ-d-u-y-σ-t | 2.301 | 3.009 | 131.3 | 2.298 | 3.008 | 131.6 | 2.301 | 3.019 | 132.8 |
| U8-ƞ-d-u-r-σ-n | 2.308 | 3.013 | 131.0 | 2.306 | 3.012 | 131.2 | 2.308 | 3.011 | 131.7 |
| U8-ƞ-d-u-w-σ-t | 2.301 | 3.009 | 131.2 | 2.296 | 3.008 | 131.7 | 2.301 | 3.008 | 133.2 |
| U8-ƞ-d-u-w-τ-t | 2.300 | 3.008 | 131.3 | 2.295 | 3.009 | 132.0 | 2.300 | 3.015 | 133.8 |
