# Supplementary material for: Computable properties of selected monomeric acylphloroglucinols with anticancer and/or antimalarial activities and first-approximation docking study
Source: J Mol Model. 2025 Mar 12;31(4):113. doi: 10.1007/s00894-025-06299-7 (PMC11903629; doi:10.1007/s00894-025-06299-7)
Supplement: Supplementary file 32 — (DOCX 46.8 KB) [file 894_2025_6299_MOESM32_ESM.docx]

**Table S18.**

**Length of the IHB (Å)** **in the calculated conformers of the considered ACPL molecules *in vacuo* and in chloroform, acetonitrile and water (respectively denoted as vac, chlrf, actn, aq in the column headings).**

DFT/B3LYP/6-31+G(d,p) and HF/6-31G(d,p) results from full optimisation calculations, respectively denoted as DFT and HF in the columns’ headings.

The various IHBs are considered individually, across the molecules and conformers in which they are present. The molecules are denoted with the symbols listed in table 1, and the conformers with the symbols listed in table 2. For each molecule, the conformers are listed in order of increasing relative energies in the DFT results *in vacuo*.

| Molecules and conformers | Length of the IHB (Å) | | | | | | | |
| --- | --- | --- | --- | --- | --- | --- | --- | --- |
|  | DFT | | | | HF | | | |
|  | vac | chlrf | actn | aq | vac | chlrf | actn | aq |
| H15···O14 | | | | | | | | |
| **U1** |  |  |  |  |  |  |  |  |
| U1-d-r-a | 1.563 | 1.552 | 1.548 | 1.547 | 1.692 | 1.685 | 1.682 | 1.682 |
| U1-d-w-a | 1.576 | 1.558 | 1.552 | 1.552 | 1.714 | 1.687 | 1.683 | 1.683 |
| U1-d-u-r-a | 1.576 | 1.719 | 1.719 | 1.719 | 1.714 | 1.706 | 1.700 | 1.699 |
| U1-d-u-w-a | 1.584 | 1.558 | 1.553 | 1.553 | 1.719 | 1.706 | 1.702 | 1.702 |
|  |  |  |  |  |  |  |  |  |
| **U2** |  |  |  |  |  |  |  |  |
| U2-d-v-a | 1.545 | 1.538 | 1.536 | 1.536 | 1.672 | 1.669 | 1.667 | 1.667 |
| U2-d-x-a | 1.546 | 1.539 | 1.536 | 1.535 | 1.673 | 1.669 | 1.667 | 1.667 |
|  |  |  |  |  |  |  |  |  |
| **U4** |  |  |  |  |  |  |  |  |
| U4-d-ε-r-x-j | 1.539 | 1.533 | 1.530 | 1.530 | 1.665 | 1.660 | 1.658 | 1.665 |
| U4-d-w-x-j | 1.547 | 1.537 | 1.533 | 1.533 | 1.671 | 1.664 | 1.661 | 1.671 |
| U4-d-ε-r-v-j | 1.541 | 1.533 | 1.530 | 1.529 | 1.666 | 1.660 | 1.658 | 1.666 |
| U4-d-w-v-k | 1.546 | 1.535 | 1.531 | 1.531 | 1.671 | 1.664 | 1.661 | 1.671 |
|  |  |  |  |  |  |  |  |  |
| **U5** |  |  |  |  |  |  |  |  |
| U5-d-r-x-j | 1.536 | 1.529 | 1.528 | 1.528 | 1.666 | 1.660 | 1.657 | 1.666 |
| U5-d-w-x-j | 1.541 | 1.531 | 1.527 | 1.527 | 1.670 | 1.662 | 1.658 | 1.658 |
| U5-d-r-v-j | 1.538 | 1.532 | 1.529 | 1.528 | 1.667 | 1.660 | 1.657 | 1.667 |
| U5-d-r-x-k | 1.537 | 1.531 | 1.528 | 1.528 | 1.667 | 1.660 | 1.657 | 1.667 |
| U5-d-w-v-k | 1.546 | 1.535 | 1.530 | 1.530 | 1.671 | 1.662 | 1.658 | 1.671 |
|  |  |  |  |  |  |  |  |  |
| **U6** |  |  |  |  |  |  |  |  |
| U6-d-w-e | 1.566 | 1.552 | 1.546 | 1.546 | 1.691 | 1.686 | 1.683 | 1.682 |
| U6-d-w-g | 1.553 | 1.541 | 1.537 | 1.536 | 1.681 | 1.674 | 1.671 | 1.671 |
| U6-d-w-c | 1.554 | 1.543 | 1.538 | 1.537 | 1.681 | 1.674 | 1.671 | 1.671 |
| U6-d-w-e-u | 1.585 | 1.564 | 1.557 | 1.556 | 1.722 | 1.713 | 1.708 | 1.707 |
| U6-d-w-f | 1.561 | 1.545 | 1.539 | 1.538 | 1.682 | 1.671 | 1.667 | 1.682 |
| U6-d-w-h | 1.538 | 1.525 | 1.520 | 1.519 | 1.663 | 1.663 | 1.661 | 1.661 |
| U6-d-y-f | 1.556 | 1.573 | 1.539 | 1.574 | 1.680 | 1.671 | 1.667 | 1.666 |
| U6-d-m-f | 1.556 | 1.556 | 1.539 | 1.550 | 1.679 | 1.671 | 1.668 | 1.667 |
|  |  |  |  |  |  |  |  |  |
| **U7** |  |  |  |  |  |  |  |  |
| U7-d-r-ᴧ-χ-α-p | 1.575 | 1.574 | 1.572 | 1.571 | 1.703 | 1.696 | 1.691 | 1.691 |
| U7-d-w-ᴧ-χ-α-p | 1.583 | 1.578 | 1.574 | 1.573 | 1.710 | 1.700 | 1.693 | 1.693 |
| U7-d-w-ᴧ-χ-α-q | 1.584 | 1.573 | 1.574 | 1.573 | 1.709 | 1.699 | 1.691 | 1.691 |
| U7-d-w-ᴧ-χ-β-p | 1.583 | 1.556 | 1.574 | 1.574 | 1.710 | 1.701 | 1.697 | 1.697 |
| U7-d-w-χ-α-p | 1.565 | 1.555 | 1.550 | 1.550 | 1.714 | 1.702 | 1.699 | 1.699 |
| U7-d-w-ᴧ-χ-α-p-u | 1.601 | 1.580 | 1.571 | 1.570 | 1.746 | 1.740 | 1.736 | 1.735 |
| U7-d-w-ᴧ-λ-α-q | 1.586 | 1.574 | 1.575 | 1.574 | 1.711 | 1.700 | 1.696 | 1.696 |
| U7-d-w-ᴧ-λ-α-p | 1.584 | 1.536 | 1.574 | 1.538 | 1.711 | 1.701 | 1.694 | 1.694 |
| U7-d-w-γ-χ-p | 1.572 | 1.543 | 1.550 | 1.538 | 1.704 | 1.689 | 1.684 | 1.683 |
|  |  |  |  |  |  |  |  |  |
| **U8** |  |  |  |  |  |  |  |  |
| U8-ƞ-d-u-y-κ-ω | 1.633 | 1.621 | 1.618 | 1.617 | 1.836 | 1.812 | 1.812 | 1.812 |
| U8-ƞ-d-u-y-κ-t | 1.632 | 1.620 | 1.616 | 1.616 | 1.835 | 1.810 | 1.810 | 1.810 |
| U8-ƞ-d-u-w-μ-t | 1.653 | 1.639 | 1.633 | 1.632 | 1.828 | 1.824 | 1.821 | 1.821 |
| U8-d-y-κ-ω | 1.632 | 1.619 | 1.616 | 1.616 | 1.812 | 1.837 | 1.861 | 1.861 |
| U8-ƞ-d-u-r-ξ-t | 1.627 | 1.620 | 1.615 | 1.615 | 1.856 | 1.811 | 1.811 | 1.811 |
| U8-ƞ-d-u-y-ς-t | 1.633 | 1.620 | 1.616 | 1.616 | 1.831 | 1.832 | 1.832 | 1.831 |
| U8-ƞ-d-u-y-δ-ω | 1.631 | 1.620 | 1.617 | 1.617 | 1.839 | 1.811 | 1.811 | 1.811 |
| U8-ƞ-d-u-y-δ-t | 1.631 | 1.619 | 1.616 | 1.616 | 1.838 | 1.809 | 1.809 | 1.809 |
| U8-ƞ-d-u-r-δ-n | 1.634 | 1.627 | 1.623 | 1.623 | 1.808 | 1.812 | 1.812 | 1.812 |
| U8-ƞ-d-u-w-δ-t | 1.652 | 1.638 | 1.632 | 1.632 | 1.826 | 1.822 | 1.819 | 1.819 |
| U8-ƞ-d-u-w-τ-t | 1.656 | 1.639 | 1.632 | 1.632 | 1.831 | 1.823 | 1.819 | 1.819 |
| H17···O14 | | | | | | | | |
| **U2** |  |  |  |  |  |  |  |  |
| U2-s-v-a | 1.570 | 1.554 | 1.548 | 1.696 | 1.696 | 1.686 | 1.682 | 1.682 |
| U2-s-v-u-a | 1.588 | 1.567 | 1.559 | 1.559 | 1.733 | 1.718 | 1.712 | 1.711 |
|  |  |  |  |  |  |  |  |  |
| **U3** |  |  |  |  |  |  |  |  |
| U3-s-x-w-a | 1.558 | 1.546 | 1.542 | 1.541 | 1.692 | 1.683 | 1.680 | 1.680 |
| U3-s-v-w-a | 1.557 | 1.546 | 1.542 | 1.542 | 1.691 | 1.683 | 1.680 | 1.680 |
| U3-s-x-w-b | 1.545 | 1.536 | 1.533 | 1.532 | 1.681 | 1.677 | 1.678 | 1.678 |
| U3-s-x-r-a | 1.565 | 1.550 | 1.545 | 1.544 | 1.697 | 1.686 | 1.683 | 1.682 |
|  |  |  |  |  |  |  |  |  |
| **U6** |  |  |  |  |  |  |  |  |
| U6-s-w-f | 1.563 | 1.548 | 1.543 | 1.542 | 1.691 | 1.681 | 1.678 | 1.678 |
| H23···O32 | | | | | | | | |
| **U4** |  |  |  |  |  |  |  |  |
| U4-d-ε-r-x-j | 1.654 | 1.644 | 1.640 | 1.640 | 1.779 | 1.773 | 1.772 | 1.779 |
| U4-d-w-x-j | 1.647 | 1.639 | 1.637 | 1.636 | 1.766 | 1.767 | 1.767 | 1.766 |
| U4-d-ε-r-v-j | 1.615 | 1.602 | 1.596 | 1.595 | 1.785 | 1.775 | 1.772 | 1.785 |
|  |  |  |  |  |  |  |  |  |
| **U5** |  |  |  |  |  |  |  |  |
| U5-d-r-x-j | 1.657 | 1.648 | 1.643 | 1.643 | 1.758 | 1.752 | 1.750 | 1.758 |
| U5-d-w-x-j | 1.660 | 1.651 | 1.647 | 1.647 | 1.760 | 1.754 | 1.752 | 1.752 |
| U5-d-r-v-j | 1.609 | 1.591 | 1.583 | 1.582 | 1.747 | 1.743 | 1.740 | 1.747 |
| U5-r-x-j | 1.649 | 1.642 | 1.640 | 1.640 | 1.752 | 1.749 | 1.748 | 1.748 |
| H26···O32 | | | | | | | | |
| **U4** |  |  |  |  |  |  |  |  |
| U4-d-ε-r-x-j | 1.694 | 1.686 | 1.683 | 1.682 | 1.814 | 1.812 | 1.811 | 1.814 |
| U4-d-w-x-j | 1.684 | 1.679 | 1.677 | 1.677 | 1.800 | 1.803 | 1.803 | 1.800 |
|  |  |  |  |  |  |  |  |  |
| **U5** |  |  |  |  |  |  |  |  |
| U5-d-r-x-j | 1.675 | 1.663 | 1.659 | 1.658 | 1.776 | 1.769 | 1.767 | 1.776 |
| U5-d-w-x-j | 1.665 | 1.657 | 1.654 | 1.654 | 1.767 | 1.764 | 1.762 | 1.762 |
| U5-d-r-x-k | 1.627 | 1.606 | 1.598 | 1.597 | 1.769 | 1.758 | 1.753 | 1.769 |
| U5-r-x-j | 1.682 | 1.669 | 1.663 | 1.663 | 1.783 | 1.774 | 1.770 | 1.770 |
| H26···O14 | | | | | | | | |
| **U7** |  |  |  |  |  |  |  |  |
| U7-d-r-ᴧ-χ-α-p | 1.930 | 1.879 | 1.860 | 1.851 | 2.069 | 2.016 | 1.991 | 1.989 |
| U7-d-w-ᴧ-χ-α-p | 1.938 | 1.879 | 1.858 | 1.856 | 2.077 | 2.020 | 1.992 | 1.990 |
| U7-d-w-ᴧ-χ-α-q | 1.943 | 1.867 | 1.850 | 1.849 | 2.074 | 2.015 | 1.990 | 1.988 |
| U7-d-w-ᴧ-χ-β-p | 1.937 | 1.875 | 1.855 | 1.854 | 2.077 | 2.019 | 1.998 | 1.996 |
| U7-d-w-ᴧ-χ-α-p-u | 1.979 | 1.885 | 1.854 | 1.858 | 2.128 | 2.060 | 2.036 | 2.034 |
| U7-d-w-χ-α-p | 1.923 | 1.863 | 1.850 | 1.849 | 2.057 | 2.014 | 1.999 | 1.998 |
| H26···O10 | | | | | | | | |
| **U8** |  |  |  |  |  |  |  |  |
| U8-ƞ-d-u-w-μ-t | 2.535 | 2.613 | 2.644 | 2.647 | 2.514 | 2.581 | 2.604 | 2.607 |
| U8-ƞ-d-u-r-ξ-t | 2.554 | 2.562 | 2.559 | 2.558 | 2.535 | 2.540 | 2.538 | 2.537 |
| U8-ƞ-d-u-y-ς-t | 2.632 | 2.681 | 2.691 | 2.692 | 2.593 | 2.633 | 2.641 | 2.642 |
| H26···O27 | | | | | | | | |
| **U8** |  |  |  |  |  |  |  |  |
| U8-ƞ-d-u-y-κ-t | 2.416 | 2.434 | 2.437 | 2.437 | 2.444 | 2.445 | 2.447 | 2.447 |
| U8-ƞ-d-u-y-δ-ω | 2.429 | 2.444 | 2.445 | 2.445 | 2.451 | 2.455 | 2.451 | 2.452 |
| U8-ƞ-d-u-y-δ-t | 2.430 | 2.444 | 2.445 | 2.444 | 2.452 | 2.452 | 2.452 | 2.452 |
| U8-ƞ-d-u-r-δ-n | 2.424 | 2.436 | 2.435 | 2.435 | 2.441 | 2.452 | 2.452 | 2.452 |
| U8-ƞ-d-u-w-δ-t | 2.428 | 2.438 | 2.437 | 2.437 | 2.446 | 2.455 | 2.452 | 2.452 |
| H28···O25 | | | | | | | | |
| **U8** |  |  |  |  |  |  |  |  |
| U8-ƞ-d-u-w-τ-t | 2.371 | 2.419 | 2.441 | 2.442 | 2.374 | 2.424 | 2.448 | 2.450 |
| U8-ƞ-d-u-w-μ-t | 2.402 | 2.422 | 2.428 | 2.428 | 2.408 | 2.428 | 2.434 | 2.434 |
| U8-ƞ-d-u-y-ς-t | 2.353 | 2.383 | 2.396 | 2.397 | 2.371 | 2.395 | 2.404 | 2.405 |
| H28···O29 | | | | | | | | |
| **U8** |  |  |  |  |  |  |  |  |
| U8-ƞ-d-u-r-ξ-t | 2.183 | 2.192 | 2.192 | 2.192 | 2.209 | 2.215 | 2.215 | 2.215 |
| U8-ƞ-d-u-y-κ-t | 2.209 | 2.239 | 2.248 | 2.249 | 2.217 | 2.241 | 2.248 | 2.249 |
| U8-ƞ-d-u-y-δ-ω | 2.204 | 2.234 | 2.244 | 2.245 | 2.213 | 2.238 | 2.245 | 2.246 |
| U8-ƞ-d-u-y-δ-t | 2.204 | 2.234 | 2.244 | 2.245 | 2.214 | 2.238 | 2.245 | 2.246 |
| U8-ƞ-d-u-r-δ-n | 2.202 | 2.237 | 2.248 | 2.249 | 2.213 | 2.240 | 2.249 | 2.249 |
| U8-ƞ-d-u-w-δ-t | 2.203 | 2.234 | 2.244 | 2.245 | 2.214 | 2.241 | 2.247 | 2.248 |
| H30···O27 | | | | | | | | |
| **U8** |  |  |  |  |  |  |  |  |
| U8-ƞ-d-u-w-τ-t | 2.219 | 2.249 | 2.261 | 2.262 | 2.233 | 2.261 | 2.272 | 2.273 |
| U8-ƞ-d-u-w-μ-t | 2.202 | 2.239 | 2.255 | 2.256 | 2.227 | 2.258 | 2.269 | 2.270 |
| H30···O31 | | | | | | | | |
| **U8** |  |  |  |  |  |  |  |  |
| U8-ƞ-d-u-y-κ-t | 1.935 | 1.910 | 1.901 | 1.900 | 2.022 | 2.012 | 2.009 | 2.009 |
| U8-ƞ-d-u-r-ξ-t | 1.944 | 1.908 | 1.896 | 1.895 | 2.025 | 2.010 | 2.004 | 2.004 |
| U8-ƞ-d-u-y-ς-t | 1.977 | 1.926 | 1.907 | 1.906 | 2.052 | 2.015 | 2.002 | 2.001 |
| U8-ƞ-d-u-y-δ-ω | 1.898 | 1.871 | 1.866 | 1.866 | 1.948 | 1.941 | 1.943 | 1.944 |
| U8-ƞ-d-u-y-δ-t | 1.898 | 1.871 | 1.866 | 1.865 | 1.948 | 1.941 | 1.944 | 1.944 |
| U8-ƞ-d-u-r-δ-n | 1.899 | 1.875 | 1.870 | 1.869 | 1.947 | 1.944 | 1.945 | 1.946 |
| U8-ƞ-d-u-w-δ-t | 1.906 | 1.879 | 1.872 | 1.872 | 1.948 | 1.944 | 1.946 | 1.946 |
| H32···O29 | | | | | | | | |
| **U8** |  |  |  |  |  |  |  |  |
| U8-ƞ-d-u-r-ξ-t | 2.481 | 2.501 | 2.522 | 2.524 | 2.429 | 2.501 | 2.522 | 2.524 |
| U8-ƞ-d-u-y-ς-t | 2.469 | 2.472 | 2.496 | 2.498 | 2.409 | 2.472 | 2.496 | 2.498 |
| U8-ƞ-d-u-y-κ-t | 2.617 | 2.495 | 2.517 | 2.519 | 2.409 | 2.495 | 2.517 | 2.519 |
| U8-ƞ-d-u-w-μ-t | 1.992 | 2.018 | 2.013 | 2.013 | 2.036 | 2.018 | 2.013 | 2.013 |
| U8-ƞ-d-u-w-τ-t | 1.997 | 2.019 | 2.015 | 2.014 | 2.037 | 2.019 | 2.015 | 2.014 |
| H17···π (C13) | | | | | | | | |
| **U8** |  |  |  |  |  |  |  |  |
| U8-ƞ-d-u-y-κ-ω | 2.184 | 2.170 | 2.168 | 2.168 | 2.301 | 2.301 | 2.303 | 2.303 |
| U8-ƞ-d-u-y-κ-t | 2.183 | 2.297 | 2.300 | 2.300 | 2.638 | 2.300 | 2.297 | 2.300 |
| U8-ƞ-d-u-w-μ-t | 2.181 | 2.297 | 2.302 | 2.303 | 2.526 | 2.303 | 2.297 | 2.303 |
| U8-ƞ-d-u-r-ξ-t | 2.183 | 2.297 | 2.300 | 2.300 | 2.522 | 2.300 | 2.297 | 2.300 |
| U8-ƞ-d-u-y-ς-t | 2.183 | 2.315 | 2.322 | 2.323 | 2.572 | 2.323 | 2.315 | 2.323 |
| U8-ƞ-d-u-y-δ-ω | 2.183 | 2.303 | 2.305 | 2.303 | 2.594 | 2.303 | 2.303 | 2.303 |
| U8-ƞ-d-u-y-δ-t | 2.181 | 2.298 | 2.301 | 2.301 | 2.596 | 2.301 | 2.298 | 2.301 |
| U8-ƞ-d-u-r-δ-n | 2.193 | 2.306 | 2.308 | 2.308 | 2.515 | 2.308 | 2.306 | 2.308 |
| U8-ƞ-d-u-w-δ-t | 2.172 | 2.296 | 2.301 | 2.301 | 2.519 | 2.301 | 2.296 | 2.301 |
| U8-ƞ-d-u-w-τ-t | 2.180 | 2.295 | 2.300 | 2.300 | 2.520 | 2.300 | 2.295 | 2.300 |
