# Supplementary material for: Computable properties of selected monomeric acylphloroglucinols with anticancer and/or antimalarial activities and first-approximation docking study
Source: J Mol Model. 2025 Mar 12;31(4):113. doi: 10.1007/s00894-025-06299-7 (PMC11903629; doi:10.1007/s00894-025-06299-7)
Supplement: Supplementary file 33 — (DOCX 22.3 KB) [file 894_2025_6299_MOESM33_ESM.docx]

**Table S19.**

**HOMO-LUMO energy gap of the calculated conformers of the considered ACPL molecules *in vacuo* and in chloroform, acetonitrile and water (respectively denoted as vac, chlrf, actn, aq in the column headings).**

DFT/B3LYP/6-31+G(d,p results from full optimisation calculations. For each molecule, the conformers are listed in order of increasing relative energies in the DFT results *in vacuo*.

| Molecules and conformers | HOMO-LUMO energy gap (kcal mol^-1^) | | | |
| --- | --- | --- | --- | --- |
|  | vac | chlrf | actn | aq |
| **U1** |  |  |  |  |
| U1-d-r-a | 109.91 | 108.67 | 108.15 | 109.91 |
| U1-d-w-a | 109.80 | 108.63 | 108.15 | 109.80 |
| U1-d-u-r-a | 103.30 | 104.27 | 104.51 | 103.30 |
| U1-d-u-w-a | 103.31 | 104.34 | 104.56 | 103.31 |
| U1-r-a | 126.82 | 121.88 | 120.06 | 126.82 |
|  |  |  |  |  |
| **U2** |  |  |  |  |
| U2-d-v-a | 99.61 | 99.49 | 99.40 | 99.61 |
| U2-s-v-a | 104.08 | 101.88 | 101.21 | 104.08 |
| U2-s-v-u-a | 95.79 | 96.37 | 96.42 | 95.79 |
| U2-d-x-a | 98.97 | 99.08 | 99.05 | 98.97 |
| U2-x-a | 123.32 | 116.07 | 113.64 | 123.32 |
|  |  |  |  |  |
| **U3** |  |  |  |  |
| U3-s-x-w-a | 100.78 | 99.53 | 98.98 | 100.78 |
| U3-s-v-w-a | 101.50 | 100.03 | 99.77 | 101.50 |
| U3-s-x-w-b | 99.12 | 97.31 | 96.59 | 99.12 |
| U3-s-x-r-a | 103.86 | 101.30 | 100.24 | 103.86 |
| U3-z-x-w | 120.26 | 114.08 | 111.80 | 120.26 |
| U3-v-w-a | 121.23 | 114.63 | 111.95 | 121.23 |
|  |  |  |  |  |
| **U4** |  |  |  |  |
| U4-d-ε-r-x-j | 63.96 | 64.50 | 64.53 | 63.96 |
| U4-d-w-x-j | 68.16 | 65.73 | 64.88 | 68.16 |
| U4-d-ε-r-v-j | 67.85 | 68.37 | 68.27 | 67.85 |
| U4-d-ε-r-x-k | 70.66 | 69.33 | 68.78 | 70.66 |
| U4-d-w-v-k | 79.84 | 76.47 | 74.84 | 79.84 |
| U4-w-v-k | 71.80 | 72.90 | 72.84 | 71.80 |
|  |  |  |  |  |
| **U5** |  |  |  |  |
| U5-d-r-x-j | 91.94 | 91.23 | 90.49 | 91.94 |
| U5-d-w-x-j | 91.03 | 90.60 | 89.98 | 91.03 |
| U5-d-r-v-j | 94.73 | 95.38 | 94.77 | 94.73 |
| U5-d-r-x-k | 93.34 | 94.33 | 94.45 | 93.34 |
| U5-r-x-j | 84.22 | 87.37 | 88.11 | 84.22 |
| U5-d-w-v-k | 96.30 | 101.37 | 99.54 | 96.30 |
|  |  |  |  |  |
| **U6** |  |  |  |  |
| U6-d-w-e | 102.79 | 102.02 | 101.69 | 102.79 |
| U6-d-w-g | 101.13 | 99.97 | 99.51 | 101.13 |
| U6-d-w-c | 101.15 | 99.99 | 99.53 | 101.15 |
| U6-s-w-f | 104.54 | 103.33 | 102.86 | 104.54 |
| U6-d-w-e-u | 96.99 | 98.32 | 98.64 | 96.99 |
| U6-d-w-f | 102.64 | 102.10 | 101.86 | 102.64 |
| U6-d-w-h | 99.76 | 98.75 | 98.34 | 99.76 |
| U6-d-y-f | 101.25 | 102.00 | 101.86 | 101.25 |
| U6-d-m-f | 101.26 | 100.43 | 100.12 | 101.26 |
| U6-w-f | 122.23 | 116.14 | 114.13 | 122.23 |
|  |  |  |  |  |
| **U7** |  |  |  |  |
| U7-d-r-ᴧ-χ-α-p | 93.92 | 95.90 | 96.41 | 93.92 |
| U7-d-w-ᴧ-χ-α-p | 94.16 | 95.98 | 96.37 | 94.16 |
| U7-d-w-ᴧ-χ-α-q | 94.96 | 96.44 | 97.23 | 94.96 |
| U7-d-w-ᴧ-χ-β-p | 95.80 | 97.60 | 98.07 | 95.80 |
| U7-d-w-χ-α-p | 97.66 | 99.29 | 99.64 | 97.66 |
| U7-d-w-ᴧ-χ-α-p-u | 94.06 | 93.94 | 93.71 | 94.06 |
| U7-d-w-ᴧ-λ-α-q | 92.30 | 95.85 | 97.08 | 92.30 |
| U7-d-w-ᴧ-λ-α-p | 92.04 | 95.51 | 96.57 | 92.04 |
| U7-d-w-γ-χ-p | 98.40 | 100.63 | 101.17 | 98.40 |
| U7-w-ᴧ-χ-α-p | 102.01 | 102.06 | 101.70 | 102.01 |
|  |  |  |  |  |
| **U8** |  |  |  |  |
| U8-ƞ-d-u-y-κ-ω | 93.48 | 95.66 | 96.15 | 93.48 |
| U8-ƞ-d-u-y-κ-t | 93.25 | 95.56 | 96.12 | 93.25 |
| U8-ƞ-d-u-w-μ-t | 93.47 | 95.54 | 96.04 | 93.47 |
| U8-d-y-κ-ω | 99.89 | 98.90 | 98.51 | 99.89 |
| U8-ƞ-d-u-r-ξ-t | 93.85 | 95.75 | 96.13 | 93.85 |
| U8-ƞ-d-u-y-ς-t | 93.83 | 95.72 | 96.12 | 93.83 |
| U8-ƞ-d-u-y-δ-ω | 93.05 | 95.50 | 96.11 | 93.05 |
| U8-ƞ-d-u-y-δ-t | 92.82 | 95.41 | 96.08 | 92.82 |
| U8-ƞ-d-u-r-δ-n | 93.00 | 95.41 | 96.01 | 93.00 |
| U8-ƞ-d-u-w-δ-t | 92.53 | 95.29 | 96.00 | 92.53 |
| U8-ƞ-s-u-w-τ-t | 93.16 | 95.45 | 96.03 | 93.16 |
| U8-y-κ-ω | 111.09 | 106.46 | 103.34 | 111.09 |
