# Supplementary material for: Computable properties of selected monomeric acylphloroglucinols with anticancer and/or antimalarial activities and first-approximation docking study
Source: J Mol Model. 2025 Mar 12;31(4):113. doi: 10.1007/s00894-025-06299-7 (PMC11903629; doi:10.1007/s00894-025-06299-7)
Supplement: Supplementary file 34 — (DOCX 23.9 KB) [file 894_2025_6299_MOESM34_ESM.docx]

**Table S20.**

**HOMO-LUMO energy gap of the calculated conformers of the considered ACPL molecules *in vacuo* and in chloroform, acetonitrile and water (respectively denoted as vac, chlrf, actn, aq in the column headings).**

HF/6-31G(d,p) results from full optimisation calculations. For each molecule, the conformers are listed in order of increasing relative energies in the DFT results *in vacuo*.

| Molecules and conformers | HOMO-LUMO energy gap (kcal mol^-1^) | | | |
| --- | --- | --- | --- | --- |
|  | vac | chlrf | actn | aq |
| **U1** |  |  |  |  |
| U1-d-r-a | 267.61 | 266.40 | 265.93 | 267.61 |
| U1-d-w-a | 267.63 | 266.42 | 265.95 | 267.63 |
| U1-d-u-r-a | 260.06 | 261.69 | 262.10 | 260.06 |
| U1-d-u-w-a | 260.39 | 262.04 | 262.42 | 260.39 |
| U1-r-a | 277.56 | 290.18 | 289.08 | 277.56 |
|  |  |  |  |  |
| **U2** |  |  |  |  |
| U2-d-v-a | 250.21 | 250.44 | 250.48 | 250.21 |
| U2-s-v-a | 255.75 | 252.95 | 252.02 | 255.75 |
| U2-s-v-u-a | 245.39 | 246.57 | 246.81 | 245.39 |
| U2-d-x-a | 249.23 | 255.64 | 249.96 | 249.23 |
| U2-x-a | 271.57 | 272.36 | 272.57 | 271.57 |
|  |  |  |  |  |
| **U3** |  |  |  |  |
| U3-s-x-w-a | 251.42 | 250.56 | 250.19 | 251.42 |
| U3-s-v-w-a | 252.52 | 251.23 | 250.72 | 252.52 |
| U3-s-x-w-b | 249.94 | 248.32 | 247.76 | 249.94 |
| U3-s-x-r-a | 255.62 | 252.55 | 251.52 | 255.62 |
| U3-z-x-w | 272.27 | 271.57 | 271.30 | 272.27 |
| U3-v-w-a | 272.86 | 271.88 | 271.50 | 272.86 |
|  |  |  |  |  |
| **U4** |  |  |  |  |
| U4-d-ε-r-x-j | 213.01 | 213.59 | 213.62 | 213.01 |
| U4-d-w-x-j | 215.29 | 214.77 | 213.65 | 215.29 |
| U4-d-ε-r-v-j | 218.26 | 218.33 | 218.13 | 218.26 |
| U4-d-ε-r-x-k | 218.94 | 219.31 | 218.78 | 218.94 |
| U4-d-w-v-k | 228.71 | 226.52 | 224.49 | 228.71 |
| U4-w-v-k | 221.36 | 222.28 | 222.21 | 221.36 |
|  |  |  |  |  |
| **U5** |  |  |  |  |
| U5-d-r-x-j | 235.50 | 235.53 | 235.55 | 235.50 |
| U5-d-w-x-j | 234.31 | 234.46 | 234.36 | 234.31 |
| U5-d-r-v-j | 240.87 | 240.96 | 240.69 | 240.87 |
| U5-d-r-x-k | 239.19 | 239.97 | 239.93 | 239.19 |
| U5-r-x-j | 232.99 | 234.95 | 235.38 | 232.99 |
| U5-d-w-v-k | 247.21 | 247.33 | 246.22 | 247.21 |
|  |  |  |  |  |
| **U6** |  |  |  |  |
| U6-d-w-e | 257.44 | 256.92 | 256.67 | 257.44 |
| U6-d-w-g | 255.97 | 255.03 | 254.64 | 255.97 |
| U6-d-w-c | 255.98 | 255.02 | 254.63 | 255.98 |
| U6-s-w-f | 258.95 | 257.96 | 257.59 | 258.95 |
| U6-d-w-e-u | 251.45 | 253.53 | 254.05 | 251.45 |
| U6-d-w-f | 257.11 | 256.76 | 256.58 | 257.11 |
| U6-d-w-h | 253.70 | 253.18 | 252.87 | 253.70 |
| U6-d-y-f | 254.34 | 253.50 | 253.31 | 254.34 |
| U6-d-m-f | 253.57 | 253.11 | 252.86 | 253.57 |
| U6-w-f | 288.63 | 285.33 | 284.16 | 288.63 |
|  |  |  |  |  |
| **U7** |  |  |  |  |
| U7-d-r-ᴧ-χ-α-p | 247.89 | 250.07 | 250.43 | 247.89 |
| U7-d-w-ᴧ-χ-α-p | 248.17 | 250.14 | 250.38 | 248.17 |
| U7-d-w-ᴧ-χ-α-q | 249.35 | 251.37 | 251.63 | 249.35 |
| U7-d-w-ᴧ-χ-β-p | 250.25 | 252.17 | 252.48 | 250.25 |
| U7-d-w-χ-α-p | 251.26 | 253.32 | 253.88 | 251.26 |
| U7-d-w-ᴧ-χ-α-p-u | 245.77 | 246.57 | 246.84 | 245.77 |
| U7-d-w-ᴧ-λ-α-q | 246.82 | 250.40 | 251.14 | 246.82 |
| U7-d-w-ᴧ-λ-α-p | 246.27 | 249.55 | 250.15 | 246.27 |
| U7-d-w-γ-χ-p | 253.07 | 255.28 | 255.80 | 253.07 |
| U7-w-ᴧ-χ-α-p | 261.28 | 262.09 | 261.94 | 261.28 |
|  |  |  |  |  |
| **U8** |  |  |  |  |
| U8-ƞ-d-u-y-κ-ω | 241.62 | 247.16 | 248.05 | 241.62 |
| U8-ƞ-d-u-y-κ-t | 241.14 | 247.02 | 248.03 | 241.14 |
| U8-ƞ-d-u-w-μ-t | 244.09 | 247.56 | 248.46 | 244.09 |
| U8-d-y-κ-ω | 252.64 | 252.74 | 251.24 | 252.64 |
| U8-ƞ-d-u-r-ξ-t | 244.33 | 247.37 | 248.12 | 244.33 |
| U8-ƞ-d-u-y-ς-t | 242.39 | 245.78 | 246.57 | 242.39 |
| U8-ƞ-d-u-y-δ-ω | 240.20 | 246.89 | 247.97 | 240.20 |
| U8-ƞ-d-u-y-δ-t | 239.68 | 246.79 | 247.97 | 239.68 |
| U8-ƞ-d-u-r-δ-n | 242.87 | 246.84 | 247.94 | 242.87 |
| U8-ƞ-d-u-w-δ-t | 242.53 | 247.14 | 248.39 | 242.53 |
| U8-ƞ-s-u-w-τ-t | 243.77 | 247.41 | 248.44 | 243.77 |
| U8-y-κ-ω | 265.48 | 262.59 | 261.55 | 265.48 |
