# Supplementary material for: Computable properties of selected monomeric acylphloroglucinols with anticancer and/or antimalarial activities and first-approximation docking study
Source: J Mol Model. 2025 Mar 12;31(4):113. doi: 10.1007/s00894-025-06299-7 (PMC11903629; doi:10.1007/s00894-025-06299-7)
Supplement: Supplementary file 35 — (DOCX 23.1 KB) [file 894_2025_6299_MOESM35_ESM.docx]

**Table S21.**

**Dipole moments of the calculated conformers of considered ACPL molecules *in vacuo* and in chloroform, acetonitrile and water (respectively denoted as vac, chlrf, actn, aq in the column headings).**

DFT/B3LYP/6-31+G(d,p results from full optimisation calculations. For each molecule, the conformers are listed in order of increasing relative energies in the DFT results *in vacuo*.

| Molecules and conformers | Dipole moment (Debye) | | | |
| --- | --- | --- | --- | --- |
|  | vac | chlrf | actn | aq |
| **U1** |  |  |  |  |
| U1-d-r-a | 3.786 | 5.143 | 5.622 | 5.666 |
| U1-d-w-a | 6.135 | 8.144 | 8.857 | 8.921 |
| U1-d-u-r-a | 1.612 | 2.222 | 2.452 | 2.474 |
| U1-d-u-w-a | 4.304 | 5.676 | 6.165 | 6.210 |
| U1-r-a | 4.625 | 6.048 | 6.561 | 6.608 |
|  |  |  |  |  |
| **U2** |  |  |  |  |
| U2-d-v-a | 1.845 | 2.598 | 2.882 | 2.909 |
| U2-s-v-a | 6.671 | 9.011 | 9.861 | 8.559 |
| U2-s-v-u-a | 5.847 | 7.785 | 8.493 | 8.559 |
| U2-d-x-a | 4.113 | 5.495 | 6.001 | 6.050 |
| U2-x-a | 4.724 | 5.747 | 4.724 | 4.724 |
|  |  |  |  |  |
| **U3** |  |  |  |  |
| U3-s-x-w-a | 3.693 | 5.050 | 5.543 | 5.588 |
| U3-s-v-w-a | 4.696 | 6.242 | 4.696 | 4.696 |
| U3-s-x-w-b | 3.927 | 5.333 | 5.845 | 5.893 |
| U3-s-x-r-a | 6.253 | 8.244 | 8.952 | 9.017 |
| U3-z-x-w | 3.385 | 4.469 | 4.870 | 4.909 |
| U3-v-w-a | 6.024 | 7.782 | 6.024 | 6.024 |
|  |  |  |  |  |
| **U4** |  |  |  |  |
| U4-d-ε-r-x-j | 4.430 | 5.694 | 6.171 | 6.217 |
| U4-d-w-x-j | 7.767 | 9.821 | 10.590 | 10.666 |
| U4-d-ε-r-v-j | 4.391 | 5.605 | 6.069 | 6.113 |
| U4-d-ε-r-x-k | 5.254 | 7.085 | 7.749 | 7.811 |
| U4-d-w-v-k | 5.324 | 7.283 | 7.924 | 7.982 |
| U4-w-v-k | 3.099 | 3.455 | 3.099 | 3.099 |
|  |  |  |  |  |
| **U5** |  |  |  |  |
| U5-d-r-x-j | 7.600 | 9.993 | 10.833 | 10.913 |
| U5-d-w-x-j | 11.072 | 14.163 | 11.072 | 11.072 |
| U5-d-r-v-j | 7.335 | 10.158 | 11.157 | 11.246 |
| U5-d-r-x-k | 8.327 | 10.866 | 11.809 | 11.897 |
| U5-r-x-j | 3.404 | 4.538 | 3.404 | 3.404 |
| U5-d-w-v-k | 10.501 | 14.033 | 15.321 | 15.441 |
|  |  |  |  |  |
| **U6** |  |  |  |  |
| U6-d-w-e | 6.654 | 8.608 | 9.297 | 9.360 |
| U6-d-w-g | 6.978 | 9.107 | 9.861 | 9.931 |
| U6-d-w-c | 6.951 | 9.052 | 9.799 | 9.867 |
| U6-s-w-f | 5.466 | 6.680 | 7.091 | 7.129 |
| U6-d-w-e-u | 4.002 | 5.378 | 4.002 | 4.002 |
| U6-d-w-f | 6.760 | 8.721 | 9.410 | 9.473 |
| U6-d-w-h | 6.837 | 8.924 | 9.665 | 9.733 |
| U6-d-y-f | 5.139 | 8.806 | 9.410 | 9.475 |
| U6-d-m-f | 5.254 | 6.633 | 7.112 | 7.155 |
| U6-w-f | 6.266 | 7.612 | 8.085 | 8.129 |
|  |  |  |  |  |
| **U7** |  |  |  |  |
| U7-d-r-ᴧ-χ-α-p | 5.061 | 7.415 | 5.061 | 5.061 |
| U7-d-w-ᴧ-χ-α-p | 6.764 | 10.392 | 11.266 | 11.346 |
| U7-d-w-ᴧ-χ-α-q | 7.808 | 10.832 | 13.430 | 14.142 |
| U7-d-w-ᴧ-χ-β-p | 6.757 | 10.595 | 11.449 | 11.526 |
| U7-d-w-χ-α-p | 4.956 | 6.838 | 4.956 | 4.956 |
| U7-d-w-ᴧ-χ-α-p-u | 4.908 | 6.809 | 4.908 | 4.908 |
| U7-d-w-ᴧ-λ-α-q | 6.203 | 8.630 | 11.796 | 11.885 |
| U7-d-w-ᴧ-λ-α-p | 4.792 | 6.569 | 9.116 | 9.180 |
| U7-d-w-γ-χ-p | 5.954 | 8.322 | 9.230 | 9.316 |
| U7-w-ᴧ-χ-α-p | 7.287 | 9.860 | 7.287 | 7.287 |
|  |  |  |  |  |
| **U8** |  |  |  |  |
| U8-ƞ-d-u-y-κ-ω | 1.830 | 2.242 | 1.830 | 1.830 |
| U8-ƞ-d-u-y-κ-t | 2.939 | 3.699 | 3.958 | 3.982 |
| U8-ƞ-d-u-w-μ-t | 4.729 | 6.525 | 7.211 | 7.276 |
| U8-d-y-κ-ω | 1.454 | 1.996 | 1.454 | 1.454 |
| U8-ƞ-d-u-r-ξ-t | 5.659 | 6.792 | 7.154 | 7.186 |
| U8-ƞ-d-u-y-ς-t | 6.050 | 7.218 | 7.586 | 7.618 |
| U8-ƞ-d-u-y-δ-ω | 1.191 | 1.616 | 1.768 | 1.782 |
| U8-ƞ-d-u-y-δ-t | 3.597 | 4.096 | 4.239 | 4.251 |
| U8-ƞ-d-u-r-δ-n | 5.054 | 6.009 | 6.275 | 6.298 |
| U8-ƞ-d-u-w-δ-t | 8.507 | 11.154 | 12.076 | 12.161 |
| U8-ƞ-s-u-w-τ-t | 6.398 | 9.188 | 10.245 | 10.345 |
| U8-y-κ-ω | 1.610 | 1.533 | 1.610 | 1.610 |
