# Supplementary material for: Computable properties of selected monomeric acylphloroglucinols with anticancer and/or antimalarial activities and first-approximation docking study
Source: J Mol Model. 2025 Mar 12;31(4):113. doi: 10.1007/s00894-025-06299-7 (PMC11903629; doi:10.1007/s00894-025-06299-7)
Supplement: Supplementary file 36 — (DOCX 23.1 KB) [file 894_2025_6299_MOESM36_ESM.docx]

**Table S22.**

**Dipole moments of the calculated conformers of considered ACPL molecules *in vacuo* and in chloroform, acetonitrile and water (respectively denoted as vac, chlrf, actn, aq in the column headings).**

HF/6-31G(d,p) results from full optimisation calculations. For each molecule, the conformers are listed in order of increasing relative energies in the DFT results *in vacuo.*

| Molecules and conformers | Dipole moment (Debye) | | | |
| --- | --- | --- | --- | --- |
|  | vac | chlrf | actn | aq |
| **U1** |  |  |  |  |
| U1-d-r-a | 3.618 | 4.666 | 5.014 | 5.046 |
| U1-d-w-a | 6.073 | 7.724 | 8.267 | 8.316 |
| U1-d-u-r-a | 1.353 | 1.818 | 1.994 | 2.011 |
| U1-d-u-w-a | 4.205 | 5.356 | 5.747 | 5.783 |
| U1-r-a | 5.253 | 6.059 | 6.455 | 6.491 |
|  |  |  |  |  |
| **U2** |  |  |  |  |
| U2-d-v-a | 1.770 | 2.363 | 2.572 | 2.591 |
| U2-s-v-a | 6.774 | 8.760 | 9.450 | 9.512 |
| U2-s-v-u-a | 5.860 | 7.537 | 8.124 | 8.178 |
| U2-d-x-a | 4.021 | 6.153 | 5.517 | 5.552 |
| U2-x-a | 4.793 | 5.566 | 5.819 | 5.842 |
|  |  |  |  |  |
| **U3** |  |  |  |  |
| U3-s-x-w-a | 3.474 | 4.537 | 4.902 | 4.935 |
| U3-s-v-w-a | 4.764 | 6.011 | 6.426 | 6.463 |
| U3-s-x-w-b | 3.686 | 4.801 | 5.190 | 5.226 |
| U3-s-x-r-a | 6.154 | 7.770 | 8.306 | 8.355 |
| U3-z-x-w | 3.896 | 4.811 | 5.118 | 5.145 |
| U3-v-w-a | 6.650 | 8.185 | 8.694 | 8.740 |
|  |  |  |  |  |
| **U4** |  |  |  |  |
| U4-d-ε-r-x-j | 4.289 | 5.102 | 5.371 | 5.395 |
| U4-d-w-x-j | 7.666 | 9.182 | 9.656 | 9.699 |
| U4-d-ε-r-v-j | 4.304 | 5.153 | 5.422 | 5.446 |
| U4-d-ε-r-x-k | 4.986 | 6.479 | 6.963 | 7.008 |
| U4-d-w-v-k | 5.532 | 7.235 | 7.785 | 7.834 |
| U4-w-v-k | 2.702 | 2.810 | 2.882 | 2.889 |
|  |  |  |  |  |
| **U5** |  |  |  |  |
| U5-d-r-x-j | 7.381 | 9.293 | 9.934 | 9.989 |
| U5-d-w-x-j | 10.949 | 13.454 | 14.278 | 14.353 |
| U5-d-r-v-j | 7.483 | 9.801 | 10.581 | 10.652 |
| U5-d-r-x-k | 8.157 | 10.306 | 11.025 | 11.090 |
| U5-r-x-j | 3.702 | 4.736 | 5.066 | 5.096 |
| U5-d-w-v-k | 10.777 | 13.742 | 14.735 | 14.824 |
|  |  |  |  |  |
| **U6** |  |  |  |  |
| U6-d-w-e | 6.528 | 8.170 | 8.711 | 8.760 |
| U6-d-w-g | 6.780 | 8.521 | 9.096 | 9.147 |
| U6-d-w-c | 6.757 | 8.488 | 9.060 | 9.112 |
| U6-s-w-f | 4.927 | 5.831 | 6.129 | 6.156 |
| U6-d-w-e-u | 3.996 | 5.170 | 5.560 | 5.595 |
| U6-d-w-f | 6.610 | 8.253 | 8.794 | 8.843 |
| U6-d-w-h | 6.680 | 8.379 | 8.944 | 8.995 |
| U6-d-y-f | 5.184 | 6.755 | 7.367 | 8.843 |
| U6-d-m-f | 5.384 | 6.461 | 6.800 | 6.836 |
| U6-w-f | 6.284 | 7.400 | 7.760 | 7.792 |
|  |  |  |  |  |
| **U7** |  |  |  |  |
| U7-d-r-ᴧ-χ-α-p | 4.556 | 5.792 | 6.230 | 6.271 |
| U7-d-w-ᴧ-χ-α-p | 6.064 | 7.880 | 8.634 | 8.696 |
| U7-d-w-ᴧ-χ-α-q | 7.152 | 9.300 | 10.132 | 10.200 |
| U7-d-w-ᴧ-χ-β-p | 6.001 | 7.876 | 8.524 | 8.583 |
| U7-d-w-χ-α-p | 5.185 | 6.646 | 7.205 | 7.255 |
| U7-d-w-ᴧ-χ-α-p-u | 3.829 | 4.950 | 5.372 | 5.410 |
| U7-d-w-ᴧ-λ-α-q | 5.847 | 7.550 | 8.157 | 8.214 |
| U7-d-w-ᴧ-λ-α-p | 3.882 | 4.966 | 5.414 | 5.453 |
| U7-d-w-γ-χ-p | 6.061 | 8.156 | 8.910 | 8.980 |
| U7-w-ᴧ-χ-α-p | 7.092 | 8.912 | 9.555 | 9.612 |
|  |  |  |  |  |
| **U8** |  |  |  |  |
| U8-ƞ-d-u-y-κ-ω | 3.624 | 2.404 | 2.568 | 2.591 |
| U8-ƞ-d-u-y-κ-t | 5.022 | 3.343 | 3.546 | 3.561 |
| U8-ƞ-d-u-w-μ-t | 4.347 | 5.728 | 6.208 | 6.252 |
| U8-d-y-κ-ω | 4.414 | 1.116 | 5.104 | 5.135 |
| U8-ƞ-d-u-r-ξ-t | 5.406 | 6.278 | 6.548 | 6.571 |
| U8-ƞ-d-u-y-ς-t | 6.317 | 7.323 | 7.664 | 7.694 |
| U8-ƞ-d-u-y-δ-ω | 5.762 | 2.207 | 2.447 | 2.472 |
| U8-ƞ-d-u-y-δ-t | 7.100 | 3.956 | 4.115 | 4.129 |
| U8-ƞ-d-u-r-δ-n | 4.897 | 5.819 | 6.071 | 6.093 |
| U8-ƞ-d-u-w-δ-t | 8.297 | 10.459 | 11.157 | 11.220 |
| U8-ƞ-s-u-w-τ-t | 6.075 | 8.395 | 9.219 | 9.296 |
| U8-y-κ-ω | 2.012 | 1.834 | 1.789 | 1.787 |
