# Supplementary material for: Computable properties of selected monomeric acylphloroglucinols with anticancer and/or antimalarial activities and first-approximation docking study
Source: J Mol Model. 2025 Mar 12;31(4):113. doi: 10.1007/s00894-025-06299-7 (PMC11903629; doi:10.1007/s00894-025-06299-7)
Supplement: Supplementary file 37 — (DOCX 23.0 KB) [file 894_2025_6299_MOESM37_ESM.docx]

**Table S23.**

**Binding score energy (kcal mol^-1^) for the docking of the ACPL molecules considered in this work against the selected biochemical targets.**

Results from the QickProp tool (Schrödinger suite).

When the target biomolecule contains more than one active site, they are indicated under the name of the protein.

(-) indicates the absence of detectable interactions between the biological target and the considered ACPL molecule.

| Molecule considered | Anticancer targets considered | | | | | | | | |
| --- | --- | --- | --- | --- | --- | --- | --- | --- | --- |
|  | EGFR | P13K | | BRAF V600b | | | JAK3 | CDK-2 | Topo I |
|  |  | (B1) | (B2) | (C1) | (C2) | (C3) | (D) |  |  |
|  |  |  |  |  |  |  |  |  |  |
| U1 | -4.142 | -5.183 | – | -8.085 | -8.215 | -4.626 | -4.799 | -6.870 | -8.116 |
| U2 | -7.405 | -6.509 | -5.189 | -8.744 | -8.433 | -6.853 | -6.494 | -7.549 | -7.903 |
| U3 | -6.762 | -5.768 | -5.233 | -9.167 | -8.676 | -4.772 | -6.393 | -6.779 | -8.497 |
| U4 | -8.013 | -6.790 | -6.649 | -11.013 | -10.770 | -5.683 | -7.619 | -6.621 | -9.008 |
| U5 | -7.502 | -10.250 | -6.691 | -10.832 | -9.133 | -5.836 | -8.544 | -7.866 | -9.928 |
| U6 | -6.529 | -5.545 | -4.445 | -8.583 | -7.896 | -5.410 | -6.024 | – | -7.367 |
| U7 | -6.675 | -7.385 | -5.276 | -10.863 | -10.862 | -6.038 | -7.275 | -7.426 | -8.782 |
| U8 | -7.290 | -7.994 | -7.224 | -9.585 | -9.087 | -6.596 | -9.814 | -8.491 | -8.412 |
| Co-crystilised ligands | -11.256 | -8.282 | -5.810 | -13.710 | -13.682 | -3.355 | -9.261 | -9.142 | -7.916 |
|  | Anticancer targets considered | | | | | | | | |
|  | H5P90 | | HER2 | | | | | |  |
|  | (G1) | (G2) | (H1) | (H2) | (H3) | (H4) | (H5) | (H6) |  |
|  |  |  |  |  |  |  |  |  |  |
| U1 | -5.500 | -5.582 | -5.918 | – | – | -4.398 | -4.984 | – |  |
| U2 | -5.898 | -6.116 | -6.602 | – | – | – | -6.801 | – |  |
| U3 | -5.924 | -5.614 | -6.338 | – | – | – | -6.558 | – |  |
| U4 | -6.570 | -7.038 | -10.779 | -5.133 | -4.978 | -4.763 | -10.611 | -5.756 |  |
| U5 | -7.365 | -7.269 | -9.927 | – | -5.837 | -5.147 | -10.500 | -5.514 |  |
| U6 | -5.886 | -5.917 | -8.061 | – | – | -4.823 | -6.801 | – |  |
| U7 | -7.209 | -6.903 | -7.355 | -4.902 | -5.955 | -5.412 | -7.618 | – |  |
| U8 | -9.458 | -8.944 | -10.350 | -6.200 | -5.004 | – | -10.579 | -5.521 |  |
| Co-crystilised ligands | -8.997 | -8.821 | -12.033 | -7.582 | -3.699 | -6.974 | -12.083 | -7.443 |  |
|  | Antimalarial targets considered | | | | | | | | |
|  | PFLDH | PFMDH | | | | | PFPMT |  |  |
|  |  | (M1) | (M2) | (M3) | (M4) | (M5) |  |  |  |
|  |  |  |  |  |  |  |  |  |  |
| U1 | -4.680 | -4.561 | -6.491 | -6.604 | – | -5.838 | – |  |  |
| U2 | -4.621 | -4.616 | -6.495 | -4.958 | -5.200 | -5.419 | – |  |  |
| U3 | -4.705 | -6.842 | -7.027 | -6.483 | -5.657 | -5.737 | – |  |  |
| U4 | -6.333 | – | – | -5.821 | – | – |  |  |  |
| U5 | -6.639 | – | – | -5.748 | – | – |  |  |  |
| U6 | -4.750 | -6.822 | -6.128 | -5.465 | -5.713 | -5.686 | -6.168 |  |  |
| U7 | -6.891 | -5.193 | -6.143 | -7.264 | -5.891 | – | – |  |  |
| U8 | -8.209 | -5.840 | -8.030 | -7.533 | -5.670 | -5.851 | – |  |  |
| Co-crystilised ligands | -4.724 | -7.312 | -7.184 | -9.633 | -7.312 | -7.312 | -10.834 |  |  |
