# Supplementary material for: Computable properties of selected monomeric acylphloroglucinols with anticancer and/or antimalarial activities and first-approximation docking study
Source: J Mol Model. 2025 Mar 12;31(4):113. doi: 10.1007/s00894-025-06299-7 (PMC11903629; doi:10.1007/s00894-025-06299-7)
Supplement: Supplementary file 38 — (DOCX 160 KB) [file 894_2025_6299_MOESM38_ESM.docx]

**Table S 24**

**Detailed information on the interactions revealed by docking studies of the considered ACPL molecules with anticancer activities and the selected biological targets**

When two interactions involve the same residue (e.g., two H-bonds with the same residue), its symbol is written twice.

**a) The targets are listed in the same sequence for each molecule**

| Target | binding energy  (kcal mol^-1^) | H-bond with | Aromatic  H-bond with | Hydrophobic interactions with | π⋅⋅⋅π stacking interactions with | polar interactions with | glycine interactions with | π⋅⋅⋅cation interactions with |
| --- | --- | --- | --- | --- | --- | --- | --- | --- |
| **U1 molecule** | | | | | | | | |
| EGFR | -4.142 | ASN842,  LYS745 | ASN842,  ASP855 | LYS728,  PHE723 |  |  | GLY796 |  |
|  |  |  |  |  |  |  |  |  |
| P13K (B1 ) | -5.183 | PHE473,  ILE420,  LYS421,  LYS421 | ILE420 | PHE473 | LYS421 | ALA612 |  |  |
|  |  |  |  |  |  |  |  |  |
| BRAF V600b (C1) | -8.085 | ASP594,  ILE573 | ILE573 | ALA481,  LYS483,  CYS532,  TRP531, |  | LEU505,  LEU567,  ILE572 |  |  |
|  |  |  |  |  |  |  |  |  |
| BRAF V600b (C2) | -8.215 | ILE573 |  |  |  | ILE572,  ALA481,  GLN530 |  |  |
|  |  |  |  |  |  |  |  |  |
| BRAF V600b (C3) | -4.626 | ILE659,  ASN660 |  |  |  | SER657 |  |  |
|  |  |  |  |  |  |  |  |  |
| JAK3 | -4.799 | ASN954,  LEU956 |  | TYR904,  LEU905,  PRO906,  CYS909 |  |  |  |  |
|  |  |  |  |  |  |  |  |  |
| CDK-2 | -6.870 | LYS89,  GLU8,  HIE84 |  | ASP145,  ALA144,  PHE80,  GLU81,  PHE82,  LEU83,  HIE84,  GLN85,  ASP86,  LEU134,  GLY13 |  |  |  |  |
|  |  |  |  |  |  |  |  |  |
| Topo I | -8.116 | DG C:12,  ASP A:535 |  | TYR A:426,  MET A:428 |  |  |  |  |
|  |  |  |  |  |  |  |  |  |
| H5P90 (G1) | -5.500 | ASN51,  ASP54 |  | ASP93,  ILE96,  GLY97 |  | ASN51,  SER52,  ASP54,  ALA55 |  | LYS58 |
|  |  |  |  |  |  |  |  |  |
| H5P90 (G2) | -5.582 | THR184,  LEU48 |  | LEU48  LEU107 |  | SER52 | ALA55 |  |
|  |  |  |  |  |  |  |  |  |
| HER2 (H1) | -5.918 | ASN850,  ASP845,  PHE731 |  | LEU785,  LYS753,  ILE752,  GLY734 |  | ASN850,  THR798 |  |  |
|  |  |  |  |  |  |  |  |  |
| HER2 (H4) | -4.398 | ASP821,  ASN813,  ASN813,  HIE809 |  |  |  | TYR803,  GLY804,  CYS805,  ASN813 |  |  |
|  |  |  |  |  |  |  |  |  |
| HER2 (H5) | -4.984 | PHE731,  ASP845,  ASN850 |  | LEU796,  VAL797,  LEU800,  LEU852 |  | ASN850 | GLY729,  ALA730 |  |
| **U2 molecule** | | | | | | | | |
| EGFR | -7.405 | ASN842,  MET793,  MET793 |  | ALA743, MET790, LEU792 |  | LEU844,  ARG841 |  |  |
|  |  |  |  |  |  |  |  |  |
| P13K (B1) | -6.509 | TYR867,  ASP841 |  | ILE963,  ASP964 |  | MET953,  ASP950 |  |  |
|  |  |  |  |  |  |  |  |  |
| P13K (B2) | -5.189 | ILE420,  PHE473 |  | ILE420,  LYS421,  GLY599,  GLN600,  GLN601,  TYR608 |  | VAL608 |  | LYS,  LYS |
|  |  |  |  |  |  |  |  |  |
| BRAF V600b (C1) | -8.744 | ASN580,  THR529,  LYS483 |  | LEU505,  PHE583,  PHE595 |  |  |  |  |
|  |  |  |  |  |  |  |  |  |
| BRAF V600b (C2) | -8.433 | ASN580,  SER536,  CYS532, |  | CYS532,  TRP531,  GLN530,  THR529,  ILE527,  ALA481,  PHE595 | PHE595 | NMA596A,  ASN580,  SER536,  SER535 |  |  |
|  |  |  |  |  |  |  |  |  |
| BRAF V600b (C3) | -6.853 | GLU648,  SER579,  ASP576 |  | ILE665,  TYR656,  PRO655,  LEU654,  LEU577,  LYS578 |  | ILE644,  VAL645 |  |  |
|  |  |  |  |  |  |  |  |  |
| JAK3(D) | -6.494 |  |  | CYS909,  GLY908,  LEU905,  TYR904,  ARG953 |  |  | CYS909,  LYS830,  GLY831, |  |
|  |  |  |  |  |  |  |  |  |
| CDK-2 | -7.549 | GLU8,  LEU83,  LEU83 |  | GLN85,  HIE84,  PHE80,  VAL64,  LEU148,  ASP145,  ALA144 | LYS89 |  |  |  |
|  |  |  |  |  |  |  |  |  |
| Topo I | -7.903 | TYR A:426,  ARG A:364 |  | TYR A:426 | DC D:112  DA D:113 |  |  |  |
|  |  |  |  |  |  |  |  |  |
| H5P90 (G1) | -5.898 | LYS58,  ASP93 |  | PHE138,  MET98 |  | LYS58,  ALA55,  ASP54,  SER52,  ASN51,  THR184,  SER113,  LYS112,  ALA111,  ILE110 |  |  |
| H5P90 (G2) | -6.116 | ASN106 |  | THR99,  GLY97,  ILE96,  ASN51,  TYR139,  PHE138,  GLY137 |  | GLY135,  ASP54 | LYS58 |  |
|  |  |  |  |  |  |  |  |  |
| HER2 (H1) | -6.602 | ARG849 |  | LEU852,  ALA751 |  |  |  |  |
|  |  |  |  |  |  |  |  |  |
| HER2 (H5) | -6.801 | ARG849 |  | MET774,  ALA771,  GLU770,  GLY865,  PHE864,  ASP863,  LEU800,  MET801,  CYS805,  PHE10004 |  |  |  |  |
| **U3 molecule** | | | | | | | | |
| EGFR | -6.762 | MET793,  MET793 |  | MET790,  GLN791,  LEU792,  MET793,  PRO794 |  |  |  |  |
|  |  |  |  |  |  |  |  |  |
| P13K (B1) | -5.768 | ASP964,  ASP841,  TYR867,  LYS833 |  | HIS1089,  LEU1090 |  | HIS948 |  |  |
|  |  |  |  |  |  |  |  |  |
| P13K (B2) | -5.233 | PHE473,  LYS425,  ASN639 |  | LYS425 |  | LEU423,  PRO424,  LYS421 |  | LYS421 |
|  |  |  |  |  |  |  |  |  |
| BRAF V600b (C1) | -9.167 | LYS483,  PHE595 |  | LEU597,  PHE595,  ALA481,  LEU514,  ILE513,  PHE583,  TRP531,  ILE527 | PHE595 |  |  |  |
|  |  |  |  |  |  |  |  |  |
| BRAF V600b (C2) | -8.676 | THR529 |  | THR529,  ILE527,  GLY466 |  |  |  |  |
|  |  |  |  |  |  |  |  |  |
| BRAF V600b (C3) | -4.772 | SER579,  GLU648, |  | ALA641,  ILE644,  LEU577 |  | GLU648 |  |  |
|  |  |  |  |  |  |  |  |  |
| JAK3 | -6.393 | GLN827,  PRO906,  LEU905 |  | GLN827,  LEU828,  GLY829,  LYS830,  GLY831 |  | GLY908,  SER907,  PRO906,  VAL836 |  |  |
|  |  |  |  |  |  |  |  |  |
| CDK-2 | -6.779 | GLU8,  HIE84,  LEU83 |  | ILE10,  PHE80,  VAL64,  ALA31,  LYS33,  LEU134 |  | GLN85,  HIE84,  ASN132,  GLN131,  GLY13 |  |  |
|  |  |  |  |  |  |  |  |  |
|  |  |  |  |  |  |  |  |  |
| Topo I | -8.497 | TYR A:426  DA D:114 |  |  |  |  |  |  |
|  |  |  |  |  |  |  |  |  |
| H5P90 (G1) | -5.924 | LYS58,  ASP54,  ASN51,  ASP93 |  | SER52 |  | LYS58,  ALA55,  ASN51,  VAL186,  THR184,  ASP93,  MET98,  LEU107,  ASN106 | PHE138,  GLY137 |  |
|  |  |  |  |  |  |  |  |  |
| H5P90 (G2) | -5.614 | LYS58,  ASP54,  ASP93 |  | ALA55,  ASP54 |  | SER52,  ASN51,  LEU48,  TYR139 | GLY135 |  |
|  |  |  |  |  |  |  |  |  |
| HER2 (H1) | -6.338 | LYS753,  ASP863 |  | THR798,  GLN799,  LEU800,  MET801,  LEU726 |  |  | GLY727,  GLY729,  ARG849 |  |
|  |  |  |  |  |  |  |  |  |
| HER2 (H5) | -6.558 | ASP863,  LYS753 |  | THR798,  GLN799,  LEU800,  MET801,  LEU726 |  |  | GLY804,  CYS805,  GLY727 |  |
| **U4 molecule** | | | | | | | | |
| EGFR | -8.013 | SER797 SER797 |  | LEU792,  ALA743,  MET790,  MET793,  GLY796 |  | SER797 |  |  |
|  |  |  |  |  |  |  |  |  |
| P13K (B1) | -6.790 | ASP950,  LYS833,  VAL882 |  | ILE879,  ILE881,  VAL882 | TYR867 |  |  |  |
|  |  |  |  |  |  |  |  |  |
| P13K (B2) | -6.649 | LYS425 |  | TYR608, |  | LYS425,  PRO424,  LEU423,  ASP422 |  | LYS421,  LYS421 |
|  |  |  |  |  |  |  |  |  |
| BRAF V600b (C1) | -11.013 | ASN581,  CYS532 |  | ASN581,  CYS532 | PHE583 | LYS578 |  |  |
|  |  |  |  |  |  |  |  |  |
| BRAF V600b (C2) | -10.770 | SER535 |  | GLN530,  THR529,  LEU514,  ALA481,  LYS483 | PHE583 |  | HIS539,  SER535 |  |
|  |  |  |  |  |  |  |  |  |
| BRAF V600b (C3) | -5.683 | ASP576,  TYR633,  PRO632 |  |  | TYR633 |  |  |  |
|  |  |  |  |  |  |  |  |  |
| JAK3 | -7.619 | CYS909, ARG911,  ASP912,  LEU828,  TYR904 |  | LEU828,  TYR904,  LEU905 |  |  |  |  |
| CDK-2 | -6.621 | GLN85 |  | ILE10,  LYS20,  VAL18 |  |  | ASP86 |  |
|  |  |  |  |  |  |  |  |  |
| Topo I | -9.008 | ASN A:722,  THR A:718,  DT B:10 |  |  | DT B:10 | THR A:718 |  |  |
|  |  |  |  |  |  |  |  |  |
| H5P90 (G1) | -6.570 | ILE110,  ASN51 |  | VAL186,  ASN51,  ASP54,  SER52,  ALA55 |  | LEU48,  THR184 |  |  |
|  |  |  |  |  |  |  |  |  |
| H5P90 (G2) | -7.038 | ASN51 |  | ALA55,  ASP54,  SER52,  VAL186,  LEU107 |  | ASN51,  LEU48,  ALA111 | A |  |
|  |  |  |  |  |  |  |  |  |
| HER2 (H1) | -10.779 | ARG849,  MET801 |  | LEU852,  THR798,  GLN799,  LEU800,  MET801,  LEU726 |  |  | GLY804,  CYS805,  GLY727,  SER728,  GLY729,  PHE731,  VAL734 |  |
|  |  |  |  |  |  |  |  |  |
| HER2 (H2) | -5.133 | LYS854,  TYR1005 |  | TYR1005,  LEU1008,  LEU1009,  PRO749 |  |  |  |  |
|  |  |  |  |  |  |  |  |  |
| HER2 (H3) | -4.978 | LYS736,  LYS736,  MET801,  ASP808,  GLU812 |  | MET801,  PRO802,  TYR803,  GLY804,  CYS805 |  | GLU812 |  |  |
|  |  |  |  |  |  |  |  |  |
| HER2 (H4) | -4.763 | MET801,  LYS736,  LYS736,  GLU812 |  | LEU800,  MET801,  PRO802,  TYR803,  GLY804 |  |  |  |  |
|  |  |  |  |  |  |  |  |  |
| HER2 (H5) | -10.611 | MET801 ARG849 | ASN850 | MET801, LEU800, GLN799, THR798, LEU726 |  | THR862 | CYS805, GLY804, GLY727, SER728, GLY729, ALA730 |  |
|  |  |  |  |  |  |  |  |  |
| HER2 (H6) | -5.756 | TYR1005 LYS854 |  | TYR1005, LEU1009, LYS854 |  |  |  |  |
| **U5 molecule** | | | | | | | | |
| EGFR | -7.502 | SER797,  SER797 |  | MET790, LEU792,  MET793,  GLY796 |  |  |  |  |
|  |  |  |  |  |  |  |  |  |
| P13K (B1) | -10.250 | VAL882, ASP950, LYS833 | TRP 812 | PRO810, ILE879, ILE881, VAL882, ALA885 | TYR867 |  |  |  |
|  |  |  |  |  |  |  |  |  |
| P13K (B2) | -6.691 | LYS425,  LYS421 |  | TYR608,  LYS425,  PRO424,  LEU423,  ILE420 |  |  |  | LYS420 |
|  |  |  |  |  |  |  |  |  |
| BRAF V600b (C1) | -10.832 | CYS532 |  |  | PHE583 | ASN581 | ILE463,  GLY464 |  |
|  |  |  |  |  |  |  |  |  |
| BRAF V600b (C2) | -9.133 | CYS532,  CYS532 |  | TRP531,  GLN530,  LYS483,  ALA481 | PHE583 |  |  |  |
|  |  |  |  |  |  |  |  |  |
| BRAF V600b (C3) | -5.836 | LEU577 |  | LEU577,  ASP576 |  |  |  |  |
|  |  |  |  |  |  |  |  |  |
| JAK3(D) | -8.544 | LEU905 |  | PRO906,  LEU905,  TYR904,  MET902,  VAL884,  VAL836 |  | LEU828,  GLY829 | GLY831 |  |
|  |  |  |  |  |  |  |  |  |
| CDK-2 | -7.866 | ILE10,  ASP86,  LYS89 |  | GLN85,  LEU83,  PHE82,  ALA31, |  |  |  |  |
|  |  |  |  |  |  |  |  |  |
| Topo I | -9.928 | ASN A:722,  DT B:10, DA D:112 | ASN352 |  | DT B:10 | ASN A:352,  THR A:718,  ASN A:352 |  |  |
|  |  |  |  |  |  |  |  |  |
| H5P90 (G1) | -7.365 | ILE110,  ASN51 |  | ALA55,  ASP54,  LEU107,  VAL150,  VAL186 |  | ALA111,  THR184,  LEU48,  ASN51,  SER52 |  |  |
|  |  |  |  |  |  |  |  |  |
| H5P90 (G2) | -7.269 | SER113,  ASN51 |  | ALA55,  ASP54,  SER52,  VAL186,  TYR139,  PHE138,  VAL136,  ASN106 |  | ASN51,  LEU48,  THR184,  ALA11,  VAL136 |  | LYS58 |
|  |  |  |  |  |  |  |  |  |
| HER2 (H1) | -9.927 | ASN850 |  | MET801,  LEU800,  GLN799 |  | ASN850 | GLY804,  CYS805,  PHE1004,  GLY732,  THR734,  ARG849 |  |
|  |  |  |  |  |  |  |  |  |
| HER2 (H3) | -5.837 | LYS736,  CYS804,  PRO802 |  |  |  |  | PRO802,  MET801 |  |
|  |  |  |  |  |  |  |  |  |
| HER2 (H4) | -5.147 | CYS805,  MET801 |  | GLY804,  PRO802,  MET801, |  |  | VAL734,  LEU852 |  |
|  |  |  |  |  |  |  |  |  |
| HER2 (H5) | -10.500 | MET801,  ARG849 |  | LEU726,  LEU852,  THR798,  GLN799,  LEU800,  MET801 |  |  | ASP863,  PHE731,  GLY804,  CYS805 |  |
|  |  |  |  |  |  |  |  |  |
| HER2 (H6) | -5.514 | LYS854,  TYR1005 |  | TYR1005,  LEU1008,  LEU1009 |  |  |  |  |
| **U6 molecule** | | | | | | | | |
| EGFR | -6.529 | MET793 |  | ALA743,  MET790,  GLN791,  LEU792,  MET793,  PRO794 |  |  |  |  |
|  |  |  |  |  |  |  |  |  |
| P13K (B1) | -5.545 | GLU880 |  | VAL882,  ILE881,  TRP812,  ASP841 |  |  |  |  |
|  |  |  |  |  |  |  |  |  |
| P13K (B2) | -4.445 | LYS421  LEU423 |  | GLN601,  VAL604,  ALA605,  ILE469 |  | TYR608 |  |  |
|  |  |  |  |  |  |  |  |  |
| BRAF V600b (C1) | -8.583 | THR529,  LYS483 |  | ILE463,  CYS532,  TRP,531,  ILE527, LEU597,  PHE595 | PHE595 |  |  |  |
|  |  |  |  |  |  |  |  |  |
| BRAF V600b (C2) | -7.896 | CYS532 |  | ILE463,  LEU514,  PHE583,  CYS532,  TRP531 | GLY466 |  |  |  |
|  |  |  |  |  |  |  |  |  |
| BRAF V600b (C3) | -5.410 | SER579, GLU648 |  | VAL654, ILE644, ALA641, TYR640 |  |  |  |  |
|  |  |  |  |  |  |  |  |  |
| JAK3(D) | -6.024 | GLY829 |  | LEU956,  ASN954  LYS830,  GLY829,  LEY828,  MET902,  GLU903,  TYR904, |  |  | CYS909,  GLY908,  SER907,  PRO906 |  |
|  |  |  |  |  |  |  |  |  |
| Topo I | -7.367 | ASP A:533,  THR A:718 |  |  |  | ILE A:535,  GLN A633,  HIE A632,  ASN A:631,  ALA A:715,  THR A:718,  ASN A:722 |  | LYS A:532 |
|  |  |  |  |  |  |  |  |  |
| H5P90 (G1) | -5.886 | GLY135,  PHE138 |  | LEU107,  ASN106 |  | TYR139,  SER113,  LYS112,  ALA111,  ILE110,  THR109,  THR152 | GLY137,  PHE138 |  |
|  |  |  |  |  |  |  |  |  |
| H5P90 (G2) | -5.917 | THR184 |  | ASN51,  VAL150,  LEU107 |  | MET98,  THR184,  VAL186,  LEU48,  LYS112 |  |  |
|  |  |  |  |  |  |  |  |  |
| HER2 (H1) | -8.061 | GLN799, |  | LEU796,  VAL797,  THR798,  GLN799,  CYS805,  VAL734,  ASP863 |  |  | MET801 |  |
|  |  |  |  |  |  |  |  |  |
| HER2 (H4) | -4.823 | MET801 |  | THR798,  GLN799,  LEU800 |  |  | PRO801,  LEU852 |  |
|  |  |  |  |  |  |  |  |  |
| HER2 (H5) | -6.801 | ALA751,  ASP863 |  | VAL734,  CYS805,  GLY804 |  | VAL797 |  |  |
| **U7 molecule** | | | | | | | | |
| EGFR | -6.675 | ASN842,  LYS745,  MET793 |  | THR854, ASP855,  ALA743,  GLN791 |  |  |  |  |
|  |  |  |  |  |  |  |  |  |
| P13K (B1) | -7.385 | TYR867,  ASP841,  ASP836,  ASP964,  ASP950,  LYS833 |  | PRO810,  PHE965,  TRP812 |  |  |  |  |
|  |  |  |  |  |  |  |  |  |
| P13K (B2) | -5.276 | LYS421,  LYS421,  LYS425 |  |  |  | LYS425,  PRO424,  LEU423’  ASP422,  LYS421,  ILE420 |  | LYS425 |
|  |  |  |  |  |  |  |  |  |
| BRAF V600b (C1) | -10.863 | THR529,  ASP594,  CYS532 |  | PHE583,  LEU597,  ILE463,  CYS532,  TRP531 | PHE583,  PHE595 | ASN580,  PHE583 |  |  |
|  |  |  |  |  |  |  |  |  |
| BRAF V600b (C2) | -10.862 | CYS532,  CYS532,  THR529 |  | CYS532,  TRP531,  GLN530,  ILE527,  ALA481,  PHE595 | PHE595,  PHE595 |  |  |  |
|  |  |  |  |  |  |  |  |  |
| BRAF V600b (C3) | -6.038 | LEU577,  ASP576 |  | ALA641,  LEU577 |  | VAL645,  GLU648,  SER579 |  |  |
|  |  |  |  |  |  |  |  |  |
| JAK3(D) | -7.275 | ASN954,  ALA966 |  | PRO906,  LEU905,  TYR904,  ALA966 |  | LEU828 | LYS830,  GLY831,  ASN832,  SER835,  GLY908,  SER907 |  |
|  |  |  |  |  |  |  |  |  |
| CDK-2 | -7.426 | ASP86,  HIE84 |  | LEU298,  LEU134, |  | ILE10 | GLY11,  GLY13,  THR14,  LYS129,  GLN131 |  |
|  |  |  |  |  |  |  |  |  |
| Topo I | -8.782 | ASN A:722,  THR A:718 |  | LYS A:425,  TYR A:426 | DT B:10 |  |  |  |
|  |  |  |  |  |  |  |  |  |
| H5P90 (G1) | -7.209 | GLY132, ASN106,  ASN51, |  | ALA55,  VAL186, |  |  | VAL136,  GLY137,  PHE138,  TYR139,  ASN51,  LYS58 |  |
|  |  |  |  |  |  |  |  |  |
| H5P90 (G2) | -6.903 | ASP93, ASN51,  PHE138 |  | SER52,  SER113,  GLY135 |  | ASN51,  LEU48 | VAL136,  GLY137,  PHE138 |  |
|  |  |  |  |  |  |  |  |  |
| HER2 (H1) | -7.355 | LYS753,  ASP863,  GLY729 |  | LEU726,  LEU800,  LEU785,  ALA751 |  |  | MET801GLY804 |  |
|  |  |  |  |  |  |  |  |  |
| HER2 (H2) | -4.902 | TYR803,  TYR1005 |  | TYR803,  PRO802,  LEU800,  GLN799,  TYR1005 |  |  |  |  |
|  |  |  |  |  |  |  |  |  |
| HER2 (H3) | -5.955 | MET801,  GLU812 |  |  |  | GLU812,  ASN813 | PRO802 |  |
|  |  |  |  |  |  |  |  |  |
| HER2 (H4) | -5.412 | MET801, MET801, ASP808 | MET801 | CYS805,  ASP808, CYS805, MET801, LEU800 |  | THR862,  ARG849,  ASN850 | GLY805, |  |
|  |  |  |  |  |  |  |  |  |
| HER2 (H5) | -7.618 | ILE752,  LYS753,  ARG849 |  | LEU726,  LEU785,  LEU796,  VAL797,  GLN799,  LEU800 |  |  | GLY804,  CYS805 |  |
| **U8 molecule** | | | | | | | | |
| EGFR | -7.290 | ASP855,  LYS745,  LYS745,  MET793 |  | ASN842,  MET790,  LEU792,  MET793 |  |  |  |  |
|  |  |  |  |  |  |  |  |  |
| P13K (B1) | -7.994 | LYS890,  LYS890,  SER806,  LYS833,  ASP841,  TYR867,  ASP964, |  | ILE831 |  | THR887,  ASP950 |  |  |
|  |  |  |  |  |  |  |  |  |
| P13K (B2) | -7.224 | ARG477,  ASN522,  LEU423,  PHE473 | PHE473,  LYS421 | LEU474,  CYS357,  NMA522A |  |  |  | LYS421 |
|  |  |  |  |  |  |  |  |  |
| BRAF V600b (C1) | -9.585 | ILE463,  GLU501 |  | PHE583,  LEU514 |  |  |  |  |
|  |  |  |  |  |  |  |  |  |
| BRAF V600b (C2) | -9.087 | SER536,  ASN580,  GLY596 |  |  | PHE595,  PHE595 | THR529,  SER535,  SER536,  HIS539,  ASN580 |  |  |
|  |  |  |  |  |  |  |  |  |
| BRAF V600b (C3) | -6.596 | PRO655,  GLU648,  ASP576,  ARG575,  ARG575 |  | ILE644,  VAL645 |  | TYR633 |  |  |
|  |  |  |  |  |  |  |  |  |
| JAK3 | -9.814 | ASP912, DYS909, TYR904 | ASP967, TYR904 | ALA853, LEU828, VAL836, CYS909 |  |  |  |  |
|  |  |  |  |  |  |  |  |  |
| CDK-2 | -8.491 | LEU83, ASP86, GLU8, ASN132 | GLU8 | LEU134, LYS33, ALA31, VAL64, GLY13 |  |  |  | LYS89 |
|  |  |  |  |  |  |  |  |  |
| Topo I | -8.412 | GLU A:356,  ASN A:722 |  |  |  | LYS A:352 |  |  |
|  |  |  |  |  |  |  |  |  |
| H5P90 (G1) | -9.458 | ASN51,  ASN51,  GLY153,  ASP54,  LYS58 | ASP93 | ILE91,  PHE138,  LEU107 |  | MET98,  ASN106 ASN51 | GLY135 |  |
|  |  |  |  |  |  |  |  |  |
| H5P90 (G2) | -8.944 | ASN106,  ASN106, ASN51, LYS58, THR184 | ASP93 | ASN51, SER52, ALA55, LYS58, MET98 |  | SER113, LYS112,  ALA111,  ILE110,  LEU107, ASN106, TYR139, PHE138,  LEU48 |  |  |
|  |  |  |  |  |  |  |  |  |
| HER2 (H1) | -10.350 | ASP863,  ASP863,  SER783,  ALA751,  LYS753 |  | ILE752,  LYS753,  LEU785,  ARG784,  SER783 |  | THR798 |  |  |
|  |  |  |  |  |  |  |  |  |
| HER2 (H2) | -6.200 | ARG713, GLU744, GLN799, LYS860, GLY778, | GLU744 | PRO780, LYS860, HIE858 |  |  | GLY778 |  |
|  |  |  |  |  |  |  |  |  |
| HER2 (H3) | -5.004 | TYR803,  TYR803,  CYS805,  ASP808,  LEU726,  ARG849 |  | ASP808 |  |  | CYS805 |  |
|  |  |  |  |  |  |  |  |  |
| HER2 (H5) | -10.579 | ASP863,  ASP863,  LYS753,  THR798 |  | THR862,  LEU852,  VAL734,  LYS753,  ILE752,  THR798 | PHE864 |  |  |  |
|  |  |  |  |  |  |  |  |  |
| HER2 (H6) | -5.521 | LYS860,  GLY778,  GLN799,  ARG713,  GLU744 |  | PRO780,  LYS860,  HIE858 |  | GLN799 | SER779,  GLY778 |  |

**b) The targets are listed in order of decreasing magnitude of the interaction energy for each molecule**

| Target | binding energy  (kcal mol^-1^) | H-bond with | Aromatic  H-bond with | Hydrophobic interactions with | π⋅⋅⋅π stacking interactions with | polar interactions with | glycine interactions with | π⋅⋅⋅cation interactions with |
| --- | --- | --- | --- | --- | --- | --- | --- | --- |
| **U1 molecule** | | | | | | | | |
| BRAF V600b (C2) | -8.215 | ILE573 |  |  |  | ILE572,  ALA481,  GLN530 |  |  |
|  |  |  |  |  |  |  |  |  |
| Topo I | -8.116 | DG C:12,  ASP A:535 |  | TYR A:426,  MET A:428 |  |  |  |  |
|  |  |  |  |  |  |  |  |  |
| BRAF V600b (C1) | -8.085 | ASP594,  ILE573 | ILE573 | ALA481,  LYS483,  CYS532,  TRP531 |  | LEU505,  LEU567,  ILE572 |  |  |
|  |  |  |  |  |  |  |  |  |
| CDK-2 | -6.870 | LYS89,  GLU8,  HIE84 |  | ASP145,  ALA144,  PHE80,  GLU81,  PHE82,  LEU83,  HIE84,  GLN85,  ASP86,  LEU134,  GLY13 |  |  |  |  |
|  |  |  |  |  |  |  |  |  |
| HER2 (H1) | -5.918 | ASN850,  ASP845,  PHE731 |  | LEU785,  LYS753,  ILE752,  GLY734 |  | ASN850,  THR798 |  |  |
|  |  |  |  |  |  |  |  |  |
| H5P90 (G2) | -5.582 | THR184,  LEU48 |  | LEU48  LEU107 |  | SER52 | ALA55 |  |
|  |  |  |  |  |  |  |  |  |
| H5P90 (G1) | -5.500 | ASN51,  ASP54 |  | ASP93,  ILE96,  GLY97 |  | ASN51,  SER52,  ASP54,  ALA55 |  | LYS58 |
|  |  |  |  |  |  |  |  |  |
| P13K (B1) | -5.183 | PHE473,  ILE420,  LYS421,  LYS421 | ILE420 | PHE473 | LYS421 | ALA612 |  |  |
|  |  |  |  |  |  |  |  |  |
| HER2 (H5) | -4.984 | PHE731,  ASP845,  ASN850 |  | LEU796,  VAL797,  LEU800,  LEU852 |  | ASN850 | GLY729,  ALA730 |  |
|  |  |  |  |  |  |  |  |  |
| JAK3 | -4.799 | ASN954,  LEU956 |  | TYR904,  LEU905,  PRO906,  CYS909 |  |  |  |  |
|  |  |  |  |  |  |  |  |  |
| BRAF V600b (C3) | -4.626 | ILE659,  ASN660 |  |  |  | SER657 |  |  |
|  |  |  |  |  |  |  |  |  |
| HER2 (H4) | -4.398 | ASP821,  ASN813,  ASN813,  HIE809 |  |  |  | TYR803,  GLY804,  CYS805,  ASN813 |  |  |
|  |  |  |  |  |  |  |  |  |
| EGFR | -4.142 | ASN842,  LYS745 | ASN842,  ASP855 | LYS728,  PHE723 |  |  | GLY796 |  |
| **U2 molecule** | | | | | | | | |
| BRAF V600b (C1) | -8.744 | ASN580,  THR529,  LYS483 |  | LEU505,  PHE583,  PHE595 |  |  |  |  |
|  |  |  |  |  |  |  |  |  |
| BRAF V600b (C2) | -8.433 | ASN580,  SER536,  CYS532, |  | CYS532,  TRP531,  GLN530,  THR529,  ILE527,  ALA481,  PHE595 | PHE595 | NMA596A,  ASN580,  SER536,  SER535 |  |  |
|  |  |  |  |  |  |  |  |  |
| Topo I | -7.903 | TYR A:426,  ARG A:364 |  | TYR A:426 | DC D:112  DA D:113 |  |  |  |
|  |  |  |  |  |  |  |  |  |
| CDK-2 | -7.549 | GLU8,  LEU83,  LEU83 |  | GLN85,  HIE84,  PHE80,  VAL64,  LEU148,  ASP145,  ALA144 | LYS89 |  |  |  |
|  |  |  |  |  |  |  |  |  |
| EGFR | -7.405 | ASN842,  MET793,  MET793 |  | ALA743, MET790, LEU792 |  | LEU844,  ARG841 |  |  |
|  |  |  |  |  |  |  |  |  |
| BRAF V600b (C3) | -6.853 | GLU648,  SER579,  ASP576 |  | ILE665,  TYR656,  PRO655,  LEU654,  LEU577,  LYS578 |  | ILE644,  VAL645 |  |  |
|  |  |  |  |  |  |  |  |  |
| HER2 (H5) | -6.801 | ARG849 |  | MET774,  ALA771,  GLU770,  GLY865,  PHE864,  ASP863,  LEU800,  MET801,  CYS805,  PHE10004 |  |  |  |  |
|  |  |  |  |  |  |  |  |  |
| HER2 (H1) | -6.602 | ARG849 |  | LEU852,  ALA751 |  |  |  |  |
|  |  |  |  |  |  |  |  |  |
| P13K (B1) | -6.509 | TYR867,  ASP841 |  | ILE963,  ASP964 |  | MET953,  ASP950 |  |  |
|  |  |  |  |  |  |  |  |  |
|  |  |  |  |  |  |  |  |  |
| P13K (B2) | -5.189 | ILE420,  PHE473 |  | ILE420,  LYS421,  GLY599,  GLN600,  GLN601,  TYR608 |  | VAL608 |  | LYS,  LYS |
|  |  |  |  |  |  |  |  |  |
| JAK3(D) | -6.494 |  |  | CYS909,  GLY908,  LEU905,  TYR904,  ARG953 |  |  | CYS909,  LYS830,  GLY831, |  |
|  |  |  |  |  |  |  |  |  |
| H5P90 (G2) | -6.116 | ASN106 |  | THR99,  GLY97,  ILE96,  ASN51,  TYR139,  PHE138,  GLY137 |  | GLY135,  ASP54 | LYS58 |  |
|  |  |  |  |  |  |  |  |  |
| H5P90 (G1) | -5.898 | LYS58,  ASP93 |  | PHE138,  MET98 |  | LYS58,  ALA55,  ASP54,  SER52,  ASN51,  THR184,  SER113,  LYS112,  ALA111,  ILE110 |  |  |
| **U3 molecule** | | | | | | | | |
| BRAF V600b (C1) | -9.167 | LYS483,  PHE595 |  | LEU597,  PHE595,  ALA481,  LEU514,  ILE513,  PHE583,  TRP531,  ILE527 | PHE595 |  |  |  |
|  |  |  |  |  |  |  |  |  |
| BRAF V600b (C2) | -8.676 | THR529 |  | THR529,  ILE527,  GLY466 |  |  |  |  |
|  |  |  |  |  |  |  |  |  |
| Topo I | -8.497 | TYR A:426  DA D:114 |  |  |  |  |  |  |
|  |  |  |  |  |  |  |  |  |
| CDK-2 | -6.779 | GLU8,  HIE84,  LEU83 |  | ILE10,  PHE80,  VAL64,  ALA31,  LYS33,  LEU134 |  | GLN85,  HIE84,  ASN132,  GLN131,  GLY13 |  |  |
|  |  |  |  |  |  |  |  |  |
| EGFR | -6.762 | MET793,  MET793 |  | MET790,  GLN791,  LEU792,  MET793,  PRO794 |  |  |  |  |
|  |  |  |  |  |  |  |  |  |
| HER2 (H5) | -6.558 | ASP863,  LYS753 |  | THR798,  GLN799,  LEU800,  MET801,  LEU726 |  |  | GLY804,  CYS805,  GLY727 |  |
|  |  |  |  |  |  |  |  |  |
| JAK3 | -6.393 | GLN827,  PRO906,  LEU905 |  | GLN827,  LEU828,  GLY829,  LYS830,  GLY831 |  | GLY908,  SER907,  PRO906,  VAL836 |  |  |
|  |  |  |  |  |  |  |  |  |
| HER2 (H1) | -6.338 | LYS753,  ASP863 |  | THR798,  GLN799,  LEU800,  MET801,  LEU726 |  |  | GLY727,  GLY729,  ARG849 |  |
|  |  |  |  |  |  |  |  |  |
| H5P90 (G1) | -5.924 | LYS58,  ASP54,  ASN51,  ASP93 |  | SER52 |  | LYS58,  ALA55,  ASN51,  VAL186,  THR184,  ASP93,  MET98,  LEU107,  ASN106 | PHE138,  GLY137 |  |
|  |  |  |  |  |  |  |  |  |
| P13K (B1) | -5.768 | ASP964,  ASP841,  TYR867,  LYS833 |  | HIS1089,  LEU1090 |  | HIS948 |  |  |
|  |  |  |  |  |  |  |  |  |
|  |  |  |  |  |  |  |  |  |
|  |  |  |  |  |  |  |  |  |
| P13K (B2) | -5.233 | PHE473,  LYS425,  ASN639 |  | LYS425 |  | LEU423,  PRO424,  LYS421 |  | LYS421 |
|  |  |  |  |  |  |  |  |  |
| H5P90 (G2) | -5.614 | LYS58,  ASP54,  ASP93 |  | ALA55,  ASP54 |  | SER52,  ASN51,  LEU48,  TYR139 | GLY135 |  |
|  |  |  |  |  |  |  |  |  |
| BRAF V600b (C3) | -4.772 | SER579,  GLU648, |  | ALA641,  ILE644,  LEU577 |  | GLU648 |  |  |
| **U4 molecule** | | | | | | | | |
| BRAF V600b (C1) | -11.013 | ASN581,  CYS532 |  | ASN581,  CYS532 | PHE583 | LYS578 |  |  |
|  |  |  |  |  |  |  |  |  |
| HER2 (H1) | -10.779 | ARG849,  MET801 |  | LEU852,  THR798,  GLN799,  LEU800,  MET801,  LEU726 |  |  | GLY804,  CYS805,  GLY727,  SER728,  GLY729,  PHE731,  VAL734 |  |
|  |  |  |  |  |  |  |  |  |
| BRAF V600b (C2) | -10.770 | SER535 |  | GLN530,  THR529,  LEU514,  ALA481,  LYS483 | PHE583 |  | HIS539,  SER535 |  |
|  |  |  |  |  |  |  |  |  |
| HER2 (H5) | -10.611 | MET801 ARG849 | ASN850 | MET801, LEU800, GLN799, THR798, LEU726 |  | THR862 | CYS805, GLY804, GLY727, SER728, GLY729, ALA730 |  |
|  |  |  |  |  |  |  |  |  |
| Topo I | -9.008 | ASN A:722,  THR A:718,  DT B:10 |  |  | DT B:10 | THR A:718 |  |  |
|  |  |  |  |  |  |  |  |  |
| EGFR | -8.013 | SER797 SER797 |  | LEU792,  ALA743,  MET790,  MET793,  GLY796 |  | SER797 |  |  |
|  |  |  |  |  |  |  |  |  |
| JAK3 | -7.619 | CYS909, ARG911,  ASP912,  LEU828,  TYR904 |  | LEU828,  TYR904,  LEU905 |  |  |  |  |
|  |  |  |  |  |  |  |  |  |
| H5P90 (G2) | -7.038 | ASN51 |  | ALA55,  ASP54,  SER52,  VAL186,  LEU107 |  | ASN51,  LEU48,  ALA111 | A |  |
|  |  |  |  |  |  |  |  |  |
| P13K (B1) | -6.790 | ASP950,  LYS833,  VAL882 |  | ILE879,  ILE881,  VAL882 | TYR867 |  |  |  |
|  |  |  |  |  |  |  |  |  |
| P13K (B2) | -6.649 | LYS425 |  | TYR608, |  | LYS425,  PRO424,  LEU423,  ASP422 |  | LYS421,  LYS421 |
|  |  |  |  |  |  |  |  |  |
| CDK-2 | -6.621 | GLN85 |  | ILE10,  LYS20,  VAL18 |  |  | ASP86 |  |
|  |  |  |  |  |  |  |  |  |
| H5P90 (G1) | -6.570 | ILE110,  ASN51 |  | VAL186,  ASN51,  ASP54,  SER52,  ALA55 |  | LEU48,  THR184 |  |  |
|  |  |  |  |  |  |  |  |  |
| HER2 (H6) | -5.756 | TYR1005 LYS854 |  | TYR1005, LEU1009, LYS854 |  |  |  |  |
|  |  |  |  |  |  |  |  |  |
| BRAF V600b (C3) | -5.683 | ASP576,  TYR633,  PRO632 |  |  | TYR633 |  |  |  |
|  |  |  |  |  |  |  |  |  |
| HER2 (H2) | -5.133 | LYS854,  TYR1005 |  | TYR1005,  LEU1008,  LEU1009,  PRO749 |  |  |  |  |
|  |  |  |  |  |  |  |  |  |
| HER2 (H3) | -4.978 | LYS736,  LYS736,  MET801,  ASP808,  GLU812 |  | MET801,  PRO802,  TYR803,  GLY804,  CYS805 |  | GLU812 |  |  |
|  |  |  |  |  |  |  |  |  |
| HER2 (H4) | -4.763 | MET801,  LYS736,  LYS736,  GLU812 |  | LEU800,  MET801,  PRO802,  TYR803,  GLY804 |  |  |  |  |
| **U5 molecule** | | | | | | | | |
| BRAF V600b (C1) | -10.832 | CYS532 |  |  | PHE583 | ASN581 | ILE463,  GLY464 |  |
|  |  |  |  |  |  |  |  |  |
| HER2 (H5) | -10.500 | MET801,  ARG849 |  | LEU726,  LEU852,  THR798,  GLN799,  LEU800,  MET801 |  |  | ASP863,  PHE731,  GLY804,  CYS805 |  |
|  |  |  |  |  |  |  |  |  |
| P13K (B1) | -10.250 | VAL882, ASP950, LYS833 | TRP 812 | PRO810, ILE879, ILE881, VAL882, ALA885 | TYR867 |  |  |  |
|  |  |  |  |  |  |  |  |  |
| Topo I | -9.928 | ASN A:722,  DT B:10, DA D:112 | ASN352 |  | DT B:10 | ASN A:352,  THR A:718,  ASN A:352 |  |  |
|  |  |  |  |  |  |  |  |  |
| HER2 (H1) | -9.927 | ASN850 |  | MET801,  LEU800,  GLN799 |  | ASN850 | GLY804,  CYS805,  PHE1004,  GLY732,  THR734,  ARG849 |  |
|  |  |  |  |  |  |  |  |  |
| BRAF V600b (C2) | -9.133 | CYS532,  CYS532 |  | TRP531,  GLN530,  LYS483,  ALA481 | PHE583 |  |  |  |
|  |  |  |  |  |  |  |  |  |
| JAK3(D) | -8.544 | LEU905 |  | PRO906,  LEU905,  TYR904,  MET902,  VAL884,  VAL836 |  | LEU828,  GLY829 | GLY831 |  |
|  |  |  |  |  |  |  |  |  |
| CDK-2 | -7.866 | ILE10,  ASP86,  LYS89 |  | GLN85,  LEU83,  PHE82,  ALA31, |  |  |  |  |
|  |  |  |  |  |  |  |  |  |
| EGFR | -7.502 | SER797,  SER797 |  | MET790, LEU792,  MET793,  GLY796 |  |  |  |  |
|  |  |  |  |  |  |  |  |  |
| H5P90 (G1) | -7.365 | ILE110,  ASN51 |  | ALA55,  ASP54,  LEU107,  VAL150,  VAL186 |  | ALA111,  THR184,  LEU48,  ASN51,  SER52 |  |  |
|  |  |  |  |  |  |  |  |  |
| H5P90 (G2) | -7.269 | SER113,  ASN51 |  | ALA55,  ASP54,  SER52,  VAL186,  TYR139,  PHE138,  VAL136,  ASN106 |  | ASN51,  LEU48,  THR184,  ALA11,  VAL136 |  | LYS58 |
|  |  |  |  |  |  |  |  |  |
| P13K (B2) | -6.691 | LYS425,  LYS421 |  | TYR608,  LYS425,  PRO424,  LEU423,  ILE420 |  |  |  | LYS420 |
|  |  |  |  |  |  |  |  |  |
| HER2 (H3) | -5.837 | LYS736,  CYS804,  PRO802 |  |  |  |  | PRO802,  MET801 |  |
|  |  |  |  |  |  |  |  |  |
| BRAF V600b (C3) | -5.836 | LEU577 |  | LEU577,  ASP576 |  |  |  |  |
|  |  |  |  |  |  |  |  |  |
| HER2 (H6) | -5.514 | LYS854,  TYR1005 |  | TYR1005,  LEU1008,  LEU1009 |  |  |  |  |
|  |  |  |  |  |  |  |  |  |
| HER2 (H4) | -5.147 | CYS805,  MET801 |  | GLY804,  PRO802,  MET801, |  |  | VAL734,  LEU852 |  |
| **U6 molecule** | | | | | | | | |
| BRAF V600b (C1) | -8.583 | THR529,  LYS483 |  | ILE463,  CYS532,  TRP,531,  ILE527, LEU597,  PHE595 | PHE595 |  |  |  |
|  |  |  |  |  |  |  |  |  |
| HER2 (H1) | -8.061 | GLN799, |  | LEU796,  VAL797,  THR798,  GLN799,  CYS805,  VAL734,  ASP863 |  |  | MET801 |  |
|  |  |  |  |  |  |  |  |  |
| BRAF V600b (C2) | -7.896 | CYS532 |  | ILE463,  LEU514,  PHE583,  CYS532,  TRP531 | GLY466 |  |  |  |
|  |  |  |  |  |  |  |  |  |
| Topo I | -7.367 | ASP A:533,  THR A:718 |  |  |  | ILE A:535,  GLN A633,  HIE A632,  ASN A:631,  ALA A:715,  THR A:718,  ASN A:722 |  | LYS A:532 |
|  |  |  |  |  |  |  |  |  |
| HER2 (H5) | -6.801 | ALA751,  ASP863 |  | VAL734,  CYS805,  GLY804 |  | VAL797 |  |  |
|  |  |  |  |  |  |  |  |  |
| EGFR | -6.529 | MET793 |  | ALA743,  MET790,  GLN791,  LEU792,  MET793,  PRO794 |  |  |  |  |
|  |  |  |  |  |  |  |  |  |
| JAK3(D) | -6.024 | GLY829 |  | LEU956,  ASN954  LYS830,  GLY829,  LEY828,  MET902,  GLU903,  TYR904, |  |  | CYS909,  GLY908,  SER907,  PRO906 |  |
|  |  |  |  |  |  |  |  |  |
| H5P90 (G2) | -5.917 | THR184 |  | ASN51,  VAL150,  LEU107 |  | MET98,  THR184,  VAL186,  LEU48,  LYS112 |  |  |
|  |  |  |  |  |  |  |  |  |
| H5P90 (G1) | -5.886 | GLY135,  PHE138 |  | LEU107,  ASN106 |  | TYR139,  SER113,  LYS112,  ALA111,  ILE110,  THR109,  THR152 | GLY137,  PHE138 |  |
|  |  |  |  |  |  |  |  |  |
| P13K (B1) | -5.545 | GLU880 |  | VAL882,  ILE881,  TRP812,  ASP841 |  |  |  |  |
|  |  |  |  |  |  |  |  |  |
| BRAF V600b (C3) | -5.410 | SER579, GLU648 |  | VAL654, ILE644, ALA641, TYR640 |  |  |  |  |
|  |  |  |  |  |  |  |  |  |
| HER2 (H4) | -4.823 | MET801 |  | THR798,  GLN799,  LEU800 |  |  | PRO801,  LEU852 |  |
|  |  |  |  |  |  |  |  |  |
| P13K (B2) | -4.445 | LYS421  LEU423 |  | GLN601,  VAL604,  ALA605,  ILE469 |  | TYR608 |  |  |
| **U7 molecule** | | | | | | | | |
| BRAF V600b (C1) | -10.863 | THR529,  ASP594,  CYS532 |  | PHE583,  LEU597,  ILE463,  CYS532,  TRP531 | PHE583,  PHE595 | ASN580,  PHE583 |  |  |
|  |  |  |  |  |  |  |  |  |
| BRAF V600b (C2) | -10.862 | CYS532,  CYS532,  THR529 |  | CYS532,  TRP531,  GLN530,  ILE527,  ALA481,  PHE595 | PHE595,  PHE595 |  |  |  |
|  |  |  |  |  |  |  |  |  |
| Topo I | -8.782 | ASN A:722,  THR A:718 |  | LYS A:425,  TYR A:426 | DT B:10 |  |  |  |
|  |  |  |  |  |  |  |  |  |
| HER2 (H5) | -7.618 | ILE752,  LYS753,  ARG849 |  | LEU726,  LEU785,  LEU796,  VAL797,  GLN799,  LEU800 |  |  | GLY804,  CYS805 |  |
|  |  |  |  |  |  |  |  |  |
| CDK-2 | -7.426 | ASP86,  HIE84 |  | LEU298,  LEU134, |  | ILE10 | GLY11,  GLY13,  THR14,  LYS129,  GLN131 |  |
|  |  |  |  |  |  |  |  |  |
|  |  | CYS532 THR529 |  | ILE527, TRP531, CYS532, PHE595, ILE463 | PHE595 |  |  |  |
|  |  |  |  |  |  |  |  |  |
| P13K (B1) | -7.385 | TYR867,  ASP841,  ASP836,  ASP964,  ASP950,  LYS833 |  | PRO810,  PHE965,  TRP812 |  |  |  |  |
|  |  |  |  |  |  |  |  |  |
| HER2 (H1) | -7.355 | LYS753,  ASP863,  GLY729 |  | LEU726,  LEU800,  LEU785,  ALA751 |  |  | MET801GLY804 |  |
|  |  |  |  |  |  |  |  |  |
| JAK3(D) | -7.275 | ASN954,  ALA966 |  | PRO906,  LEU905,  TYR904,  ALA966 |  | LEU828 | LYS830,  GLY831,  ASN832,  SER835,  GLY908,  SER907 |  |
|  |  |  |  |  |  |  |  |  |
| H5P90 (G1) | -7.209 | GLY132, ASN106,  ASN51 |  | ALA55,  VAL186 |  |  | VAL136,  GLY137,  PHE138,  TYR139,  ASN51,  LYS58 |  |
|  |  |  |  |  |  |  |  |  |
| H5P90 (G2) | -6.903 | ASP93, ASN51,  PHE138 |  | SER52,  SER113,  GLY135 |  | ASN51,  LEU48 | VAL136,  GLY137,  PHE138 |  |
|  |  |  |  |  |  |  |  |  |
| EGFR | -6.675 | ASN842,  LYS745,  MET793 |  | THR854, ASP855,  ALA743,  GLN791 |  |  |  |  |
|  |  |  |  |  |  |  |  |  |
| BRAF V600b (C3) | -6.038 | LEU577,  ASP576 |  | ALA641,  LEU577 |  | VAL645,  GLU648,  SER579 |  |  |
|  |  |  |  |  |  |  |  |  |
| HER2 (H3) | -5.955 | MET801,  GLU812 |  |  |  | GLU812,  ASN813 | PRO802 |  |
|  |  |  |  |  |  |  |  |  |
| HER2 (H4) | -5.412 | MET801, MET801, ASP808 | MET801 | CYS805,  ASP808, CYS805, MET801, LEU800 |  | THR862,  ARG849,  ASN850 | GLY805, |  |
|  |  |  |  |  |  |  |  |  |
| P13K (B2) | -5.276 | LYS421,  LYS421,  LYS425 |  |  |  | LYS425,  PRO424,  LEU423’  ASP422,  LYS421,  ILE420 |  | LYS425 |
|  |  |  |  |  |  |  |  |  |
| HER2 (H2) | -4.902 | TYR803,  TYR1005 |  | TYR803,  PRO802,  LEU800,  GLN799,  TYR1005 |  |  |  |  |
| **U8 molecule** | | | | | | | | |
| HER2 (H5) | -10.579 | ASP863,  ASP863,  LYS753,  THR798 |  | THR862,  LEU852,  VAL734,  LYS753,  ILE752,  THR798 | PHE864 |  |  |  |
|  |  |  |  |  |  |  |  |  |
| HER2 (H1) | -10.350 | ASP863,  ASP863,  SER783,  ALA751,  LYS753 |  | ILE752,  LYS753,  LEU785,  ARG784,  SER783 |  | THR798 |  |  |
|  |  |  |  |  |  |  |  |  |
| JAK3 | -9.814 | ASP912, DYS909, TYR904 | ASP967, TYR904 | ALA853, LEU828, VAL836, CYS909 |  |  |  |  |
|  |  |  |  |  |  |  |  |  |
| BRAF V600b (C1) | -9.585 | ILE463,  GLU501 |  | PHE583,  LEU514 |  |  |  |  |
|  |  |  |  |  |  |  |  |  |
| H5P90 (G1) | -9.458 | ASN51,  ASN51,  GLY153,  ASP54,  LYS58 | ASP93 | ILE91,  PHE138,  LEU107 |  | MET98,  ASN106 ASN51 | GLY135 |  |
|  |  |  |  |  |  |  |  |  |
| BRAF V600b (C2) | -9.087 | SER536,  ASN580,  GLY596 |  |  | PHE595,  PHE595 | THR529,  SER535,  SER536,  HIS539,  ASN580 |  |  |
|  |  |  |  |  |  |  |  |  |
| H5P90 (G2) | -8.944 | ASN106,  ASN106, ASN51, LYS58, THR184 | ASP93 | ASN51, SER52, ALA55, LYS58, MET98 |  | SER113, LYS112,  ALA111,  ILE110,  LEU107, ASN106, TYR139, PHE138,  LEU48 |  |  |
|  |  |  |  |  |  |  |  |  |
| CDK-2 | -8.491 | LEU83, ASP86, GLU8, ASN132 | GLU8 | LEU134, LYS33, ALA31, VAL64, GLY13 |  |  |  | LYS89 |
|  |  |  |  |  |  |  |  |  |
| Topo I | -8.412 | GLU A:356,  ASN A:722 |  |  |  | LYS A:352 |  |  |
|  |  |  |  |  |  |  |  |  |
| P13K (B1) | -7.994 | LYS890,  LYS890,  SER806,  LYS833,  ASP841,  TYR867,  ASP964, |  | ILE831 |  | THR887,  ASP950 |  |  |
|  |  |  |  |  |  |  |  |  |
| EGFR | -7.290 | ASP855,  LYS745,  LYS745,  MET793 |  | ASN842,  MET790,  LEU792,  MET793 |  |  |  |  |
|  |  |  |  |  |  |  |  |  |
| P13K (B2) | -7.224 | ARG477,  ASN522,  LEU423,  PHE473 | PHE473,  LYS421 | LEU474,  CYS357,  NMA522A |  |  |  | LYS421 |
|  |  |  |  |  |  |  |  |  |
| BRAF V600b (C3) | -6.596 | PRO655,  GLU648,  ASP576,  ARG575,  ARG575 |  | ILE644,  VAL645 |  | TYR633 |  |  |
|  |  |  |  |  |  |  |  |  |
| HER2 (H2) | -6.200 | ARG713, GLU744, GLN799, LYS860, GLY778, | GLU744 | PRO780, LYS860, HIE858 |  |  | GLY778 |  |
|  |  |  |  |  |  |  |  |  |
| HER2 (H6) | -5.521 | LYS860,  GLY778,  GLN799,  ARG713,  GLU744 |  | PRO780,  LYS860,  HIE858 |  | GLN799 | SER779,  GLY778 |  |
|  |  |  |  |  |  |  |  |  |
| HER2 (H3) | -5.004 | TYR803,  TYR803,  CYS805,  ASP808,  LEU726,  ARG849 |  | ASP808 |  |  | CYS805 |  |
