# Supplementary material for: Computable properties of selected monomeric acylphloroglucinols with anticancer and/or antimalarial activities and first-approximation docking study
Source: J Mol Model. 2025 Mar 12;31(4):113. doi: 10.1007/s00894-025-06299-7 (PMC11903629; doi:10.1007/s00894-025-06299-7)
Supplement: Supplementary file 39 — (DOCX 72.1 KB) [file 894_2025_6299_MOESM39_ESM.docx]

**Table S 25**

**Detailed information on the interactions revealed by docking studies of the considered ACPL molecules with antimalarial activities and the selected biological targets**

When two interactions involve the same residue (e.g., two H-bonds with the same residue), its symbol is written twice.

1. **The targets are listed in the same sequence for each molecule**

| Target | binding energy  (kcal mol^-1^) | H-bond with | Aromatic  H-bond with | Hydrophobic interactions with | π⋅⋅⋅π stacking interactions with | polar interactions with | glycine interactions with | π⋅⋅⋅cation interactions with |
| --- | --- | --- | --- | --- | --- | --- | --- | --- |
| **U1 molecule** | | | | | | | | |
| PFLDH | -4.680 | ASP143,  ASP143,  ASN140 |  | SER245,  PRO246,  TYR247,  ILE31,  PRO250,  LEU163,  VAL138,  ALA194 |  |  |  |  |
|  |  |  |  |  |  |  |  |  |
| PFMDH (M1) | -4.561 |  |  | VAL B:282,  GLU B:283,  HIE B:260 | PHE D:284 | THR B:271 |  |  |
|  |  |  |  |  |  |  |  |  |
| PFMDH (M2) | -6.491 | VAL B:187 |  | LYS B:158,  LEU B:159,  LYS B:160,  VAL B:187,  PHE D:284,  VAL D:282,  PRO D:281 | PHE D:284 | VAL D:169,  VAL B:190 |  |  |
|  |  |  |  |  |  |  |  |  |
| PFMDH (M3) | -6.604 | ASN119,  GLY78 |  | ILE64,  ILE7,  SER100 |  | ILE97,  ALA77,  GLY78,  THR76 | GLY8,  SER9,  GLY10,  GLN11,  ILE12 |  |
| PFMDH (M5) | -5.838 | HIE A:260,  GLU A:283,  LYS C:198 |  | LYS A:273,  ALA A272,  THR A:271,  LEU A:250,  PHE A:251 |  | VAL A,169,  VAL C:188 |  |  |
| **U2 molecule** | | | | | | | | |
| PFLDH | -4.621 | ARG171,  ARG171 |  | ILE31,  LEU167,  ASP168,  ALA236 |  | THR97,  GLY99,  THR101 | HIS195,  GLY196,  MET199,  ACE108 |  |
|  |  |  |  |  |  |  |  |  |
| PFMDH (M1) | -4.616 | TYR B:256,  HIE B:260 |  | THR B:252,  PHE B:251,  LEU B:250,  THR B:271,  ALA B:272,  LYS B:273,  HIE B:260  ASN B:258 |  |  |  |  |
| PFMDH (M2) | -6.495 | LYS B:198,  LEU B:159 |  | HIE D:260,  PRO D:281,  VAL D:281 | LYS D:273 |  |  |  |
|  |  |  |  |  |  |  |  |  |
| PFMDH (M3) | -4.958 |  |  | ILE64,  THR76,  ALA77,  GLY78,  TYR31,  ASP32,  VAL33 |  |  | GLY78,  VAL79,  GLN80,  ILE37 |  |
|  |  |  |  |  |  |  |  |  |
| PFMDH (M4) | -5.200 | LYS A:198,  PRO C:281 |  | PRO C:281,  GLU C:283,  PHE C:284,  LEU C:250 | HIE C:260 | THR C:252 |  |  |
|  |  |  |  |  |  |  |  |  |
| PFMDH (M5) | -5.419 | LYS A:273,  PRO A:281 |  | PRO A:281,  VAL A:282,  PHE A:284,  LEU A:250,  THR A:271 |  | ASN C:188,  VAL A:169,  VAL C:190,  THR A:252 |  |  |
| **U3 molecule** | | | | | | | | |
| PFLDH | -4.705 | VAL233,  ARG171 |  | PRO250,  LEU167,  VAL240,  ASN140,  LYS102 |  |  | ARG171 |  |
|  |  |  |  |  |  |  |  |  |
| PFMDH (M1) | -6.842 | LYS B:273,  PRO B:281  PRO B:281,  LYS D:198 |  | HIE B:280,  PHE D:195,  THR B:271 |  | THR B:252,  PHE B:251 |  |  |
|  |  |  |  |  |  |  |  |  |
| PFMDH (M2) | -7.027 | PRO D:281,  LYS B:198,  LYS B:198 |  | PRO D:281,  VAL D:282,  LYS B: 198,  PHE B:195,  LEU D:250,  PHE D:251 | LYS B:198 |  |  |  |
|  |  |  |  |  |  |  |  |  |
| PFMDH (M3) | -6.483 | THR76,  ASP32 |  |  |  | SER227,  ASN119,  SER118,  GLN80,  GLY78,  ALA77 | GLY8,  SER9,  GLY10,  GLN11,  ILE37 |  |
|  |  |  |  |  |  |  |  |  |
| PFMDH (M4) | -5.657 | PRO C:281,  HIE C:280,  LYS A:198 |  |  |  | ASN A:188,  VAL A:190,  THR C:252,  PHE C:252,  PHE C:251 |  |  |
| PFMDH (M5) | -5.737 | HIE A:280,  LYS A:273 |  | PHE A:284,  VAL C:190,  LEU C :159,  LYS C :198,  MET C :200 |  |  |  |  |
|  |  |  |  |  |  |  |  |  |
| PFPMT | -6.168 | ILE36, ASP128 |  | GLY63, GLY65, ILE90, ASP85, ILE36, TYR27, LEU240 |  |  | GLY243 TRP244 |  |
| **U4 molecule** | | | | | | | | |
| PFLDH | -6.333 | PRO246 |  | LEU167 |  | ASN140,  PRO246 |  |  |
|  |  |  |  |  |  |  |  |  |
| PFMDH (M3) | -5.821 | GLY78,  THR76 |  | VAL33,  VAL34,  ILE37 |  | GLN11,  GLY10,  SER118,  ASN119,  GLN80,  VAL79,  GLY78,  ALA77 |  |  |
| **U5 molecule** | | | | | | | | |
| PFLDH | -6.639 | ARG171 |  | PRO246,  LEU163 |  | ASN140,  PRO141,  VAL142 |  |  |
|  |  |  |  |  |  |  |  |  |
| PFMDH (M3) | -5.748 | ASP32 |  | ALA77,  THR76,  ILE97,  GLY10 |  |  | GLY78,  GLY8 |  |
| **U6 molecule** | | | | | | | | |
| PFLDH | -4.750 | ARG171 |  | ILE31,  ILE254,  PRO250,  TYR247,  PRO246 |  |  | VAL142,  ASP143,  HIS195,  GLY196,  ASN197,  MET199 |  |
|  |  |  |  |  |  |  |  |  |
| PFMDH (M1) | -6.822 | LYS B:273,  HIE B:280 |  | LEU D:159,  LYS B:273,  ALA B:272,  THR B:271,  LEU B:250 |  | PHE B:250,  THR B:252,  PHE B:284,  VAL B:281 |  |  |
|  |  |  |  |  |  |  |  |  |
| PFMDH (M2) | -6.128 | LEU B:159,  LYS D:273 |  | ALA D:272,  PHE D:284,  LEU D :251,  THR D :252,  VAL B :187 |  |  |  |  |
|  |  |  |  |  |  |  |  |  |
| PFMDH (M3) | -5.465 | ASN119,  GLY78 |  |  |  | MET142 | VAL79 |  |
|  |  |  |  |  |  |  |  |  |
| PFMDH (M4) | -5.713 | HIE C:280 |  | VAL A:161,  ALA C:272 |  | THR C:252,  PHE C:251,  LEU C:250,  PHE C:284,  GLU C:283,  VAL C:282 | LYS C:273 |  |
|  |  |  |  |  |  |  |  |  |
| PFMDH (M5) | -5.686 | LYS A:273 |  | PRO A:281,  VAL A:282,  PHE A:284,  VAL C:187 |  | ASN C:188,  VAL C:190,  THR A:252,  PHE A:251,  LEU A:250 |  |  |
| PFPMT | -6.168 | ASP128,  ILE36 |  | LA129,  LEU131 |  | LEU240 | GLY243 | ASP128,  ILE36 |
| **U7 molecule** | | | | | | | | |
| PFLDH | -6.891 | ASN197,  HIS195,  GLU321 |  | VAL240,  PRO250,  LEU167,  ILE31,  LEU163 |  |  | ASN197 |  |
|  |  |  |  |  |  |  |  |  |
| PFMDH (M1) | -5.193 | LYS B:273,  LYS B:273 |  | ALA B:272,  THR B:271,  LEU B:250,  ASN D:188,  VAL D :187,  LEU D :159  LYS D :160,  PRO B :281,  HIE B :280 |  |  |  |  |
|  |  |  |  |  |  |  |  |  |
| PFMDH (M2) | -6.143 | LYS D:273 |  | VAL B:187,  LEU D:250,  PHE D:284,  GLU D:283 |  |  |  |  |
|  |  |  |  |  |  |  |  |  |
| PFMDH (M3) | -7.264 | ILE229,  GLN11 |  | VAL117.  GLY78 |  | GLN80,  GLN11,  ILE12,  SER227 | ILE97 |  |
| PFMDH (M4) | -5.891 | PRO C:280,  ASN A:199 |  | VAL A:161,  VAL C:282,  PRO C:281,  HIE C:280 |  |  |  |  |
| **U8 molecule** | | | | | | | | |
| PFLDH | -8.209 | LYS198, ASN197, ACE108, VAL233 |  | THR232, VAL233, ASN234, ASN235, MET325 |  |  |  |  |
|  |  |  |  |  |  |  |  |  |
| PFMDH (M1) | -5.840 | LYS B:273,  HIE B:260 |  | HIE B:260,  VAL D:161,  LEU B:250,  PHE B:284,  GLU B:282 |  |  |  |  |
|  |  |  |  |  |  |  |  |  |
| PFMDH (M2) | -8.030 | LYS B:160,  LYS B:160, LEU B:159 | LEU B:159 | PHE B:195, VAL B:187, LEU D:250, PHE D:284 |  |  |  |  |
|  |  |  |  |  |  |  |  |  |
| PFMDH (M3) | -7.533 | ASN119, THR76,  ASP32,  ASP32 | GLN80,  GLY78, VAL117 | GLN11 |  | ASN94,  ASN119, SER118, VAL117, MET142,  PRO23 | GLY10, SER9, GLY8, THR76, ALA77, GLY78, VAL79,  GLN80 |  |
|  |  |  |  |  |  |  |  |  |
| PFMDH (M4) | -5.670 | LYS A:198 |  | LEU C:250,  LYS C:273 |  |  |  |  |
|  |  |  |  |  |  |  |  |  |
| PFMDH (M5) | -5.851 | LYS C:198,  LYS C:198,  LYS C :198,  PRO A :281 |  |  |  | ASN C :199,  MET C :200 |  | LYS A :273 |

**b) The targets are listed in order of decreasing magnitude of the interaction energy for each molecule**

| Target | binding energy  (kcal mol^-1^) | H-bond with | Aromatic  H-bond with | Hydrophobic interactions with | π⋅⋅⋅π stacking interactions with | polar interactions with | glycine interactions with | π⋅⋅⋅cation interactions with |
| --- | --- | --- | --- | --- | --- | --- | --- | --- |
| **U1 molecule** | | | | | | | | |
| PFMDH (M3) | -6.604 | ASN119,  GLY78 |  | ILE64,  ILE7,  SER100 |  | ILE97,  ALA77,  GLY78,  THR76 | GLY8,  SER9,  GLY10,  GLN11,  ILE12 |  |
|  |  |  |  |  |  |  |  |  |
| PFMDH (M2) | -6.491 | VAL B:187 |  | LYS B:158,  LEU B:159,  LYS B:160,  VAL B:187,  PHE D:284,  VAL D:282,  PRO D:281 | PHE D:284 | VAL D:169,  VAL B:190 |  |  |
|  |  |  |  |  |  |  |  |  |
| PFMDH (M5) | -5.838 | HIE A:260,  GLU A:283,  LYS C:198 |  | LYS A:273,  ALA A272,  THR A:271,  LEU A:250,  PHE A:251 |  | VAL A,169,  VAL C:188 |  |  |
|  |  |  |  |  |  |  |  |  |
| PFLDH | -4.680 | ASP143,  ASP143,  ASN140 |  | SER245,  PRO246,  TYR247,  ILE31,  PRO250,  LEU163,  VAL138,  ALA194 |  |  |  |  |
|  |  |  |  |  |  |  |  |  |
| PFMDH (M1) | -4.561 |  |  | VAL B:282,  GLU B:283,  HIE B:260 | PHE D:284 | THR B:271 |  |  |
| **U2 molecule** | | | | | | | | |
| PFMDH (M2) | -6.495 | LYS B:198,  LEU B:159 |  | HIE D:260,  PRO D:281,  VAL D:281 | LYS D:273 |  |  |  |
|  |  |  |  |  |  |  |  |  |
| PFMDH (M5) | -5.419 | LYS A:273,  PRO A:281 |  | PRO A:281,  VAL A:282,  PHE A:284,  LEU A:250,  THR A:271 |  | ASN C:188,  VAL A:169,  VAL C:190,  THR A:252 |  |  |
|  |  |  |  |  |  |  |  |  |
| PFMDH (M4) | -5.200 | LYS A:198,  PRO C:281 |  | PRO C:281,  GLU C:283,  PHE C:284,  LEU C:250 | HIE C:260 | THR C:252 |  |  |
|  |  |  |  |  |  |  |  |  |
| PFMDH (M3) | -4.958 |  |  | ILE64,  THR76,  ALA77,  GLY78,  TYR31,  ASP32,  VAL33 |  |  | GLY78,  VAL79,  GLN80,  ILE37 |  |
|  |  |  |  |  |  |  |  |  |
| PFLDH | -4.621 | ARG171,  ARG171 |  | ILE31,  LEU167,  ASP168,  ALA236 |  | THR97,  GLY99,  THR101 | HIS195,  GLY196,  MET199,  ACE108 |  |
|  |  |  |  |  |  |  |  |  |
| PFMDH (M1) | -4.616 | TYR B:256,  HIE B:260 |  | THR B:252,  PHE B:251,  LEU B:250,  THR B:271,  ALA B:272,  LYS B:273,  HIE B:260  ASN B:258 |  |  |  |  |
| **U3 molecule** | | | | | | | | |
| PFMDH (M2) | -7.027 | PRO D:281,  LYS B:198,  LYS B:198 |  | PRO D:281,  VAL D:282,  LYS B: 198,  PHE B:195,  LEU D:250,  PHE D:251 | LYS B:198 |  |  |  |
|  |  |  |  |  |  |  |  |  |
| PFMDH (M1) | -6.842 | LYS B:273,  PRO B:281  PRO B:281,  LYS D:198 |  | HIE B:280,  PHE D:195,  THR B:271 |  | THR B:252,  PHE B:251 |  |  |
|  |  |  |  |  |  |  |  |  |
| PFMDH (M3) | -6.483 | THR76,  ASP32 |  |  |  | SER227,  ASN119,  SER118,  GLN80,  GLY78,  ALA77 | GLY8,  SER9,  GLY10,  GLN11,  ILE37 |  |
|  |  |  |  |  |  |  |  |  |
| PFPMT | -6.168 | ILE36, ASP128 |  | GLY63, GLY65, ILE90, ASP85, ILE36, TYR27, LEU240 |  |  | GLY243 TRP244 |  |
|  |  |  |  |  |  |  |  |  |
| PFMDH (M5) | -5.737 | HIE A:280,  LYS A:273 |  | PHE A:284,  VAL C:190,  LEU C :159,  LYS C :198,  MET C :200 |  |  |  |  |
|  |  |  |  |  |  |  |  |  |
| PFMDH (M4) | -5.657 | PRO C:281,  HIE C:280,  LYS A:198 |  |  |  | ASN A:188,  VAL A:190,  THR C:252,  PHE C:252,  PHE C:251 |  |  |
|  |  |  |  |  |  |  |  |  |
| PFLDH | -4.705 | VAL233,  ARG171 |  | PRO250,  LEU167,  VAL240,  ASN140,  LYS102 |  |  | ARG171 |  |
| **U4 molecule** | | | | | | | | |
| PFLDH | -6.333 | PRO246 |  | LEU167 |  | ASN140,  PRO246 |  |  |
|  |  |  |  |  |  |  |  |  |
| PFMDH (M3) | -5.821 | GLY78,  THR76 |  | VAL33,  VAL34,  ILE37 |  | GLN11,  GLY10,  SER118,  ASN119,  GLN80,  VAL79,  GLY78,  ALA77 |  |  |
| **U5 molecule** | | | | | | | | |
| PFLDH | -6.639 | ARG171 |  | PRO246,  LEU163 |  | ASN140,  PRO141,  VAL142 |  |  |
|  |  |  |  |  |  |  |  |  |
| PFMDH (M3) | -5.748 | ASP32 |  | ALA77,  THR76,  ILE97,  GLY10 |  |  | GLY78,  GLY8 |  |
| **U6 molecule** | | | | | | | | |
| PFMDH (M1) | -6.822 | LYS B:273,  HIE B:280 |  | LEU D:159,  LYS B:273,  ALA B:272,  THR B:271,  LEU B:250 |  | PHE B:250,  THR B:252,  PHE B:284,  VAL B:281 |  |  |
|  |  |  |  |  |  |  |  |  |
| PFPMT | -6.168 | ASP128,  ILE36 |  | LA129,  LEU131 |  | LEU240 | GLY243 | ASP128,  ILE36 |
|  |  |  |  |  |  |  |  |  |
| PFMDH (M2) | -6.128 | LEU B:159,  LYS D:273 |  | ALA D:272,  PHE D:284,  LEU D :251,  THR D :252,  VAL B :187 |  |  |  |  |
|  |  |  |  |  |  |  |  |  |
| PFMDH (M4) | -5.713 | HIE C:280 |  | VAL A:161,  ALA C:272 |  | THR C:252,  PHE C:251,  LEU C:250,  PHE C:284,  GLU C:283,  VAL C:282 | LYS C:273 |  |
|  |  |  |  |  |  |  |  |  |
| PFMDH (M5) | -5.686 | LYS A:273 |  | PRO A:281,  VAL A:282,  PHE A:284,  VAL C:187 |  | ASN C:188,  VAL C:190,  THR A:252,  PHE A:251,  LEU A:250 |  |  |
|  |  |  |  |  |  |  |  |  |
| PFMDH (M3) | -5.465 | ASN119,  GLY78 |  |  |  | MET142 | VAL79 |  |
|  |  |  |  |  |  |  |  |  |
| PFLDH | -4.750 | ARG171 |  | ILE31,  ILE254,  PRO250,  TYR247,  PRO246 |  |  | VAL142,  ASP143,  HIS195,  GLY196,  ASN197,  MET199 |  |
| **U7 molecule** | | | | | | | | |
| PFMDH (M3) | -7.264 | ILE229,  GLN11 |  | VAL117.  GLY78 |  | GLN80,  GLN11,  ILE12,  SER227 | ILE97 |  |
|  |  |  |  |  |  |  |  |  |
| PFLDH | -6.891 | ASN197,  HIS195,  GLU321 |  | VAL240,  PRO250,  LEU167,  ILE31,  LEU163 |  |  | ASN197 |  |
|  |  |  |  |  |  |  |  |  |
| PFMDH (M2) | -6.143 | LYS D:273 |  | VAL B:187,  LEU D:250,  PHE D:284,  GLU D:283 |  |  |  |  |
|  |  |  |  |  |  |  |  |  |
| PFMDH (M4) | -5.891 | PRO C:280,  ASN A:199 |  | VAL A:161,  VAL C:282,  PRO C:281,  HIE C:280 |  |  |  |  |
|  |  |  |  |  |  |  |  |  |
| PFMDH (M1) | -5.193 | LYS B:273,  LYS B:273 |  | ALA B:272,  THR B:271,  LEU B:250,  ASN D:188,  VAL D :187,  LEU D :159  LYS D :160,  PRO B :281,  HIE B :280 |  |  |  |  |
| **U8 molecule** | | | | | | | | |
| PFLDH | -8.209 | LYS198, ASN197, ACE108, VAL233 |  | THR232, VAL233, ASN234, ASN235, MET325 |  |  |  |  |
|  |  |  |  |  |  |  |  |  |
| PFMDH (M2) | -8.030 | LYS B:160,  LYS B:160, LEU B:159 | LEU B:159 | PHE B:195, VAL B:187, LEU D:250, PHE D:284 |  |  |  |  |
|  |  |  |  |  |  |  |  |  |
| PFMDH (M3) | -7.533 | ASN119, THR76,  ASP32,  ASP32 | GLN80,  GLY78, VAL117 | GLN11 |  | ASN94,  ASN119, SER118, VAL117, MET142,  PRO23 | GLY10, SER9, GLY8, THR76, ALA77, GLY78, VAL79,  GLN80 |  |
|  |  |  |  |  |  |  |  |  |
| PFMDH (M5) | -5.851 | LYS C:198,  LYS C:198,  LYS C :198,  PRO A :281 |  |  |  | ASN C :199,  MET C :200 |  | LYS A :273 |
|  |  |  |  |  |  |  |  |  |
| PFMDH (M1) | -5.840 | LYS B:273,  HIE B:260 |  | HIE B:260,  VAL D:161,  LEU B:250,  PHE B:284,  GLU B:282 |  |  |  |  |
|  |  |  |  |  |  |  |  |  |
| PFMDH (M4) | -5.670 | LYS A:198 |  | LEU C:250,  LYS C:273 |  |  |  |  |
