# Supplementary material for: Computable properties of selected monomeric acylphloroglucinols with anticancer and/or antimalarial activities and first-approximation docking study
Source: J Mol Model. 2025 Mar 12;31(4):113. doi: 10.1007/s00894-025-06299-7 (PMC11903629; doi:10.1007/s00894-025-06299-7)
Supplement: Supplementary file 40 — (DOCX 15.6 KB) [file 894_2025_6299_MOESM40_ESM.docx]

**Table S26.**

**Values of the properties considered by Lipinski’s rule-of-five for the molecules considered in this work.**

The properties are the molecular mass (MW), the maximum number of H-bond donors (HBD), the maximum number of H-bond acceptors (HBA) and the predicted octanol/water partition coefficient (QPlogP_o/w_). The values were calculated using QikProp [73]. The row under the names of the properties shows the criteria set by Lipinsky rule.

| Molecule considered | MW  (a.m.u.) | HBD | HBA | QPlogP_o/w_ | Number of violations of Lipinsky rule |
| --- | --- | --- | --- | --- | --- |
|  | < 500 | < 5 | < 10 | < 5 |  |
|  |  |  |  |  |  |
| U1 | 390.6 | 1.0 | 2.3 | 6.7 | 1.0 |
| U2 | 426.5 | 1.0 | 3.8 | 5.5 | 1.0 |
| U3 | 426.5 | 2.0 | 4.8 | 4.8 | 0.0 |
| U4 | 434.4 | 1.0 | 6.8 | 2.5 | 0.0 |
| U5 | 420.4 | 1.0 | 4.8 | 3.4 | 0.0 |
| U6 | 292.4 | 0.0 | 2.3 | 4.5 | 0.0 |
| U7 | 456.5 | 3.0 | 4.5 | 4.7 | 0.0 |
| U8 | 252.3 | 0.0 | 3.3 | 1.9 | 0.0 |
